# Amoebal Endosymbiont *Parachlamydia acanthamoebae* Bn_9_ Can Grow in Immortal Human Epithelial HEp-2 Cells at Low Temperature; An *In Vitro* Model System to Study Chlamydial Evolution

**DOI:** 10.1371/journal.pone.0116486

**Published:** 2015-02-02

**Authors:** Chikayo Yamane, Tomohiro Yamazaki, Shinji Nakamura, Junji Matsuo, Kasumi Ishida, Sumire Yamazaki, Satoshi Oguri, Natsumi Shouji, Yasuhiro Hayashi, Mitsutaka Yoshida, Yimin, Hiroyuki Yamaguchi

**Affiliations:** 1 Department of Medical Laboratory Science, Faculty of Health Sciences, Hokkaido University, North-12, West-5, Kita-ku, Sapporo, Japan; 2 Research Fellow of Japan Society for the Promotion of Science, Kojimachi Business Center Building, 5-3-1 Kojimachi, Chiyoda-ku, Tokyo, Japan; 3 Division of Biomedical Imaging Research, Juntendo University Graduate School of Medicine, 2-1-1 Hongo, Bunkyo-ku, Tokyo, Japan; 4 Hokkaido University Hospital, North-14, West-5, Kita-ku, Sapporo, Japan; 5 Division of Ultrastructural Research, Juntendo University Graduate School of Medicine, 2-1-1 Hongo, Bunkyo-ku, Tokyo, Japan; 6 Department of Advanced Medicine, Graduate School of Medicine, Hokkaido University, Nishi-7, Kita-15, Kita-ku, Sapporo, Japan; University of California Merced, UNITED STATES

## Abstract

Ancient chlamydiae diverged into pathogenic and environmental chlamydiae 0.7–1.4 billion years ago. However, how pathogenic chlamydiae adapted to mammalian cells that provide a stable niche at approximately 37°C, remains unknown, although environmental chlamydiae have evolved as endosymbionts of lower eukaryotes in harsh niches of relatively low temperatures. Hence, we assessed whether an environmental chlamydia, *Parachlamydia* Bn_9_, could grow in human HEp-2 cells at a low culture temperature of 30°C. The assessment of inclusion formation by quantitative RT-PCR revealed that the numbers of bacterial inclusion bodies and the transcription level of *16SrRNA* significantly increased after culture at 30°C compared to at 37°C. Confocal microscopy showed that the bacteria were located close to HEp-2 nuclei and were actively replicative. Transmission electron microscopy also revealed replicating bacteria consisting of reticular bodies, but with a few elementary bodies. Cytochalasin D and rifampicin inhibited inclusion formation. Lactacystin slightly inhibited bacterial inclusion formation. KEGG analysis using a draft genome sequence of the bacteria revealed that it possesses metabolic pathways almost identical to those of pathogenic chlamydia. Interestingly, comparative genomic analysis with pathogenic chlamydia revealed that the *Parachlamydia* similarly possess the genes encoding Type III secretion system, but lacking genes encoding inclusion membrane proteins (IncA to G) required for inclusion maturation. Taken together, we conclude that ancient chlamydiae had the potential to grow in human cells, but overcoming the thermal gap was a critical event for chlamydial adaptation to human cells.

## Introduction

The obligate intracellular bacteria, chlamydiae, have successfully adapted to several distinct hosts. Ancient chlamydiae diverged into pathogenic and environmental species 0.7–1.4 billion years ago [1, 2] and pathogenic chlamydiae had successfully adapted to mammals, including humans, in a stable niche of approximately 37°C [3–7]. All pathogenic chlamydiae are well-known human pathogens, such as *Chlamydia trachomatis* or *C. pneumoniae*; *C. trachomatis* is the leading cause of ocular infection resulting into trachoma, a preventable blindness [8], and sexually transmitted disease [9]. *C. pneumoniae* is an important cause of community-acquired pneumonia [10], possibly responsible for several chronic diseases such as atherosclerosis and asthma [11].

Environmental chlamydiae have adapted to lower eukaryotes, including free-living amoebae such as *Acanthamoeba*, living in harsh environments at relatively low temperatures [12]. In fact, we have previously shown that environmental chlamydiae (*Protochlamydia* R18, *Parachlamydia* Bn_9_) failed to grow in the immortalized epithelial cell line, HEp-2, at 37°C [13–15]. However, in contrast to these findings, we have also found that an amoebal endosymbiont, *Protochlamydia*, found in HS-T3 *Acanthamoeba* isolated from a hot spring, successfully adapted to HEp-2 cells at 37°C with active replication [16]. More importantly, while the evolution of pathogenic chlamydiae has involved a decrease in genome size to approximately 1.0–1.2 Mb, which may be a strategy to evade the host immune network, resulting in a shift to parasitic energy and metabolic requirements [17], the genomes of representative environmental chlamydiae are not decreasing and have stabilized at 2.4–3.0 Mb [12, 18, 19]. These findings indicate that ancient pathogenic features are strongly selected for in environmental chlamydiae. Thus, ancient pathogenic chlamydiae could not readily grow in mammalian cells within a stable niche of approximately 37°C and there might be a significant temperature gap that ancient pathogenic chlamydiae had to overcome to successfully adapt to mammals, including humans.

However, it is well known that both types of chlamydiae have a similar intracellular developmental cycle after sequestration by the plasma membrane into so-called inclusion bodies. The developmental cycle is defined by two distinct forms: the elementary body (EB), which is the infectious form to the host cells, and the reticulate body (RB), which is the replicative form in cells [20]. This is strictly operated using the type III secretion system, which is well conserved in both pathogenic and environmental species [21, 22], although effector molecules have few similarities among chlamydiae [23]. Meanwhile, it has been known that chlamydial proteosome/protease–like activity factor (CPAF) is an essential effector for the suppression of apoptosis of infected cells and for avoiding the immune response [24, 25] and for providing inclusion membrane with flexibility for normally maturing chlamydiae [26]. Thus, environmental chlamydiae are likely to be a living fossil filled with clues that may reveal how pathogenic chlamydiae could successfully adapt to become pathogens of mammalian cells.

In this study, we therefore established *Parachlamydia* Bn_9_ infected HEp-2 cells at low temperature 30°C as an *in vitro* model to study chlamydial evolution.

## Results

### *Parachlamydia* Bn_9_ failed to grow in host amoebae at 37°C

**Figure 1:**
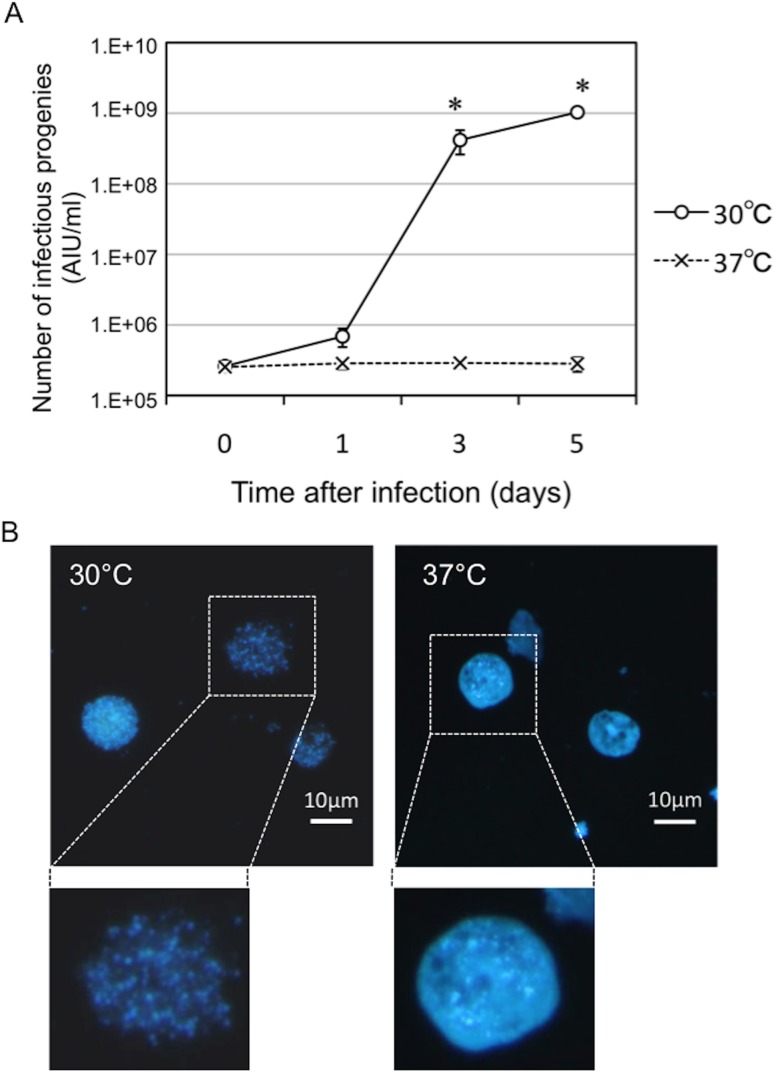
Changes in the growth rate of *Parachlamydia* Bn_9_ in C3 amoebae at 30 or 37°C. The amoebae were infected with the bacteria (MOI 10), and then incubated for 5 days at 30 or 37°C. The bacterial growth was assessed by conventional fluorescence microscopy. (A) Number of infection progenies in the C3 amoebae infected with *Parachlamydia* Bn_9_. The bacterial numbers were estimated by AIU assays [13]. Data are the means ± SD from at least three experiments. *P < 0.05 vs. each culture (30 or 37°C) at immediately after infection. (B) Representative DAPI images of the C3 amoebae infected with *Parachlamydia* Bn_9_ at 3 days after infection. Squares surrounded by dotted lines are enlarged below.

To confirm that *Parachlamydia* Bn_9_ did not adapt at 37°C, we used a previously established amoeba-infectious unit (AIU) assay [13] with 4’,6-diamidino-2-phenylindole (DAPI) staining to determine whether the bacteria could grow in C3 amoebae at 37°C. A failure of growth was seen at 37°C, while at 30°C the number of infectious bacterial progeny was significantly greater (Fig. 1A). DAPI staining showed amoebal rupture at 30°C, indicating active bacterial growth (Fig. 1B). Together, the findings confirmed that the bacteria adapted and grew well at a low temperature in the host amoebae.

### Low-temperature-adapted *Parachlamydia* Bn_9_ can actively grow in immortalized human HEp-2 cells at 30°C, but not at 37°C

**Figure 2:**
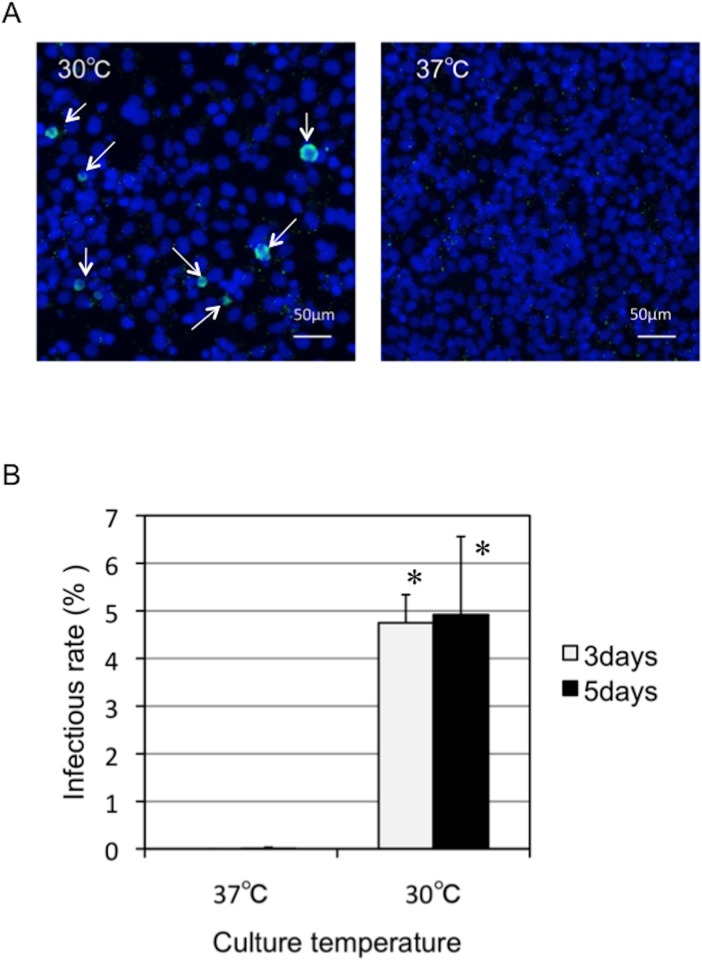
Changes in the infectious rate of *Parachlamydia* Bn_9_ in HEp-2 cells at 30 or 37°C. The HEp-2 cells were infected with the bacteria (MOI 50), and then incubated for 5 days at 30 or 37°C. Inclusion formation was assessed by conventional fluorescence microscopy. (A) Representative inclusion images of HEp-2 cells infected with *Parachlamydia* Bn_9_ at 3 days after infection. Arrows show the inclusion bodies (Green). Blue, DAPI staining. (B) Changes in the inclusion formation rate of *Parachlamydia* Bn_9_ in HEp-2 cells. Data are the means + SD from at least three experiments. *P < 0.05 vs. each culture period (3 or 5 days) at 37°C.

**Figure 3:**
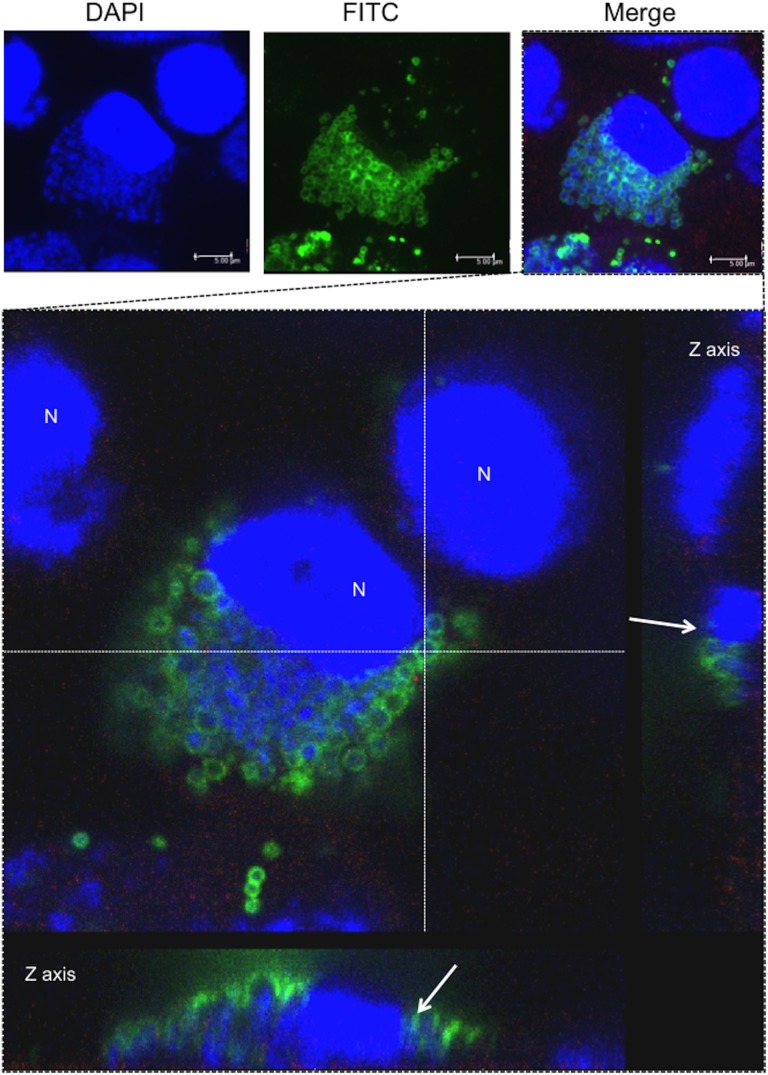
Localization of inclusion bodies formed in the HEp-2 cells infected with *Parachlamydia* Bn_9_ at 30°C. The HEp-2 cells were infected with the bacteria (MOI 10) and then incubated for 5 days at 30°C. The inclusion bodies were assessed 3 days after infection using confocal laser microscopy. The top three images show a representative inclusion body formed in the infected HEp-2 cells. The image with a square surrounded by dotted lines is enlarged below. Arrows in the Z axis panels show bacterial location close to the nucleus of the HEp-2 cells. Blue, DAPI. Green, bacterial cluster. N, HEp-2 nucleus.

We have previously found that *Parachlamydia* Bn_9_ cannot grow in HEp-2 cells at 37°C [13, 14], supporting low-temperature adaptation of the bacteria. However, these findings raise the question of whether the *Parachlamydia* would be able to grow in mammalian cells at a lower temperature, such as 30°C. We therefore assessed whether the bacteria could replicate and grow in HEp-2 cells at 30°C using an inclusion-forming assay and fluorescence microscopy. As expected, specific inclusion bodies formed in HEp-2 cells at 30°C, but not at 37°C (Fig. 2A, arrows), suggesting active growth of the bacteria in the cells depending on culture temperature. We also estimated the bacterial infectious rate in HEp-2 cell cultures based on inclusion formation, although the estimated infectious rates were not high, at approximately 5% (Fig. 2B). We next assessed morphological features and bacterial localization in the HEp-2 cells by confocal microscopy. We observed bacterial cluster inclusions, but not those that are typical of the sequestered inclusion membranes usually seen for pathogenic chlamydiae (Fig. 3). Furthermore, the bacterial clusters appeared to be adjacent to nuclei (Fig. 3, white arrows; See Movie S1), suggesting an intimate interaction between the bacteria and the nucleus of host cells. In contrast, at 37°C, the bacteria failed to replicate inside HEp-2 cells, although the bacteria were translocated close to nucleus (S1 Fig.). As a control experiment, we also confirmed using conventional and confocal fluorescence microscopes that in contrast to 37°C, both the pathogenic chlamydiea (*C. pneumoniae* and *C. trachomatis*) failed to grow into HEp-2 cells at 30°C [S2 Fig. (conventional fluorescence microscope) and S3 Fig. (confocal fluorescence microscope)].

### *Parachlamydia* Bn_9_ grow in immortalized human HEp-2 cells at 30°C with developmental cycle

**Figure 4:**
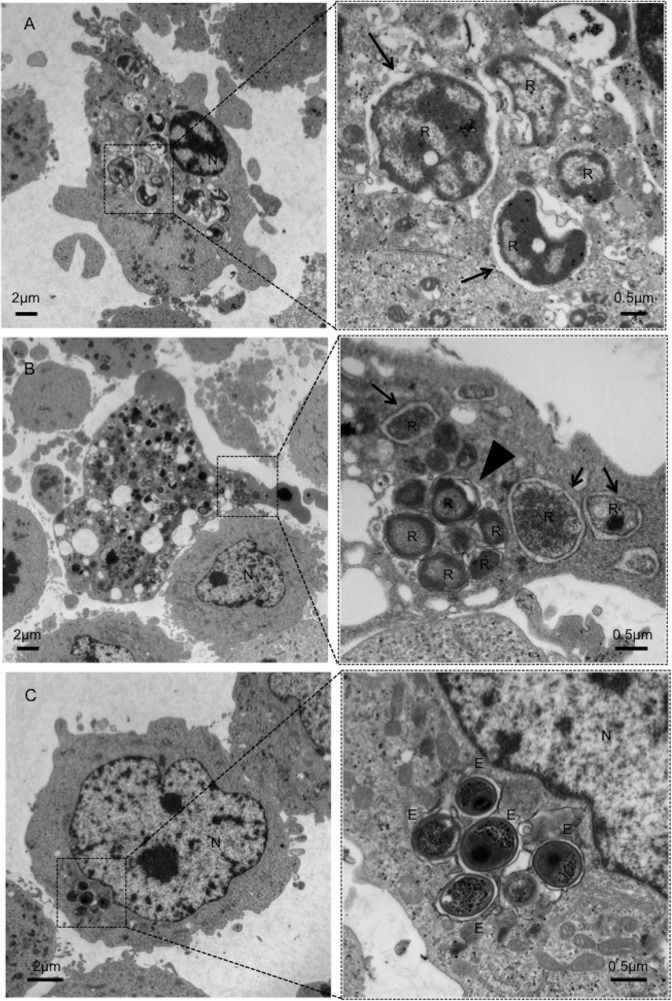
Representative TEM image showing the ultrastructure of *Parachlamydia* Bn_9_ in HEp-2 cells (A-C). The squares surrounded by dotted lines are enlarged right panels. Arrows show the bacterial RBs surrounded by plasma membrane. Arrowhead shows an inclusion body. E, EB. R, RB. N, nucleus.

**Figure 5:**
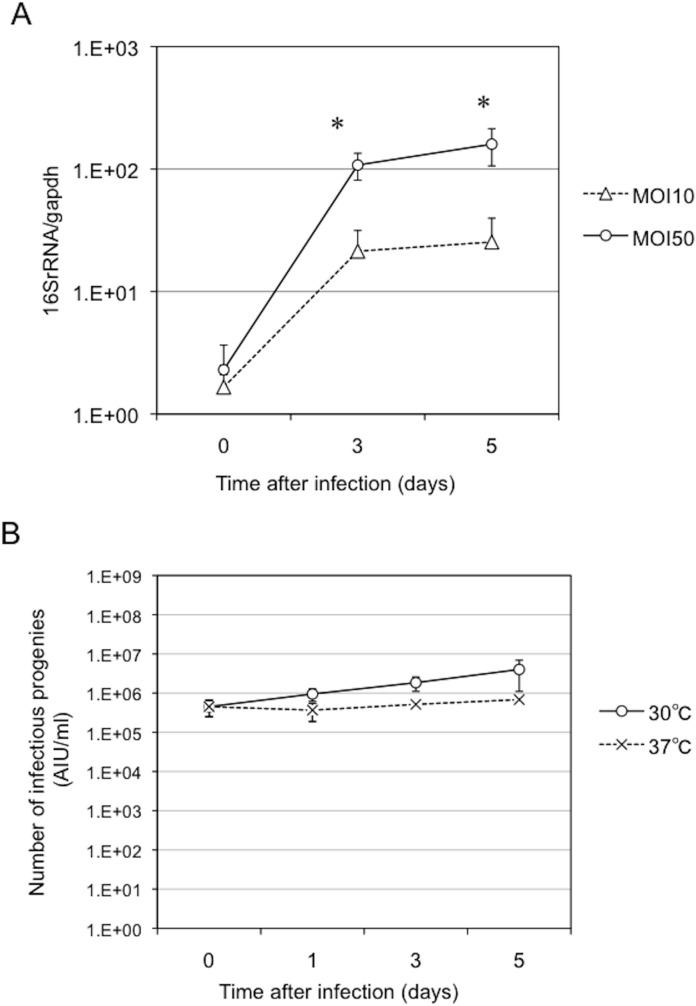
Changes in bacterial growth in the HEp-2 cells infected with *Parachlamydia* Bn_9_ at 30 or 37°C. The HEp-2 cells were infected with the bacteria (MOI 10 or 50), and then incubated for 5 days at 30 or 37°C. Bacterial growth was assessed using qRT-PCR and AIU assays [13]. The cells infected with the bacteria at MOI 50 alone were used for the AIU assay. (A) Change in the level of bacterial *16SrRNA* transcripts in infected HEp-2 cells. Each value shows a ratio of *16SrRNA* transcripts to housekeeping *gapdh* transcripts. Data are the means ± SD from at least three experiments. *P < 0.05 vs. culture with MOI 50. (B) Change in amount of infectious bacterial progeny in infected HEp-2 cells. Each value shows the amount of infection progeny as an AIU value, using a co-culture of the C3 amoebae, as described previously [13]. Data are the means ± SD from at least three experiments.

To further explore the traits of *Parachlamydia* Bn_9_ in HEp-2 cells, bacterial growth was monitored by various methods, including transmission electron microscopy (TEM), qRT-PCR targeting the *16SrRNA* gene, and AIU assays. TEM analysis clearly revealed several differentiated RBs surrounded by plasma membrane (Fig. 4A and B, arrows) and actively replicating RBs into an inclusion body (Fig. 4B, arrowhead). In contrast, the presence of EBs in the infected cells was minimal (Fig. 4C). qRT-PCR showed a significant increase in bacterial *16SrRNA* transcripts depending upon the multiplicity of infection (MOI) (Fig. 5A). Moreover, a slight increase in the numbers of infectious bacterial particles (EBs), estimated by AIU assays, was seen, but without statistical significance (Fig. 5B). In contrast to the active replication of RBs, the maturation to EBs into the HEp-2 cells might be minimal, possibly explaining the slight increase of AIU value. Thus, taken together, we concluded that the bacteria could grow well in HEp-2 cells at 30°C, with developmental cycle, however, minimally possessing secondary infectious ability.

### Intracellular growth mechanism of *Parachlamydia* Bn_9_ in HEp-2 cells at 30°C

**Figure 6:**
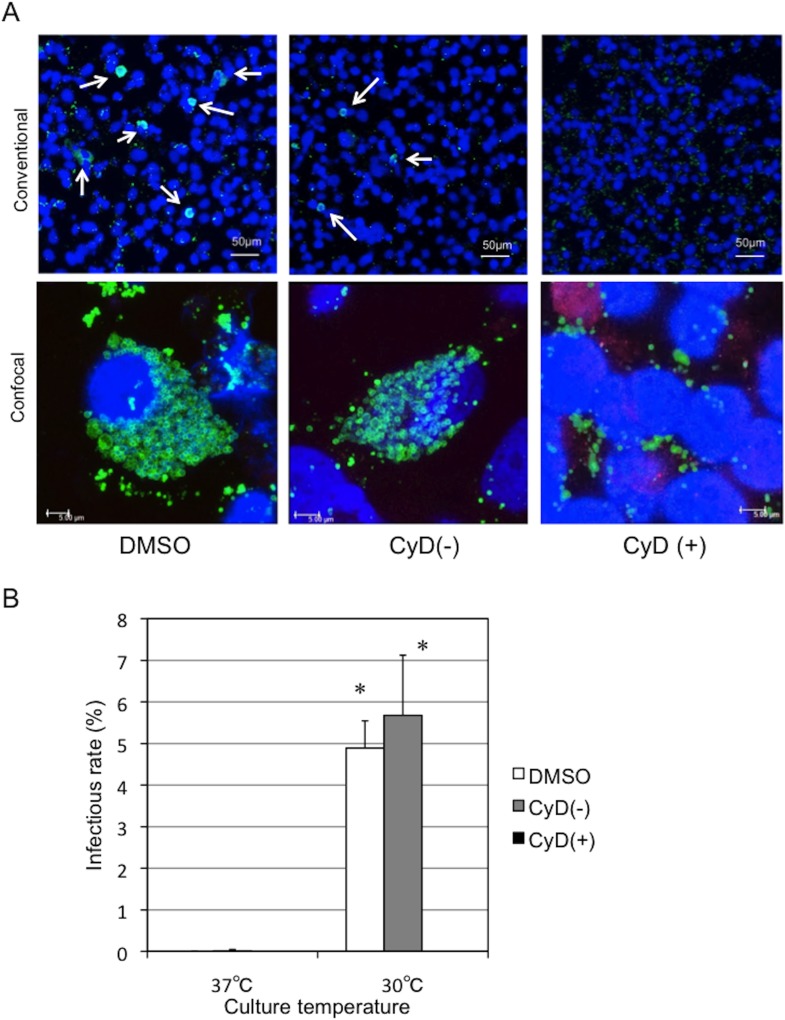
Effect of cytochalasin D on inclusion formation in HEp-2 cells infected with *Parachlamydia* Bn_9_. The HEp-2 cells were infected with the bacteria (MOI 10), in the presence or absence of cytochalasin D (2.5 µg/ml) and then incubated for 5 days at 30°C. Inclusion formation was assessed using conventional and confocal fluorescence microscopy. (A) Representative images showing inclusion formation in infected HEp-2 cells in the presence or absence of cytochalasin D. The images were captured 3 days after infection. Conventional, Observation using conventional fluorescence microscopy. Confocal, Observation using confocal fluorescence microscopy. DMSO, a solvent control. CyD(+), cytochalasin D. CyD(-), medium alone. (B) Change in inclusion formation rate in infected HEp-2 cells in the presence or absence of cytochalasin D. See above. Data are the means + SD from at least three experiments. *P < 0.05 vs. each culture [DMSO, CyD(-), CyD(+)] at 37°C.

**Figure 7:**
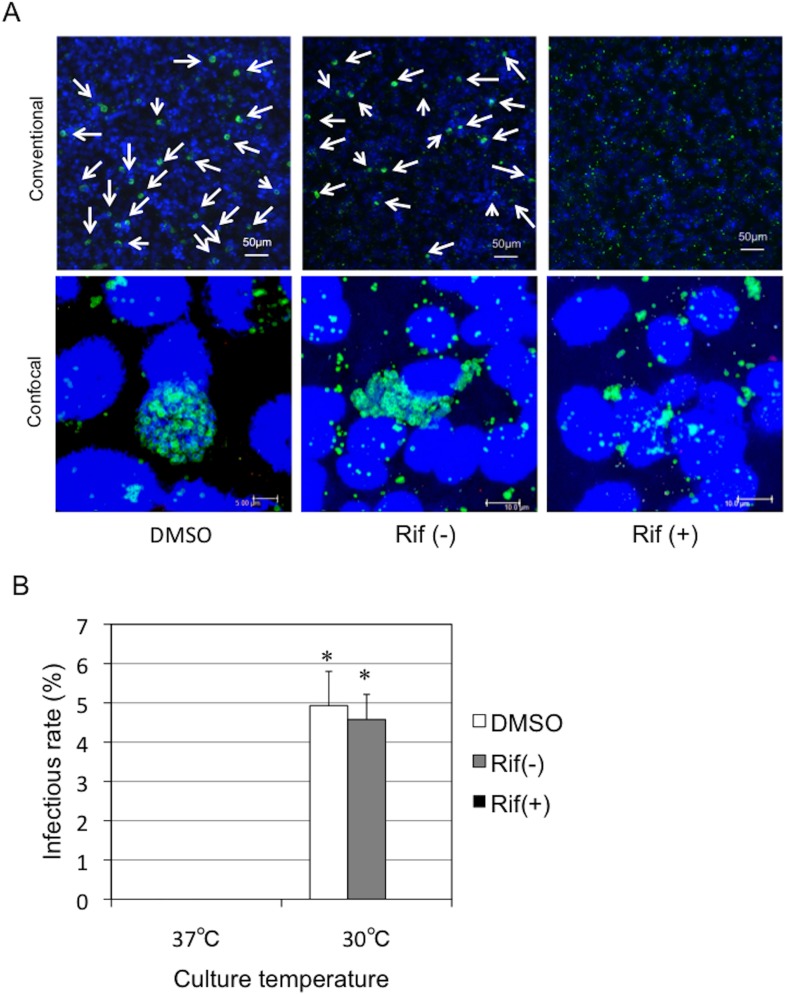
Effect of rifampicin on inclusion formation in HEp-2 cells infected with *Parachlamydia* Bn_9_. The HEp-2 cells were infected with the bacteria (MOI 10), in the presence or absence of rifampicin (0.5 µM) and then incubated for 5 days at 30°C. Inclusion formation was assessed using conventional and confocal fluorescence microscopy. (A) Representative images showing inclusion formation in infected HEp-2 cells in the presence or absence of rifampicin. The images were captured 3 days after infection. Conventional, Observation using conventional fluorescence microscopy. Confocal, Observation using confocal fluorescence microscopy. DMSO, a solvent control. Rif(+), rifampicin. Rif(-), medium alone. (B) Change in inclusion formation rate in infected HEp-2 cells in the presence or absence of rifampicin. See above. Data are the means + SD from at least three experiments. *P < 0.05 vs. each culture [DMSO, Rif(-), Rif(+)] at 37°C.

**Figure 8:**
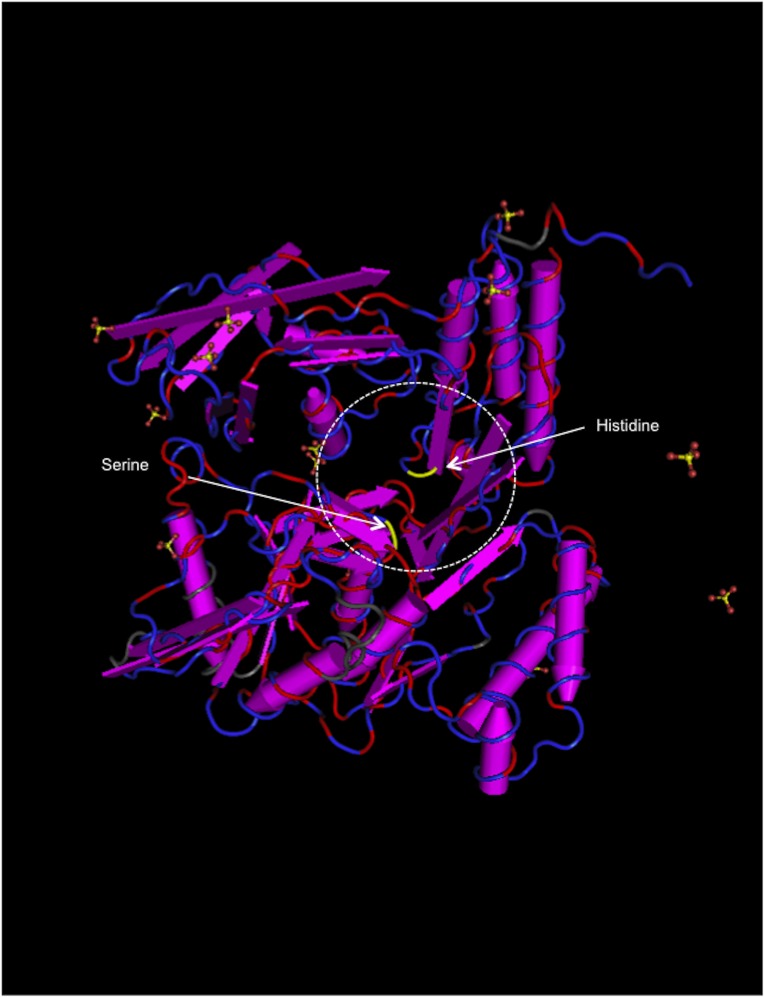
Predicted three-dimensional structure of *Parachalmydia* Bn_9_ CPAF (query: RAST gene ID peg.785). The structure was constructed by alignment with *Chlamydia trachomatis* chain A, crystal structure of mature CPAF (3DOR_A). Both serine and histidine (yellow), which are critical amino acids of the active site of pathogenic chlamydial CPAF (Bednar *et al*., 2011), are well conserved. Red line, identical sequences. Blue line, similar sequences. Gray, non-conserved sequences. See S2 Fig.

**Figure 9:**
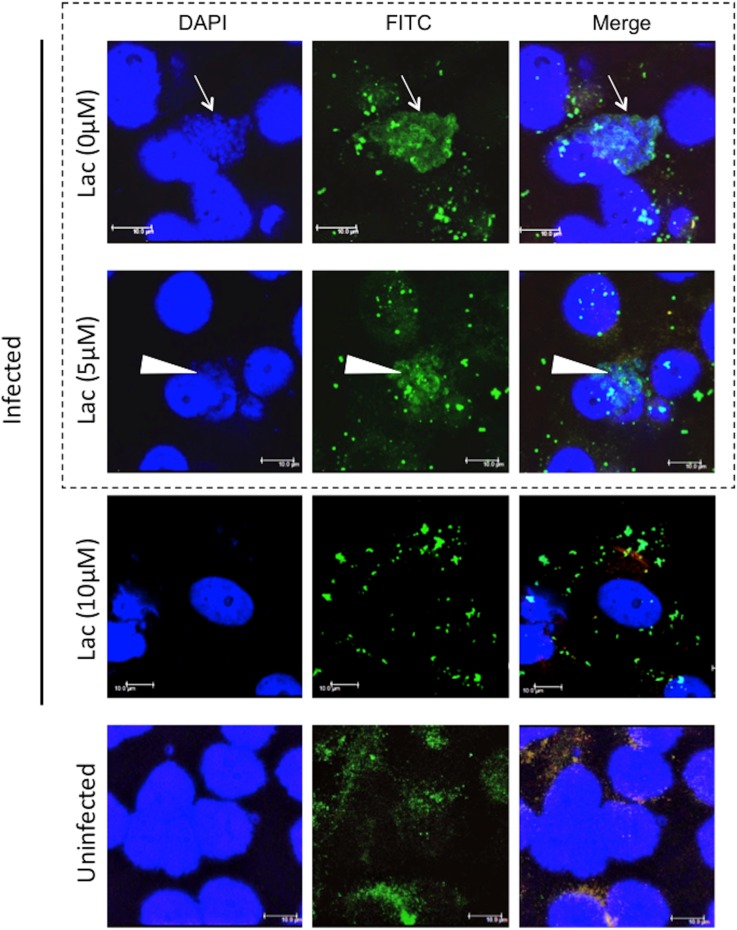
Effect of lactacystin on inclusion formation in HEp-2 cells infected with *Parachlamydia* Bn_9_. The HEp-2 cells were infected with the bacteria (MOI 10), in the presence or absence of lactacystin (5 or 10 µM) and then incubated for 5 days at 30°C. Inclusion formation was assessed using confocal laser microscopy. The images were captured 3 days after infection. Arrows show healthy inclusion formation. Arrowheads show disrupted inclusion in the presence of lactacystin (5 µM). Addition of 10 µM lactacystin completely abolished the formation of typical inclusion bodies in HEp-2 cells. For showing more conclusive image, the images surrounded by dashed line were enlarged into S9 Fig.

We next assessed whether actin polymerization was required for bacterial inclusion formation using cytochalasin D, an inhibitor that blocks actin remodeling [27]. Treatment with cytochalasin D, which is a critical inhibitor of pathogenic chlamydiae [28], inhibited inclusion formation in HEp-2 cells (Fig. 6), indicating that actin polymerization is required for bacterial inclusion formation in HEp-2 cells. We also investigated whether RNA transcription was required for inclusion formation after bacterial entry into the HEp-2 cells using rifampicin, which specifically inhibits bacterial RNA polymerase. As expected, treatment with rifampicin blocked inclusion formation (Fig. 7), indicating a requirement for RNA transcription for bacterial growth inside cells. In chlamydial protease-like activity factor (CPAF) (*C. trachomatis;* DUW_3CX), histidine-101 and serine-499 are two critical residues in the active site [29], and are conserved in *Parachlamydia* Bn_9_ (Fig. 8; S4 Fig.). It is therefore possible that *Parachlamydia* Bn_9_ CPAF is active with possibly an essential role in host cell manipulation. Lactacystin, which directly binds to and blocks pathogenic chlamydial CPAF activity [29], slightly inhibited inclusion formation in infected cells [Fig. 9; the enlarged images surrounded by dashed line are seen as a supplementary data (S5 Fig.)], although no significant change of infectious rates was seen (S6 Fig.).

### A draft genome of *Parachlamydia* Bn_9_ reveals growth potential in human cells

**Figure 10:**
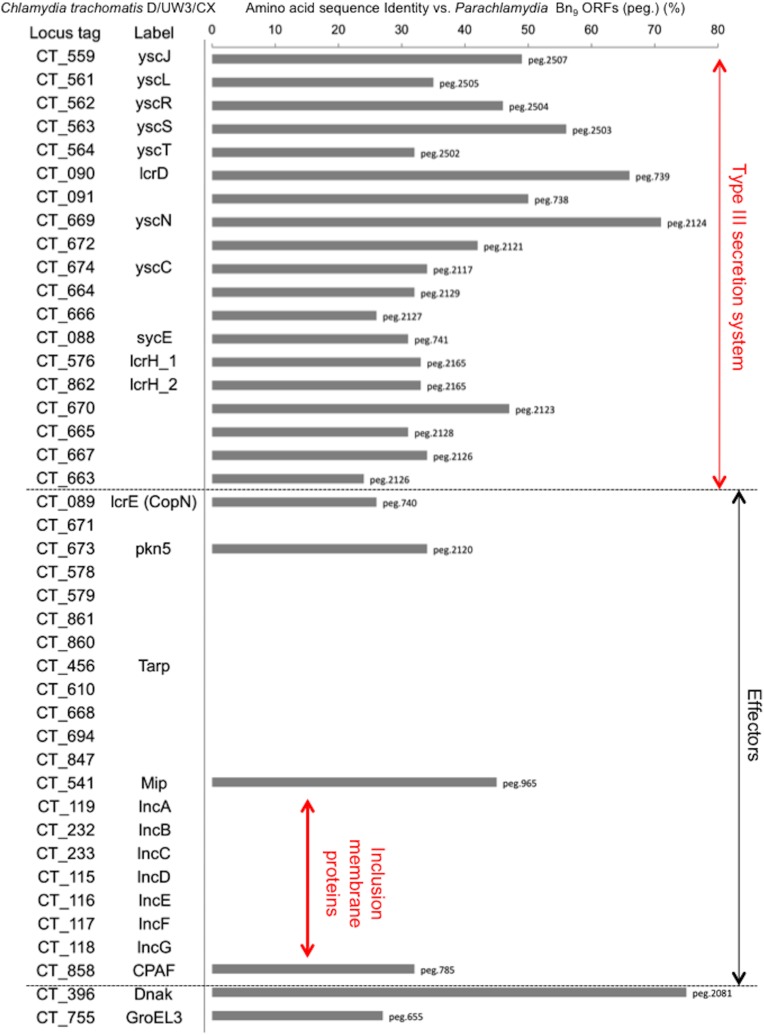
Amino-acid sequence identity of type III secretion system-associated and effector proteins of the *C. trachomatis* D/UW3/CX into *Parachlamydia* Bn_9_. Each of the values (top hit alone) shows percentage identity (%) of *C. trachomatis* D/UW3/CX (NC_000117.1) into *Parachlamydia* Bn_9_ (BAWW01000001-BAWW 01000072). “peg” numbers reveal locus tags for the *Parachlamydia* annotated by using RAST server. See Materials and Methods “Genome sequencing and annotation” and S1 Table. Cut off, E value < 1.0E^-10^.

A draft genome of *Parachlamydia* Bn_9_ was determined by Illumina GAIIx sequencing, assembled using ABySS-pe, and then annotated by the RAST server with manual local BLAST analysis. The draft genome of *Parachlamydia* was 3,000,662 bp (total contig size) in 72 scaffold contigs with a GC content of 39% (DDBJ accession number: BAWW01000001-BAWW01000072). The genome contains 2,748 protein-coding sequences (CDSs), and 46 RNAs (S1 Table). We used KEGG analysis to assess whether the *Parachlamydia* Bn_9_ genome could generate all pathogenic chlamydial metabolic pathways required for human cell adaptation. The draft genome clearly revealed the bacteria to possess metabolic pathways that are almost identical to those of pathogenic chlamydiae, suggesting growth potential in human cells (S7 Fig.). Because of type III secretion system with effector proteins required for host cellular manipulation into pathogenic chlamydiae, we also performed comparative genomic analysis of *Parachlamydia* Bn_9_ with *C. trachomatis* L2 434/Bu. As a result, the genomic analysis revealed that both the bacteria possessed the gene clusters encoding Type III secretion system, although no genes encoding inclusion membrane proteins (IncA-G) responsible for inclusion maturation, which is critically associated with the bacterial survival into the host cells, was found in the *Parachlamydia* Bn_9_ (Fig. 10).

## Discussion

While pathogenic chlamydiae are obligate intracellular pathogens that have successfully adapted to mammalian cells providing a stable-temperature environment, it is likely that ancient pathogenic chlamydiae were endosymbionts of lower invertebrates, living in soil or water environments with a relatively low temperature [1, 2]. Therefore, overcoming the temperature gap was a critical factor for ancient pathogenic chlamydiae to adapt to mammalian cells. Although the mechanism for temperature adaptation remains unknown, demonstrating environmental chlamydia replication in human cells may provide insight. Several environmental chlamydiae strains (e.g. *Parachlamydia* Bn_9_, Hall′s coccus, UV-7; *Protochlamydia* UWE25, R18) are available for such research [2, 30, 31, 32]. *Parachlamydia* Bn_9_, isolated from human nasal mucosa, possesses the highest growth potential in human cells among these strains [33] (See S2 Table). We therefore used strain *Parachlamydia* Bn_9_ to show bacterial growth in human epithelial HEp-2 cells at a low culture temperature of 30°C.

Although our model shows the growth of *Parachlamydia* Bn_9_ in HEp-2 cells at low temperature, the estimated infectious rates were not high, at approximately 5%. The bacterial stock was prepared from amoebal rapture solution and contained amoebal components; therefore, it was possible that these components could inhibit bacterial adhesion to HEp-2 cells. Therefore, to rule out this possibility, the experiment was repeated using bacteria purified by ultra centrifugation to remove amoebal components. However, the infectious rates, improved only slightly (data not shown), indicating the influence of amoebal components was minimal. In addition, we also confirmed that heat-treated bacteria formed no inclusion bodies in HEp-2 cells (data not shown). Thus, taken together, we concluded that the bacteria could replicate and actively grow in HEp-2 cells at the low culture temperature of 30°C, (and that artificial bacterial auto-aggregation did not occur), although the reason for low infectious rates remains unclear.

The data obtained from inclusion formation, TEM, AIU and qRT-PCR analyses revealed that the bacteria could grow well in HEp-2 cells at 30°C, with developmental cycle, however, minimally possessing re-differentiation from RB to EB with secondary infectious ability. While the exact reason for limited re-differentiation remains unknown, we noticed that unreplicated RBs surrounded by plasma membrane were seen in infected HEp-2 cells. Thus, because of presumably the RB surrounding membrane insufficiently decorated by effectors secreted from the bacteria, the RBs may be easily attacked and lose the ability to differentiate; in fact, effector homologues responsible for the cellular manipulation of pathogenic chlamydiae were minimally found into the draft genome of the *Prachlamydia* (See Fig. 10). Such limited re-differentiation into inclusion membrane are likely to be traits of ancient pathogenic chlamydiae at an early stage of adaption to mammalian cells. In addition, although the viability of infected HEp-2 cells was barely maintained over 5 days at a low culture temperature of 30°C, whether the low-culture temperature interfered with normal cellular homeostasis remains to be clear.

Cytochalasin D, which is a critical inhibitor of pathogenic chlamydial invasion and expansion for inclusion [28], inhibited inclusion formation in HEp-2 cells, indicating that *Parachlamydia* entry to cells is required to induce actin polymerization in HEp-2 cells. In contrast, the draft genome of *Parachlamydia* revealed an absence of translocated actin-recruiting phosphoprotein (TARP), similar to the other environmental chlamydiae [22]. TARP plays an essential role in pathogenic chlamydia entry via enhancement of actin polymerization. Although the exact mechanism by which bacteria internalize into cells remains unknown, bacterial entry may not require TARP, indicating a role for other unknown bacterial effectors. We also found that treatment with rifampicin also blocked inclusion formation, indicating a requirement for RNA transcription for bacterial growth inside cells. Thus, taken together, we concluded that *Parachlamydia* possesses the potential for active bacterial replication in HEp-2 cells at the low culture temperature of 30°C, with developmental cycle.

CPAF is widely conserved among chlamydiae, including *Parachlamydia* Bn_9_[15, 22], and it is intriguing that pathogenic chlamydial CPAF directly contributes to the prevention of apoptosis of infected cells through degradation of BH3-only proteins to maintain infected host cells [24, 25]. CPAF is also required to provide inclusion membrane with flexibility to facilitate chlamydial maturation into inclusion bodies [26]. Meanwhile, histidine-101 and serine-499 in CPAF (*C. trachomatis;* DUW_3CX), which are two critical residues in the active site [29], are conserved in *Parachlamydia* Bn_9_, suggesting that *Parachlamydia* CPAF is active and also has a critical role in mammalian cells. It is therefore expected that a CPAF inhibitor, lactacystin, which directly binds to critical CPAF residues and blocks pathogenic chlamydial CPAF activity [29], would work similarly to inhibit *Parachlamydia* Bn_9_ CPAF activity, possibly preventing inclusion formation. Lactacystin slightly modified inclusion formation in cells, suggesting that CPAF may be involved in bacterial growth in HEp-2 cells at low temperature. Furthermore, no significant change of infectious rate was seen, partly because of difficulty in separating abnormal bacterial clusters (faintish inclusion-like bodies) from normal inclusion bodies by conventional microscopy. Although the exact reason why the number of abnormal bacterial clusters increased remains unknown, we are speculating that even though in the presence of lactacystin the bacteria could replicate slowly with the bacterial disruption, resulting in faintish inclusion-like bodies.

The draft genome of *Parachlamydia* Bn_9_ revealed the possession of metabolic pathways almost identical to those of pathogenic chlamydiae, suggesting a growth potential in human cells. Meanwhile, the bacteria entered HEp-2 cells and were close to the nucleus without inclusion maturation, but the bacteria failed to grow in HEp-2 cells at 37°C. Interestingly, the comparative genomic analysis of *Parachlamydia* Bn_9_ with *C. trachomatis* L2 434/Bu revealed that while both the bacteria possessed the gene clusters encoding Type III secretion system, no gene encoding inclusion membrane proteins (A-G) responsible for inclusion maturation associating with vacuole fusion was seen in the *P. acanthamoebae* Bn_9_, as well as previous study [22]. The finding appears to show a possible explanation why *Parachlamydia* Bn_9_ lacked inclusion maturation. It is also possible that this growth failure is due to an increase in the expression of unknown harmful effectors secreted by the bacteria into HEp-2 cells. Interestingly, we have previously reported that the CPAF of another environmental chlamydia, *Protochlamydia* R18, which was isolated from river, is involved in the induction of apoptosis due to mitochondria dysfunction in bacterial-stimulated HEp-2 cells at 37°C [15], suggesting a substrate change depending on the temperature environment. This may change the target substrate at 37°C. Even if this is true, how the substrate change occurs remains unknown.

## Conclusion

This is the first description of the environmental chlamydia, *Parachlamydia* Bn_9_, partially growing in human immortal epithelial HEp-2 cells at a low-temperature condition of 30°C. This indicates that the bacteria possess potential for adaptation in human cells and overcoming a temperature gap may have been a critical event for the adaptation of ancient chlamydiae in mammalian cells. This is a suitable *in vitro* model system to explore the process of ancient chlamydial evolution to overcome a temperature gap, providing understanding for host-parasite interaction in bacterial pathogenesis.

## Materials and Methods

### Amoebae and human cell lines

*Acanthamoeba castellanii* C3 (ATCC 50739) (C3 amoebae) was purchased from the American Type Culture Collection (ATCC). The amoebae were maintained in peptone-yeast-glucose (PYG) liquid medium [0.75% (w/v) peptone, 0.75% (w/v) yeast extract, and 1.5% (w/v) glucose] at 30°C under humid conditions. Immortalized human epithelial HEp-2 cells (RCB1889) purchased from the Reken Cell Bank (Ibaraki, Japan) were also cultured at 37°C in 5% CO_2_ in Dulbecco′s Modified Eagle′s Medium (DMEM) (Sigma, St. Louis, MO, USA), containing 10% heat-inactivated fetal calf serum (FCS) with antibiotics (100U/ml penicillin and 100 µg/ml streptomycin) (Sigma).

### Bacteria and assessment of bacterial growth

*Parachlamydia* Bn_9_ (ATCC VR-1476) was purchased from ATCC [33]. The bacteria were propagated in the C3 amoeba cell culture system according to methods described previously [13]. In brief, infected amoebae were harvested and disrupted by freeze-thawing. Following centrifugation at 180 × *g* for 5 min to remove cell debris, the bacteria were concentrated by high-speed centrifugation at 8,000 × *g* for 10 min. The bacterial pellet was resuspended in sucrose-phosphate-glutamic acid buffer (0.2 M sucrose, 3.8 mM KH_2_PO_4_, 6.7 mM Na_2_HPO_4_, and 5 mM L-glutamic acid; pH 7.4), and then stored at −80°C until use. The number of infectious progeny was determined with an amoeba-infectious unit (AIU) assay, using a co-culture of the C3 amoebae, as described previously [13]. Pathogenic chlamydiae (*C. pneumoniae* TW183 and *C. trachomatis* L2434/Bu) were also used for a control experiment to confirm growth failure at low temperature. Both the pathogenic bacteria were propagated in a HEp-2 cell culture system as described previously [34].

### AIU assay

AIU assay was performed according to the method established previously [13]. Each of the cell lysates including the bacteria was diluted from 10^0^–10^-7^ with PYG broth and incubated with C3 amoebae (10^4^–10^5^ per well) in PYG broth containing cycloheximide (200μg/ml). The infectious rate of the bacteria to the amoebae in each of the wells was determined by microscopic observation at a magnification of ×100, following DAPI staining; ten fields were randomly selected for this assessment. The estimated infectious rates were plotted as a logistic sigmoid dilution curve, and the 50%-infectious dose was corresponded to the bacterial numbers [See reference 13].

### Bacterial genomic DNA extraction for genome sequencing

*Parachlamydia*-infected amoebae were collected by centrifugation at 1,500 × *g* for 30 min. The resulting pellets were suspended in PYG medium. Amoebae were disrupted by bead-beating for 5 min according to a previously described method [35], and then centrifuged at 150 × *g* for 5 min to remove cell debris. The supernatant including intact bacteria was treated with DNase (Sigma) for 30 min at room temperature. After washing, the bacteria were resuspended in 10 mM HEPES buffer containing 145 mM NaCl. The suspension was carefully overlaid onto 30% Percoll, and then the bacteria were collected from the lower layer following centrifugation at 30,000 × *g* for 30 min. Bacterial genomic DNA was extracted from bacterial pellets with a phenol-chloroform method.

### Infection

*Parachlamydia* Bn_9_ (2.5 × 10^6^ AIU/well) were added to each well of a 24-well plate seeded with C3 amoebae (2.5 × 10^5^ cells/well) at a multiplicity of infection (MOI) of 10 suspended in PYG broth and incubated for 1 h. After washing, the cultures were resuspended in fresh medium and incubated for up to 5 days at two distinct temperatures (30 or 37°C) in a normal atmosphere. During the culture period, amoebal cells were regularly collected to assess bacterial growth using the AIU assay and DAPI staining. Similarly, HEp-2 cells (2 × 10^5^ cells/well) were infected with bacteria(2 × 10^6^ or 1 × 10^7^ AIU/well) at a MOI of 10 or 50. The infected HEp-2 cells were harvested by centrifugation (800 × *g*) for 1 h. After washing with DMEM to remove noninfectious bacteria, the cells were incubated in DMEM containing 10% FCS with antibiotics for up to 5 days at two distinct temperatures (30 or 37°C) in 5–8% CO_2_; the low temperature condition (30°C) was set up using AnaeroPack (microaerphile) (Mitsubishi Gas Chemical Company, Tokyo, Japan). In some experiments, cytochalasin D (final concentration 2.5 μg/ml), rifampicin (final concentration 0.5 μM), and lactacystin (final concentration 5 or 10 μM) were added to the culture. The cells were collected immediately after infection and then daily throughout the culture period for determination of inclusion formation by conventional immunofluorescence (or confocal) microscopy, and evaluation of bacterial numbers via AIU assays, bacterial morphology with TEM, and *16S rRNA* transcript levels using qRT-PCR. Also, total genomic DNA was extracted from some cultures as described below (See *Genome sequencing and annotation*) and was used for *Parachlamydia* Bn_9_ whole genome sequencing. In addition, the number of inclusion bodies formed into HEp-2 cells per total culture cells was estimated as bacterial infectious rate (%). Specifically, the number of inclusion bodies was estimated by the observation onto three to five randomly selected fields with more than 100 cells under a fluorescence microscope.

### Immunocytochemistry

Immortalized cells adhering to cover slips in 24-well plates were washed with cold PBS and then fixed in 4% (w/v) paraformaldehyde. Fixed cells were incubated with rabbit serum containing anti-*Parachlamydia* polyclonal antibodies for 1 h at room temperature, and then reacted with FITC-labeled anti-rabbit IgG secondary antibody (Sigma). After staining, images were captured using either a conventional fluorescence microscope or a confocal laser fluorescence microscope. Rabbit serum containing anti-*Parachlamydia* antibodies against formalin fixed EBs was produced by Iwaki (Tokyo, Japan).

### TEM

*Parachlamydia*-infected HEp-2 cells were immersed in a fixative containing 3% glutaraldehyde in 0.1 M phosphate buffered saline (PBS), pH 7.4, for 24 h at 4°C. Following a brief wash with PBS, cells were processed by alcohol dehydration and embedding in Epon 813. Ultrathin cell sections were stained with lead citrate and uranium acetate prior to visualization by electron microscopy (on a Hitachi H7100; Hitachi, Tokyo, Japan) as described previously [13].

### qRT-PCR

Total RNA was extracted from *Parachlamydia*-infected cells using an RNeasy Mini Kit (Qiagen) according to the manufacturer′s protocol. Extracted RNA was treated with DNase I (DNA-free; Ambion) to eliminate contaminating DNA. The resulting RNA preparations were confirmed to be DNA-free when a negative result was produced using PCR without the reverse transcription step. Reverse transcription of 2 µg of total RNA by avian myeloblastosis virus reverse transcriptase was performed with random primers in a commercial reaction mixture (Reverse Transcription System; Promega, Madison, WI, USA). Template genomic DNA and resulting cDNA samples were then subjected to PCR with pairs of primers specific for *Chlamydiales 16SrRNA* [36] and host cellular *gapdh* (sense, 5′-AACGGGAAGCTCACTGGCATG-3′; antisense, 5′-TCCACCAACCTGTTGCTGTAG-3′) [34]. The thermal cycling conditions were 95°C for 10 min, followed by 40 cycles of 95°C for 15 s, 60°C for 1 min, and 72°C for 20 s. Standard curves for chlamydial *16SrRNA* cDNA and *gapdh* cDNA were constructed using a series of diluted *Parachlamydia* and HEp-2 RNA samples extracted from normally cultured HEp-2 cells. The data are expressed as a ratio of the *16S rRNA* transcripts: *gapdh* transcripts (for *Parachlamydia*-infected cells).

### Genome sequencing and annotation

*Parachlamydia* Bn_9_ DNA libraries were prepared using a TruSeq DNA Sample Kit (Illumina, San Diego, CA). Sequencing runs for paired-end sequences were performed using an Illumina Genome Analyzer (Illumina Hiseq, Illumina). The sequencing runs and read assembly of the libraries were carried out by Hokkaido System Science (Sapporo, Japan). Annotation of genes from the draft genome sequence was performed using Rapid Annotation using Subsystem Technology (RAST: http://rast.nmpdr.org/) [37] with a local manual BLASTp search.

### Prediction of 3D structure for annotated genes

The three-dimensional structure of the CPAF annotated protein sequence was predicted using a web program, protein BLAST, in the Molecular Modeling Database (MMDB) (http://www.ncbi.nlm.nih.gov/Structure/MMDB/mmdb.shtml) [35]. Cn3D 4.3 (http://www.ncbi.nlm.nih.gov/Structure/CN3D/cn3dmac.shtml) was used to display the predicted structure [38].

### Statistical analysis

Data were compared using Student′s *t*-test. A *P*-value of less than 0.05 was considered significant.

### Contig sequence accession numbers

The draft genome sequence for the *Parachlamydia* Bn_9_ strain has been deposited in the DNA Data Bank of Japan [DDBJ accession numbers: BAWW01000001-BAWW01000072].

## Supporting Information

S1 Fig Localization of *Parachlamydia* Bn_9_ in HEp-2 cells at 37°C. The HEp-2 cells were infected with the bacteria (MOI 10), and then incubated for 5 days at 37°C. Inclusion formation was assessed at 3 days after infection using confocal laser microscopy. The top three images show no inclusion bodies formed in infected HEp-2 cells. The image surrounded by dotted lines is enlarged below. Arrows in the Z-axis panel show that the bacteria located close to the nucleus of HEp-2 cells do not form bacterial clusters. Blue, DAPI. Green, bacteria. N, HEp-2 nucleus.
(PDF)

S2 Fig Pathogenic chlamydiea failed to form inclusion bodies into HEp-2 cells at 30°C (conventional fluorescence microscopic analysis). The HEp-2 cells were infected with each of the bacteria (MOI 10), and then incubated for 3 days at either 30°C or 37°C. Inclusion formation was assessed at 3 days after infection using conventional fluorescence microscope. PaBn_9_, *Parachlamydia* Bn_9_. CtL2_434/Bu, *C. trachomatis* L2 434/Bu. CpTW183, *C. pneumoniae* TW183. Magnification, ×200.
(PDF)

S3 Fig Pathogenic chlamydiea failed to grow into HEp-2 cells at 30°C (confocal fluorescence microscope). See the legend for S2 Fig. Inclusion formation was assessed at 3 days after infection using confocal fluorescence microscope. PaBn_9_, *Parachlamydia* Bn_9_. CtL2_434/Bu, *C. trachomatis* L2 434/Bu. CpTW183, *C. pneumoniae* TW183.
(PDF)

S4 Fig Structure based sequence alignment between *Parachlamydia* Bn_9_ CPAF (query: RAST gene ID peg. 785) and *C. trachomatis* CPAF (3DOR_A). (A) Putative conserved domain on the query sequence. (B) Sequence alignment. Arrows show serine and histidine, which are critical amino acids of the active site of pathogenic chlamydial CPAF [29], are well conserved. Red line, identical sequences. Blue line, similar sequences. Gray, non-conserved sequences.
(PDF)

S5 Fig Effect of lactacystin on inclusion formation in HEp-2 cells infected with *Parachlamydia* Bn_9_. For showing more conclusive effect of lactacystin, the images [Lac (0µM) and Lac (5 µM)] surrounded by dashed line into Fig. 9 were enlarged as a supplementary data into this figure.
(PDF)

S6 Fig Effect of lactacystin on inclusion formation in HEp-2 cells infected with *Parachlamydia* Bn_9_. The HEp-2 cells were infected with the bacteria (MOI 10), in the presence or absence of lactacystin (5 or 10 µM) and then incubated for 5 days at 30 or 37°C. Inclusion formation was assessed using conventional fluorescence microscopy. (A) Representative images showing inclusion formation in infected HEp-2 cells in the presence or absence of lactacystin. The images were captured 3 days after infection. Lac, lactacystin. (B) Change in infectious rate in infected HEp-2 cells in the presence or absence of lactacystin. See above. Data are the means + SD from at least three independent experiments performed in triplicate. *P < 0.05 vs. each culture [Lac(-), Lac5µM, Lac10µM] at 37°C. NS, no statistical significance.
(PDF)

S7 Fig Comparison of *Parachlamydia* Bn_9_ metabolic pathways with those of *C. trachomatis* L2 434 Bu (NC010287.1). Blue lines, unique to *Parachlamydia* active modules. Red lines, shared modules. Green lines; modules specific for *C. trachomatis*.
(PDF)

S1 Movie Three-dimensional structure of a *Parachalmydia* Bn_9_ cluster formed in an infected HEp-2 cell. The HEp-2 cells were infected with the bacteria (MOI 10), and then incubated for 5 days at 30°C. Blue, DAPI. Green, bacteria.
(MOV)

S1 Table *Parachlamydia acanthamoebae* Bn_9_ gene IDs with features. (PDF)

S2 Table Properties with sources of *Parachlamydia* and *Protochlamydia* strains used for comparative genomic analysis. (PDF)
