# Supplementary figures and images for: Amoebal Endosymbiont Parachlamydia acanthamoebae Bn9 Can Grow in Immortal Human Epithelial HEp-2 Cells at Low Temperature; An In Vitro Model System to Study Chlamydial Evolution
Source: PLoS One. 2015 Feb 2;10(2):e0116486. doi: 10.1371/journal.pone.0116486 (PMC4314085; doi:10.1371/journal.pone.0116486)

Supplementary figure 1

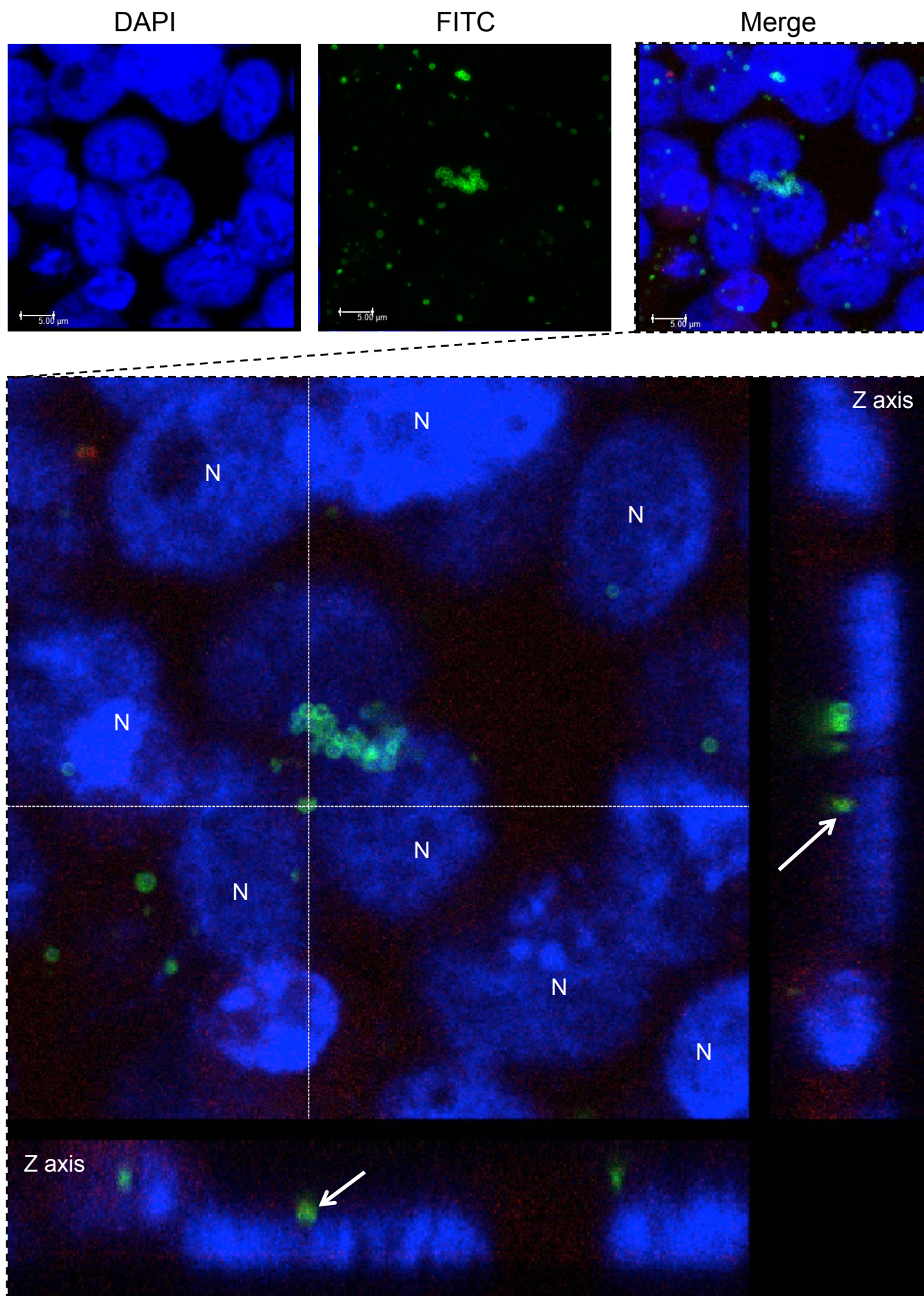

Supplement: S1 Fig — The HEp-2 cells were infected with the bacteria (MOI 10), and then incubated for 5 days at 37°C. Inclusion formation was assessed at 3 days after infection using confocal laser microscopy. The top three images show no inclusion bodies formed in infected HEp-2 cells. The image surrounded by dotted lines is enlarged below. Arrows in the Z-axis panel show that the bacteria located close to the nucleus of HEp-2 cells do not form bacterial clusters. Blue, DAPI. Green, bacteria. N, HEp-2 nucleus. (PDF) [file pone.0116486.s001.pdf]

Supplementary figure 2

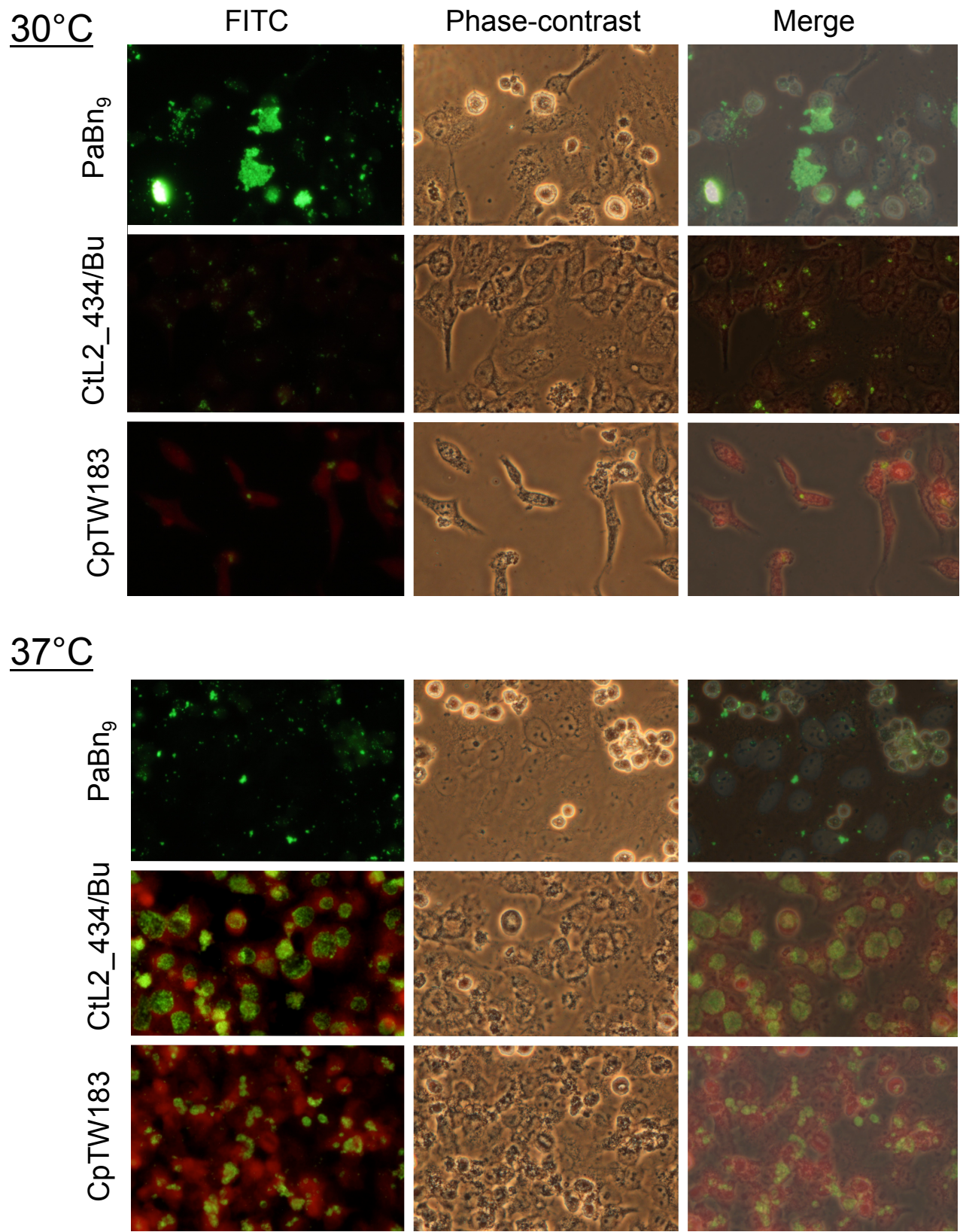

Supplement: S2 Fig — The HEp-2 cells were infected with each of the bacteria (MOI 10), and then incubated for 3 days at either 30°C or 37°C. Inclusion formation was assessed at 3 days after infection using conventional fluorescence microscope. PaBn9, Parachlamydia Bn9. CtL2_434/Bu, C. trachomatis L2 434/Bu. CpTW183, C. pneumoniae TW183. Magnification, ×200. (PDF) [file pone.0116486.s002.pdf]

Supplementary figure 3

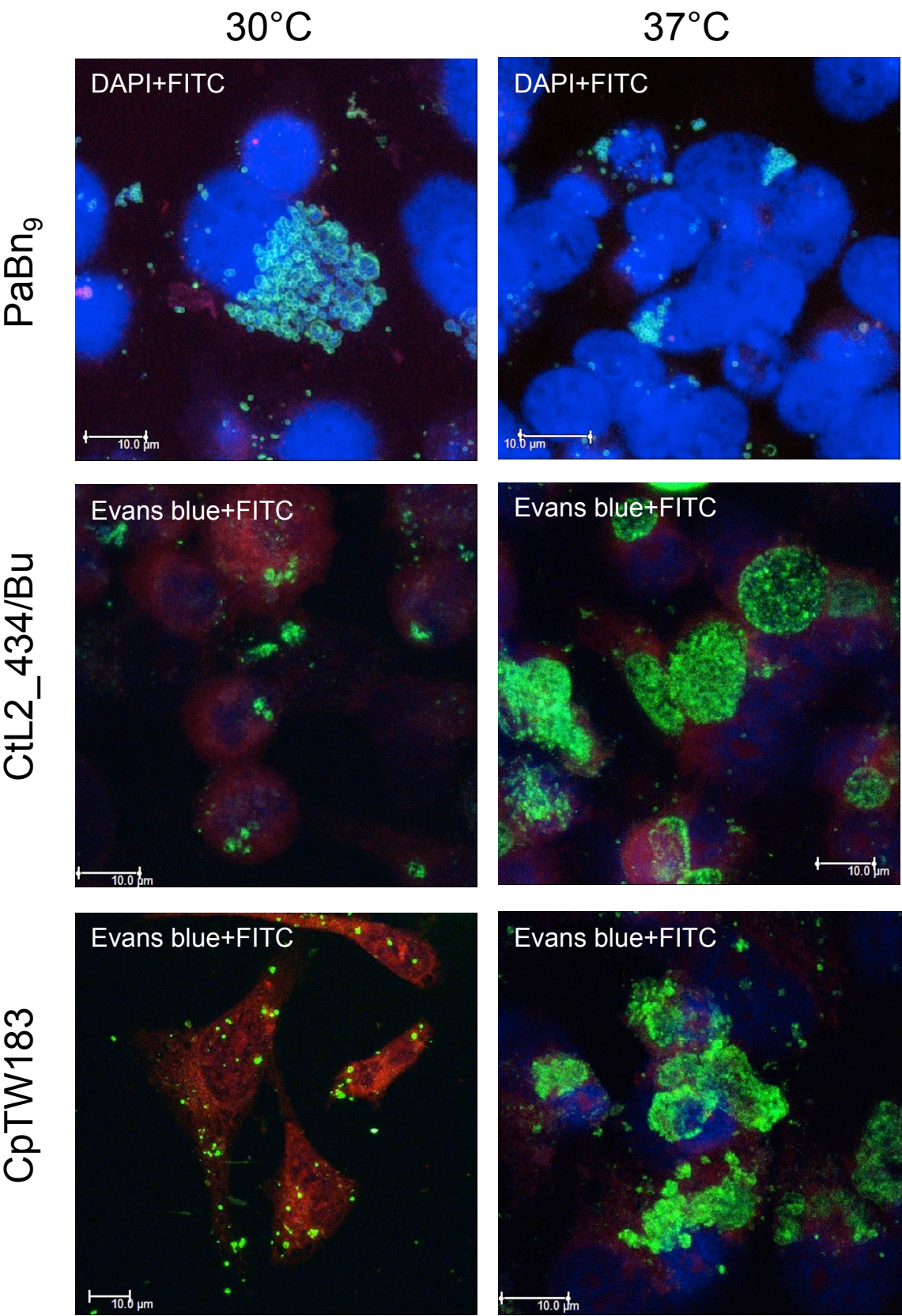

Supplement: S3 Fig — See the legend for S2 Fig. Inclusion formation was assessed at 3 days after infection using confocal fluorescence microscope. PaBn9, Parachlamydia Bn9. CtL2_434/Bu, C. trachomatis L2 434/Bu. CpTW183, C. pneumoniae TW183. (PDF) [file pone.0116486.s003.pdf]

Supplementary figure S5 (The images surrounded by dashed line into Figure 9 are enlarged)

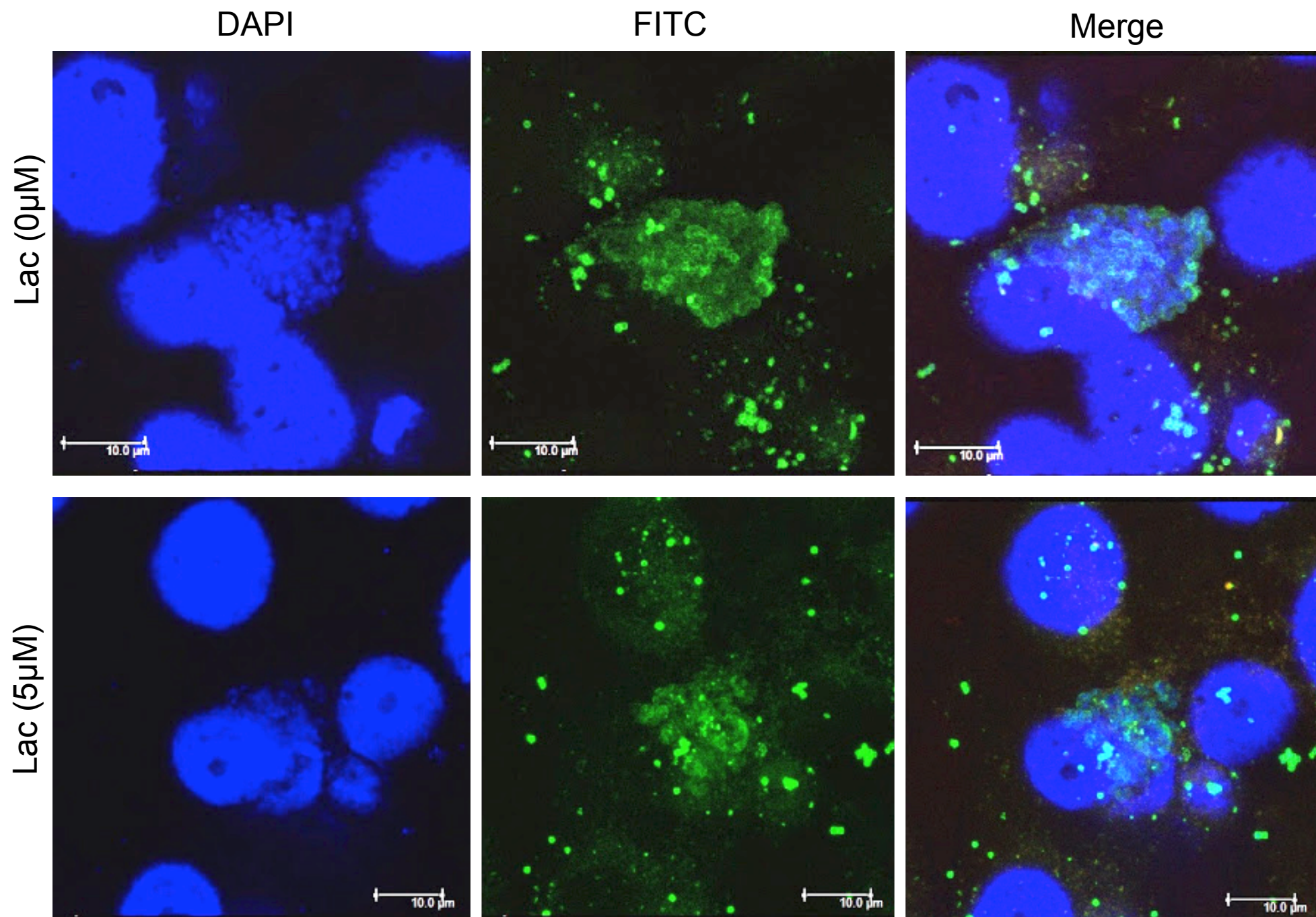

Supplement: S5 Fig — For showing more conclusive effect of lactacystin, the images [Lac (0µM) and Lac (5 µM)] surrounded by dashed line into Fig. 9 were enlarged as a supplementary data into this figure. (PDF) [file pone.0116486.s005.pdf]

Supplementary figure 6

A

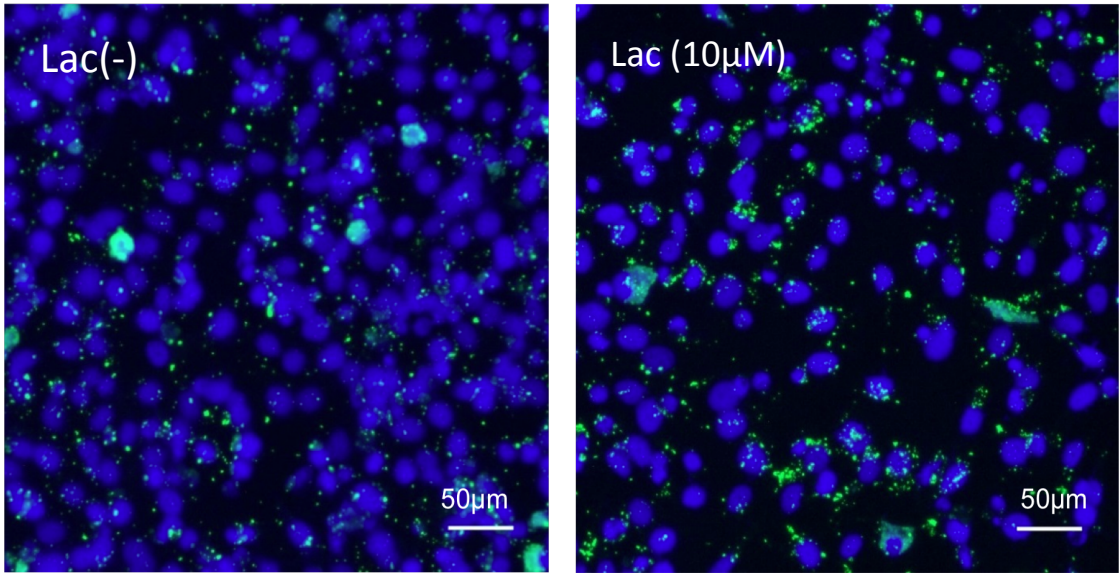

B

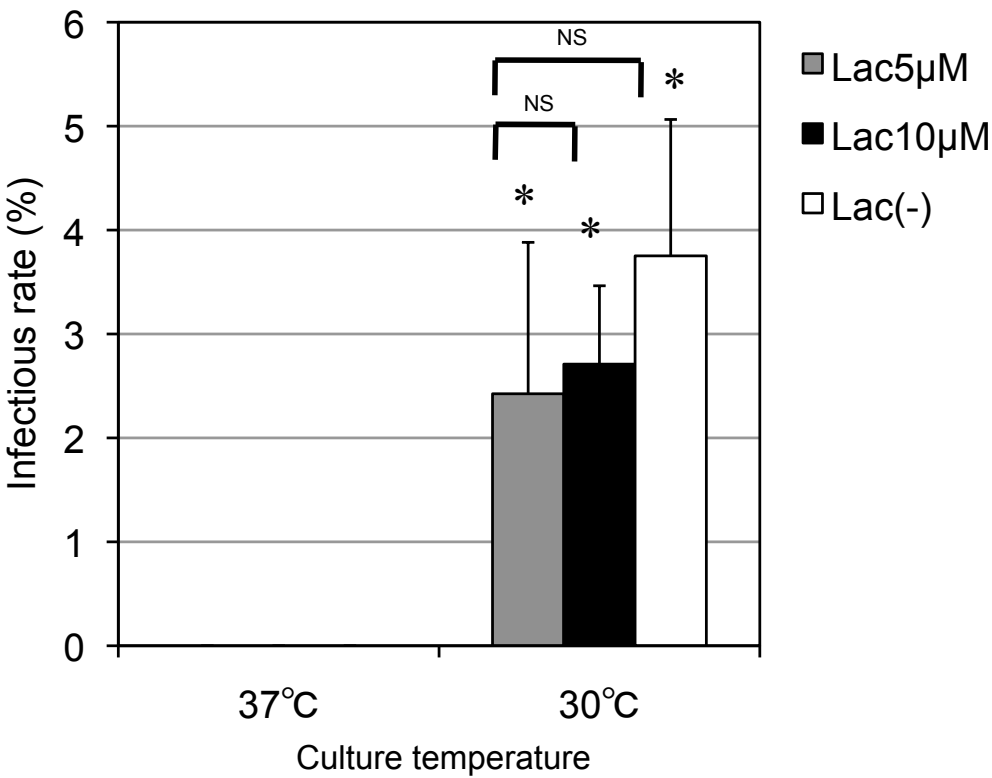

Supplement: S6 Fig — The HEp-2 cells were infected with the bacteria (MOI 10), in the presence or absence of lactacystin (5 or 10 µM) and then incubated for 5 days at 30 or 37°C. Inclusion formation was assessed using conventional fluorescence microscopy. (A) Representative images showing inclusion formation in infected HEp-2 cells in the presence or absence of lactacystin. The images were captured 3 days after infection. Lac, lactacystin. (B) Change in infectious rate in infected HEp-2 cells in the presence or absence of lactacystin. See above. Data are the means + SD from at least three independent experiments performed in triplicate. *P < 0.05 vs. each culture [Lac(-), Lac5µM, Lac10µM] at 37°C. NS, no statistical significance. (PDF) [file pone.0116486.s006.pdf]

Supplementary figure S7

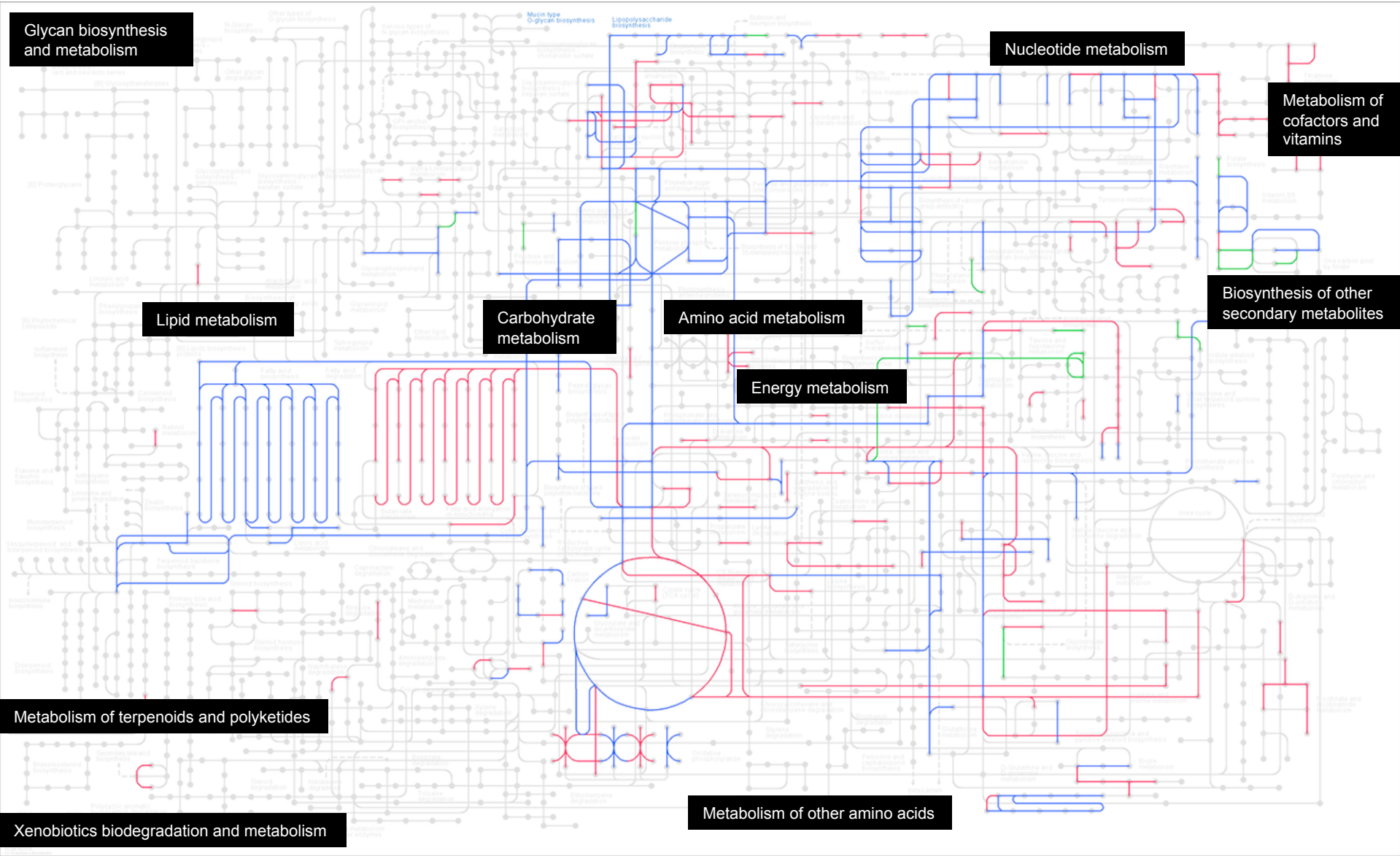

Supplement: S7 Fig — Blue lines, unique to Parachlamydia active modules. Red lines, shared modules. Green lines; modules specific for C. trachomatis. (PDF) [file pone.0116486.s007.pdf]
