# Supplementary material for: Amoebal Endosymbiont Parachlamydia acanthamoebae Bn9 Can Grow in Immortal Human Epithelial HEp-2 Cells at Low Temperature; An In Vitro Model System to Study Chlamydial Evolution
Source: PLoS One. 2015 Feb 2;10(2):e0116486. doi: 10.1371/journal.pone.0116486 (PMC4314085; doi:10.1371/journal.pone.0116486)
Supplement: S4 Fig — (A) Putative conserved domain on the query sequence. (B) Sequence alignment. Arrows show serine and histidine, which are critical amino acids of the active site of pathogenic chlamydial CPAF [29], are well conserved. Red line, identical sequences. Blue line, similar sequences. Gray, non-conserved sequences. (PDF) [file pone.0116486.s004.pdf]

Supplementary figure 4

A

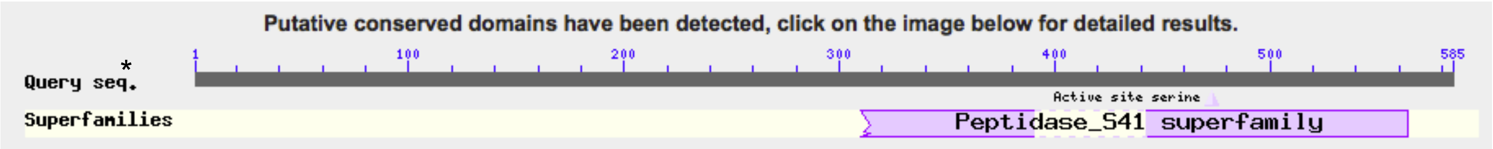

*Query: *Parachlamydia acanthamoebae* Bn9 peg.785

B

E-value: **1.45e-92**, bit-score: **299**, aligned-length: **538**, Identity to query: **35%**

```
                 10        20        30        40        50        60        70        80
         ....*....|....*....|....*....|....*....|....*....|....*....|....*....|....*....|
273329* 26 SLLRQEMVEDLHFIRKNFQIKYVPADWKLLQFGWNLNQQFDNAEAEITSLSSPTLKDYHKILNKLFTSAADYHVNVSFYS 105
3DOR_A**  1 SLVCKNALQDLSFLEHLLQVKYAPKTWKEQYLGWDLVQSSVSAQQKLRTQENPSTSFCQQVLADFIGGLNDFHAGVTFFA 80

                 90       100       110       120       130       140       150       160
         ....*....|....*....|....*....|....*....|....*....|....*....|....*....|....*....|
273329 106 TEMASLPFHLQ-GAEGRYFitWINEEKLPEECldwSIGDEVIAFDGQDIHEFVQNLRRSEHdrHNEKTDQRFAERSLTLR 184
3DOR_A  81 IESAYLPYTVQkSSDGRFY--FVDIMTFSSEI---RVGDELLEVDGAPVQDVLATLYGSNH--KGTAAEESAALRTLFSR 153

                170       180       190       200       210       220       230       240
         ....*....|....*....|....*....|....*....|....*....|....*....|....*....|....*....|
273329 185 AGMFAEDVPQGNVEVDlIHKKTGEQKSYVLTWTYHPEQVKEIPL--PSLVARGISEK-QPFFQR---PYYKKMML--TPL 256
3DOR_A 154 MASLGHKVPSGRTTLK-IRRPFGTTREVRVKWRYVPEGVGDLATiaPSIRAPQLQKSmRSFFPKkddAFHRSSSLfySPM 232

                250       260       270       280       290       300       310       320
         ....*....|....*....|....*....|....*....|....*....|....*....|....*....|....*....|
273329 257 YAKF-----NQPESRVVEDVHNLGARKSFVPPLGSIVWQSppDSPFHAYICETP----SRQRIGYIRIATYDGTDDEVV- 326
3DOR_A 233 VPHFwaelrNHYATSGLKSGYNIGSTDGFLPVIGPVIWES--EGLFRAYISSVTdgdgKSHKVGFLRIPTYSWQDMEDFd 310

                330       340       350       360       370       380       390       400
         ....*....|....*....|....*....|....*....|....*....|....*....|....*....|....*....|
273329 327 --------EFAKLIDYFNPRTQALVVDQVNNPGGSVFYSYGLLSLLTDQPLELPKYKMAITQEEVMEALETLDTLKEITN 398
3DOR_A 311 psgpppweEFAKIIQVFSSNTEALIIDQTNNPGGSVLYLYALLSMLTDRPLELPKHRMILTQDEVVDALDWLTLLENVDT 390

                410       420       430       440       450       460       470       480
         ....*....|....*....|....*....|....*....|....*....|....*....|....*....|....*....|
273329 399 DEEAIDVCGPSLHGYAVSFELVQSVIQYFQFVLSEWEEGKY-VTSPVHLL-VNTIAPSPFVTYDKPMVVLVNEFDISCGD 476
3DOR_A 391 NVESRLALGDNMEGYTVDLQVAEYLKSFGRQVLNCWSKGDIeLSTPIPLFgFEKIHPHPRVQYSKPICVLINEQDFSCAD 470

                490       500       510       520       530       540       550       560
         ....*....|....*....|....*....|....*....|....*....|....*....|....*....|....*....|
273329 477 SFPCILQDNQRALIFGTRTAGAGGTVLGGGHPNRLGIAGYSYTASLLERHtDGTPIENLGVTPDVSYSLTPEDFQngYKG 556
3DOR_A 471 FFPVVLKDNDRALIVGTRTAGAGGFVFNVQFPNRTGIKTCSLTGSLAVRE-HGAFIENIGVEPHIDLPFTANDIR--YKG 547

         ....*..
273329 557 YKAALQE 563
3DOR_A 548 YSEYLDK 554
```

*273329 (Query): *Parachlamydia acanthamoebae* Bn9 peg.785

**3DOR_A: *Chlamydia trachomatis* Chain A, Crystal Structure Of Mature Cpaf
