# Supplementary material for: Amoebal Endosymbiont Parachlamydia acanthamoebae Bn9 Can Grow in Immortal Human Epithelial HEp-2 Cells at Low Temperature; An In Vitro Model System to Study Chlamydial Evolution
Source: PLoS One. 2015 Feb 2;10(2):e0116486. doi: 10.1371/journal.pone.0116486 (PMC4314085; doi:10.1371/journal.pone.0116486)
Supplement: S1 Table — (PDF) [file pone.0116486.s009.pdf]

Supplementary table 1. *Parachlamydia acanthamoebae* Bn$_9$ gene IDs with features

contig_id	feature_id	type	location	start	stop	strand	function	figfam	evidence_codes
NODE_111_length_231_cov_85.299271	fig|6666666.34159.peg.1	peg	NODE_111_length_231_cov_85.299271_6_200	6	200	+	hypothetical protein		
NODE_11_length_9935_cov_43.540089	fig|6666666.34159.peg.2	peg	NODE_11_length_9935_cov_43.540089_48_638	48	638	+	hypothetical protein		
NODE_11_length_9935_cov_43.540089	fig|6666666.34159.peg.3	peg	NODE_11_length_9935_cov_43.540089_1602_646	1602	646	-	hypothetical protein		
NODE_11_length_9935_cov_43.540089	fig|6666666.34159.peg.4	peg	NODE_11_length_9935_cov_43.540089_2349_3230	2349	3230	+	possible hydrolase		
NODE_11_length_9935_cov_43.540089	fig|6666666.34159.peg.5	peg	NODE_11_length_9935_cov_43.540089_3324_3208	3324	3208	-	hypothetical protein		
NODE_11_length_9935_cov_43.540089	fig|6666666.34159.peg.6	peg	NODE_11_length_9935_cov_43.540089_3279_4262	3279	4262	+	oxidoreductase, aldo/keto reductase family		
NODE_11_length_9935_cov_43.540089	fig|6666666.34159.peg.7	peg	NODE_11_length_9935_cov_43.540089_4293_5039	4293	5039	+	3-oxoacyl-[acyl-carrier protein] reductase (EC 1.1.1.100)	FIG00621114	idu(12);CBSS-246196.1.peg.364 idu(12);Fatty_Acid_Biosynthesis_FASII
NODE_11_length_9935_cov_43.540089	fig|6666666.34159.peg.8	peg	NODE_11_length_9935_cov_43.540089_5887_5081	5887	5081	-	hypothetical protein		
NODE_11_length_9935_cov_43.540089	fig|6666666.34159.peg.9	peg	NODE_11_length_9935_cov_43.540089_5994_7436	5994	7436	+	Putative nucleoside-diphosphate-sugar epimerase	FIG00868982	ff
NODE_11_length_9935_cov_43.540089	fig|6666666.34159.peg.10	peg	NODE_11_length_9935_cov_43.540089_8616_7420	8616	7420	-	drug resistance transporter, Bcr/CflA family		
NODE_11_length_9935_cov_43.540089	fig|6666666.34159.peg.11	peg	NODE_11_length_9935_cov_43.540089_9122_8709	9122	8709	-	239AB		
NODE_11_length_9935_cov_43.540089	fig|6666666.34159.peg.12	peg	NODE_11_length_9935_cov_43.540089_9187_9300	9187	9300	+	hypothetical protein		
NODE_11_length_9935_cov_43.540089	fig|6666666.34159.peg.13	peg	NODE_11_length_9935_cov_43.540089_9721_9389	9721	9389	-	hypothetical protein		
NODE_12_length_62042_cov_43.469894	fig|6666666.34159.peg.14	peg	NODE_12_length_62042_cov_43.469894_657_2201	657	2201	+	hypothetical protein	FIG00638284	ff
NODE_12_length_62042_cov_43.469894	fig|6666666.34159.peg.15	peg	NODE_12_length_62042_cov_43.469894_2317_2598	2317	2598	+	hypothetical protein		
NODE_12_length_62042_cov_43.469894	fig|6666666.34159.peg.16	peg	NODE_12_length_62042_cov_43.469894_2845_3981	2845	3981	+	hypothetical protein		
NODE_12_length_62042_cov_43.469894	fig|6666666.34159.peg.17	peg	NODE_12_length_62042_cov_43.469894_4049_5515	4049	5515	+	Peptidase S10, serine carboxypeptidase		
NODE_12_length_62042_cov_43.469894	fig|6666666.34159.peg.18	peg	NODE_12_length_62042_cov_43.469894_5594_6019	5594	6019	+	Sulfur acceptor protein SufE for iron-sulfur cluster assembly	FIG00073306	isu;Iron-sulfur_cluster_assembly
NODE_12_length_62042_cov_43.469894	fig|6666666.34159.peg.19	peg	NODE_12_length_62042_cov_43.469894_6117_7538	6117	7538	+	protein tyrosine phosphatase, receptor type, E [EC:3.1.3.48]		
NODE_12_length_62042_cov_43.469894	fig|6666666.34159.peg.20	peg	NODE_12_length_62042_cov_43.469894_8896_7577	8896	7577	-	hypothetical protein	FIG00638284	ff
NODE_12_length_62042_cov_43.469894	fig|6666666.34159.peg.21	peg	NODE_12_length_62042_cov_43.469894_11255_9285	11255	9285	-	chemotaxis transducer		
NODE_12_length_62042_cov_43.469894	fig|6666666.34159.peg.22	peg	NODE_12_length_62042_cov_43.469894_13335_11308	13335	11308	-	hypothetical protein	FIG00638284	ff
NODE_12_length_62042_cov_43.469894	fig|6666666.34159.peg.23	peg	NODE_12_length_62042_cov_43.469894_15115_13568	15115	13568	-	hypothetical protein	FIG00638284	ff
NODE_12_length_62042_cov_43.469894	fig|6666666.34159.peg.24	peg	NODE_12_length_62042_cov_43.469894_15926_15441	15926	15441	-	Nucleoside diphosphate kinase (EC 2.7.4.6)	FIG00000251	icw(1);Purine_conversions icw(1);pyrimidine_conversions
NODE_12_length_62042_cov_43.469894	fig|6666666.34159.peg.25	peg	NODE_12_length_62042_cov_43.469894_16418_15936	16418	15936	-	anthranilate/para-aminobenzoate synthases component II		
NODE_12_length_62042_cov_43.469894	fig|6666666.34159.peg.26	peg	NODE_12_length_62042_cov_43.469894_16946_16515	16946	16515	-	Nucleoside diphosphate kinase (EC 2.7.4.6)	FIG00000251	icw(1);Purine_conversions icw(1);pyrimidine_conversions
NODE_12_length_62042_cov_43.469894	fig|6666666.34159.peg.27	peg	NODE_12_length_62042_cov_43.469894_18569_17286	18569	17286	-	Isocitrate lyase (EC 4.1.3.1)	FIG00000818	isu;Glyoxylate_bypass isu;Glyoxylate_bypass_cluster
NODE_12_length_62042_cov_43.469894	fig|6666666.34159.peg.28	peg	NODE_12_length_62042_cov_43.469894_20180_18579	20180	18579	-	Malate synthase (EC 2.3.3.9)	FIG00001217	icw(1);Glyoxylate_bypass icw(1);Glyoxylate_bypass_cluster
NODE_12_length_62042_cov_43.469894	fig|6666666.34159.peg.29	peg	NODE_12_length_62042_cov_43.469894_21272_20274	21272	20274	-	hypothetical protein		
NODE_12_length_62042_cov_43.469894	fig|6666666.34159.peg.30	peg	NODE_12_length_62042_cov_43.469894_21413_21919	21413	21919	+	Non-specific DNA-binding protein Dps / Iron-binding ferritin-like antioxidant protein / Ferroxidase (EC 1.16.3.1)	FIG00109595	idu(1);Oxidative_stress idu(1);Oxidative_stress idu(1);Oxidative_stress
NODE_12_length_62042_cov_43.469894	fig|6666666.34159.peg.31	peg	NODE_12_length_62042_cov_43.469894_21976_22176	21976	22176	+	hypothetical protein		
NODE_12_length_62042_cov_43.469894	fig|6666666.34159.peg.32	peg	NODE_12_length_62042_cov_43.469894_22334_22864	22334	22864	+	hypothetical protein		
NODE_12_length_62042_cov_43.469894	fig|6666666.34159.peg.33	peg	NODE_12_length_62042_cov_43.469894_22994_25396	22994	25396	+	Phenylalanyl-tRNA synthetase beta chain (EC 6.1.1.20)	FIG00000116	isu;tRNA_aminoacylation,_Phe
NODE_12_length_62042_cov_43.469894	fig|6666666.34159.peg.34	peg	NODE_12_length_62042_cov_43.469894_25423_26391	25423	26391	+	hypothetical protein		
NODE_12_length_62042_cov_43.469894	fig|6666666.34159.peg.35	peg	NODE_12_length_62042_cov_43.469894_26977_26423	26977	26423	-	hypothetical protein		
NODE_12_length_62042_cov_43.469894	fig|6666666.34159.peg.36	peg	NODE_12_length_62042_cov_43.469894_27042_27587	27042	27587	+	Methylated-DNA--protein-cysteine methyltransferase (EC 2.1.1.63)	FIG00000328	idu(1);DNA_repair,_bacterial idu(1);CBSS-393124.3.peg.2657
NODE_12_length_62042_cov_43.469894	fig|6666666.34159.peg.37	peg	NODE_12_length_62042_cov_43.469894_27584_27781	27584	27781	+	hypothetical protein		
NODE_12_length_62042_cov_43.469894	fig|6666666.34159.peg.38	peg	NODE_12_length_62042_cov_43.469894_27900_28088	27900	28088	+	hypothetical protein		
NODE_12_length_62042_cov_43.469894	fig|6666666.34159.peg.39	peg	NODE_12_length_62042_cov_43.469894_28311_34967	28311	34967	+	hypothetical protein	FIG00638284	ff
NODE_12_length_62042_cov_43.469894	fig|6666666.34159.peg.40	peg	NODE_12_length_62042_cov_43.469894_35112_42023	35112	42023	+	hypothetical protein	FIG00638284	ff
NODE_12_length_62042_cov_43.469894	fig|6666666.34159.peg.41	peg	NODE_12_length_62042_cov_43.469894_42170_42331	42170	42331	+	hypothetical protein		
NODE_12_length_62042_cov_43.469894	fig|6666666.34159.peg.42	peg	NODE_12_length_62042_cov_43.469894_42744_50051	42744	50051	+	hypothetical protein	FIG00638284	ff
NODE_12_length_62042_cov_43.469894	fig|6666666.34159.peg.43	peg	NODE_12_length_62042_cov_43.469894_51128_50100	51128	50100	-	L-threonine 3-dehydrogenase (EC 1.1.1.103)	FIG00003022	isu;Threonine_degradation icw(1);Glycine_Biosynthesis
NODE_12_length_62042_cov_43.469894	fig|6666666.34159.peg.44	peg	NODE_12_length_62042_cov_43.469894_52426_51239	52426	51239	-	2-amino-3-ketobutyrate coenzyme A ligase (EC 2.3.1.29)	FIG00000741	isu;Glycine_and_Serine_Utilization isu;Glycine_Biosynthesis
NODE_12_length_62042_cov_43.469894	fig|6666666.34159.peg.45	peg	NODE_12_length_62042_cov_43.469894_52577_54949	52577	54949	+	Glucoamylase (EC 3.2.1.3)	FIG00012589	isu;Trehalose_Biosynthesis isu;Maltose_and_Maltodextrin_Utilization
NODE_12_length_62042_cov_43.469894	fig|6666666.34159.peg.46	peg	NODE_12_length_62042_cov_43.469894_55137_56135	55137	56135	+	cellulose 1,4-beta-cellobiosidase		
NODE_12_length_62042_cov_43.469894	fig|6666666.34159.peg.47	peg	NODE_12_length_62042_cov_43.469894_56295_58019	56295	58019	+	Pyruvate oxidase [ubiquinone, cytochrome] (EC 1.2.2.2)	FIG00001377	isu;Pyruvate_metabolism_II:_acetyl-CoA,_acetogenesis_from_pyruvate
NODE_12_length_62042_cov_43.469894	fig|6666666.34159.peg.48	peg	NODE_12_length_62042_cov_43.469894_58032_58211	58032	58211	+	hypothetical protein		
NODE_12_length_62042_cov_43.469894	fig|6666666.34159.peg.49	peg	NODE_12_length_62042_cov_43.469894_58225_58380	58225	58380	+	hypothetical protein	FIG00638284	ff
NODE_12_length_62042_cov_43.469894	fig|6666666.34159.peg.50	peg	NODE_12_length_62042_cov_43.469894_58412_59182	58412	59182	+	FIG003003: hypothetical protein	FIG00634249	ff
NODE_12_length_62042_cov_43.469894	fig|6666666.34159.peg.51	peg	NODE_12_length_62042_cov_43.469894_59333_59476	59333	59476	+	hypothetical protein		
NODE_12_length_62042_cov_43.469894	fig|6666666.34159.peg.52	peg	NODE_12_length_62042_cov_43.469894_59470_59586	59470	59586	+	hypothetical protein		
NODE_12_length_62042_cov_43.469894	fig|6666666.34159.peg.53	peg	NODE_12_length_62042_cov_43.469894_59742_59900	59742	59900	+	hypothetical protein	FIG00638284	ff
NODE_12_length_62042_cov_43.469894	fig|6666666.34159.peg.54	peg	NODE_12_length_62042_cov_43.469894_59888_60055	59888	60055	+	transposase, mutator type		
NODE_12_length_62042_cov_43.469894	fig|6666666.34159.peg.55	peg	NODE_12_length_62042_cov_43.469894_60126_60242	60126	60242	+	hypothetical protein		
NODE_12_length_62042_cov_43.469894	fig|6666666.34159.peg.56	peg	NODE_12_length_62042_cov_43.469894_60688_60428	60688	60428	-	hypothetical protein		
NODE_12_length_62042_cov_43.469894	fig|6666666.34159.peg.57	peg	NODE_12_length_62042_cov_43.469894_61490_61251	61490	61251	-	hypothetical protein		

NODE_12_length_62042_cov_43.469894	fig|6666666.34159.peg.58	peg	NODE_12_length_62042_cov_43.469894_61784_61518	61784	61518	-	hypothetical protein		
NODE_14_length_49616_cov_43.592384	fig|6666666.34159.peg.59	peg	NODE_14_length_49616_cov_43.592384_1728_1204	1728	1204	-	hypothetical protein		
NODE_14_length_49616_cov_43.592384	fig|6666666.34159.peg.60	peg	NODE_14_length_49616_cov_43.592384_1851_1991	1851	1991	+	hypothetical protein		
NODE_14_length_49616_cov_43.592384	fig|6666666.34159.peg.61	peg	NODE_14_length_49616_cov_43.592384_2372_2226	2372	2226	-	hypothetical protein		
NODE_14_length_49616_cov_43.592384	fig|6666666.34159.peg.62	peg	NODE_14_length_49616_cov_43.592384_7341_2803	7341	2803	-	hypothetical protein	FIG00638284	ff
NODE_14_length_49616_cov_43.592384	fig|6666666.34159.peg.63	peg	NODE_14_length_49616_cov_43.592384_9355_7379	9355	7379	-	Ankyrin repeat		
NODE_14_length_49616_cov_43.592384	fig|6666666.34159.peg.64	peg	NODE_14_length_49616_cov_43.592384_9531_10406	9531	10406	+	hypothetical protein		
NODE_14_length_49616_cov_43.592384	fig|6666666.34159.peg.65	peg	NODE_14_length_49616_cov_43.592384_11398_10559	11398	10559	-	protein of unknown function DUF81		
NODE_14_length_49616_cov_43.592384	fig|6666666.34159.peg.66	peg	NODE_14_length_49616_cov_43.592384_11397_11516	11397	11516	+	hypothetical protein		
NODE_14_length_49616_cov_43.592384	fig|6666666.34159.peg.67	peg	NODE_14_length_49616_cov_43.592384_11603_11478	11603	11478	-	hypothetical protein		
NODE_14_length_49616_cov_43.592384	fig|6666666.34159.peg.68	peg	NODE_14_length_49616_cov_43.592384_11602_12132	11602	12132	+	hypothetical protein		
NODE_14_length_49616_cov_43.592384	fig|6666666.34159.peg.69	peg	NODE_14_length_49616_cov_43.592384_12220_12840	12220	12840	+	Aquaporin Z	FIG00008396	isu;Osmoregulation
NODE_14_length_49616_cov_43.592384	fig|6666666.34159.peg.70	peg	NODE_14_length_49616_cov_43.592384_12857_13309	12857	13309	+	conserved hypothetical protein		
NODE_14_length_49616_cov_43.592384	fig|6666666.34159.peg.71	peg	NODE_14_length_49616_cov_43.592384_14655_13351	14655	13351	-	hypothetical protein	FIG00638284	ff
NODE_14_length_49616_cov_43.592384	fig|6666666.34159.peg.72	peg	NODE_14_length_49616_cov_43.592384_14875_14717	14875	14717	-	hypothetical protein		
NODE_14_length_49616_cov_43.592384	fig|6666666.34159.peg.73	peg	NODE_14_length_49616_cov_43.592384_16629_14947	16629	14947	-	hypothetical protein	FIG00638284	ff
NODE_14_length_49616_cov_43.592384	fig|6666666.34159.peg.74	peg	NODE_14_length_49616_cov_43.592384_18704_16653	18704	16653	-	hypothetical protein		
NODE_14_length_49616_cov_43.592384	fig|6666666.34159.peg.75	peg	NODE_14_length_49616_cov_43.592384_19085_19771	19085	19771	+	Methyltransferase FkbM		
NODE_14_length_49616_cov_43.592384	fig|6666666.34159.peg.76	peg	NODE_14_length_49616_cov_43.592384_19789_20643	19789	20643	+	Peptide methionine sulfoxide reductase MsrB (EC 1.8.4.12) / Peptide methionine sulfoxide reductase MsrA (EC 1.8.4.11)	FIG00019601	isu;Peptide_methionine_sulfoxide_reductase isu;Peptide_methionine_sulfoxide_reductase isu;Unknown_carbohydrate_utilization_(_cluster [illegible]
NODE_14_length_49616_cov_43.592384	fig|6666666.34159.peg.77	peg	NODE_14_length_49616_cov_43.592384_20651_21685	20651	21685	+	hypothetical protein		
NODE_14_length_49616_cov_43.592384	fig|6666666.34159.peg.78	peg	NODE_14_length_49616_cov_43.592384_23142_21682	23142	21682	-	3'-to-5' exoribonuclease RNase R	FIG00000308	idu(1);RNA_processing_and_degradation,_bacterial
NODE_14_length_49616_cov_43.592384	fig|6666666.34159.peg.79	peg	NODE_14_length_49616_cov_43.592384_23961_23158	23961	23158	-	cytochrome c family protein		
NODE_14_length_49616_cov_43.592384	fig|6666666.34159.peg.80	peg	NODE_14_length_49616_cov_43.592384_24160_24645	24160	24645	+	hypothetical protein		
NODE_14_length_49616_cov_43.592384	fig|6666666.34159.peg.81	peg	NODE_14_length_49616_cov_43.592384_25403_24603	25403	24603	-	extracellular solute-binding protein, family 3		
NODE_14_length_49616_cov_43.592384	fig|6666666.34159.peg.82	peg	NODE_14_length_49616_cov_43.592384_25558_26043	25558	26043	+	conserved protein		
NODE_14_length_49616_cov_43.592384	fig|6666666.34159.peg.83	peg	NODE_14_length_49616_cov_43.592384_26036_26245	26036	26245	+	hypothetical protein		
NODE_14_length_49616_cov_43.592384	fig|6666666.34159.peg.84	peg	NODE_14_length_49616_cov_43.592384_27668_26346	27668	26346	-	putative 7-dehydrocholesterol reductase	FIG01414196	ff
NODE_14_length_49616_cov_43.592384	fig|6666666.34159.peg.85	peg	NODE_14_length_49616_cov_43.592384_27833_30340	27833	30340	+	Glycogen phosphorylase (EC 2.4.1.1)	FIG00000476	idu(1);Glycogen_metabolism idu(1);Maltose_and_Maltodextrin_Utilization idu(1);Glycogen_metabolism_cluster [illegible]
NODE_14_length_49616_cov_43.592384	fig|6666666.34159.peg.86	peg	NODE_14_length_49616_cov_43.592384_33797_30375	33797	30375	-	Ankyrin		
NODE_14_length_49616_cov_43.592384	fig|6666666.34159.peg.87	peg	NODE_14_length_49616_cov_43.592384_38427_33973	38427	33973	-	hypothetical protein	FIG00638284	ff
NODE_14_length_49616_cov_43.592384	fig|6666666.34159.peg.88	peg	NODE_14_length_49616_cov_43.592384_38714_39370	38714	39370	+	hypothetical protein		
NODE_14_length_49616_cov_43.592384	fig|6666666.34159.peg.89	peg	NODE_14_length_49616_cov_43.592384_41221_39449	41221	39449	-	hypothetical protein		
NODE_14_length_49616_cov_43.592384	fig|6666666.34159.peg.90	peg	NODE_14_length_49616_cov_43.592384_43195_41375	43195	41375	-	hypothetical protein		
NODE_14_length_49616_cov_43.592384	fig|6666666.34159.peg.91	peg	NODE_14_length_49616_cov_43.592384_44376_43297	44376	43297	-	Beta-lactamase class C and other penicillin binding proteins	FIG00000681	isu;Beta-lactamase
NODE_14_length_49616_cov_43.592384	fig|6666666.34159.peg.92	peg	NODE_14_length_49616_cov_43.592384_44562_44714	44562	44714	+	hypothetical protein		
NODE_14_length_49616_cov_43.592384	fig|6666666.34159.peg.93	peg	NODE_14_length_49616_cov_43.592384_44722_45231	44722	45231	+	hypothetical protein		
NODE_14_length_49616_cov_43.592384	fig|6666666.34159.peg.94	peg	NODE_14_length_49616_cov_43.592384_45251_46279	45251	46279	+	possible oxalate decarboxylase		
NODE_14_length_49616_cov_43.592384	fig|6666666.34159.peg.95	peg	NODE_14_length_49616_cov_43.592384_46350_47024	46350	47024	+	hypothetical protein		
NODE_14_length_49616_cov_43.592384	fig|6666666.34159.peg.96	peg	NODE_14_length_49616_cov_43.592384_47625_47065	47625	47065	-	hypothetical protein		
NODE_14_length_49616_cov_43.592384	fig|6666666.34159.peg.97	peg	NODE_14_length_49616_cov_43.592384_47826_49217	47826	49217	+	Fumarate hydratase class II (EC 4.2.1.2)	FIG00000340	isu;TCA_Cycle
NODE_15_length_1635_cov_84.039581	fig|6666666.34159.peg.98	peg	NODE_15_length_1635_cov_84.039581_125_352	125	352	+	hypothetical protein		
NODE_15_length_1635_cov_84.039581	fig|6666666.34159.peg.99	peg	NODE_15_length_1635_cov_84.039581_1616_489	1616	489	-	hypothetical protein		
NODE_16_length_3420_cov_113.238724	fig|6666666.34159.peg.100	peg	NODE_16_length_3420_cov_113.238724_35_232	35	232	+	hypothetical protein		
NODE_16_length_3420_cov_113.238724	fig|6666666.34159.rna.1	rna	NODE_16_length_3420_cov_113.238724_3332_299	3332	299	-	Large Subunit Ribosomal RNA; lsuRNA; LSU rRNA		
NODE_1_length_34968_cov_44.611431	fig|6666666.34159.peg.102	peg	NODE_1_length_34968_cov_44.611431_638_1603	638	1603	+	Lipase	FIG01228631	ff
NODE_1_length_34968_cov_44.611431	fig|6666666.34159.rna.2	rna	NODE_1_length_34968_cov_44.611431_1716_1789	1716	1789	+	tRNA-Met-CAT		
NODE_1_length_34968_cov_44.611431	fig|6666666.34159.peg.103	peg	NODE_1_length_34968_cov_44.611431_1984_3087	1984	3087	+	Phage integrase		
NODE_1_length_34968_cov_44.611431	fig|6666666.34159.peg.104	peg	NODE_1_length_34968_cov_44.611431_3196_3966	3196	3966	+	hypothetical protein		
NODE_1_length_34968_cov_44.611431	fig|6666666.34159.peg.105	peg	NODE_1_length_34968_cov_44.611431_4005_4484	4005	4484	+	hypothetical protein		
NODE_1_length_34968_cov_44.611431	fig|6666666.34159.peg.106	peg	NODE_1_length_34968_cov_44.611431_5045_5182	5045	5182	+	hypothetical protein		
NODE_1_length_34968_cov_44.611431	fig|6666666.34159.peg.107	peg	NODE_1_length_34968_cov_44.611431_5172_7061	5172	7061	+	Phage/plasmid primase P4-like		
NODE_1_length_34968_cov_44.611431	fig|6666666.34159.peg.108	peg	NODE_1_length_34968_cov_44.611431_7134_7787	7134	7787	+	hypothetical protein		
NODE_1_length_34968_cov_44.611431	fig|6666666.34159.peg.109	peg	NODE_1_length_34968_cov_44.611431_7855_7989	7855	7989	+	hypothetical protein		
NODE_1_length_34968_cov_44.611431	fig|6666666.34159.peg.110	peg	NODE_1_length_34968_cov_44.611431_8593_8147	8593	8147	-	hypothetical protein		
NODE_1_length_34968_cov_44.611431	fig|6666666.34159.peg.111	peg	NODE_1_length_34968_cov_44.611431_8812_9066	8812	9066	+	transcriptional regulator, XRE family		
NODE_1_length_34968_cov_44.611431	fig|6666666.34159.peg.112	peg	NODE_1_length_34968_cov_44.611431_10698_9079	10698	9079	-	hypothetical protein	FIG00638284	ff
NODE_1_length_34968_cov_44.611431	fig|6666666.34159.peg.113	peg	NODE_1_length_34968_cov_44.611431_10965_10768	10965	10768	-	hypothetical protein		
NODE_1_length_34968_cov_44.611431	fig|6666666.34159.peg.114	peg	NODE_1_length_34968_cov_44.611431_11114_12121	11114	12121	+	uncharacterized membrane-anchored protein		
NODE_1_length_34968_cov_44.611431	fig|6666666.34159.peg.115	peg	NODE_1_length_34968_cov_44.611431_12118_12258	12118	12258	+	hypothetical protein		

NODE_1_length_34968_cov_44.611431	fig|6666666.34159.peg.116	peg	NODE_1_length_34968_cov_44.611431_12251_12805	12251	12805	+	hypothetical protein		
NODE_1_length_34968_cov_44.611431	fig|6666666.34159.peg.117	peg	NODE_1_length_34968_cov_44.611431_12802_13539	12802	13539	+	hypothetical protein		
NODE_1_length_34968_cov_44.611431	fig|6666666.34159.peg.118	peg	NODE_1_length_34968_cov_44.611431_13654_13809	13654	13809	+	hypothetical protein		
NODE_1_length_34968_cov_44.611431	fig|6666666.34159.peg.119	peg	NODE_1_length_34968_cov_44.611431_14496_13816	14496	13816	-	hypothetical protein		
NODE_1_length_34968_cov_44.611431	fig|6666666.34159.peg.120	peg	NODE_1_length_34968_cov_44.611431_15110_15391	15110	15391	+	hypothetical protein		
NODE_1_length_34968_cov_44.611431	fig|6666666.34159.peg.121	peg	NODE_1_length_34968_cov_44.611431_15402_15548	15402	15548	+	hypothetical protein		
NODE_1_length_34968_cov_44.611431	fig|6666666.34159.peg.122	peg	NODE_1_length_34968_cov_44.611431_15550_15798	15550	15798	+	hypothetical protein		
NODE_1_length_34968_cov_44.611431	fig|6666666.34159.peg.123	peg	NODE_1_length_34968_cov_44.611431_15858_15998	15858	15998	+	hypothetical protein		
NODE_1_length_34968_cov_44.611431	fig|6666666.34159.peg.124	peg	NODE_1_length_34968_cov_44.611431_16074_16391	16074	16391	+	hypothetical protein		
NODE_1_length_34968_cov_44.611431	fig|6666666.34159.peg.125	peg	NODE_1_length_34968_cov_44.611431_16418_16882	16418	16882	+	hypothetical protein		
NODE_1_length_34968_cov_44.611431	fig|6666666.34159.peg.126	peg	NODE_1_length_34968_cov_44.611431_17252_17461	17252	17461	+	hypothetical protein		
NODE_1_length_34968_cov_44.611431	fig|6666666.34159.peg.127	peg	NODE_1_length_34968_cov_44.611431_17658_17774	17658	17774	+	hypothetical protein		
NODE_1_length_34968_cov_44.611431	fig|6666666.34159.peg.128	peg	NODE_1_length_34968_cov_44.611431_17774_17995	17774	17995	+	hypothetical protein		
NODE_1_length_34968_cov_44.611431	fig|6666666.34159.peg.129	peg	NODE_1_length_34968_cov_44.611431_18196_18360	18196	18360	+	hypothetical protein		
NODE_1_length_34968_cov_44.611431	fig|6666666.34159.peg.130	peg	NODE_1_length_34968_cov_44.611431_18467_20338	18467	20338	+	hypothetical protein		
NODE_1_length_34968_cov_44.611431	fig|6666666.34159.peg.131	peg	NODE_1_length_34968_cov_44.611431_21319_20726	21319	20726	-	hypothetical protein		
NODE_1_length_34968_cov_44.611431	fig|6666666.34159.peg.132	peg	NODE_1_length_34968_cov_44.611431_21920_22567	21920	22567	+	hypothetical protein		
NODE_1_length_34968_cov_44.611431	fig|6666666.34159.peg.133	peg	NODE_1_length_34968_cov_44.611431_22582_23556	22582	23556	+	putative capsule biosynthesis protein		
NODE_1_length_34968_cov_44.611431	fig|6666666.34159.peg.134	peg	NODE_1_length_34968_cov_44.611431_23989_23678	23989	23678	-	hypothetical protein		
NODE_1_length_34968_cov_44.611431	fig|6666666.34159.peg.135	peg	NODE_1_length_34968_cov_44.611431_25136_24303	25136	24303	-	Aldo-keto reductase	FIG00639501	ff
NODE_1_length_34968_cov_44.611431	fig|6666666.34159.peg.136	peg	NODE_1_length_34968_cov_44.611431_25684_25169	25684	25169	-	DSBA oxidoreductase		
NODE_1_length_34968_cov_44.611431	fig|6666666.34159.peg.137	peg	NODE_1_length_34968_cov_44.611431_26164_25697	26164	25697	-	Alkyl hydroperoxide reductase subunit C-like protein	FIG01258694	idu(1);Oxidative_stress idu(1);Rubrerythrin idu(1);Thioredoxin-disulfide_reductase
NODE_1_length_34968_cov_44.611431	fig|6666666.34159.peg.138	peg	NODE_1_length_34968_cov_44.611431_26423_26545	26423	26545	+	hypothetical protein		
NODE_1_length_34968_cov_44.611431	fig|6666666.34159.peg.139	peg	NODE_1_length_34968_cov_44.611431_26615_28330	26615	28330	+	Arylsulfatase (EC 3.1.6.1)	FIG00016566	icw(1);Sulfatases_and_sulfatase_modifying_factor_1 isu;Galactosylceramide_and_Sulfatide_metabolis
NODE_1_length_34968_cov_44.611431	fig|6666666.34159.peg.140	peg	NODE_1_length_34968_cov_44.611431_28749_29774	28749	29774	+	Sulfatase modifying factor 1 precursor (C-alpha-formylglycine-generating enzyme 1)	FIG00003489	isu;Sulfatases_and_sulfatase_modifying_factor_1
NODE_1_length_34968_cov_44.611431	fig|6666666.34159.peg.141	peg	NODE_1_length_34968_cov_44.611431_29911_31131	29911	31131	+	hypothetical protein	FIG00638284	ff
NODE_1_length_34968_cov_44.611431	fig|6666666.34159.peg.142	peg	NODE_1_length_34968_cov_44.611431_31791_31189	31791	31189	-	Uracil phosphoribosyltransferase (EC 2.4.2.9)	FIG00000396	isu;De_Novo_Pyrimidine_Synthesis isu;pyrimidine_conversions
NODE_1_length_34968_cov_44.611431	fig|6666666.34159.peg.143	peg	NODE_1_length_34968_cov_44.611431_32665_32318	32665	32318	-	hypothetical protein		
NODE_1_length_34968_cov_44.611431	fig|6666666.34159.peg.144	peg	NODE_1_length_34968_cov_44.611431_34766_32760	34766	32760	-	hypothetical protein		
NODE_24_length_220_cov_285.777771	fig|6666666.34159.peg.145	peg	NODE_24_length_220_cov_285.777771_46_162	46	162	+	hypothetical protein		
NODE_28_length_58613_cov_42.360600	fig|6666666.34159.peg.146	peg	NODE_28_length_58613_cov_42.360600_857_708	857	708	-	hypothetical protein		
NODE_28_length_58613_cov_42.360600	fig|6666666.34159.peg.147	peg	NODE_28_length_58613_cov_42.360600_1148_1894	1148	1894	+	3-oxoacyl-[acyl-carrier protein] reductase (EC 1.1.1.100)	FIG00621114	idu(12);CBSS-246196.1.peg.364 idu(12);Fatty_Acid_Biosynthesis_FASII
NODE_28_length_58613_cov_42.360600	fig|6666666.34159.peg.148	peg	NODE_28_length_58613_cov_42.360600_1983_3473	1983	3473	+	ABC transporter, ATP-binding protein	FIG00744535	ff
NODE_28_length_58613_cov_42.360600	fig|6666666.34159.peg.149	peg	NODE_28_length_58613_cov_42.360600_3772_4899	3772	4899	+	Multicopper oxidase	FIG00060246	idu(1);Copper_homeostasis
NODE_28_length_58613_cov_42.360600	fig|6666666.34159.peg.150	peg	NODE_28_length_58613_cov_42.360600_6376_4859	6376	4859	-	hypothetical protein		
NODE_28_length_58613_cov_42.360600	fig|6666666.34159.peg.151	peg	NODE_28_length_58613_cov_42.360600_7532_6627	7532	6627	-	hypothetical protein		
NODE_28_length_58613_cov_42.360600	fig|6666666.34159.peg.152	peg	NODE_28_length_58613_cov_42.360600_8467_7646	8467	7646	-	3-oxoacyl-[acyl-carrier protein] reductase (EC 1.1.1.100)	FIG00621114	idu(12);CBSS-246196.1.peg.364 idu(12);Fatty_Acid_Biosynthesis_FASII
NODE_28_length_58613_cov_42.360600	fig|6666666.34159.peg.153	peg	NODE_28_length_58613_cov_42.360600_10631_8472	10631	8472	-	Membrane protein containing HD superfamily hydrolase domain, YQFF ortholog	FIG00002508	ff
NODE_28_length_58613_cov_42.360600	fig|6666666.34159.peg.154	peg	NODE_28_length_58613_cov_42.360600_10831_11802	10831	11802	+	UDP-glucose 4-epimerase (EC 5.1.3.2)	FIG00022300	idu(1);Rhamnose_containing_glycans idu(1);CBSS-296591.1.peg.2330 idu(1);Lactose_and_Galactose_Uptake_and_Utili
NODE_28_length_58613_cov_42.360600	fig|6666666.34159.peg.155	peg	NODE_28_length_58613_cov_42.360600_13619_11799	13619	11799	-	hypothetical protein		
NODE_28_length_58613_cov_42.360600	fig|6666666.34159.peg.156	peg	NODE_28_length_58613_cov_42.360600_13797_15335	13797	15335	+	DNA polymerase III subunits gamma and tau (EC 2.7.7.7)	FIG00000414	isu;DNA_processing_cluster
NODE_28_length_58613_cov_42.360600	fig|6666666.34159.peg.157	peg	NODE_28_length_58613_cov_42.360600_15350_15664	15350	15664	+	FIG00899469: hypothetical protein	FIG00899468	ff
NODE_28_length_58613_cov_42.360600	fig|6666666.34159.peg.158	peg	NODE_28_length_58613_cov_42.360600_17878_16109	17878	16109	-	Phosphoenolpyruvate-protein phosphotransferase of PTS system (EC 2.7.3.9)	FIG00028694	isu;Fructose_utilization isu;Mannitol_Utilization
NODE_28_length_58613_cov_42.360600	fig|6666666.34159.peg.159	peg	NODE_28_length_58613_cov_42.360600_18148_17879	18148	17879	-	Phosphocarrier protein of PTS system	FIG01954533	ff
NODE_28_length_58613_cov_42.360600	fig|6666666.34159.peg.160	peg	NODE_28_length_58613_cov_42.360600_19095_18145	19095	18145	-	HPr kinase/phosphorylase (EC 2.7.1.-) (EC 2.7.4.-)	FIG00004170	isu;HPr_catabolite_repression_system icw(1);Mannitol_Utilization
NODE_28_length_58613_cov_42.360600	fig|6666666.34159.peg.161	peg	NODE_28_length_58613_cov_42.360600_20203_19349	20203	19349	-	hypothetical protein	FIG00638284	ff
NODE_28_length_58613_cov_42.360600	fig|6666666.34159.peg.162	peg	NODE_28_length_58613_cov_42.360600_21516_20311	21516	20311	-	hypothetical protein	FIG00638284	ff
NODE_28_length_58613_cov_42.360600	fig|6666666.34159.peg.163	peg	NODE_28_length_58613_cov_42.360600_23630_21597	23630	21597	-	hypothetical protein	FIG00638284	ff
NODE_28_length_58613_cov_42.360600	fig|6666666.34159.peg.164	peg	NODE_28_length_58613_cov_42.360600_23776_25278	23776	25278	+	RNA polymerase sigma-54 factor RpoN	FIG00006092	isu;Flagellar_motility isu;Flagellum isu;Transcription_initiation,_bacterial_sigma_factors
NODE_28_length_58613_cov_42.360600	fig|6666666.34159.peg.165	peg	NODE_28_length_58613_cov_42.360600_26130_25258	26130	25258	-	hypothetical protein		
NODE_28_length_58613_cov_42.360600	fig|6666666.34159.peg.166	peg	NODE_28_length_58613_cov_42.360600_26129_26329	26129	26329	+	hypothetical protein		
NODE_28_length_58613_cov_42.360600	fig|6666666.34159.peg.167	peg	NODE_28_length_58613_cov_42.360600_26599_26925	26599	26925	+	hypothetical protein		
NODE_28_length_58613_cov_42.360600	fig|6666666.34159.peg.168	peg	NODE_28_length_58613_cov_42.360600_27058_27828	27058	27828	+	hypothetical protein	FIG00638284	ff
NODE_28_length_58613_cov_42.360600	fig|6666666.34159.peg.169	peg	NODE_28_length_58613_cov_42.360600_28484_30322	28484	30322	+	tRNA uridine 5-carboxymethylaminomethyl modification enzyme GidA	FIG00000576	isu;RNA_modification_and_chromosome_partitioning_cluster isu;tRNA_modification_Bacteria isu;mnm5U34_biosynthesis_bacteria
NODE_28_length_58613_cov_42.360600	fig|6666666.34159.peg.170	peg	NODE_28_length_58613_cov_42.360600_30330_31091	30330	31091	+	Lipoate-protein ligase A type 2	FIG00063913	isu;Lipoic_acid_metabolism isu;Glycine_cleavage_system
NODE_28_length_58613_cov_42.360600	fig|6666666.34159.peg.171	peg	NODE_28_length_58613_cov_42.360600_31750_31049	31750	31049	-	UDP-N-acetylglucosamine pyrophosphorylase related protein		
NODE_28_length_58613_cov_42.360600	fig|6666666.34159.peg.172	peg	NODE_28_length_58613_cov_42.360600_33412_31766	33412	31766	-	hypothetical protein	FIG00638284	ff
NODE_28_length_58613_cov_42.360600	fig|6666666.34159.peg.173	peg	NODE_28_length_58613_cov_42.360600_34019_33501	34019	33501	-	putative hydrolase		
NODE_28_length_58613_cov_42.360600	fig|6666666.34159.peg.174	peg	NODE_28_length_58613_cov_42.360600_34176_34000	34176	34000	-	hypothetical protein		

NODE_28_length_58613_cov_42.360600	fig|6666666.34159.peg.175	peg	NODE_28_length_58613_cov_42.360600_34283_34137	34283	34137	-	hypothetical protein		
NODE_28_length_58613_cov_42.360600	fig|6666666.34159.peg.176	peg	NODE_28_length_58613_cov_42.360600_35750_34392	35750	34392	-	Hexose phosphate uptake regulatory protein UhpC	FIG00004821	idu(1);Hexose_Phosphate_Uptake_System
NODE_28_length_58613_cov_42.360600	fig|6666666.34159.peg.177	peg	NODE_28_length_58613_cov_42.360600_36233_35943	36233	35943	-	SSU ribosomal protein S20p	FIG00000213	ff
NODE_28_length_58613_cov_42.360600	fig|6666666.34159.peg.178	peg	NODE_28_length_58613_cov_42.360600_36408_36277	36408	36277	-	hypothetical protein		
NODE_28_length_58613_cov_42.360600	fig|6666666.34159.peg.179	peg	NODE_28_length_58613_cov_42.360600_36544_38007	36544	38007	+	hypothetical protein	FIG00638284	ff
NODE_28_length_58613_cov_42.360600	fig|6666666.34159.peg.180	peg	NODE_28_length_58613_cov_42.360600_39119_38064	39119	38064	-	Flagellar hook-length control protein FliK		idu(1);Flagellum
NODE_28_length_58613_cov_42.360600	fig|6666666.34159.peg.181	peg	NODE_28_length_58613_cov_42.360600_39073_39216	39073	39216	+	hypothetical protein		
NODE_28_length_58613_cov_42.360600	fig|6666666.34159.peg.182	peg	NODE_28_length_58613_cov_42.360600_39230_39433	39230	39433	+	hypothetical protein		
NODE_28_length_58613_cov_42.360600	fig|6666666.34159.peg.183	peg	NODE_28_length_58613_cov_42.360600_39387_40745	39387	40745	+	type I secretion outer membrane protein		
NODE_28_length_58613_cov_42.360600	fig|6666666.34159.peg.184	peg	NODE_28_length_58613_cov_42.360600_40742_42220	40742	42220	+	hypothetical protein	FIG00638284	ff
NODE_28_length_58613_cov_42.360600	fig|6666666.34159.peg.185	peg	NODE_28_length_58613_cov_42.360600_42217_42894	42217	42894	+	ABC transporter, ATP-binding protein		
NODE_28_length_58613_cov_42.360600	fig|6666666.34159.peg.186	peg	NODE_28_length_58613_cov_42.360600_42884_44110	42884	44110	+	Macrolide export ATP-binding/permease protein MacB (EC 3.6.3.-)	FIG00001667	isu;Multidrug_Resistance_Efflux_Pumps
NODE_28_length_58613_cov_42.360600	fig|6666666.34159.peg.187	peg	NODE_28_length_58613_cov_42.360600_44949_44107	44949	44107	-	Competence protein, putative		
NODE_28_length_58613_cov_42.360600	fig|6666666.34159.peg.188	peg	NODE_28_length_58613_cov_42.360600_45241_45366	45241	45366	+	hypothetical protein		
NODE_28_length_58613_cov_42.360600	fig|6666666.34159.peg.189	peg	NODE_28_length_58613_cov_42.360600_45402_47042	45402	47042	+	RNA polymerase sigma factor RpoD	FIG00038814	isu;CBSS-349161.4.peg.2417 isu;Flagellum isu;Transcription_initiation,_bacterial_sigma_fact[illegible]
NODE_28_length_58613_cov_42.360600	fig|6666666.34159.peg.190	peg	NODE_28_length_58613_cov_42.360600_47115_46999	47115	46999	-	hypothetical protein		
NODE_28_length_58613_cov_42.360600	fig|6666666.34159.peg.191	peg	NODE_28_length_58613_cov_42.360600_47102_49333	47102	49333	+	DinG family ATP-dependent helicase YoaA	FIG00000680	isu;DNA_repair,_bacterial_DinG_and_relatives
NODE_28_length_58613_cov_42.360600	fig|6666666.34159.peg.192	peg	NODE_28_length_58613_cov_42.360600_50923_49550	50923	49550	-	hypothetical protein	FIG00638284	ff
NODE_28_length_58613_cov_42.360600	fig|6666666.34159.peg.193	peg	NODE_28_length_58613_cov_42.360600_51328_52362	51328	52362	+	hypothetical protein		
NODE_28_length_58613_cov_42.360600	fig|6666666.34159.rna.3	rna	NODE_28_length_58613_cov_42.360600_52725_52798	52725	52798	+	tRNA-His-GTG		
NODE_28_length_58613_cov_42.360600	fig|6666666.34159.peg.194	peg	NODE_28_length_58613_cov_42.360600_52938_53708	52938	53708	+	Undecaprenyl-diphosphatase (EC 3.6.1.27)	FIG00000555	ff
NODE_28_length_58613_cov_42.360600	fig|6666666.34159.peg.195	peg	NODE_28_length_58613_cov_42.360600_53762_56218	53762	56218	+	Cell division protein FtsK	FIG00000287	isu;Unspecified_monosaccharide_transport_cluster
NODE_28_length_58613_cov_42.360600	fig|6666666.34159.peg.196	peg	NODE_28_length_58613_cov_42.360600_56564_56220	56564	56220	-	hypothetical protein		
NODE_28_length_58613_cov_42.360600	fig|6666666.34159.peg.197	peg	NODE_28_length_58613_cov_42.360600_57954_56665	57954	56665	-	hypothetical protein		
NODE_28_length_58613_cov_42.360600	fig|6666666.34159.peg.198	peg	NODE_28_length_58613_cov_42.360600_58172_57978	58172	57978	-	hypothetical protein		
NODE_2_length_244240_cov_42.654095	fig|6666666.34159.peg.199	peg	NODE_2_length_244240_cov_42.654095_2200_200	2200	200	-	Glycogen debranching enzyme (EC 3.2.1.-)	FIG00109688	isu;Trehalose_Biosynthesis isu;Glycogen_metabolism isu;Glycogen_metabolism_cluster
NODE_2_length_244240_cov_42.654095	fig|6666666.34159.peg.200	peg	NODE_2_length_244240_cov_42.654095_2577_3083	2577	3083	+	FIG00493912: hypothetical protein	FIG00493911	ff
NODE_2_length_244240_cov_42.654095	fig|6666666.34159.peg.201	peg	NODE_2_length_244240_cov_42.654095_3983_3135	3983	3135	-	hypothetical protein		
NODE_2_length_244240_cov_42.654095	fig|6666666.34159.peg.202	peg	NODE_2_length_244240_cov_42.654095_4140_4628	4140	4628	+	single-strand DNA-binding protein		
NODE_2_length_244240_cov_42.654095	fig|6666666.34159.peg.203	peg	NODE_2_length_244240_cov_42.654095_5704_4625	5704	4625	-	DNA polymerase IV (EC 2.7.7.7)	FIG00023943	isu;DNA_repair,_bacterial
NODE_2_length_244240_cov_42.654095	fig|6666666.34159.peg.204	peg	NODE_2_length_244240_cov_42.654095_5888_6136	5888	6136	+	hypothetical protein		
NODE_2_length_244240_cov_42.654095	fig|6666666.34159.peg.205	peg	NODE_2_length_244240_cov_42.654095_6279_6938	6279	6938	+	hypothetical protein		
NODE_2_length_244240_cov_42.654095	fig|6666666.34159.peg.206	peg	NODE_2_length_244240_cov_42.654095_6931_8496	6931	8496	+	N-acetylneuraminate synthase (EC 2.5.1.56)	FIG00008273	isu;CMP-N-acetylneuraminate_Biosynthesis isu;Sialic_Acid_Metabolism
NODE_2_length_244240_cov_42.654095	fig|6666666.34159.peg.207	peg	NODE_2_length_244240_cov_42.654095_8493_9158	8493	9158	+	HAD-superfamily hydrolase, subfamily IA, variant 3		
NODE_2_length_244240_cov_42.654095	fig|6666666.34159.peg.208	peg	NODE_2_length_244240_cov_42.654095_9121_9813	9121	9813	+	NUDIX hydrolase		
NODE_2_length_244240_cov_42.654095	fig|6666666.34159.peg.209	peg	NODE_2_length_244240_cov_42.654095_9873_10898	9873	10898	+	DNA polymerase III delta subunit (EC 2.7.7.7)	FIG00106663	ff
NODE_2_length_244240_cov_42.654095	fig|6666666.34159.peg.210	peg	NODE_2_length_244240_cov_42.654095_11368_10931	11368	10931	-	hypothetical protein		
NODE_2_length_244240_cov_42.654095	fig|6666666.34159.peg.211	peg	NODE_2_length_244240_cov_42.654095_11513_11334	11513	11334	-	hypothetical protein		
NODE_2_length_244240_cov_42.654095	fig|6666666.34159.peg.212	peg	NODE_2_length_244240_cov_42.654095_12344_11667	12344	11667	-	hypothetical protein		
NODE_2_length_244240_cov_42.654095	fig|6666666.34159.peg.213	peg	NODE_2_length_244240_cov_42.654095_12550_13266	12550	13266	+	SAM-dependent methyltransferase	FIG00494178	ff
NODE_2_length_244240_cov_42.654095	fig|6666666.34159.peg.214	peg	NODE_2_length_244240_cov_42.654095_13496_14044	13496	14044	+	FIG00494315: hypothetical protein	FIG00493199	ff
NODE_2_length_244240_cov_42.654095	fig|6666666.34159.peg.215	peg	NODE_2_length_244240_cov_42.654095_14100_15254	14100	15254	+	Hypothetical radical SAM family enzyme, NOT coproporphyrinogen III oxidase, oxygen-independent	FIG00105365	isu;Heat_shock_dnaK_gene_cluster_extended isu;Heme_and_Siroheme_Biosynthesis
NODE_2_length_244240_cov_42.654095	fig|6666666.34159.peg.216	peg	NODE_2_length_244240_cov_42.654095_15622_15251	15622	15251	-	hypothetical protein		
NODE_2_length_244240_cov_42.654095	fig|6666666.34159.peg.217	peg	NODE_2_length_244240_cov_42.654095_16304_15960	16304	15960	-	hypothetical protein		
NODE_2_length_244240_cov_42.654095	fig|6666666.34159.peg.218	peg	NODE_2_length_244240_cov_42.654095_17151_16384	17151	16384	-	6-phosphogluconolactonase (EC 3.1.1.31), eukaryotic type	FIG00000776	icw(1);Pentose_phosphate_pathway
NODE_2_length_244240_cov_42.654095	fig|6666666.34159.peg.219	peg	NODE_2_length_244240_cov_42.654095_18261_17161	18261	17161	-	OpcA, an allosteric effector of glucose-6-phosphate dehydrogenase, cyanobacterial		
NODE_2_length_244240_cov_42.654095	fig|6666666.34159.peg.220	peg	NODE_2_length_244240_cov_42.654095_19833_18277	19833	18277	-	Glucose-6-phosphate 1-dehydrogenase (EC 1.1.1.49)	FIG00000332	isu;Pentose_phosphate_pathway
NODE_2_length_244240_cov_42.654095	fig|6666666.34159.peg.221	peg	NODE_2_length_244240_cov_42.654095_19921_20139	19921	20139	+	hypothetical protein		
NODE_2_length_244240_cov_42.654095	fig|6666666.34159.peg.222	peg	NODE_2_length_244240_cov_42.654095_20228_20446	20228	20446	+	hypothetical protein		
NODE_2_length_244240_cov_42.654095	fig|6666666.34159.peg.223	peg	NODE_2_length_244240_cov_42.654095_20797_20411	20797	20411	-	hypothetical protein		
NODE_2_length_244240_cov_42.654095	fig|6666666.34159.peg.224	peg	NODE_2_length_244240_cov_42.654095_21431_21123	21431	21123	-	hypothetical protein		
NODE_2_length_244240_cov_42.654095	fig|6666666.34159.peg.225	peg	NODE_2_length_244240_cov_42.654095_21414_21764	21414	21764	+	hypothetical protein		
NODE_2_length_244240_cov_42.654095	fig|6666666.34159.peg.226	peg	NODE_2_length_244240_cov_42.654095_21860_22486	21860	22486	+	phage integrase family protein		
NODE_2_length_244240_cov_42.654095	fig|6666666.34159.peg.227	peg	NODE_2_length_244240_cov_42.654095_22473_23189	22473	23189	+	hypothetical protein		
NODE_2_length_244240_cov_42.654095	fig|6666666.34159.peg.228	peg	NODE_2_length_244240_cov_42.654095_23274_23471	23274	23471	+	hypothetical protein		
NODE_2_length_244240_cov_42.654095	fig|6666666.34159.peg.229	peg	NODE_2_length_244240_cov_42.654095_23474_23791	23474	23791	+	hypothetical protein		
NODE_2_length_244240_cov_42.654095	fig|6666666.34159.peg.230	peg	NODE_2_length_244240_cov_42.654095_23776_25962	23776	25962	+	hypothetical protein	FIG00638284	ff
NODE_2_length_244240_cov_42.654095	fig|6666666.34159.peg.231	peg	NODE_2_length_244240_cov_42.654095_26045_26401	26045	26401	+	hypothetical protein		
NODE_2_length_244240_cov_42.654095	fig|6666666.34159.peg.232	peg	NODE_2_length_244240_cov_42.654095_26402_26608	26402	26608	+	hypothetical protein		

NODE_2_length_244240_cov_42.654095	fig|6666666.34159.peg.233	peg	NODE_2_length_244240_cov_42.654095_26702_26932	26702	26932	+	transcriptional regulator, XRE family		
NODE_2_length_244240_cov_42.654095	fig|6666666.34159.peg.234	peg	NODE_2_length_244240_cov_42.654095_26922_29411	26922	29411	+	Type I restriction-modification system, DNA-methyltransferase subunit M (EC 2.1.1.72)	FIG00000477	icw(1);Restriction-Modification_System icw(1);Type_I_Restriction-Modification
NODE_2_length_244240_cov_42.654095	fig|6666666.34159.peg.235	peg	NODE_2_length_244240_cov_42.654095_29408_30508	29408	30508	+	MloA protein, putative	FIG01450305	ff
NODE_2_length_244240_cov_42.654095	fig|6666666.34159.peg.236	peg	NODE_2_length_244240_cov_42.654095_30699_32021	30699	32021	+	Type I restriction-modification system, specificity subunit S (EC 3.1.21.3)	FIG00000494	isu;Restriction-Modification_System isu;Type_I_Restriction-Modification
NODE_2_length_244240_cov_42.654095	fig|6666666.34159.peg.237	peg	NODE_2_length_244240_cov_42.654095_32018_35137	32018	35137	+	Type I restriction-modification system, restriction subunit R (EC 3.1.21.3)	FIG00016514	icw(1);Restriction-Modification_System icw(1);Type_I_Restriction-Modification
NODE_2_length_244240_cov_42.654095	fig|6666666.34159.peg.238	peg	NODE_2_length_244240_cov_42.654095_35140_35853	35140	35853	+	Putative predicted metal-dependent hydrolase	FIG00002849	icw(2);Restriction-Modification_System
NODE_2_length_244240_cov_42.654095	fig|6666666.34159.peg.239	peg	NODE_2_length_244240_cov_42.654095_35980_36876	35980	36876	+	hypothetical protein		
NODE_2_length_244240_cov_42.654095	fig|6666666.34159.peg.240	peg	NODE_2_length_244240_cov_42.654095_36889_37695	36889	37695	+	hypothetical protein		
NODE_2_length_244240_cov_42.654095	fig|6666666.34159.peg.241	peg	NODE_2_length_244240_cov_42.654095_37826_38221	37826	38221	+	conserved hypothetical protein		
NODE_2_length_244240_cov_42.654095	fig|6666666.34159.peg.242	peg	NODE_2_length_244240_cov_42.654095_38209_38580	38209	38580	+	hypothetical protein		
NODE_2_length_244240_cov_42.654095	fig|6666666.34159.peg.243	peg	NODE_2_length_244240_cov_42.654095_39394_38879	39394	38879	-	Protein of unknown function DUF55	FIG00984653	ff
NODE_2_length_244240_cov_42.654095	fig|6666666.34159.peg.244	peg	NODE_2_length_244240_cov_42.654095_39921_39454	39921	39454	-	Putative Holliday junction resolvase (EC 3.1.-.-)	FIG00010099	ff
NODE_2_length_244240_cov_42.654095	fig|6666666.34159.peg.245	peg	NODE_2_length_244240_cov_42.654095_41540_39918	41540	39918	-	CTP synthase (EC 6.3.4.2)	FIG00000176	isu;CTP_synthase_(EC_6.3.4.2)_cluster isu;pyrimidine_conversions
NODE_2_length_244240_cov_42.654095	fig|6666666.34159.peg.246	peg	NODE_2_length_244240_cov_42.654095_42286_41492	42286	41492	-	3-deoxy-manno-octulosonate cytidylyltransferase (EC 2.7.7.38)	FIG00000603	isu;KDO2-Lipid_A_biosynthesis
NODE_2_length_244240_cov_42.654095	fig|6666666.34159.peg.247	peg	NODE_2_length_244240_cov_42.654095_42439_52524	42439	52524	+	hypothetical protein	FIG00638284	ff
NODE_2_length_244240_cov_42.654095	fig|6666666.34159.peg.248	peg	NODE_2_length_244240_cov_42.654095_52538_53077	52538	53077	+	hypothetical protein		
NODE_2_length_244240_cov_42.654095	fig|6666666.34159.peg.249	peg	NODE_2_length_244240_cov_42.654095_53138_53323	53138	53323	+	hypothetical protein		
NODE_2_length_244240_cov_42.654095	fig|6666666.34159.peg.250	peg	NODE_2_length_244240_cov_42.654095_53619_54851	53619	54851	+	WD-repeat protein		
NODE_2_length_244240_cov_42.654095	fig|6666666.34159.peg.251	peg	NODE_2_length_244240_cov_42.654095_55158_57188	55158	57188	+	hypothetical protein		
NODE_2_length_244240_cov_42.654095	fig|6666666.34159.peg.252	peg	NODE_2_length_244240_cov_42.654095_57313_59397	57313	59397	+	hypothetical protein		
NODE_2_length_244240_cov_42.654095	fig|6666666.34159.peg.253	peg	NODE_2_length_244240_cov_42.654095_61252_59420	61252	59420	-	GTP-binding protein TypA/BipA	FIG00000268	ff
NODE_2_length_244240_cov_42.654095	fig|6666666.34159.peg.254	peg	NODE_2_length_244240_cov_42.654095_61601_63100	61601	63100	+	Mg/Co/Ni transporter MgtE / CBS domain	FIG00437800	idu(2);Magnesium_transport
NODE_2_length_244240_cov_42.654095	fig|6666666.34159.peg.255	peg	NODE_2_length_244240_cov_42.654095_63119_63811	63119	63811	+	hypothetical protein		
NODE_2_length_244240_cov_42.654095	fig|6666666.34159.peg.256	peg	NODE_2_length_244240_cov_42.654095_64223_65392	64223	65392	+	hypothetical protein		
NODE_2_length_244240_cov_42.654095	fig|6666666.34159.peg.257	peg	NODE_2_length_244240_cov_42.654095_66104_65442	66104	65442	-	mcbg protein, putative		
NODE_2_length_244240_cov_42.654095	fig|6666666.34159.peg.258	peg	NODE_2_length_244240_cov_42.654095_66365_66126	66365	66126	-	hypothetical protein		
NODE_2_length_244240_cov_42.654095	fig|6666666.34159.peg.259	peg	NODE_2_length_244240_cov_42.654095_67519_66710	67519	66710	-	Beta-lactamase class D	FIG00029203	isu;Beta-lactamase
NODE_2_length_244240_cov_42.654095	fig|6666666.34159.peg.260	peg	NODE_2_length_244240_cov_42.654095_67698_68594	67698	68594	+	PROBABLE CONSERVED LIPOPROTEIN LPQO	FIG01320919	ff
NODE_2_length_244240_cov_42.654095	fig|6666666.34159.peg.261	peg	NODE_2_length_244240_cov_42.654095_69832_68798	69832	68798	-	hypothetical protein		
NODE_2_length_244240_cov_42.654095	fig|6666666.34159.peg.262	peg	NODE_2_length_244240_cov_42.654095_70532_69924	70532	69924	-	hypothetical protein		
NODE_2_length_244240_cov_42.654095	fig|6666666.34159.peg.263	peg	NODE_2_length_244240_cov_42.654095_71335_70727	71335	70727	-	hypothetical protein		
NODE_2_length_244240_cov_42.654095	fig|6666666.34159.peg.264	peg	NODE_2_length_244240_cov_42.654095_71377_71496	71377	71496	+	hypothetical protein		
NODE_2_length_244240_cov_42.654095	fig|6666666.34159.peg.265	peg	NODE_2_length_244240_cov_42.654095_71480_71605	71480	71605	+	hypothetical protein		
NODE_2_length_244240_cov_42.654095	fig|6666666.34159.rna.4	rna	NODE_2_length_244240_cov_42.654095_71627_71698	71627	71698	+	tRNA-Gln-TTG		
NODE_2_length_244240_cov_42.654095	fig|6666666.34159.peg.266	peg	NODE_2_length_244240_cov_42.654095_71726_72688	71726	72688	+	Ribose-phosphate pyrophosphokinase (EC 2.7.6.1)	FIG00096830	isu;Pentose_phosphate_pathway isu;De_Novo_Purine_Biosynthesis
NODE_2_length_244240_cov_42.654095	fig|6666666.34159.peg.267	peg	NODE_2_length_244240_cov_42.654095_72791_73351	72791	73351	+	LSU ribosomal protein L25p	FIG00001896	ff
NODE_2_length_244240_cov_42.654095	fig|6666666.34159.peg.268	peg	NODE_2_length_244240_cov_42.654095_73362_73958	73362	73958	+	Peptidyl-tRNA hydrolase (EC 3.1.1.29)	FIG00000151	isu;Cell_division-ribosomal_stress_proteins_cluster isu;Sporulation
NODE_2_length_244240_cov_42.654095	fig|6666666.34159.peg.269	peg	NODE_2_length_244240_cov_42.654095_74009_74353	74009	74353	+	SSU ribosomal protein S6p	FIG00000170	ff
NODE_2_length_244240_cov_42.654095	fig|6666666.34159.peg.270	peg	NODE_2_length_244240_cov_42.654095_74367_74624	74367	74624	+	SSU ribosomal protein S18p @ SSU ribosomal protein S18p, zinc-independent	FIG00000127	isu;Staphylococcal_pathogenicity_islands_SaPI
NODE_2_length_244240_cov_42.654095	fig|6666666.34159.peg.271	peg	NODE_2_length_244240_cov_42.654095_74651_75148	74651	75148	+	LSU ribosomal protein L9p	FIG00000205	ff
NODE_2_length_244240_cov_42.654095	fig|6666666.34159.peg.272	peg	NODE_2_length_244240_cov_42.654095_75185_76066	75185	76066	+	4-diphosphocytidyl-2-C-methyl-D-erythritol kinase (EC 2.7.1.148)	FIG00000377	isu;Nonmevalonate_Branch_of_Isoprenoid_Biosynthesis isu;Isoprenoid_Biosynthesis
NODE_2_length_244240_cov_42.654095	fig|6666666.34159.peg.273	peg	NODE_2_length_244240_cov_42.654095_79475_76032	79475	76032	-	hypothetical protein	FIG00638284	ff
NODE_2_length_244240_cov_42.654095	fig|6666666.34159.peg.274	peg	NODE_2_length_244240_cov_42.654095_80758_79472	80758	79472	-	DNA repair exonuclease family protein YhaO	FIG01303866	isu;DNA_repair,_bacterial
NODE_2_length_244240_cov_42.654095	fig|6666666.34159.peg.275	peg	NODE_2_length_244240_cov_42.654095_81752_80751	81752	80751	-	Biotin synthase (EC 2.8.1.6)	FIG00000467	isu;Biotin_biosynthesis_Experimental isu;Biotin_synthesis_cluster isu;Biotin_biosynthesis
NODE_2_length_244240_cov_42.654095	fig|6666666.34159.peg.276	peg	NODE_2_length_244240_cov_42.654095_82039_82233	82039	82233	+	hypothetical protein		
NODE_2_length_244240_cov_42.654095	fig|6666666.34159.peg.277	peg	NODE_2_length_244240_cov_42.654095_82986_83717	82986	83717	+	hypothetical protein		
NODE_2_length_244240_cov_42.654095	fig|6666666.34159.peg.278	peg	NODE_2_length_244240_cov_42.654095_84114_84293	84114	84293	+	hypothetical protein		
NODE_2_length_244240_cov_42.654095	fig|6666666.34159.peg.279	peg	NODE_2_length_244240_cov_42.654095_84997_84503	84997	84503	-	GCN5-related N-acetyltransferase		
NODE_2_length_244240_cov_42.654095	fig|6666666.34159.peg.280	peg	NODE_2_length_244240_cov_42.654095_85158_85811	85158	85811	+	hypothetical protein		
NODE_2_length_244240_cov_42.654095	fig|6666666.34159.peg.281	peg	NODE_2_length_244240_cov_42.654095_87948_85864	87948	85864	-	Transglutaminase-like enzyme		
NODE_2_length_244240_cov_42.654095	fig|6666666.34159.peg.282	peg	NODE_2_length_244240_cov_42.654095_88155_88751	88155	88751	+	hypothetical protein		
NODE_2_length_244240_cov_42.654095	fig|6666666.34159.peg.283	peg	NODE_2_length_244240_cov_42.654095_90438_88834	90438	88834	-	hypothetical protein	FIG00638284	ff
NODE_2_length_244240_cov_42.654095	fig|6666666.34159.peg.284	peg	NODE_2_length_244240_cov_42.654095_91014_90601	91014	90601	-	4-hydroxybenzoyl-CoA thioesterase family active site	FIG00002515	isu;Ton_and_Tol_transport_systems
NODE_2_length_244240_cov_42.654095	fig|6666666.34159.peg.285	peg	NODE_2_length_244240_cov_42.654095_91091_91720	91091	91720	+	hypothetical protein		
NODE_2_length_244240_cov_42.654095	fig|6666666.34159.peg.286	peg	NODE_2_length_244240_cov_42.654095_91810_92193	91810	92193	+	biphenyl-2,3-diol 1,2-dioxygenase III-related protein	FIG00105129	isu;Biphenyl_Degradation
NODE_2_length_244240_cov_42.654095	fig|6666666.34159.peg.287	peg	NODE_2_length_244240_cov_42.654095_92327_92202	92327	92202	-	hypothetical protein		
NODE_2_length_244240_cov_42.654095	fig|6666666.34159.peg.288	peg	NODE_2_length_244240_cov_42.654095_92286_93146	92286	93146	+	hypothetical protein	FIG00638284	ff
NODE_2_length_244240_cov_42.654095	fig|6666666.34159.peg.289	peg	NODE_2_length_244240_cov_42.654095_93224_95134	93224	95134	+	Peptidase S9, prolyl oligopeptidase active site region precursor	FIG00839635	ff
NODE_2_length_244240_cov_42.654095	fig|6666666.34159.peg.290	peg	NODE_2_length_244240_cov_42.654095_95571_95131	95571	95131	-	hypothetical protein		

NODE_2_length_244240_cov_42.654095	fig|6666666.34159.peg.291	peg	NODE_2_length_244240_cov_42.654095_96004_95531	96004	95531	-	hypothetical protein		
NODE_2_length_244240_cov_42.654095	fig|6666666.34159.peg.292	peg	NODE_2_length_244240_cov_42.654095_96569_96354	96569	96354	-	hypothetical protein		
NODE_2_length_244240_cov_42.654095	fig|6666666.34159.peg.293	peg	NODE_2_length_244240_cov_42.654095_96758_97477	96758	97477	+	Mg(2+) transport ATPase protein C	FIG00436510	isu;Magnesium_transport
NODE_2_length_244240_cov_42.654095	fig|6666666.34159.peg.294	peg	NODE_2_length_244240_cov_42.654095_97510_98481	97510	98481	+	Quinone oxidoreductase (EC 1.6.5.5)	FIG00002982	idu(1);Quinone_oxidoreductase_family
NODE_2_length_244240_cov_42.654095	fig|6666666.34159.peg.295	peg	NODE_2_length_244240_cov_42.654095_99808_98597	99808	98597	-	Na+/H+ antiporter	FIG00008246	ff
NODE_2_length_244240_cov_42.654095	fig|6666666.34159.peg.296	peg	NODE_2_length_244240_cov_42.654095_100610_102595	100610	1E+05	+	hypothetical protein	FIG00638284	ff
NODE_2_length_244240_cov_42.654095	fig|6666666.34159.peg.297	peg	NODE_2_length_244240_cov_42.654095_103081_102722	103081	1E+05	-	hypothetical protein		
NODE_2_length_244240_cov_42.654095	fig|6666666.34159.peg.298	peg	NODE_2_length_244240_cov_42.654095_103892_104005	103892	1E+05	+	hypothetical protein		
NODE_2_length_244240_cov_42.654095	fig|6666666.34159.peg.299	peg	NODE_2_length_244240_cov_42.654095_105326_104049	105326	1E+05	-	Multicopper oxidase	FIG00060246	idu(1);Copper_homeostasis
NODE_2_length_244240_cov_42.654095	fig|6666666.34159.peg.300	peg	NODE_2_length_244240_cov_42.654095_106699_105323	106699	1E+05	-	Copper tolerance protein	FIG00004286	icw(1);Copper_homeostasis isu;Copper_transport_and_blue_copper_proteins
NODE_2_length_244240_cov_42.654095	fig|6666666.34159.peg.301	peg	NODE_2_length_244240_cov_42.654095_106839_109484	106839	1E+05	+	hypothetical protein	FIG00638284	ff
NODE_2_length_244240_cov_42.654095	fig|6666666.34159.peg.302	peg	NODE_2_length_244240_cov_42.654095_109558_109785	109558	1E+05	+	hypothetical protein		
NODE_2_length_244240_cov_42.654095	fig|6666666.34159.peg.303	peg	NODE_2_length_244240_cov_42.654095_109927_111216	109927	1E+05	+	hypothetical protein	FIG00638284	ff
NODE_2_length_244240_cov_42.654095	fig|6666666.34159.peg.304	peg	NODE_2_length_244240_cov_42.654095_111667_112545	111667	1E+05	+	3-oxoacyl-[acyl-carrier protein] reductase (EC 1.1.1.100)	FIG00621114	idu(12);CBSS-246196.1.peg.364 idu(12);Fatty_Acid_Biosynthesis_FASII
NODE_2_length_244240_cov_42.654095	fig|6666666.34159.peg.305	peg	NODE_2_length_244240_cov_42.654095_112645_113142	112645	1E+05	+	hypothetical protein		
NODE_2_length_244240_cov_42.654095	fig|6666666.34159.peg.306	peg	NODE_2_length_244240_cov_42.654095_113396_113172	113396	1E+05	-	hypothetical protein		
NODE_2_length_244240_cov_42.654095	fig|6666666.34159.peg.307	peg	NODE_2_length_244240_cov_42.654095_113979_113416	113979	1E+05	-	hypothetical protein		
NODE_2_length_244240_cov_42.654095	fig|6666666.34159.peg.308	peg	NODE_2_length_244240_cov_42.654095_114297_114079	114297	1E+05	-	Carbonic anhydrase (EC 4.2.1.1)		icw(1);Cyanate_hydrolysis
NODE_2_length_244240_cov_42.654095	fig|6666666.34159.peg.309	peg	NODE_2_length_244240_cov_42.654095_114427_114308	114427	1E+05	-	hypothetical protein		
NODE_2_length_244240_cov_42.654095	fig|6666666.34159.peg.310	peg	NODE_2_length_244240_cov_42.654095_115752_114496	115752	1E+05	-	Mg(2+) transport ATPase, P-type (EC 3.6.3.2)	FIG01123715	icw(1);Magnesium_transport
NODE_2_length_244240_cov_42.654095	fig|6666666.34159.peg.311	peg	NODE_2_length_244240_cov_42.654095_116819_115803	116819	1E+05	-	Mg(2+) transport ATPase, P-type (EC 3.6.3.2)	FIG01123715	icw(1);Magnesium_transport
NODE_2_length_244240_cov_42.654095	fig|6666666.34159.peg.312	peg	NODE_2_length_244240_cov_42.654095_117166_116834	117166	1E+05	-	hypothetical protein		
NODE_2_length_244240_cov_42.654095	fig|6666666.34159.peg.313	peg	NODE_2_length_244240_cov_42.654095_119660_117375	119660	1E+05	-	Carbonic anhydrase (EC 4.2.1.1)	FIG00023019	icw(1);Cyanate_hydrolysis
NODE_2_length_244240_cov_42.654095	fig|6666666.34159.peg.314	peg	NODE_2_length_244240_cov_42.654095_119842_119955	119842	1E+05	+	hypothetical protein		
NODE_2_length_244240_cov_42.654095	fig|6666666.34159.peg.315	peg	NODE_2_length_244240_cov_42.654095_119939_120055	119939	1E+05	+	hypothetical protein		
NODE_2_length_244240_cov_42.654095	fig|6666666.34159.peg.316	peg	NODE_2_length_244240_cov_42.654095_120247_122856	120247	1E+05	+	Hypothetical protein		
NODE_2_length_244240_cov_42.654095	fig|6666666.34159.peg.317	peg	NODE_2_length_244240_cov_42.654095_122901_123308	122901	1E+05	+	Putative superfamily I DNA helicases	FIG01315518	ff
NODE_2_length_244240_cov_42.654095	fig|6666666.34159.peg.318	peg	NODE_2_length_244240_cov_42.654095_123326_123457	123326	1E+05	+	hypothetical protein		
NODE_2_length_244240_cov_42.654095	fig|6666666.34159.peg.319	peg	NODE_2_length_244240_cov_42.654095_123520_124554	123520	1E+05	+	Chalcone synthase (EC 2.3.1.74)	FIG00443156	isu;Flavanone_biosynthesis
NODE_2_length_244240_cov_42.654095	fig|6666666.34159.peg.320	peg	NODE_2_length_244240_cov_42.654095_124577_125230	124577	1E+05	+	hypothetical protein	FIG00638284	ff
NODE_2_length_244240_cov_42.654095	fig|6666666.34159.peg.321	peg	NODE_2_length_244240_cov_42.654095_125227_126255	125227	1E+05	+	hypothetical protein		
NODE_2_length_244240_cov_42.654095	fig|6666666.34159.peg.322	peg	NODE_2_length_244240_cov_42.654095_128286_126259	128286	1E+05	-	TPR repeat		
NODE_2_length_244240_cov_42.654095	fig|6666666.34159.peg.323	peg	NODE_2_length_244240_cov_42.654095_128384_129277	128384	1E+05	+	Aminoglycoside phosphotransferase	FIG01358230	ff
NODE_2_length_244240_cov_42.654095	fig|6666666.34159.peg.324	peg	NODE_2_length_244240_cov_42.654095_129362_130303	129362	1E+05	+	hypothetical protein		
NODE_2_length_244240_cov_42.654095	fig|6666666.34159.peg.325	peg	NODE_2_length_244240_cov_42.654095_130353_131804	130353	1E+05	+	protein containing metallophosphoesterase domain(EC:3.1.-)		
NODE_2_length_244240_cov_42.654095	fig|6666666.34159.peg.326	peg	NODE_2_length_244240_cov_42.654095_131986_131825	131986	1E+05	-	hypothetical protein		
NODE_2_length_244240_cov_42.654095	fig|6666666.34159.peg.327	peg	NODE_2_length_244240_cov_42.654095_132406_133584	132406	1E+05	+	hypothetical protein		
NODE_2_length_244240_cov_42.654095	fig|6666666.34159.peg.328	peg	NODE_2_length_244240_cov_42.654095_134927_133710	134927	1E+05	-	hypothetical protein		
NODE_2_length_244240_cov_42.654095	fig|6666666.34159.peg.329	peg	NODE_2_length_244240_cov_42.654095_135151_135738	135151	1E+05	+	hypothetical protein	FIG00638284	ff
NODE_2_length_244240_cov_42.654095	fig|6666666.34159.peg.330	peg	NODE_2_length_244240_cov_42.654095_136954_135944	136954	1E+05	-	hypothetical protein	FIG00638284	ff
NODE_2_length_244240_cov_42.654095	fig|6666666.34159.peg.331	peg	NODE_2_length_244240_cov_42.654095_137559_136951	137559	1E+05	-	hypothetical protein	FIG00638284	ff
NODE_2_length_244240_cov_42.654095	fig|6666666.34159.peg.332	peg	NODE_2_length_244240_cov_42.654095_137796_137659	137796	1E+05	-	hypothetical protein		
NODE_2_length_244240_cov_42.654095	fig|6666666.34159.peg.333	peg	NODE_2_length_244240_cov_42.654095_138486_138373	138486	1E+05	-	hypothetical protein		
NODE_2_length_244240_cov_42.654095	fig|6666666.34159.peg.334	peg	NODE_2_length_244240_cov_42.654095_139891_138593	139891	1E+05	-	hypothetical protein		
NODE_2_length_244240_cov_42.654095	fig|6666666.34159.peg.335	peg	NODE_2_length_244240_cov_42.654095_140530_140390	140530	1E+05	-	hypothetical protein		
NODE_2_length_244240_cov_42.654095	fig|6666666.34159.peg.336	peg	NODE_2_length_244240_cov_42.654095_141690_140623	141690	1E+05	-	Phosphoenolpyruvate carboxykinase [ATP] (EC 4.1.1.49)	FIG00000834	isu;Pyruvate_metabolism_I:_anaplerotic_reactions,_PEP
NODE_2_length_244240_cov_42.654095	fig|6666666.34159.peg.337	peg	NODE_2_length_244240_cov_42.654095_141919_151050	141919	2E+05	+	hypothetical protein	FIG00638284	ff
NODE_2_length_244240_cov_42.654095	fig|6666666.34159.peg.338	peg	NODE_2_length_244240_cov_42.654095_151053_152243	151053	2E+05	+	hypothetical protein	FIG00638284	ff
NODE_2_length_244240_cov_42.654095	fig|6666666.34159.peg.339	peg	NODE_2_length_244240_cov_42.654095_152268_154643	152268	2E+05	+	hypothetical protein	FIG00638284	ff
NODE_2_length_244240_cov_42.654095	fig|6666666.34159.peg.340	peg	NODE_2_length_244240_cov_42.654095_154768_157071	154768	2E+05	+	hypothetical protein	FIG00638284	ff
NODE_2_length_244240_cov_42.654095	fig|6666666.34159.peg.341	peg	NODE_2_length_244240_cov_42.654095_158408_157170	158408	2E+05	-	amino acid permease		
NODE_2_length_244240_cov_42.654095	fig|6666666.34159.peg.342	peg	NODE_2_length_244240_cov_42.654095_159204_158458	159204	2E+05	-	Glutamine amidotransferase, class I	FIG01310956	ff
NODE_2_length_244240_cov_42.654095	fig|6666666.34159.peg.343	peg	NODE_2_length_244240_cov_42.654095_159446_160042	159446	2E+05	+	hypothetical protein		
NODE_2_length_244240_cov_42.654095	fig|6666666.34159.peg.344	peg	NODE_2_length_244240_cov_42.654095_160522_169674	160522	2E+05	+	hypothetical protein	FIG00638284	ff
NODE_2_length_244240_cov_42.654095	fig|6666666.34159.peg.345	peg	NODE_2_length_244240_cov_42.654095_170440_169724	170440	2E+05	-	DNA repair protein RadC	FIG00037431	isu;DNA_repair,_bacterial
NODE_2_length_244240_cov_42.654095	fig|6666666.34159.peg.346	peg	NODE_2_length_244240_cov_42.654095_171747_170437	171747	2E+05	-	GTP-binding protein HflX	FIG00000304	isu;Hfl_operon
NODE_2_length_244240_cov_42.654095	fig|6666666.34159.peg.347	peg	NODE_2_length_244240_cov_42.654095_171815_172585	171815	2E+05	+	hypothetical protein		
NODE_2_length_244240_cov_42.654095	fig|6666666.34159.peg.348	peg	NODE_2_length_244240_cov_42.654095_173337_172543	173337	2E+05	-	Metal-dependent hydrolases of the beta-lactamase superfamily I; PhnP protein	FIG01955808	isu;Beta-lactamase
NODE_2_length_244240_cov_42.654095	fig|6666666.34159.peg.349	peg	NODE_2_length_244240_cov_42.654095_173915_173334	173915	2E+05	-	hypothetical protein		

NODE_2_length_244240_cov_42.654095	fig|6666666.34159.peg.350	peg	NODE_2_length_244240_cov_42.654095_174326_174919	174326	2E+05	+	YheO-like PAS domain		
NODE_2_length_244240_cov_42.654095	fig|6666666.34159.peg.351	peg	NODE_2_length_244240_cov_42.654095_174921_175829	174921	2E+05	+	Aspartate aminotransferase (EC 2.6.1.1)	FIG00719736	idu(1);Coenzyme_M_biosynthesis_--_gjo idu(1);Glutamine,_Glutamate,_Aspartate_and_As
NODE_2_length_244240_cov_42.654095	fig|6666666.34159.peg.352	peg	NODE_2_length_244240_cov_42.654095_175833_176042	175833	2E+05	+	aminotransferase, classes I and II		
NODE_2_length_244240_cov_42.654095	fig|6666666.34159.peg.353	peg	NODE_2_length_244240_cov_42.654095_176045_176929	176045	2E+05	+	COG0384: Predicted epimerase, PhzC/PhzF homolog		
NODE_2_length_244240_cov_42.654095	fig|6666666.34159.peg.354	peg	NODE_2_length_244240_cov_42.654095_176926_177867	176926	2E+05	+	Ornithine cyclodeaminase (EC 4.3.1.12)	FIG00000614	ff
NODE_2_length_244240_cov_42.654095	fig|6666666.34159.peg.355	peg	NODE_2_length_244240_cov_42.654095_178000_178134	178000	2E+05	+	hypothetical protein		
NODE_2_length_244240_cov_42.654095	fig|6666666.34159.peg.356	peg	NODE_2_length_244240_cov_42.654095_178367_178167	178367	2E+05	-	hypothetical protein		
NODE_2_length_244240_cov_42.654095	fig|6666666.34159.peg.357	peg	NODE_2_length_244240_cov_42.654095_178446_180728	178446	2E+05	+	hypothetical protein	FIG00638284	ff
NODE_2_length_244240_cov_42.654095	fig|6666666.34159.peg.358	peg	NODE_2_length_244240_cov_42.654095_180721_181287	180721	2E+05	+	hypothetical protein		
NODE_2_length_244240_cov_42.654095	fig|6666666.34159.peg.359	peg	NODE_2_length_244240_cov_42.654095_182100_181297	182100	2E+05	-	hypothetical protein		
NODE_2_length_244240_cov_42.654095	fig|6666666.34159.peg.360	peg	NODE_2_length_244240_cov_42.654095_182473_182772	182473	2E+05	+	hypothetical protein		
NODE_2_length_244240_cov_42.654095	fig|6666666.34159.peg.361	peg	NODE_2_length_244240_cov_42.654095_183220_182948	183220	2E+05	-	hypothetical protein	FIG00638284	ff
NODE_2_length_244240_cov_42.654095	fig|6666666.34159.peg.362	peg	NODE_2_length_244240_cov_42.654095_184856_183252	184856	2E+05	-	hypothetical protein		
NODE_2_length_244240_cov_42.654095	fig|6666666.34159.peg.363	peg	NODE_2_length_244240_cov_42.654095_186076_185039	186076	2E+05	-	putative DNA polymerase III, delta' subunit, holB		
NODE_2_length_244240_cov_42.654095	fig|6666666.34159.peg.364	peg	NODE_2_length_244240_cov_42.654095_186819_186139	186819	2E+05	-	Thymidylate kinase (EC 2.7.4.9)	FIG00000175	idu(1);pyrimidine_conversions idu(1);CBSS-393133.3.peg.2787
NODE_2_length_244240_cov_42.654095	fig|6666666.34159.peg.365	peg	NODE_2_length_244240_cov_42.654095_189457_186860	189457	2E+05	-	DNA gyrase subunit A (EC 5.99.1.3)	FIG00000080	icw(1);DNA_gyrase_subunits icw(1);DNA_topoisomerases,_Type_II,_ATP-
NODE_2_length_244240_cov_42.654095	fig|6666666.34159.peg.366	peg	NODE_2_length_244240_cov_42.654095_191985_189484	191985	2E+05	-	DNA gyrase subunit B (EC 5.99.1.3)	FIG00028203	isu;DNA_gyrase_subunits isu;DNA_topoisomerases,_Type_II,_ATP-
NODE_2_length_244240_cov_42.654095	fig|6666666.34159.peg.367	peg	NODE_2_length_244240_cov_42.654095_192368_192030	192368	2E+05	-	hypothetical protein		
NODE_2_length_244240_cov_42.654095	fig|6666666.34159.peg.368	peg	NODE_2_length_244240_cov_42.654095_193418_192423	193418	2E+05	-	hypothetical protein	FIG00638284	ff
NODE_2_length_244240_cov_42.654095	fig|6666666.34159.peg.369	peg	NODE_2_length_244240_cov_42.654095_193599_195689	193599	2E+05	+	O-linked GlcNAc transferase	FIG01477005	ff
NODE_2_length_244240_cov_42.654095	fig|6666666.34159.peg.370	peg	NODE_2_length_244240_cov_42.654095_195705_197711	195705	2E+05	+	DNA ligase (EC 6.5.1.2)	FIG00051439	isu;DNA_Repair_Base_Excision isu;CBSS-393121.3.peg.1913
NODE_2_length_244240_cov_42.654095	fig|6666666.34159.peg.371	peg	NODE_2_length_244240_cov_42.654095_197826_197713	197826	2E+05	-	hypothetical protein		
NODE_2_length_244240_cov_42.654095	fig|6666666.34159.peg.372	peg	NODE_2_length_244240_cov_42.654095_197778_198314	197778	2E+05	+	Superoxide dismutase [Cu-Zn] precursor (EC 1.15.1.1)	FIG00001181	isu;Oxidative_stress isu;Protection_from_Reactive_Oxygen_Species
NODE_2_length_244240_cov_42.654095	fig|6666666.34159.peg.373	peg	NODE_2_length_244240_cov_42.654095_199013_198318	199013	2E+05	-	Queuosine Biosynthesis QueC ATPase	FIG00000491	idu(1);tRNA_modification_Bacteria idu(1);Queuosine-Archaeosine_Biosynthesis
NODE_2_length_244240_cov_42.654095	fig|6666666.34159.peg.374	peg	NODE_2_length_244240_cov_42.654095_200294_199119	200294	2E+05	-	Branched-chain amino acid transport system carrier protein	FIG00905542	ff
NODE_2_length_244240_cov_42.654095	fig|6666666.34159.peg.375	peg	NODE_2_length_244240_cov_42.654095_200644_201174	200644	2E+05	+	hypothetical protein		
NODE_2_length_244240_cov_42.654095	fig|6666666.34159.peg.376	peg	NODE_2_length_244240_cov_42.654095_201703_201239	201703	2E+05	-	hypothetical protein		
NODE_2_length_244240_cov_42.654095	fig|6666666.34159.peg.377	peg	NODE_2_length_244240_cov_42.654095_202343_201795	202343	2E+05	-	ADP-ribose pyrophosphatase (EC 3.6.1.13)	FIG00000367	isu;NAD_and_NADP_cofactor_biosynthesis_global isu;CBSS-216591.1.peg.168
NODE_2_length_244240_cov_42.654095	fig|6666666.34159.peg.378	peg	NODE_2_length_244240_cov_42.654095_203410_202430	203410	2E+05	-	hypothetical protein		
NODE_2_length_244240_cov_42.654095	fig|6666666.34159.peg.379	peg	NODE_2_length_244240_cov_42.654095_203637_204023	203637	2E+05	+	COG2363	FIG00018398	ff
NODE_2_length_244240_cov_42.654095	fig|6666666.34159.peg.380	peg	NODE_2_length_244240_cov_42.654095_204020_205189	204020	2E+05	+	LSU m5C1962 methyltransferase RlmI	FIG00004612	isu;CBSS-83333.1.peg.946 isu;RNA_methylation isu;CBSS-326442.4.peg.1852
NODE_2_length_244240_cov_42.654095	fig|6666666.34159.peg.381	peg	NODE_2_length_244240_cov_42.654095_208325_205494	208325	2E+05	-	hypothetical protein	FIG00638284	ff
NODE_2_length_244240_cov_42.654095	fig|6666666.34159.peg.382	peg	NODE_2_length_244240_cov_42.654095_209652_208423	209652	2E+05	-	hypothetical protein	FIG00638284	ff
NODE_2_length_244240_cov_42.654095	fig|6666666.34159.peg.383	peg	NODE_2_length_244240_cov_42.654095_210735_209656	210735	2E+05	-	Diaminohydroxyphosphoribosylaminopyrimidine deaminase (EC 3.5.4.26) / 5-amino-6-(5-phosphoribosylamino)uracil	FIG00000384	isu;Riboflavin_synthesis_cluster isu;Riboflavin_synthesis_cluster
NODE_2_length_244240_cov_42.654095	fig|6666666.34159.peg.384	peg	NODE_2_length_244240_cov_42.654095_212745_210760	212745	2E+05	-	Acetyl-coenzyme A synthetase (EC 6.2.1.1)	FIG00022631	isu;Pyruvate_metabolism_II:_acetyl-CoA,_acetogenesis_from_pyruvate
NODE_2_length_244240_cov_42.654095	fig|6666666.34159.peg.385	peg	NODE_2_length_244240_cov_42.654095_212744_212857	212744	2E+05	+	hypothetical protein		
NODE_2_length_244240_cov_42.654095	fig|6666666.34159.peg.386	peg	NODE_2_length_244240_cov_42.654095_212991_213512	212991	2E+05	+	Peptidyl-prolyl cis-trans isomerase PpiB (EC 5.2.1.8)		isu;Queuosine-Archaeosine_Biosynthesis isu;Peptidyl-prolyl_cis-trans_isomerase
NODE_2_length_244240_cov_42.654095	fig|6666666.34159.peg.387	peg	NODE_2_length_244240_cov_42.654095_213525_214433	213525	2E+05	+	hypothetical protein	FIG00638284	ff
NODE_2_length_244240_cov_42.654095	fig|6666666.34159.peg.388	peg	NODE_2_length_244240_cov_42.654095_214442_215431	214442	2E+05	+	hypothetical protein		
NODE_2_length_244240_cov_42.654095	fig|6666666.34159.peg.389	peg	NODE_2_length_244240_cov_42.654095_215477_216112	215477	2E+05	+	hypothetical protein		
NODE_2_length_244240_cov_42.654095	fig|6666666.34159.peg.390	peg	NODE_2_length_244240_cov_42.654095_216225_216109	216225	2E+05	-	hypothetical protein		
NODE_2_length_244240_cov_42.654095	fig|6666666.34159.peg.391	peg	NODE_2_length_244240_cov_42.654095_216359_216517	216359	2E+05	+	hypothetical protein		
NODE_2_length_244240_cov_42.654095	fig|6666666.34159.peg.392	peg	NODE_2_length_244240_cov_42.654095_216749_218989	216749	2E+05	+	hypothetical protein		
NODE_2_length_244240_cov_42.654095	fig|6666666.34159.peg.393	peg	NODE_2_length_244240_cov_42.654095_219856_219032	219856	2E+05	-	hypothetical protein	FIG00638284	ff
NODE_2_length_244240_cov_42.654095	fig|6666666.34159.peg.394	peg	NODE_2_length_244240_cov_42.654095_221051_219888	221051	2E+05	-	Citrate synthase (si) (EC 2.3.3.1)	FIG00130344	icw(1);TCA_Cycle icw(1);Glyoxylate_bypass
NODE_2_length_244240_cov_42.654095	fig|6666666.34159.peg.395	peg	NODE_2_length_244240_cov_42.654095_222066_221077	222066	2E+05	-	Malate dehydrogenase (EC 1.1.1.37)	FIG00000455	isu;TCA_Cycle isu;Glyoxylate_bypass
NODE_2_length_244240_cov_42.654095	fig|6666666.34159.peg.396	peg	NODE_2_length_244240_cov_42.654095_222318_222464	222318	2E+05	+	hypothetical protein		
NODE_2_length_244240_cov_42.654095	fig|6666666.34159.peg.397	peg	NODE_2_length_244240_cov_42.654095_222600_222472	222600	2E+05	-	hypothetical protein		
NODE_2_length_244240_cov_42.654095	fig|6666666.34159.peg.398	peg	NODE_2_length_244240_cov_42.654095_223325_223999	223325	2E+05	+	Queuosine Biosynthesis QueE Radical SAM	FIG00000490	icw(1);tRNA_modification_Bacteria isu;Queuosine-Archaeosine_Biosynthesis
NODE_2_length_244240_cov_42.654095	fig|6666666.34159.peg.399	peg	NODE_2_length_244240_cov_42.654095_224001_224666	224001	2E+05	+	Queuosine Biosynthesis QueC ATPase	FIG00000491	idu(1);tRNA_modification_Bacteria icw(1);Queuosine-Archaeosine_Biosynthesis
NODE_2_length_244240_cov_42.654095	fig|6666666.34159.peg.400	peg	NODE_2_length_244240_cov_42.654095_225927_224659	225927	2E+05	-	hypothetical protein		
NODE_2_length_244240_cov_42.654095	fig|6666666.34159.peg.401	peg	NODE_2_length_244240_cov_42.654095_230454_226366	230454	2E+05	-	hypothetical protein	FIG00638284	ff
NODE_2_length_244240_cov_42.654095	fig|6666666.34159.peg.402	peg	NODE_2_length_244240_cov_42.654095_230672_232750	230672	2E+05	+	Thymidylate kinase (EC 2.7.4.9)	FIG00000175	idu(1);pyrimidine_conversions idu(1);CBSS-393133.3.peg.2787
NODE_2_length_244240_cov_42.654095	fig|6666666.34159.peg.403	peg	NODE_2_length_244240_cov_42.654095_232957_233760	232957	2E+05	+	hypothetical protein		
NODE_2_length_244240_cov_42.654095	fig|6666666.34159.peg.404	peg	NODE_2_length_244240_cov_42.654095_235177_234056	235177	2E+05	-	hypothetical protein		
NODE_2_length_244240_cov_42.654095	fig|6666666.34159.peg.405	peg	NODE_2_length_244240_cov_42.654095_235911_236771	235911	2E+05	+	hypothetical protein		
NODE_2_length_244240_cov_42.654095	fig|6666666.34159.peg.406	peg	NODE_2_length_244240_cov_42.654095_239104_236951	239104	2E+05	-	Potassium efflux system KefA protein / Small-conductance mechanosensitive channel	FIG00021330	isu;Potassium_homeostasis
NODE_2_length_244240_cov_42.654095	fig|6666666.34159.peg.407	peg	NODE_2_length_244240_cov_42.654095_239241_241496	239241	2E+05	+	Transcription accessory protein (S1 RNA-binding domain)	FIG00001712	isu;CBSS-243265.1.peg.198 isu;Cell_division-ribosomal_stress_proteins_cluster
NODE_2_length_244240_cov_42.654095	fig|6666666.34159.peg.408	peg	NODE_2_length_244240_cov_42.654095_241493_242779	241493	2E+05	+	3-deoxy-D-manno-octulosonic-acid transferase (EC 2.-.-.-)	FIG00000616	isu;LOS_core_oligosaccharide_biosynthesis isu;KDO2-Lipid_A_biosynthesis

NODE_2_length_244240_cov_42.654095	fig|6666666.34159.rna.5	rna	NODE_2_length_244240_cov_42.654095_242881_242953	242881	2E+05	+	tRNA-Met-CAT		
NODE_2_length_244240_cov_42.654095	fig|6666666.34159.rna.6	rna	NODE_2_length_244240_cov_42.654095_242960_243032	242960	2E+05	+	tRNA-Met-CAT		
NODE_2_length_244240_cov_42.654095	fig|6666666.34159.rna.7	rna	NODE_2_length_244240_cov_42.654095_243095_243167	243095	2E+05	+	tRNA-Met-CAT		
NODE_2_length_244240_cov_42.654095	fig|6666666.34159.peg.409	peg	NODE_2_length_244240_cov_42.654095_243892_243200	243892	2E+05	-	hypothetical protein		
NODE_30_length_529_cov_40.372414	fig|6666666.34159.rna.8	rna	NODE_30_length_529_cov_40.372414_311_238	311	238	-	tRNA-Ile-GAT		
NODE_30_length_529_cov_40.372414	fig|6666666.34159.rna.9	rna	NODE_30_length_529_cov_40.372414_392_320	392	320	-	tRNA-Ala-TGC		
NODE_31_length_149586_cov_42.053596	fig|6666666.34159.peg.410	peg	NODE_31_length_149586_cov_42.053596_47_412	47	412	+	hypothetical protein		
NODE_31_length_149586_cov_42.053596	fig|6666666.34159.peg.411	peg	NODE_31_length_149586_cov_42.053596_1866_574	1866	574	-	hypothetical protein		
NODE_31_length_149586_cov_42.053596	fig|6666666.34159.peg.412	peg	NODE_31_length_149586_cov_42.053596_2930_1866	2930	1866	-	hypothetical protein		
NODE_31_length_149586_cov_42.053596	fig|6666666.34159.peg.413	peg	NODE_31_length_149586_cov_42.053596_3186_3821	3186	3821	+	V-type ATP synthase subunit E (EC 3.6.3.14)		icw(4);V-Type_ATP_synthase
NODE_31_length_149586_cov_42.053596	fig|6666666.34159.peg.414	peg	NODE_31_length_149586_cov_42.053596_4161_3952	4161	3952	-	hypothetical protein		
NODE_31_length_149586_cov_42.053596	fig|6666666.34159.peg.415	peg	NODE_31_length_149586_cov_42.053596_4258_4136	4258	4136	-	hypothetical protein		
NODE_31_length_149586_cov_42.053596	fig|6666666.34159.peg.416	peg	NODE_31_length_149586_cov_42.053596_4561_4683	4561	4683	+	V-type ATP synthase subunit C (EC 3.6.3.14)	FIG00002560	icw(3);V-Type_ATP_synthase
NODE_31_length_149586_cov_42.053596	fig|6666666.34159.peg.417	peg	NODE_31_length_149586_cov_42.053596_4680_6467	4680	6467	+	V-type ATP synthase subunit A (EC 3.6.3.14)	FIG00001902	icw(4);V-Type_ATP_synthase
NODE_31_length_149586_cov_42.053596	fig|6666666.34159.peg.418	peg	NODE_31_length_149586_cov_42.053596_6483_7799	6483	7799	+	V-type ATP synthase subunit B (EC 3.6.3.14)	FIG00134167	icw(1);V-Type_ATP_synthase
NODE_31_length_149586_cov_42.053596	fig|6666666.34159.peg.419	peg	NODE_31_length_149586_cov_42.053596_7809_8459	7809	8459	+	V-type ATP synthase subunit D (EC 3.6.3.14)	FIG00002460	icw(2);V-Type_ATP_synthase
NODE_31_length_149586_cov_42.053596	fig|6666666.34159.peg.420	peg	NODE_31_length_149586_cov_42.053596_8449_10368	8449	10368	+	V-type ATP synthase subunit I (EC 3.6.3.14)	FIG00140555	isu;V-Type_ATP_synthase
NODE_31_length_149586_cov_42.053596	fig|6666666.34159.peg.421	peg	NODE_31_length_149586_cov_42.053596_10387_10815	10387	10815	+	V-type ATP synthase subunit K (EC 3.6.3.14)	FIG00003998	icw(3);V-Type_ATP_synthase
NODE_31_length_149586_cov_42.053596	fig|6666666.34159.peg.422	peg	NODE_31_length_149586_cov_42.053596_11246_10869	11246	10869	-	hypothetical protein		
NODE_31_length_149586_cov_42.053596	fig|6666666.34159.peg.423	peg	NODE_31_length_149586_cov_42.053596_11482_12756	11482	12756	+	hypothetical protein		
NODE_31_length_149586_cov_42.053596	fig|6666666.34159.peg.424	peg	NODE_31_length_149586_cov_42.053596_12947_12789	12947	12789	-	hypothetical protein		
NODE_31_length_149586_cov_42.053596	fig|6666666.34159.peg.425	peg	NODE_31_length_149586_cov_42.053596_13021_13206	13021	13206	+	hypothetical protein		
NODE_31_length_149586_cov_42.053596	fig|6666666.34159.peg.426	peg	NODE_31_length_149586_cov_42.053596_13457_13227	13457	13227	-	hypothetical protein		
NODE_31_length_149586_cov_42.053596	fig|6666666.34159.peg.427	peg	NODE_31_length_149586_cov_42.053596_13692_13579	13692	13579	-	hypothetical protein		
NODE_31_length_149586_cov_42.053596	fig|6666666.34159.peg.428	peg	NODE_31_length_149586_cov_42.053596_14768_13692	14768	13692	-	2-keto-3-deoxy-D-arabino-heptulosonate-7-phosphate synthase I alpha (EC 2.5.1.54)	FIG00002110	isu;Common_Pathway_For_Synthesis_of_Aromatic_Compounds_(DAHP_synthase_to_chorismate)
NODE_31_length_149586_cov_42.053596	fig|6666666.34159.peg.429	peg	NODE_31_length_149586_cov_42.053596_14861_16483	14861	16483	+	hypothetical protein		
NODE_31_length_149586_cov_42.053596	fig|6666666.34159.peg.430	peg	NODE_31_length_149586_cov_42.053596_16608_16754	16608	16754	+	hypothetical protein		
NODE_31_length_149586_cov_42.053596	fig|6666666.34159.peg.431	peg	NODE_31_length_149586_cov_42.053596_16782_18164	16782	18164	+	Sodium/glycine symporter GlyP	FIG00007058	icw(1);Glycine_cleavage_system
NODE_31_length_149586_cov_42.053596	fig|6666666.34159.peg.432	peg	NODE_31_length_149586_cov_42.053596_18185_19570	18185	19570	+	Sodium/glycine symporter GlyP	FIG00007058	icw(1);Glycine_cleavage_system
NODE_31_length_149586_cov_42.053596	fig|6666666.34159.peg.433	peg	NODE_31_length_149586_cov_42.053596_22499_19626	22499	19626	-	Valyl-tRNA synthetase (EC 6.1.1.9)	FIG00000129	isu;tRNA_aminoacylation,_Val
NODE_31_length_149586_cov_42.053596	fig|6666666.34159.peg.434	peg	NODE_31_length_149586_cov_42.053596_24282_22582	24282	22582	-	hypothetical protein	FIG00638284	ff
NODE_31_length_149586_cov_42.053596	fig|6666666.34159.peg.435	peg	NODE_31_length_149586_cov_42.053596_26090_24312	26090	24312	-	hypothetical protein	FIG00638284	ff
NODE_31_length_149586_cov_42.053596	fig|6666666.34159.peg.436	peg	NODE_31_length_149586_cov_42.053596_26291_28165	26291	28165	+	hypothetical protein	FIG00638284	ff
NODE_31_length_149586_cov_42.053596	fig|6666666.34159.peg.437	peg	NODE_31_length_149586_cov_42.053596_28296_29234	28296	29234	+	hypothetical protein		
NODE_31_length_149586_cov_42.053596	fig|6666666.34159.peg.438	peg	NODE_31_length_149586_cov_42.053596_30407_29310	30407	29310	-	Ribosomal RNA small subunit methyltransferase B (EC 2.1.1.-)	FIG00138175	ff
NODE_31_length_149586_cov_42.053596	fig|6666666.34159.peg.439	peg	NODE_31_length_149586_cov_42.053596_30629_31066	30629	31066	+	hypothetical protein		
NODE_31_length_149586_cov_42.053596	fig|6666666.34159.peg.440	peg	NODE_31_length_149586_cov_42.053596_31099_32832	31099	32832	+	Apolipoprotein N-acyltransferase (EC 2.3.1.-)	FIG01303912	isu;Lipoprotein_Biosynthesis
NODE_31_length_149586_cov_42.053596	fig|6666666.34159.peg.441	peg	NODE_31_length_149586_cov_42.053596_32969_33853	32969	33853	+	UDP-3-O-[3-hydroxymyristoyl] N-acetylglucosamine deacetylase (EC 3.5.1.-)	FIG00000615	icw(1);KDO2-Lipid_A_biosynthesis
NODE_31_length_149586_cov_42.053596	fig|6666666.34159.peg.442	peg	NODE_31_length_149586_cov_42.053596_33850_34323	33850	34323	+	(3R)-hydroxymyristoyl-[acyl carrier protein] dehydratase (EC 4.2.1.-)	FIG00000393	isu;Fatty_Acid_Biosynthesis_FASII icw(1);Llipid_A_biosynthesis_cluster
NODE_31_length_149586_cov_42.053596	fig|6666666.34159.peg.443	peg	NODE_31_length_149586_cov_42.053596_34374_35228	34374	35228	+	Acyl-[acyl-carrier-protein]--UDP-N-acetylglucosamine O-acyltransferase (EC 2.3.1.129)	FIG00000508	isu;Llipid_A_biosynthesis_cluster isu;KDO2-Lipid_A_biosynthesis
NODE_31_length_149586_cov_42.053596	fig|6666666.34159.peg.444	peg	NODE_31_length_149586_cov_42.053596_35333_36283	35333	36283	+	Methionyl-tRNA formyltransferase (EC 2.1.2.9)	FIG00000112	isu;CBSS-89187.3.peg.2957 isu;Translation_initiation_factors_bacterial
NODE_31_length_149586_cov_42.053596	fig|6666666.34159.peg.445	peg	NODE_31_length_149586_cov_42.053596_36283_37005	36283	37005	+	hypothetical protein		
NODE_31_length_149586_cov_42.053596	fig|6666666.34159.peg.446	peg	NODE_31_length_149586_cov_42.053596_37340_38542	37340	38542	+	hypothetical protein		
NODE_31_length_149586_cov_42.053596	fig|6666666.34159.peg.447	peg	NODE_31_length_149586_cov_42.053596_38602_38895	38602	38895	+	hypothetical protein		
NODE_31_length_149586_cov_42.053596	fig|6666666.34159.peg.448	peg	NODE_31_length_149586_cov_42.053596_39351_40013	39351	40013	+	LSU ribosomal protein L3p (L3e)	FIG00001818	ff
NODE_31_length_149586_cov_42.053596	fig|6666666.34159.peg.449	peg	NODE_31_length_149586_cov_42.053596_40034_40717	40034	40717	+	LSU ribosomal protein L4p (L1e)	FIG00000263	ff
NODE_31_length_149586_cov_42.053596	fig|6666666.34159.peg.450	peg	NODE_31_length_149586_cov_42.053596_40751_41086	40751	41086	+	LSU ribosomal protein L23p (L23Ae)	FIG00000271	ff
NODE_31_length_149586_cov_42.053596	fig|6666666.34159.peg.451	peg	NODE_31_length_149586_cov_42.053596_41146_41991	41146	41991	+	LSU ribosomal protein L2p (L8e)	FIG00000223	ff
NODE_31_length_149586_cov_42.053596	fig|6666666.34159.peg.452	peg	NODE_31_length_149586_cov_42.053596_41994_42257	41994	42257	+	SSU ribosomal protein S19p (S15e)	FIG00000199	ff
NODE_31_length_149586_cov_42.053596	fig|6666666.34159.peg.453	peg	NODE_31_length_149586_cov_42.053596_42615_43265	42615	43265	+	SSU ribosomal protein S3p (S3e)	FIG00000218	ff
NODE_31_length_149586_cov_42.053596	fig|6666666.34159.peg.454	peg	NODE_31_length_149586_cov_42.053596_43280_43699	43280	43699	+	LSU ribosomal protein L16p (L10e)	FIG00001132	ff
NODE_31_length_149586_cov_42.053596	fig|6666666.34159.peg.455	peg	NODE_31_length_149586_cov_42.053596_43710_43925	43710	43925	+	hypothetical protein		
NODE_31_length_149586_cov_42.053596	fig|6666666.34159.peg.456	peg	NODE_31_length_149586_cov_42.053596_43935_44177	43935	44177	+	SSU ribosomal protein S17p (S11e)	FIG00000181	ff
NODE_31_length_149586_cov_42.053596	fig|6666666.34159.peg.457	peg	NODE_31_length_149586_cov_42.053596_44291_44656	44291	44656	+	LSU ribosomal protein L14p (L23e)	FIG00090781	ff
NODE_31_length_149586_cov_42.053596	fig|6666666.34159.peg.458	peg	NODE_31_length_149586_cov_42.053596_44659_45033	44659	45033	+	LSU ribosomal protein L24p (L26e)	FIG00000198	ff
NODE_31_length_149586_cov_42.053596	fig|6666666.34159.peg.459	peg	NODE_31_length_149586_cov_42.053596_45036_45593	45036	45593	+	LSU ribosomal protein L5p (L11e)	FIG00000174	ff
NODE_31_length_149586_cov_42.053596	fig|6666666.34159.peg.460	peg	NODE_31_length_149586_cov_42.053596_45611_46012	45611	46012	+	SSU ribosomal protein S8p (S15Ae)	FIG00000153	ff
NODE_31_length_149586_cov_42.053596	fig|6666666.34159.peg.461	peg	NODE_31_length_149586_cov_42.053596_46077_46622	46077	46622	+	LSU ribosomal protein L6p (L9e)	FIG00001577	ff
NODE_31_length_149586_cov_42.053596	fig|6666666.34159.peg.462	peg	NODE_31_length_149586_cov_42.053596_46686_47054	46686	47054	+	LSU ribosomal protein L18p (L5e)	FIG00000200	ff

NODE_31_length_149586_cov_42.053596	fig|6666666.34159.peg.463	peg	NODE_31_length_149586_cov_42.053596_47095_47595	47095	47595	+	SSU ribosomal protein S5p (S2e)	FIG00000155	isu;Ribosomal_protein_S5p_acylation
NODE_31_length_149586_cov_42.053596	fig|6666666.34159.peg.464	peg	NODE_31_length_149586_cov_42.053596_47592_48047	47592	48047	+	LSU ribosomal protein L15p (L27Ae)	FIG00000166	ff
NODE_31_length_149586_cov_42.053596	fig|6666666.34159.peg.465	peg	NODE_31_length_149586_cov_42.053596_48178_49548	48178	49548	+	Preprotein translocase secY subunit (TC 3.A.5.1.1)	FIG00937056	ff
NODE_31_length_149586_cov_42.053596	fig|6666666.34159.peg.466	peg	NODE_31_length_149586_cov_42.053596_49627_49995	49627	49995	+	SSU ribosomal protein S13p (S18e)	FIG00000160	ff
NODE_31_length_149586_cov_42.053596	fig|6666666.34159.peg.467	peg	NODE_31_length_149586_cov_42.053596_50013_50423	50013	50423	+	SSU ribosomal protein S11p (S14e)	FIG00055673	ff
NODE_31_length_149586_cov_42.053596	fig|6666666.34159.peg.468	peg	NODE_31_length_149586_cov_42.053596_50553_51668	50553	51668	+	DNA-directed RNA polymerase alpha subunit (EC 2.7.7.6)	FIG00000140	isu;RNA_polymerase_bacterial
NODE_31_length_149586_cov_42.053596	fig|6666666.34159.peg.469	peg	NODE_31_length_149586_cov_42.053596_51749_52108	51749	52108	+	LSU ribosomal protein L17p	FIG00000154	ff
NODE_31_length_149586_cov_42.053596	fig|6666666.34159.peg.470	peg	NODE_31_length_149586_cov_42.053596_54225_52171	54225	52171	-	methyl-accepting chemotaxis sensory transducer		
NODE_31_length_149586_cov_42.053596	fig|6666666.34159.peg.471	peg	NODE_31_length_149586_cov_42.053596_54382_55323	54382	55323	+	unknown protein	FIG00764722	ff
NODE_31_length_149586_cov_42.053596	fig|6666666.34159.peg.472	peg	NODE_31_length_149586_cov_42.053596_56261_55314	56261	55314	-	Bifunctional protein: zinc-containing alcohol dehydrogenase; quinone oxidoreductase (NADPH:quinone reductase) (EC 1.1.1.-); Similar to arginate lyase	FIG01393346	ff
NODE_31_length_149586_cov_42.053596	fig|6666666.34159.peg.473	peg	NODE_31_length_149586_cov_42.053596_56764_56291	56764	56291	-	Non-specific DNA-binding protein Dps / Iron-binding ferritin-like antioxidant protein / Ferroxidase (EC 1.16.3.1)	FIG00109595	idu(1);Oxidative_stress idu(1);Oxidative_stress idu(1);Oxidative_stress
NODE_31_length_149586_cov_42.053596	fig|6666666.34159.rna.10	rna	NODE_31_length_149586_cov_42.053596_56999_57082	56999	57082	+	tRNA-Leu-CAG		isu;tRNAs
NODE_31_length_149586_cov_42.053596	fig|6666666.34159.peg.474	peg	NODE_31_length_149586_cov_42.053596_57106_57729	57106	57729	+	Thiamin pyrophosphokinase (EC 2.7.6.2)	FIG00001175	isu;Thiamin_biosynthesis
NODE_31_length_149586_cov_42.053596	fig|6666666.34159.peg.475	peg	NODE_31_length_149586_cov_42.053596_57926_59734	57926	59734	+	Translation elongation factor LepA	FIG00000165	isu;Heat_shock_dnaK_gene_cluster_extended isu;Translation_elongation_factors_bacterial
NODE_31_length_149586_cov_42.053596	fig|6666666.34159.peg.476	peg	NODE_31_length_149586_cov_42.053596_59894_60685	59894	60685	+	Undecaprenyl pyrophosphate synthetase (EC 2.5.1.31)	FIG00000110	ff
NODE_31_length_149586_cov_42.053596	fig|6666666.34159.peg.477	peg	NODE_31_length_149586_cov_42.053596_60682_61536	60682	61536	+	Phosphatidate cytidylyltransferase (EC 2.7.7.41)		
NODE_31_length_149586_cov_42.053596	fig|6666666.34159.peg.478	peg	NODE_31_length_149586_cov_42.053596_61546_62238	61546	62238	+	Cytidylate kinase (EC 2.7.4.14)	FIG00041008	isu;pyrimidine_conversions
NODE_31_length_149586_cov_42.053596	fig|6666666.34159.peg.479	peg	NODE_31_length_149586_cov_42.053596_63092_62277	63092	62277	-	Glutamine Binding Protein		
NODE_31_length_149586_cov_42.053596	fig|6666666.34159.peg.480	peg	NODE_31_length_149586_cov_42.053596_63357_64343	63357	64343	+	Ferrochelatase, protoheme ferro-lyase (EC 4.99.1.1)	FIG00000345	isu;Heme_and_Siroheme_Biosynthesis
NODE_31_length_149586_cov_42.053596	fig|6666666.34159.peg.481	peg	NODE_31_length_149586_cov_42.053596_64612_65958	64612	65958	+	Chromosomal replication initiator protein DnaA	FIG00000441	idu(1);DNA_replication_cluster_1
NODE_31_length_149586_cov_42.053596	fig|6666666.34159.peg.482	peg	NODE_31_length_149586_cov_42.053596_67448_65955	67448	65955	-	Oligopeptide transport system permease protein OppC (TC 3.A.1.5.1)	FIG00455853	isu;ABC_transporter_oligopeptide_(TC_3.A.1.5.1)
NODE_31_length_149586_cov_42.053596	fig|6666666.34159.peg.483	peg	NODE_31_length_149586_cov_42.053596_68953_67454	68953	67454	-	Oligopeptide transport system permease protein OppB (TC 3.A.1.5.1)	FIG00945384	icw(1);ABC_transporter_oligopeptide_(TC_3.A.1.5.1)
NODE_31_length_149586_cov_42.053596	fig|6666666.34159.peg.484	peg	NODE_31_length_149586_cov_42.053596_71009_68955	71009	68955	-	Oligopeptide ABC transporter, periplasmic oligopeptide-binding protein OppA (TC 3.A.1.5.1)	FIG00451095	icw(2);ABC_transporter_oligopeptide_(TC_3.A.1.5.1) isu;Sex_pheromones_in_Enterococcus_faecalis_a
NODE_31_length_149586_cov_42.053596	fig|6666666.34159.peg.485	peg	NODE_31_length_149586_cov_42.053596_71266_72792	71266	72792	+	hypothetical protein		
NODE_31_length_149586_cov_42.053596	fig|6666666.34159.peg.486	peg	NODE_31_length_149586_cov_42.053596_73026_72868	73026	72868	-	hypothetical protein		
NODE_31_length_149586_cov_42.053596	fig|6666666.34159.peg.487	peg	NODE_31_length_149586_cov_42.053596_73107_73304	73107	73304	+	hypothetical protein		
NODE_31_length_149586_cov_42.053596	fig|6666666.34159.peg.488	peg	NODE_31_length_149586_cov_42.053596_74063_73434	74063	73434	-	hypothetical protein		
NODE_31_length_149586_cov_42.053596	fig|6666666.34159.peg.489	peg	NODE_31_length_149586_cov_42.053596_75426_74053	75426	74053	-	hypothetical protein		
NODE_31_length_149586_cov_42.053596	fig|6666666.34159.peg.490	peg	NODE_31_length_149586_cov_42.053596_76206_75430	76206	75430	-	methylase		
NODE_31_length_149586_cov_42.053596	fig|6666666.34159.peg.491	peg	NODE_31_length_149586_cov_42.053596_76495_76283	76495	76283	-	hypothetical protein		
NODE_31_length_149586_cov_42.053596	fig|6666666.34159.peg.492	peg	NODE_31_length_149586_cov_42.053596_77306_76677	77306	76677	-	hypothetical protein		
NODE_31_length_149586_cov_42.053596	fig|6666666.34159.peg.493	peg	NODE_31_length_149586_cov_42.053596_77567_77367	77567	77367	-	hypothetical protein		
NODE_31_length_149586_cov_42.053596	fig|6666666.34159.peg.494	peg	NODE_31_length_149586_cov_42.053596_77704_80277	77704	80277	+	Glycogen phosphorylase (EC 2.4.1.1)	FIG00000476	idu(1);Glycogen_metabolism idu(1);Maltose_and_Maltodextrin_Utilization idu(1);Glycogen_metabolism_cluster
NODE_31_length_149586_cov_42.053596	fig|6666666.34159.peg.495	peg	NODE_31_length_149586_cov_42.053596_81061_80339	81061	80339	-	Na(+)-translocating NADH-quinone reductase subunit E (EC 1.6.5.-)	FIG00001289	icw(1);Na(+)-translocating_NADH-quinone_oxidoreductase_and_rnf-like_group_of_electron_transport_complexes
NODE_31_length_149586_cov_42.053596	fig|6666666.34159.peg.496	peg	NODE_31_length_149586_cov_42.053596_81706_81074	81706	81074	-	Na(+)-translocating NADH-quinone reductase subunit D (EC 1.6.5.-)	FIG00002053	isu;Na(+)-translocating_NADH-quinone_oxidoreductase_and_rnf-like_group_of_electron_transport_complexes
NODE_31_length_149586_cov_42.053596	fig|6666666.34159.peg.497	peg	NODE_31_length_149586_cov_42.053596_82645_81731	82645	81731	-	Na(+)-translocating NADH-quinone reductase subunit C (EC 1.6.5.-)	FIG00036997	icw(2);Na(+)-translocating_NADH-quinone_oxidoreductase_and_rnf-like_group_of_electron_transport_complexes
NODE_31_length_149586_cov_42.053596	fig|6666666.34159.peg.498	peg	NODE_31_length_149586_cov_42.053596_84164_82626	84164	82626	-	Na(+)-translocating NADH-quinone reductase subunit B (EC 1.6.5.-)	FIG00033847	icw(3);Na(+)-translocating_NADH-quinone_oxidoreductase_and_rnf-like_group_of_electron_transport_complexes
NODE_31_length_149586_cov_42.053596	fig|6666666.34159.peg.499	peg	NODE_31_length_149586_cov_42.053596_84407_89668	84407	89668	+	hypothetical protein	FIG00638284	ff
NODE_31_length_149586_cov_42.053596	fig|6666666.34159.peg.500	peg	NODE_31_length_149586_cov_42.053596_91706_89670	91706	89670	-	hypothetical protein		
NODE_31_length_149586_cov_42.053596	fig|6666666.34159.peg.501	peg	NODE_31_length_149586_cov_42.053596_92118_92945	92118	92945	+	unknown protein	FIG00764722	ff
NODE_31_length_149586_cov_42.053596	fig|6666666.34159.peg.502	peg	NODE_31_length_149586_cov_42.053596_92963_93709	92963	93709	+	Competence protein F homolog, phosphoribosyltransferase domain; protein YhgH required for utilization of DNA as sole source of carbon and energy		isu;Biotin_biosynthesis_Experimental isu;CBSS-216591.1.peg.168
NODE_31_length_149586_cov_42.053596	fig|6666666.34159.peg.503	peg	NODE_31_length_149586_cov_42.053596_94259_93690	94259	93690	-	hypothetical protein		
NODE_31_length_149586_cov_42.053596	fig|6666666.34159.peg.504	peg	NODE_31_length_149586_cov_42.053596_94525_94680	94525	94680	+	hypothetical protein		
NODE_31_length_149586_cov_42.053596	fig|6666666.34159.peg.505	peg	NODE_31_length_149586_cov_42.053596_95553_94762	95553	94762	-	hypothetical protein		
NODE_31_length_149586_cov_42.053596	fig|6666666.34159.peg.506	peg	NODE_31_length_149586_cov_42.053596_96110_95619	96110	95619	-	hypothetical protein		
NODE_31_length_149586_cov_42.053596	fig|6666666.34159.peg.507	peg	NODE_31_length_149586_cov_42.053596_97594_96203	97594	96203	-	2-methylcitrate dehydratase (EC 4.2.1.79)	FIG00001287	icw(1);Methylcitrate_cycle
NODE_31_length_149586_cov_42.053596	fig|6666666.34159.peg.508	peg	NODE_31_length_149586_cov_42.053596_98771_97638	98771	97638	-	2-methylcitrate synthase (EC 2.3.3.5)	FIG00001030	icw(2);Methylcitrate_cycle
NODE_31_length_149586_cov_42.053596	fig|6666666.34159.peg.509	peg	NODE_31_length_149586_cov_42.053596_99632_98781	99632	98781	-	Methylisocitrate lyase (EC 4.1.3.30)	FIG00003486	isu;Methylcitrate_cycle
NODE_31_length_149586_cov_42.053596	fig|6666666.34159.peg.510	peg	NODE_31_length_149586_cov_42.053596_99796_100554	99796	1E+05	+	3-hydroxybutyryl-CoA dehydratase (EC 4.2.1.55)	FIG00002255	isu;Polyhydroxybutyrate_metabolism isu;Acetyl-CoA_fermentation_to_Butyrate
NODE_31_length_149586_cov_42.053596	fig|6666666.34159.peg.511	peg	NODE_31_length_149586_cov_42.053596_100564_101898	100564	1E+05	+	278aa long hypothetical 3-hydroxybutyryl-CoA dehydrogenase		
NODE_31_length_149586_cov_42.053596	fig|6666666.34159.peg.512	peg	NODE_31_length_149586_cov_42.053596_101908_103143	101908	1E+05	+	Thiolase		
NODE_31_length_149586_cov_42.053596	fig|6666666.34159.peg.513	peg	NODE_31_length_149586_cov_42.053596_103156_104301	103156	1E+05	+	Butyryl-CoA dehydrogenase (EC 1.3.99.2)	FIG00009149	ff
NODE_31_length_149586_cov_42.053596	fig|6666666.34159.peg.514	peg	NODE_31_length_149586_cov_42.053596_104618_104319	104618	1E+05	-	hypothetical protein		
NODE_31_length_149586_cov_42.053596	fig|6666666.34159.peg.515	peg	NODE_31_length_149586_cov_42.053596_104786_106072	104786	1E+05	+	Seryl-tRNA synthetase (EC 6.1.1.11)	FIG00000121	isu;Glycine_and_Serine_Utilization isu;tRNA_aminoacylation,_Ser isu;CBSS-326442.4.peg.1852
NODE_31_length_149586_cov_42.053596	fig|6666666.34159.peg.516	peg	NODE_31_length_149586_cov_42.053596_106111_107394	106111	1E+05	+	COG1362: Aspartyl aminopeptidase		
NODE_31_length_149586_cov_42.053596	fig|6666666.34159.peg.517	peg	NODE_31_length_149586_cov_42.053596_108825_107428	108825	1E+05	-	hypothetical protein		
NODE_31_length_149586_cov_42.053596	fig|6666666.34159.peg.518	peg	NODE_31_length_149586_cov_42.053596_109068_111038	109068	1E+05	+	1-hydroxy-2-methyl-2-(E)-butenyl 4-diphosphate synthase (EC 1.17.7.1)	FIG00000449	isu;Nonmevalonate_Branch_of_Isoprenoid_Biosynthesis isu;Isoprenoid_Biosynthesis
NODE_31_length_149586_cov_42.053596	fig|6666666.34159.peg.519	peg	NODE_31_length_149586_cov_42.053596_111168_111019	111168	1E+05	-	hypothetical protein		
NODE_31_length_149586_cov_42.053596	fig|6666666.34159.peg.520	peg	NODE_31_length_149586_cov_42.053596_113578_111227	113578	1E+05	-	hypothetical protein	FIG00638284	ff

NODE_31_length_149586_cov_42.053596	fig|6666666.34159.peg.521	peg	NODE_31_length_149586_cov_42.053596_113684_113553	113684	1E+05	-	hypothetical protein		
NODE_31_length_149586_cov_42.053596	fig|6666666.34159.peg.522	peg	NODE_31_length_149586_cov_42.053596_113922_115274	113922	1E+05	+	Hexose phosphate uptake regulatory protein UhpC	FIG00004821	idu(1);Hexose_Phosphate_Uptake_System
NODE_31_length_149586_cov_42.053596	fig|6666666.34159.peg.523	peg	NODE_31_length_149586_cov_42.053596_115714_115313	115714	1E+05	-	hypothetical protein		
NODE_31_length_149586_cov_42.053596	fig|6666666.34159.peg.524	peg	NODE_31_length_149586_cov_42.053596_116069_115863	116069	1E+05	-	hypothetical protein		
NODE_31_length_149586_cov_42.053596	fig|6666666.34159.peg.525	peg	NODE_31_length_149586_cov_42.053596_116394_120158	116394	1E+05	+	DNA polymerase III alpha subunit (EC 2.7.7.7)	FIG00041038	isu;Inteins
NODE_31_length_149586_cov_42.053596	fig|6666666.34159.peg.526	peg	NODE_31_length_149586_cov_42.053596_120215_121213	120215	1E+05	+	hypothetical protein		
NODE_31_length_149586_cov_42.053596	fig|6666666.34159.peg.527	peg	NODE_31_length_149586_cov_42.053596_121194_121757	121194	1E+05	+	FIG00899450: hypothetical protein	FIG00899449	ff
NODE_31_length_149586_cov_42.053596	fig|6666666.34159.peg.528	peg	NODE_31_length_149586_cov_42.053596_121738_122181	121738	1E+05	+	Serine-protein kinase RsbW (EC 2.7.11.1)		isu;SigmaB_stress_responce_regulation
NODE_31_length_149586_cov_42.053596	fig|6666666.34159.peg.529	peg	NODE_31_length_149586_cov_42.053596_122630_122178	122630	1E+05	-	FIG00899519: hypothetical protein	FIG00899516	ff
NODE_31_length_149586_cov_42.053596	fig|6666666.34159.peg.530	peg	NODE_31_length_149586_cov_42.053596_122855_124138	122855	1E+05	+	D-alanyl-D-alanine carboxypeptidase (EC 3.4.16.4)	FIG00561498	isu;Murein_Hydrolases isu;CBSS-84588.1.peg.1247 isu;Metallocarboxypeptidases_(EC_3.4.17.-)
NODE_31_length_149586_cov_42.053596	fig|6666666.34159.peg.531	peg	NODE_31_length_149586_cov_42.053596_124150_125175	124150	1E+05	+	Oxidoreductase	FIG01321859	ff
NODE_31_length_149586_cov_42.053596	fig|6666666.34159.peg.532	peg	NODE_31_length_149586_cov_42.053596_125265_126488	125265	1E+05	+	hypothetical protein		
NODE_31_length_149586_cov_42.053596	fig|6666666.34159.peg.533	peg	NODE_31_length_149586_cov_42.053596_127333_126485	127333	1E+05	-	Dimethyladenosine transferase (EC 2.1.1.-)	FIG00000225	ff
NODE_31_length_149586_cov_42.053596	fig|6666666.34159.peg.534	peg	NODE_31_length_149586_cov_42.053596_128407_127343	128407	1E+05	-	hypothetical protein	FIG00638284	ff
NODE_31_length_149586_cov_42.053596	fig|6666666.34159.peg.535	peg	NODE_31_length_149586_cov_42.053596_128693_129115	128693	1E+05	+	hypothetical protein		
NODE_31_length_149586_cov_42.053596	fig|6666666.34159.peg.536	peg	NODE_31_length_149586_cov_42.053596_129176_129460	129176	1E+05	+	hypothetical protein		
NODE_31_length_149586_cov_42.053596	fig|6666666.34159.peg.537	peg	NODE_31_length_149586_cov_42.053596_129511_129873	129511	1E+05	+	putative mip (macrophage infectivity potentiator, fkbp-type peptidyl-prolyl cis-trans isomerase)		
NODE_31_length_149586_cov_42.053596	fig|6666666.34159.peg.538	peg	NODE_31_length_149586_cov_42.053596_130180_130401	130180	1E+05	+	hypothetical protein		
NODE_31_length_149586_cov_42.053596	fig|6666666.34159.peg.539	peg	NODE_31_length_149586_cov_42.053596_130533_131300	130533	1E+05	+	hypothetical protein		
NODE_31_length_149586_cov_42.053596	fig|6666666.34159.peg.540	peg	NODE_31_length_149586_cov_42.053596_132202_132906	132202	1E+05	+	hypothetical protein		
NODE_31_length_149586_cov_42.053596	fig|6666666.34159.peg.541	peg	NODE_31_length_149586_cov_42.053596_133063_132893	133063	1E+05	-	hypothetical protein		
NODE_31_length_149586_cov_42.053596	fig|6666666.34159.peg.542	peg	NODE_31_length_149586_cov_42.053596_133096_133344	133096	1E+05	+	hypothetical protein		
NODE_31_length_149586_cov_42.053596	fig|6666666.34159.peg.543	peg	NODE_31_length_149586_cov_42.053596_133892_134032	133892	1E+05	+	hypothetical protein		
NODE_31_length_149586_cov_42.053596	fig|6666666.34159.peg.544	peg	NODE_31_length_149586_cov_42.053596_134029_134733	134029	1E+05	+	hypothetical protein		
NODE_31_length_149586_cov_42.053596	fig|6666666.34159.peg.545	peg	NODE_31_length_149586_cov_42.053596_134869_134720	134869	1E+05	-	hypothetical protein		
NODE_31_length_149586_cov_42.053596	fig|6666666.34159.peg.546	peg	NODE_31_length_149586_cov_42.053596_135724_135858	135724	1E+05	+	hypothetical protein		
NODE_31_length_149586_cov_42.053596	fig|6666666.34159.peg.547	peg	NODE_31_length_149586_cov_42.053596_136245_136054	136245	1E+05	-	hypothetical protein		
NODE_31_length_149586_cov_42.053596	fig|6666666.34159.peg.548	peg	NODE_31_length_149586_cov_42.053596_136316_137020	136316	1E+05	+	hypothetical protein		
NODE_31_length_149586_cov_42.053596	fig|6666666.34159.peg.549	peg	NODE_31_length_149586_cov_42.053596_137630_137466	137630	1E+05	-	hypothetical protein		
NODE_31_length_149586_cov_42.053596	fig|6666666.34159.peg.550	peg	NODE_31_length_149586_cov_42.053596_138453_137644	138453	1E+05	-	NADPH dependent preQ0 reductase (EC 1.7.1.13)	FIG00001032	ff
NODE_31_length_149586_cov_42.053596	fig|6666666.34159.peg.551	peg	NODE_31_length_149586_cov_42.053596_141542_138513	141542	1E+05	-	hypothetical protein	FIG00638284	ff
NODE_31_length_149586_cov_42.053596	fig|6666666.34159.peg.552	peg	NODE_31_length_149586_cov_42.053596_141976_141563	141976	1E+05	-	hypothetical protein		
NODE_31_length_149586_cov_42.053596	fig|6666666.34159.peg.553	peg	NODE_31_length_149586_cov_42.053596_142367_141966	142367	1E+05	-	hypothetical protein		
NODE_31_length_149586_cov_42.053596	fig|6666666.34159.peg.554	peg	NODE_31_length_149586_cov_42.053596_142449_142601	142449	1E+05	+	hypothetical protein		
NODE_31_length_149586_cov_42.053596	fig|6666666.34159.peg.555	peg	NODE_31_length_149586_cov_42.053596_142832_145267	142832	1E+05	+	Xylulose-5-phosphate phosphoketolase (EC 4.1.2.9); Fructose-6-phosphate phosphoketolase (EC 4.1.2.22)	FIG00137542	isu;Fermentations:_Lactate isu;Fermentations:_Lactate isu;Pentose_phosphate_pathway
NODE_31_length_149586_cov_42.053596	fig|6666666.34159.peg.556	peg	NODE_31_length_149586_cov_42.053596_145322_145621	145322	1E+05	+	mannose-6-phosphate isomerase		
NODE_31_length_149586_cov_42.053596	fig|6666666.34159.peg.557	peg	NODE_31_length_149586_cov_42.053596_147045_145627	147045	1E+05	-	similar to hypothetical protein BC002942		
NODE_31_length_149586_cov_42.053596	fig|6666666.34159.peg.558	peg	NODE_31_length_149586_cov_42.053596_147698_147135	147698	1E+05	-	FIG01123217: hypothetical protein	FIG01123215	ff
NODE_31_length_149586_cov_42.053596	fig|6666666.34159.peg.559	peg	NODE_31_length_149586_cov_42.053596_148149_147811	148149	1E+05	-	Ethidium bromide-methyl viologen resistance protein EmrE		
NODE_31_length_149586_cov_42.053596	fig|6666666.34159.peg.560	peg	NODE_31_length_149586_cov_42.053596_149028_148162	149028	1E+05	-	hypothetical protein		
NODE_35_length_946_cov_95.930748	fig|6666666.34159.peg.561	peg	NODE_35_length_946_cov_95.930748_62_469	62	469	+	Mobile element protein	FIG01306568	ff
NODE_36_length_2370_cov_47.893673	fig|6666666.34159.peg.562	peg	NODE_36_length_2370_cov_47.893673_818_705	818	705	-	hypothetical protein		
NODE_36_length_2370_cov_47.893673	fig|6666666.34159.peg.563	peg	NODE_36_length_2370_cov_47.893673_1300_917	1300	917	-	hypothetical protein		
NODE_36_length_2370_cov_47.893673	fig|6666666.34159.peg.564	peg	NODE_36_length_2370_cov_47.893673_2014_1301	2014	1301	-	guanine-specific ribonuclease N1 and T1		
NODE_3_length_285037_cov_42.248081	fig|6666666.34159.peg.565	peg	NODE_3_length_285037_cov_42.248081_566_1318	566	1318	+	hypothetical protein	FIG00638284	ff
NODE_3_length_285037_cov_42.248081	fig|6666666.34159.peg.566	peg	NODE_3_length_285037_cov_42.248081_1527_1832	1527	1832	+	hypothetical protein		
NODE_3_length_285037_cov_42.248081	fig|6666666.34159.peg.567	peg	NODE_3_length_285037_cov_42.248081_1998_2153	1998	2153	+	hypothetical protein		
NODE_3_length_285037_cov_42.248081	fig|6666666.34159.peg.568	peg	NODE_3_length_285037_cov_42.248081_2333_3178	2333	3178	+	hypothetical protein		
NODE_3_length_285037_cov_42.248081	fig|6666666.34159.rna.11	rna	NODE_3_length_285037_cov_42.248081_5007_4926	5007	4926	-	tRNA-Leu-TAG		
NODE_3_length_285037_cov_42.248081	fig|6666666.34159.peg.569	peg	NODE_3_length_285037_cov_42.248081_6246_5053	6246	5053	-	Branched-chain amino acid transport system carrier protein		
NODE_3_length_285037_cov_42.248081	fig|6666666.34159.peg.570	peg	NODE_3_length_285037_cov_42.248081_7921_6602	7921	6602	-	hypothetical protein	FIG00638284	ff
NODE_3_length_285037_cov_42.248081	fig|6666666.34159.peg.571	peg	NODE_3_length_285037_cov_42.248081_8027_8434	8027	8434	+	UPF0225 protein YchJ		isu;Broadly_distributed_proteins_not_in_subsystems
NODE_3_length_285037_cov_42.248081	fig|6666666.34159.peg.572	peg	NODE_3_length_285037_cov_42.248081_8543_9289	8543	9289	+	hypothetical protein		
NODE_3_length_285037_cov_42.248081	fig|6666666.34159.peg.573	peg	NODE_3_length_285037_cov_42.248081_9847_9323	9847	9323	-	Bis(5'-nucleosyl)-tetraphosphatase (asymmetrical) (EC 3.6.1.17)	FIG00008590	isu;CBSS-176299.4.peg.1996A isu;pyrimidine_conversions
NODE_3_length_285037_cov_42.248081	fig|6666666.34159.peg.574	peg	NODE_3_length_285037_cov_42.248081_10070_10507	10070	10507	+	putative MutT/nudix-family hydrolase		
NODE_3_length_285037_cov_42.248081	fig|6666666.34159.peg.575	peg	NODE_3_length_285037_cov_42.248081_10488_10628	10488	10628	+	hypothetical protein		
NODE_3_length_285037_cov_42.248081	fig|6666666.34159.peg.576	peg	NODE_3_length_285037_cov_42.248081_11502_10675	11502	10675	-	3-oxoacyl-[acyl-carrier protein] reductase (EC 1.1.1.100)	FIG00621114	idu(12);CBSS-246196.1.peg.364 idu(12);Fatty_Acid_Biosynthesis_FASII
NODE_3_length_285037_cov_42.248081	fig|6666666.34159.peg.577	peg	NODE_3_length_285037_cov_42.248081_12087_11818	12087	11818	-	putative transposase OrfA		
NODE_3_length_285037_cov_42.248081	fig|6666666.34159.peg.578	peg	NODE_3_length_285037_cov_42.248081_12339_12118	12339	12118	-	regulatory protein, MerR:Resolvase, N-terminal		

NODE_3_length_285037_cov_42.248081	fig|6666666.34159.peg.579	peg	NODE_3_length_285037_cov_42.248081_12712_12422	12712	12422	-	3-deoxy-D-manno-octulosonate 8-phosphate phosphatase (EC 3.1.3.45)		isu;KDO2-Lipid_A_biosynthesis
NODE_3_length_285037_cov_42.248081	fig|6666666.34159.peg.580	peg	NODE_3_length_285037_cov_42.248081_12840_12703	12840	12703	-	hypothetical protein		
NODE_3_length_285037_cov_42.248081	fig|6666666.34159.peg.581	peg	NODE_3_length_285037_cov_42.248081_13289_12837	13289	12837	-	Conserved hypothetical protein 22		
NODE_3_length_285037_cov_42.248081	fig|6666666.34159.peg.582	peg	NODE_3_length_285037_cov_42.248081_15081_13279	15081	13279	-	hypothetical protein	FIG00638284	ff
NODE_3_length_285037_cov_42.248081	fig|6666666.34159.peg.583	peg	NODE_3_length_285037_cov_42.248081_17578_15185	17578	15185	-	Lead, cadmium, zinc and mercury transporting ATPase (EC 3.6.3.3) (EC 3.6.3.5); Copper-translocating P-type ATPase (EC 3.6.3.4)	FIG00658111	idu(3);Copper_Transport_System idu(3);CBSS-196620.1.peg.2477 idu(3);Copper_homeostasis
NODE_3_length_285037_cov_42.248081	fig|6666666.34159.peg.584	peg	NODE_3_length_285037_cov_42.248081_17689_18225	17689	18225	+	Peptide deformylase (EC 3.5.1.88)	FIG00000017	idu(1);CBSS-89187.3.peg.2957 idu(1);Translation_termination_factors_bacterial
NODE_3_length_285037_cov_42.248081	fig|6666666.34159.peg.585	peg	NODE_3_length_285037_cov_42.248081_18513_18226	18513	18226	-	Preprotein translocase subunit SecG (TC 3.A.5.1.1)	FIG00001563	icw(1);CBSS-331978.3.peg.2915
NODE_3_length_285037_cov_42.248081	fig|6666666.34159.peg.586	peg	NODE_3_length_285037_cov_42.248081_19298_18513	19298	18513	-	Triosephosphate isomerase (EC 5.3.1.1)	FIG00000076	isu;Calvin-Benson_cycle isu;Glycolysis_and_Gluconeogenesis isu;CBSS-331978.3.peg.2915
NODE_3_length_285037_cov_42.248081	fig|6666666.34159.peg.587	peg	NODE_3_length_285037_cov_42.248081_19460_20059	19460	20059	+	DNA-3-methyladenine glycosylase II (EC 3.2.2.21)	FIG00001134	isu;DNA_Repair_Base_Excision
NODE_3_length_285037_cov_42.248081	fig|6666666.34159.peg.588	peg	NODE_3_length_285037_cov_42.248081_20079_20732	20079	20732	+	Transporter	FIG00450284	ff
NODE_3_length_285037_cov_42.248081	fig|6666666.34159.peg.589	peg	NODE_3_length_285037_cov_42.248081_20732_21415	20732	21415	+	hypothetical protein		
NODE_3_length_285037_cov_42.248081	fig|6666666.34159.peg.590	peg	NODE_3_length_285037_cov_42.248081_21419_21874	21419	21874	+	hypothetical protein		
NODE_3_length_285037_cov_42.248081	fig|6666666.34159.peg.591	peg	NODE_3_length_285037_cov_42.248081_22791_21871	22791	21871	-	hypothetical protein		
NODE_3_length_285037_cov_42.248081	fig|6666666.34159.peg.592	peg	NODE_3_length_285037_cov_42.248081_24640_22832	24640	22832	-	hypothetical protein		
NODE_3_length_285037_cov_42.248081	fig|6666666.34159.peg.593	peg	NODE_3_length_285037_cov_42.248081_26641_24659	26641	24659	-	hypothetical protein	FIG00638284	ff
NODE_3_length_285037_cov_42.248081	fig|6666666.34159.peg.594	peg	NODE_3_length_285037_cov_42.248081_28222_26732	28222	26732	-	hypothetical protein		
NODE_3_length_285037_cov_42.248081	fig|6666666.34159.peg.595	peg	NODE_3_length_285037_cov_42.248081_29261_28305	29261	28305	-	Formiminoglutamase (EC 3.5.3.8)	FIG00138112	isu;Histidine_Degradation
NODE_3_length_285037_cov_42.248081	fig|6666666.34159.peg.596	peg	NODE_3_length_285037_cov_42.248081_30486_29254	30486	29254	-	Imidazolonepropionase (EC 3.5.2.7)	FIG00057840	icw(1);Histidine_Degradation
NODE_3_length_285037_cov_42.248081	fig|6666666.34159.peg.597	peg	NODE_3_length_285037_cov_42.248081_32164_30479	32164	30479	-	Urocanate hydratase (EC 4.2.1.49)	FIG00094176	icw(2);Histidine_Degradation
NODE_3_length_285037_cov_42.248081	fig|6666666.34159.peg.598	peg	NODE_3_length_285037_cov_42.248081_33675_32176	33675	32176	-	Histidine ammonia-lyase (EC 4.3.1.3)	FIG00091940	icw(3);Histidine_Degradation
NODE_3_length_285037_cov_42.248081	fig|6666666.34159.peg.599	peg	NODE_3_length_285037_cov_42.248081_34292_33795	34292	33795	-	hypothetical protein		
NODE_3_length_285037_cov_42.248081	fig|6666666.34159.peg.600	peg	NODE_3_length_285037_cov_42.248081_35189_34398	35189	34398	-	hypothetical protein	FIG00638284	ff
NODE_3_length_285037_cov_42.248081	fig|6666666.34159.peg.601	peg	NODE_3_length_285037_cov_42.248081_35425_35694	35425	35694	+	SSU ribosomal protein S15p (S13e)	FIG00000144	ff
NODE_3_length_285037_cov_42.248081	fig|6666666.34159.peg.602	peg	NODE_3_length_285037_cov_42.248081_36006_35860	36006	35860	-	hypothetical protein		
NODE_3_length_285037_cov_42.248081	fig|6666666.34159.peg.603	peg	NODE_3_length_285037_cov_42.248081_35985_38075	35985	38075	+	Polyribonucleotide nucleotidyltransferase (EC 2.7.7.8)	FIG00000391	isu;Polyadenylation_bacterial
NODE_3_length_285037_cov_42.248081	fig|6666666.34159.peg.604	peg	NODE_3_length_285037_cov_42.248081_38201_40789	38201	40789	+	hypothetical protein		
NODE_3_length_285037_cov_42.248081	fig|6666666.34159.peg.605	peg	NODE_3_length_285037_cov_42.248081_40917_43445	40917	43445	+	hypothetical protein	FIG00638284	ff
NODE_3_length_285037_cov_42.248081	fig|6666666.34159.peg.606	peg	NODE_3_length_285037_cov_42.248081_43520_45325	43520	45325	+	hypothetical protein	FIG00638284	ff
NODE_3_length_285037_cov_42.248081	fig|6666666.34159.peg.607	peg	NODE_3_length_285037_cov_42.248081_45421_45308	45421	45308	-	hypothetical protein		
NODE_3_length_285037_cov_42.248081	fig|6666666.34159.peg.608	peg	NODE_3_length_285037_cov_42.248081_45402_47723	45402	47723	+	hypothetical protein	FIG00638284	ff
NODE_3_length_285037_cov_42.248081	fig|6666666.34159.peg.609	peg	NODE_3_length_285037_cov_42.248081_47864_47724	47864	47724	-	hypothetical protein		
NODE_3_length_285037_cov_42.248081	fig|6666666.34159.peg.610	peg	NODE_3_length_285037_cov_42.248081_47918_49810	47918	49810	+	hypothetical protein	FIG00638284	ff
NODE_3_length_285037_cov_42.248081	fig|6666666.34159.peg.611	peg	NODE_3_length_285037_cov_42.248081_50047_50217	50047	50217	+	hypothetical protein		
NODE_3_length_285037_cov_42.248081	fig|6666666.34159.peg.612	peg	NODE_3_length_285037_cov_42.248081_53219_50262	53219	50262	-	hypothetical protein	FIG00638284	ff
NODE_3_length_285037_cov_42.248081	fig|6666666.34159.peg.613	peg	NODE_3_length_285037_cov_42.248081_54583_53423	54583	53423	-	hypothetical protein		
NODE_3_length_285037_cov_42.248081	fig|6666666.34159.peg.614	peg	NODE_3_length_285037_cov_42.248081_54728_55609	54728	55609	+	Methylenetetrahydrofolate dehydrogenase (NADP+) (EC 1.5.1.5) / Methenyltetrahydrofolate cyclohydrolase (EC 3.5.4.9)	FIG00000105	isu;One-carbon_metabolism_by_tetrahydropterines isu;One
NODE_3_length_285037_cov_42.248081	fig|6666666.34159.peg.615	peg	NODE_3_length_285037_cov_42.248081_55690_56634	55690	56634	+	thiamine biosynthesis lipoprotein		
NODE_3_length_285037_cov_42.248081	fig|6666666.34159.peg.616	peg	NODE_3_length_285037_cov_42.248081_57150_56614	57150	56614	-	type III secretion chaperone	FIG00493336	ff
NODE_3_length_285037_cov_42.248081	fig|6666666.34159.peg.617	peg	NODE_3_length_285037_cov_42.248081_58204_57290	58204	57290	-	probable XerD protein		
NODE_3_length_285037_cov_42.248081	fig|6666666.34159.peg.618	peg	NODE_3_length_285037_cov_42.248081_58734_58201	58734	58201	-	hypothetical protein		
NODE_3_length_285037_cov_42.248081	fig|6666666.34159.peg.619	peg	NODE_3_length_285037_cov_42.248081_60343_58721	60343	58721	-	Glucose-6-phosphate isomerase (EC 5.3.1.9)	FIG00000245	isu;Glycolysis_and_Gluconeogenesis
NODE_3_length_285037_cov_42.248081	fig|6666666.34159.peg.620	peg	NODE_3_length_285037_cov_42.248081_60595_60431	60595	60431	-	hypothetical protein		
NODE_3_length_285037_cov_42.248081	fig|6666666.34159.peg.621	peg	NODE_3_length_285037_cov_42.248081_61544_61131	61544	61131	-	hypothetical protein		
NODE_3_length_285037_cov_42.248081	fig|6666666.34159.peg.622	peg	NODE_3_length_285037_cov_42.248081_61827_61696	61827	61696	-	hypothetical protein		
NODE_3_length_285037_cov_42.248081	fig|6666666.34159.peg.623	peg	NODE_3_length_285037_cov_42.248081_61985_62176	61985	62176	+	hypothetical protein		
NODE_3_length_285037_cov_42.248081	fig|6666666.34159.peg.624	peg	NODE_3_length_285037_cov_42.248081_63246_62368	63246	62368	-	hypothetical protein		
NODE_3_length_285037_cov_42.248081	fig|6666666.34159.peg.625	peg	NODE_3_length_285037_cov_42.248081_63527_63411	63527	63411	-	hypothetical protein		
NODE_3_length_285037_cov_42.248081	fig|6666666.34159.peg.626	peg	NODE_3_length_285037_cov_42.248081_64586_63543	64586	63543	-	hypothetical protein		
NODE_3_length_285037_cov_42.248081	fig|6666666.34159.peg.627	peg	NODE_3_length_285037_cov_42.248081_65302_64598	65302	64598	-	tRNA (guanine46-N7-)-methyltransferase (EC 2.1.1.33)	FIG00002733	isu;tRNA_modification_Bacteria isu;RNA_methylation
NODE_3_length_285037_cov_42.248081	fig|6666666.34159.peg.628	peg	NODE_3_length_285037_cov_42.248081_66195_65359	66195	65359	-	rRNA small subunit methyltransferase I	FIG00000260	isu;Heat_shock_dnaK_gene_cluster_extended isu;16S_rRNA_modification_within_P_site_of_ribosome
NODE_3_length_285037_cov_42.248081	fig|6666666.34159.rna.12	rna	NODE_3_length_285037_cov_42.248081_66290_66219	66290	66219	-	tRNA-Cys-GCA		isu;tRNAs
NODE_3_length_285037_cov_42.248081	fig|6666666.34159.peg.629	peg	NODE_3_length_285037_cov_42.248081_67531_66392	67531	66392	-	Fatty acid desaturase (EC 1.14.19.1); Delta-9 fatty acid desaturase (EC 1.14.19.1)	FIG01185489	ff
NODE_3_length_285037_cov_42.248081	fig|6666666.34159.peg.630	peg	NODE_3_length_285037_cov_42.248081_67871_67617	67871	67617	-	hypothetical protein		
NODE_3_length_285037_cov_42.248081	fig|6666666.34159.peg.631	peg	NODE_3_length_285037_cov_42.248081_68019_69386	68019	69386	+	UDP-N-acetylmuramoylalanyl-D-glutamyl-2,6-diaminopimelate--D-alanyl-D-alanine ligase (EC 6.3.2.10)	FIG00024401	idu(1);Methicillin_resistance_in_Staphylococci
NODE_3_length_285037_cov_42.248081	fig|6666666.34159.peg.632	peg	NODE_3_length_285037_cov_42.248081_69399_70619	69399	70619	+	Phospho-N-acetylmuramoyl-pentapeptide-transferase (EC 2.7.8.13)	FIG00000221	ff
NODE_3_length_285037_cov_42.248081	fig|6666666.34159.peg.633	peg	NODE_3_length_285037_cov_42.248081_70631_71980	70631	71980	+	UDP-N-acetylmuramoylalanine--D-glutamate ligase (EC 6.3.2.9)	FIG00085033	ff
NODE_3_length_285037_cov_42.248081	fig|6666666.34159.peg.634	peg	NODE_3_length_285037_cov_42.248081_71993_72691	71993	72691	+	Membrane-bound lytic murein transglycosylase D precursor (EC 3.2.1.-)	FIG00008294	isu;CBSS-228410.1.peg.134 isu;Murein_Hydrolases isu;CBSS-342610.3.peg.1536
NODE_3_length_285037_cov_42.248081	fig|6666666.34159.peg.635	peg	NODE_3_length_285037_cov_42.248081_72695_73846	72695	73846	+	Cell division protein FtsW	FIG00000025	ff
NODE_3_length_285037_cov_42.248081	fig|6666666.34159.peg.636	peg	NODE_3_length_285037_cov_42.248081_73874_75052	73874	75052	+	hypothetical protein		

NODE_3_length_285037_cov_42.248081	fig|6666666.34159.peg.637	peg	NODE_3_length_285037_cov_42.248081_75980_75087	75980	75087	-	Cytidine deaminase (EC 3.5.4.5)	FIG00000720	isu;tRNA_modification_Bacteria isu;pyrimidine_conversions
NODE_3_length_285037_cov_42.248081	fig|6666666.34159.peg.638	peg	NODE_3_length_285037_cov_42.248081_75984_76157	75984	76157	+	hypothetical protein		
NODE_3_length_285037_cov_42.248081	fig|6666666.34159.peg.639	peg	NODE_3_length_285037_cov_42.248081_76182_77411	76182	77411	+	hypothetical protein		
NODE_3_length_285037_cov_42.248081	fig|6666666.34159.peg.640	peg	NODE_3_length_285037_cov_42.248081_77415_79214	77415	79214	+	hypothetical protein		
NODE_3_length_285037_cov_42.248081	fig|6666666.34159.peg.641	peg	NODE_3_length_285037_cov_42.248081_79368_80705	79368	80705	+	hypothetical protein	FIG00638284	ff
NODE_3_length_285037_cov_42.248081	fig|6666666.34159.peg.642	peg	NODE_3_length_285037_cov_42.248081_83356_80762	83356	80762	-	DNA topoisomerase I (EC 5.99.1.2)	FIG00000204	isu;Inteins icw(1);CBSS-272943.3.peg.1367 isu;pVir_Plasmid_of_Campylobacter isu;DNA_topoisomerases,_Type_I,_ATP[illegible]
NODE_3_length_285037_cov_42.248081	fig|6666666.34159.peg.643	peg	NODE_3_length_285037_cov_42.248081_84353_83391	84353	83391	-	Rossmann fold nucleotide-binding protein Smf possibly involved in DNA uptake	FIG00035268	isu;CBSS-272943.3.peg.1367
NODE_3_length_285037_cov_42.248081	fig|6666666.34159.peg.644	peg	NODE_3_length_285037_cov_42.248081_84419_84535	84419	84535	+	hypothetical protein		
NODE_3_length_285037_cov_42.248081	fig|6666666.34159.peg.645	peg	NODE_3_length_285037_cov_42.248081_84801_85481	84801	85481	+	hypothetical protein		
NODE_3_length_285037_cov_42.248081	fig|6666666.34159.peg.646	peg	NODE_3_length_285037_cov_42.248081_85617_85793	85617	85793	+	hypothetical protein		
NODE_3_length_285037_cov_42.248081	fig|6666666.34159.peg.647	peg	NODE_3_length_285037_cov_42.248081_85808_86023	85808	86023	+	hypothetical protein		
NODE_3_length_285037_cov_42.248081	fig|6666666.34159.peg.648	peg	NODE_3_length_285037_cov_42.248081_86149_86889	86149	86889	+	short-chain dehydrogenase/reductase SDR		
NODE_3_length_285037_cov_42.248081	fig|6666666.34159.peg.649	peg	NODE_3_length_285037_cov_42.248081_87647_86886	87647	86886	-	hypothetical protein		
NODE_3_length_285037_cov_42.248081	fig|6666666.34159.peg.650	peg	NODE_3_length_285037_cov_42.248081_88967_87654	88967	87654	-	Glucose-1-phosphate adenylyltransferase (EC 2.7.7.27)	FIG00000717	isu;Glycogen_metabolism isu;Glycogen_metabolism_cluster
NODE_3_length_285037_cov_42.248081	fig|6666666.34159.peg.651	peg	NODE_3_length_285037_cov_42.248081_89002_89118	89002	89118	+	hypothetical protein		
NODE_3_length_285037_cov_42.248081	fig|6666666.34159.peg.652	peg	NODE_3_length_285037_cov_42.248081_89210_90508	89210	90508	+	putative facilitator of salicylate uptake	FIG00134951	isu;Salicylate_ester_degradation isu;Salicylate_and_gentisate_catabolism
NODE_3_length_285037_cov_42.248081	fig|6666666.34159.peg.653	peg	NODE_3_length_285037_cov_42.248081_90813_91028	90813	91028	+	hypothetical protein		
NODE_3_length_285037_cov_42.248081	fig|6666666.34159.peg.654	peg	NODE_3_length_285037_cov_42.248081_91056_91919	91056	91919	+	Quinolinate phosphoribosyltransferase [decarboxylating] (EC 2.4.2.19)	FIG00000413	isu;NAD_and_NADP_cofactor_biosynthesis_global
NODE_3_length_285037_cov_42.248081	fig|6666666.34159.peg.655	peg	NODE_3_length_285037_cov_42.248081_91949_93544	91949	93544	+	Heat shock protein 60 family chaperone GroEL	FIG00000056	idu(2);Staphylococcal_pathogenicity_islands_SaPI idu(2);GroEL_GroES
NODE_3_length_285037_cov_42.248081	fig|6666666.34159.peg.656	peg	NODE_3_length_285037_cov_42.248081_93629_93495	93629	93495	-	hypothetical protein		
NODE_3_length_285037_cov_42.248081	fig|6666666.34159.peg.657	peg	NODE_3_length_285037_cov_42.248081_93609_94745	93609	94745	+	hypothetical protein	FIG00638284	ff
NODE_3_length_285037_cov_42.248081	fig|6666666.34159.peg.658	peg	NODE_3_length_285037_cov_42.248081_94742_95734	94742	95734	+	transport system permease protein		
NODE_3_length_285037_cov_42.248081	fig|6666666.34159.peg.659	peg	NODE_3_length_285037_cov_42.248081_95737_96447	95737	96447	+	Iron ABC transporter, ATP-binding protein		
NODE_3_length_285037_cov_42.248081	fig|6666666.34159.peg.660	peg	NODE_3_length_285037_cov_42.248081_96596_97894	96596	97894	+	hypothetical protein		
NODE_3_length_285037_cov_42.248081	fig|6666666.34159.peg.661	peg	NODE_3_length_285037_cov_42.248081_97891_99213	97891	99213	+	hypothetical protein		
NODE_3_length_285037_cov_42.248081	fig|6666666.34159.peg.662	peg	NODE_3_length_285037_cov_42.248081_99442_99233	99442	99233	-	hypothetical protein		
NODE_3_length_285037_cov_42.248081	fig|6666666.34159.peg.663	peg	NODE_3_length_285037_cov_42.248081_99745_100977	99745	1E+05	+	hypothetical protein		
NODE_3_length_285037_cov_42.248081	fig|6666666.34159.peg.664	peg	NODE_3_length_285037_cov_42.248081_101199_102428	101199	1E+05	+	hypothetical protein		
NODE_3_length_285037_cov_42.248081	fig|6666666.34159.peg.665	peg	NODE_3_length_285037_cov_42.248081_102579_102701	102579	1E+05	+	hypothetical protein		
NODE_3_length_285037_cov_42.248081	fig|6666666.34159.peg.666	peg	NODE_3_length_285037_cov_42.248081_102702_103160	102702	1E+05	+	hypothetical protein	FIG00638284	ff
NODE_3_length_285037_cov_42.248081	fig|6666666.34159.peg.667	peg	NODE_3_length_285037_cov_42.248081_103164_103949	103164	1E+05	+	Septum site-determining protein MinD	FIG00000519	idu(1);Septum_site-determining_cluster_Min
NODE_3_length_285037_cov_42.248081	fig|6666666.34159.peg.668	peg	NODE_3_length_285037_cov_42.248081_103958_105739	103958	1E+05	+	putative phosphoglucomutase/phosphomannomutase		
NODE_3_length_285037_cov_42.248081	fig|6666666.34159.peg.669	peg	NODE_3_length_285037_cov_42.248081_105816_107816	105816	1E+05	+	hypothetical protein	FIG00638284	ff
NODE_3_length_285037_cov_42.248081	fig|6666666.34159.peg.670	peg	NODE_3_length_285037_cov_42.248081_108399_107959	108399	1E+05	-	hypothetical protein		
NODE_3_length_285037_cov_42.248081	fig|6666666.34159.peg.671	peg	NODE_3_length_285037_cov_42.248081_109115_108408	109115	1E+05	-	FIG00758134: hypothetical protein	FIG00758132	ff
NODE_3_length_285037_cov_42.248081	fig|6666666.34159.peg.672	peg	NODE_3_length_285037_cov_42.248081_109871_109176	109871	1E+05	-	FIG00759512: hypothetical protein	FIG00759511	ff
NODE_3_length_285037_cov_42.248081	fig|6666666.34159.peg.673	peg	NODE_3_length_285037_cov_42.248081_111139_109889	111139	1E+05	-	FIG00759148: hypothetical protein	FIG00759147	ff
NODE_3_length_285037_cov_42.248081	fig|6666666.34159.peg.674	peg	NODE_3_length_285037_cov_42.248081_112605_111142	112605	1E+05	-	ribosomal protein S6 glutaminyl transferase related protein	FIG01354674	ff
NODE_3_length_285037_cov_42.248081	fig|6666666.34159.peg.675	peg	NODE_3_length_285037_cov_42.248081_113537_112812	113537	1E+05	-	hypothetical protein		
NODE_3_length_285037_cov_42.248081	fig|6666666.34159.peg.676	peg	NODE_3_length_285037_cov_42.248081_114716_113550	114716	1E+05	-	hypothetical protein		
NODE_3_length_285037_cov_42.248081	fig|6666666.34159.peg.677	peg	NODE_3_length_285037_cov_42.248081_115224_114697	115224	1E+05	-	Type III secretion chaperone protein for YopD (SycD)		
NODE_3_length_285037_cov_42.248081	fig|6666666.34159.peg.678	peg	NODE_3_length_285037_cov_42.248081_115757_115230	115757	1E+05	-	hypothetical protein		
NODE_3_length_285037_cov_42.248081	fig|6666666.34159.peg.679	peg	NODE_3_length_285037_cov_42.248081_116052_117665	116052	1E+05	+	hypothetical protein	FIG00638284	ff
NODE_3_length_285037_cov_42.248081	fig|6666666.34159.peg.680	peg	NODE_3_length_285037_cov_42.248081_117675_118010	117675	1E+05	+	membrane protein of unknown function		
NODE_3_length_285037_cov_42.248081	fig|6666666.34159.peg.681	peg	NODE_3_length_285037_cov_42.248081_119531_118011	119531	1E+05	-	hypothetical protein	FIG00638284	ff
NODE_3_length_285037_cov_42.248081	fig|6666666.34159.peg.682	peg	NODE_3_length_285037_cov_42.248081_119571_119699	119571	1E+05	+	hypothetical protein		
NODE_3_length_285037_cov_42.248081	fig|6666666.34159.peg.683	peg	NODE_3_length_285037_cov_42.248081_119696_121300	119696	1E+05	+	hypothetical protein		
NODE_3_length_285037_cov_42.248081	fig|6666666.34159.peg.684	peg	NODE_3_length_285037_cov_42.248081_121310_121918	121310	1E+05	+	NADPH:quinone oxidoreductase	FIG01132576	ff
NODE_3_length_285037_cov_42.248081	fig|6666666.34159.peg.685	peg	NODE_3_length_285037_cov_42.248081_121915_122412	121915	1E+05	+	hypothetical protein		
NODE_3_length_285037_cov_42.248081	fig|6666666.34159.peg.686	peg	NODE_3_length_285037_cov_42.248081_122990_122409	122990	1E+05	-	hypothetical protein		
NODE_3_length_285037_cov_42.248081	fig|6666666.34159.peg.687	peg	NODE_3_length_285037_cov_42.248081_123183_123752	123183	1E+05	+	Deoxycytidine triphosphate deaminase (EC 3.5.4.13)	FIG00102132	isu;pyrimidine_conversions
NODE_3_length_285037_cov_42.248081	fig|6666666.34159.peg.688	peg	NODE_3_length_285037_cov_42.248081_123794_124132	123794	1E+05	+	hypothetical protein		
NODE_3_length_285037_cov_42.248081	fig|6666666.34159.peg.689	peg	NODE_3_length_285037_cov_42.248081_124198_125454	124198	1E+05	+	CBS domain protein		
NODE_3_length_285037_cov_42.248081	fig|6666666.34159.peg.690	peg	NODE_3_length_285037_cov_42.248081_125482_126726	125482	1E+05	+	Hemolysin	FIG00005887	ff
NODE_3_length_285037_cov_42.248081	fig|6666666.34159.peg.691	peg	NODE_3_length_285037_cov_42.248081_126833_128032	126833	1E+05	+	Cold-shock DEAD-box protein A	FIG00001381	idu(1);ATP-dependent_RNA_helicases,_bacterial
NODE_3_length_285037_cov_42.248081	fig|6666666.34159.peg.692	peg	NODE_3_length_285037_cov_42.248081_128037_128792	128037	1E+05	+	hypothetical protein		
NODE_3_length_285037_cov_42.248081	fig|6666666.34159.peg.693	peg	NODE_3_length_285037_cov_42.248081_129774_128833	129774	1E+05	-	4-hydroxy-3-methylbut-2-enyl diphosphate reductase (EC 1.17.1.2)	FIG00000381	isu;Nonmevalonate_Branch_of_Isoprenoid_Biosynthesis isu;Isoprenoid_Biosynthesis
NODE_3_length_285037_cov_42.248081	fig|6666666.34159.peg.694	peg	NODE_3_length_285037_cov_42.248081_131519_129861	131519	1E+05	-	hypothetical protein	FIG00638284	ff
NODE_3_length_285037_cov_42.248081	fig|6666666.34159.peg.695	peg	NODE_3_length_285037_cov_42.248081_131821_131549	131821	1E+05	-	Acylphosphate phosphohydrolase (EC 3.6.1.7), putative	FIG00614545	isu;Pyruvate_metabolism_II:_acetyl-CoA,_acetogenesis_from_pyruvate

NODE_3_length_285037_cov_42.248081	fig|6666666.34159.peg.696	peg	NODE_3_length_285037_cov_42.248081_131930_133729	131930	1E+05	+	hypothetical protein	FIG00638284	ff
NODE_3_length_285037_cov_42.248081	fig|6666666.34159.peg.697	peg	NODE_3_length_285037_cov_42.248081_133729_137226	133729	1E+05	+	hypothetical protein	FIG00638284	ff
NODE_3_length_285037_cov_42.248081	fig|6666666.34159.peg.698	peg	NODE_3_length_285037_cov_42.248081_137348_138136	137348	1E+05	+	FIG00899508: hypothetical protein	FIG00899507	ff
NODE_3_length_285037_cov_42.248081	fig|6666666.34159.peg.699	peg	NODE_3_length_285037_cov_42.248081_138137_138907	138137	1E+05	+	Methionine ABC transporter ATP-binding protein	FIG00835431	isu;Methionine_Degradation
NODE_3_length_285037_cov_42.248081	fig|6666666.34159.peg.700	peg	NODE_3_length_285037_cov_42.248081_138911_140122	138911	1E+05	+	FIG00899462: hypothetical protein	FIG00899461	ff
NODE_3_length_285037_cov_42.248081	fig|6666666.34159.peg.701	peg	NODE_3_length_285037_cov_42.248081_140160_140729	140160	1E+05	+	transcriptional regulator, putative	FIG01333326	ff
NODE_3_length_285037_cov_42.248081	fig|6666666.34159.peg.702	peg	NODE_3_length_285037_cov_42.248081_141675_140770	141675	1E+05	-	FKBP-type peptidyl-prolyl cis-trans isomerase		
NODE_3_length_285037_cov_42.248081	fig|6666666.34159.peg.703	peg	NODE_3_length_285037_cov_42.248081_142619_141672	142619	1E+05	-	hypothetical protein		
NODE_3_length_285037_cov_42.248081	fig|6666666.34159.peg.704	peg	NODE_3_length_285037_cov_42.248081_144192_142771	144192	1E+05	-	Phytoene dehydrogenase and related proteins	FIG00139162	isu;Carotenoids
NODE_3_length_285037_cov_42.248081	fig|6666666.34159.peg.705	peg	NODE_3_length_285037_cov_42.248081_151659_144271	151659	1E+05	-	hypothetical protein	FIG00638284	ff
NODE_3_length_285037_cov_42.248081	fig|6666666.34159.peg.706	peg	NODE_3_length_285037_cov_42.248081_151916_152956	151916	2E+05	+	hypothetical protein	FIG00638284	ff
NODE_3_length_285037_cov_42.248081	fig|6666666.34159.peg.707	peg	NODE_3_length_285037_cov_42.248081_156555_152953	156555	2E+05	-	hypothetical protein	FIG00638284	ff
NODE_3_length_285037_cov_42.248081	fig|6666666.34159.peg.708	peg	NODE_3_length_285037_cov_42.248081_157117_156731	157117	2E+05	-	SSU ribosomal protein S9p (S16e)	FIG00001110	ff
NODE_3_length_285037_cov_42.248081	fig|6666666.34159.peg.709	peg	NODE_3_length_285037_cov_42.248081_157579_157136	157579	2E+05	-	LSU ribosomal protein L13p (L13Ae)	FIG00000211	ff
NODE_3_length_285037_cov_42.248081	fig|6666666.34159.peg.710	peg	NODE_3_length_285037_cov_42.248081_157762_157613	157762	2E+05	-	hypothetical protein		
NODE_3_length_285037_cov_42.248081	fig|6666666.34159.peg.711	peg	NODE_3_length_285037_cov_42.248081_157840_159153	157840	2E+05	+	tRNA-i(6)A37 methylthiotransferase	FIG00108342	isu;tRNA_processing isu;tRNA_modification_Bacteria isu;Methylthiotransferases
NODE_3_length_285037_cov_42.248081	fig|6666666.34159.peg.712	peg	NODE_3_length_285037_cov_42.248081_159309_159515	159309	2E+05	+	hypothetical protein		
NODE_3_length_285037_cov_42.248081	fig|6666666.34159.peg.713	peg	NODE_3_length_285037_cov_42.248081_159532_161253	159532	2E+05	+	hypothetical protein	FIG00638284	ff
NODE_3_length_285037_cov_42.248081	fig|6666666.34159.peg.714	peg	NODE_3_length_285037_cov_42.248081_161269_163278	161269	2E+05	+	Ribonucleotide reductase of class II (coenzyme B12-dependent) (EC 1.17.4.1)	FIG00010285	isu;Inteins isu;Ribonucleotide_reduction
NODE_3_length_285037_cov_42.248081	fig|6666666.34159.peg.715	peg	NODE_3_length_285037_cov_42.248081_163461_163922	163461	2E+05	+	hypothetical protein		
NODE_3_length_285037_cov_42.248081	fig|6666666.34159.peg.716	peg	NODE_3_length_285037_cov_42.248081_164165_165898	164165	2E+05	+	proteophosphoglycan ppg4		
NODE_3_length_285037_cov_42.248081	fig|6666666.34159.peg.717	peg	NODE_3_length_285037_cov_42.248081_165926_166885	165926	2E+05	+	hypothetical protein		
NODE_3_length_285037_cov_42.248081	fig|6666666.34159.peg.718	peg	NODE_3_length_285037_cov_42.248081_167895_166915	167895	2E+05	-	FIG00899417: hypothetical protein	FIG00899415	ff
NODE_3_length_285037_cov_42.248081	fig|6666666.34159.peg.719	peg	NODE_3_length_285037_cov_42.248081_168837_168025	168837	2E+05	-	putative batE protein	FIG00899440	ff
NODE_3_length_285037_cov_42.248081	fig|6666666.34159.peg.720	peg	NODE_3_length_285037_cov_42.248081_170559_168841	170559	2E+05	-	unknown protein	FIG00764722	ff
NODE_3_length_285037_cov_42.248081	fig|6666666.34159.peg.721	peg	NODE_3_length_285037_cov_42.248081_172280_170553	172280	2E+05	-	unknown protein	FIG00764722	ff
NODE_3_length_285037_cov_42.248081	fig|6666666.34159.peg.722	peg	NODE_3_length_285037_cov_42.248081_173371_172277	173371	2E+05	-	BatB	FIG00938010	isu;Aerotolerance_operon_in_Bacteroides_and_potentially_orthologous_operons_in_other_organisms
NODE_3_length_285037_cov_42.248081	fig|6666666.34159.peg.723	peg	NODE_3_length_285037_cov_42.248081_174489_173368	174489	2E+05	-	putative batA protein		
NODE_3_length_285037_cov_42.248081	fig|6666666.34159.peg.724	peg	NODE_3_length_285037_cov_42.248081_175338_174493	175338	2E+05	-	hypothetical protein		
NODE_3_length_285037_cov_42.248081	fig|6666666.34159.peg.725	peg	NODE_3_length_285037_cov_42.248081_176189_175335	176189	2E+05	-	Chromosome (plasmid) partitioning protein ParB / Stage 0 sporulation protein J	FIG00021843	isu;RNA_modification_and_chromosome_partitioning_cluster isu;Plasmid_replication
NODE_3_length_285037_cov_42.248081	fig|6666666.34159.peg.726	peg	NODE_3_length_285037_cov_42.248081_176361_177122	176361	2E+05	+	hypothetical protein		
NODE_3_length_285037_cov_42.248081	fig|6666666.34159.peg.727	peg	NODE_3_length_285037_cov_42.248081_177496_177684	177496	2E+05	+	hypothetical protein		
NODE_3_length_285037_cov_42.248081	fig|6666666.34159.peg.728	peg	NODE_3_length_285037_cov_42.248081_179241_177760	179241	2E+05	-	hypothetical protein		
NODE_3_length_285037_cov_42.248081	fig|6666666.34159.peg.729	peg	NODE_3_length_285037_cov_42.248081_180445_180206	180445	2E+05	-	hypothetical protein		
NODE_3_length_285037_cov_42.248081	fig|6666666.34159.peg.730	peg	NODE_3_length_285037_cov_42.248081_180942_180781	180942	2E+05	-	hypothetical protein		
NODE_3_length_285037_cov_42.248081	fig|6666666.34159.peg.731	peg	NODE_3_length_285037_cov_42.248081_181712_182503	181712	2E+05	+	hypothetical protein		
NODE_3_length_285037_cov_42.248081	fig|6666666.34159.peg.732	peg	NODE_3_length_285037_cov_42.248081_183438_182647	183438	2E+05	-	hypothetical protein		
NODE_3_length_285037_cov_42.248081	fig|6666666.34159.peg.733	peg	NODE_3_length_285037_cov_42.248081_183856_184635	183856	2E+05	+	hypothetical protein		
NODE_3_length_285037_cov_42.248081	fig|6666666.34159.peg.734	peg	NODE_3_length_285037_cov_42.248081_184798_185574	184798	2E+05	+	hypothetical protein		
NODE_3_length_285037_cov_42.248081	fig|6666666.34159.peg.735	peg	NODE_3_length_285037_cov_42.248081_185725_186471	185725	2E+05	+	hypothetical protein		
NODE_3_length_285037_cov_42.248081	fig|6666666.34159.peg.736	peg	NODE_3_length_285037_cov_42.248081_187665_186514	187665	2E+05	-	Methyltransferase (EC 2.1.1.-)	FIG00515945	ff
NODE_3_length_285037_cov_42.248081	fig|6666666.34159.peg.737	peg	NODE_3_length_285037_cov_42.248081_188778_187684	188778	2E+05	-	GTP-binding and nucleic acid-binding protein YchF	FIG00049915	ff
NODE_3_length_285037_cov_42.248081	fig|6666666.34159.peg.738	peg	NODE_3_length_285037_cov_42.248081_189077_190147	189077	2E+05	+	Type III secretion inner membrane protein (YscU,SpaS,EscU,HrcU,SsaU, homologous to flagellar export components)	FIG00002913	ff
NODE_3_length_285037_cov_42.248081	fig|6666666.34159.peg.739	peg	NODE_3_length_285037_cov_42.248081_190287_192347	190287	2E+05	+	Low Calcium Response D (Type III secretion inner membrane protein SctV)	FIG00899523	ff
NODE_3_length_285037_cov_42.248081	fig|6666666.34159.peg.740	peg	NODE_3_length_285037_cov_42.248081_192405_193595	192405	2E+05	+	Low Calcium Response E (CopN) (Type III secreted protein SctW)	FIG01305472	ff
NODE_3_length_285037_cov_42.248081	fig|6666666.34159.peg.741	peg	NODE_3_length_285037_cov_42.248081_193616_194086	193616	2E+05	+	probable type III secretion chaperone sycE		
NODE_3_length_285037_cov_42.248081	fig|6666666.34159.peg.742	peg	NODE_3_length_285037_cov_42.248081_194144_194404	194144	2E+05	+	hypothetical protein		
NODE_3_length_285037_cov_42.248081	fig|6666666.34159.peg.743	peg	NODE_3_length_285037_cov_42.248081_194394_195656	194394	2E+05	+	Long-chain-fatty-acid--CoA ligase (EC 6.2.1.3)		idu(5);n-Phenylalkanoic_acid_degradation idu(5);Fatty_acid_metabolism_cluster idu(5);Biotin_synthesis_cluster
NODE_3_length_285037_cov_42.248081	fig|6666666.34159.peg.744	peg	NODE_3_length_285037_cov_42.248081_195700_196377	195700	2E+05	+	3-oxoacyl-[acyl-carrier protein] reductase (EC 1.1.1.100)	FIG00621114	idu(12);CBSS-246196.1.peg.364 idu(12);Fatty_Acid_Biosynthesis_FASII
NODE_3_length_285037_cov_42.248081	fig|6666666.34159.peg.745	peg	NODE_3_length_285037_cov_42.248081_196388_197209	196388	2E+05	+	putative aminoglycoside N(3')-acetyltransferase III(EC:2.3.1.81)		
NODE_3_length_285037_cov_42.248081	fig|6666666.34159.peg.746	peg	NODE_3_length_285037_cov_42.248081_197206_198504	197206	2E+05	+	hypothetical protein	FIG00638284	ff
NODE_3_length_285037_cov_42.248081	fig|6666666.34159.peg.747	peg	NODE_3_length_285037_cov_42.248081_198720_200045	198720	2E+05	+	hypothetical protein		
NODE_3_length_285037_cov_42.248081	fig|6666666.34159.peg.748	peg	NODE_3_length_285037_cov_42.248081_200089_201774	200089	2E+05	+	hypothetical protein	FIG00638284	ff
NODE_3_length_285037_cov_42.248081	fig|6666666.34159.peg.749	peg	NODE_3_length_285037_cov_42.248081_201771_202430	201771	2E+05	+	hypothetical protein		
NODE_3_length_285037_cov_42.248081	fig|6666666.34159.peg.750	peg	NODE_3_length_285037_cov_42.248081_202829_203182	202829	2E+05	+	hypothetical protein		
NODE_3_length_285037_cov_42.248081	fig|6666666.34159.peg.751	peg	NODE_3_length_285037_cov_42.248081_203239_203364	203239	2E+05	+	hypothetical protein		
NODE_3_length_285037_cov_42.248081	fig|6666666.34159.peg.752	peg	NODE_3_length_285037_cov_42.248081_203764_203603	203764	2E+05	-	hypothetical protein		
NODE_3_length_285037_cov_42.248081	fig|6666666.34159.peg.753	peg	NODE_3_length_285037_cov_42.248081_204050_203730	204050	2E+05	-	hypothetical protein		
NODE_3_length_285037_cov_42.248081	fig|6666666.34159.peg.754	peg	NODE_3_length_285037_cov_42.248081_204967_204542	204967	2E+05	-	hypothetical protein		

NODE_3_length_285037_cov_42.248081	fig|6666666.34159.peg.755	peg	NODE_3_length_285037_cov_42.248081_204954_205088	204954	2E+05	+	hypothetical protein		
NODE_3_length_285037_cov_42.248081	fig|6666666.34159.peg.756	peg	NODE_3_length_285037_cov_42.248081_207362_205215	207362	2E+05	-	Enoyl-CoA hydratase (EC 4.2.1.17) / Delta(3)-cis-delta(2)-trans-enoyl-CoA isomerase (EC 5.3.3.8) / 3-hydroxyacyl-CoA dehydrogenase (EC 1.1.1.35) / 3-hydroxybutyryl-CoA	FIG00000622	isu;n-Phenylalkanoic_acid_degradation icw(1);n-Phenylalkanoic_acid_degradation icw(2);n-Phenylalkanoic_acid_degradation icw(2);n-
NODE_3_length_285037_cov_42.248081	fig|6666666.34159.peg.757	peg	NODE_3_length_285037_cov_42.248081_208638_207364	208638	2E+05	-	3-ketoacyl-CoA thiolase (EC 2.3.1.16) @ Acetyl-CoA acetyltransferase (EC 2.3.1.9)	FIG00000004	isu;CBSS-246196.1.peg.364 icw(2);n-Phenylalkanoic_acid_degradation isu;Ethylmalonyl
NODE_3_length_285037_cov_42.248081	fig|6666666.34159.peg.758	peg	NODE_3_length_285037_cov_42.248081_210383_208635	210383	2E+05	-	Long-chain-fatty-acid--CoA ligase (EC 6.2.1.3)	FIG00018699	icw(1);n-Phenylalkanoic_acid_degradation icw(1);Fatty_acid_metabolism_cluster idu(5);Biotin_synthesis_cluster
NODE_3_length_285037_cov_42.248081	fig|6666666.34159.peg.759	peg	NODE_3_length_285037_cov_42.248081_210640_211818	210640	2E+05	+	Putrescine transport ATP-binding protein PotA (TC 3.A.1.11.1)	FIG00007514	isu;Polyamine_Metabolism
NODE_3_length_285037_cov_42.248081	fig|6666666.34159.peg.760	peg	NODE_3_length_285037_cov_42.248081_211815_212690	211815	2E+05	+	Spermidine Putrescine ABC transporter permease component PotB (TC 3.A.1.11.1)	FIG00004037	icw(2);Polyamine_Metabolism
NODE_3_length_285037_cov_42.248081	fig|6666666.34159.peg.761	peg	NODE_3_length_285037_cov_42.248081_212687_213460	212687	2E+05	+	Spermidine Putrescine ABC transporter permease component potC (TC_3.A.1.11.1)	FIG00003894	icw(1);Polyamine_Metabolism
NODE_3_length_285037_cov_42.248081	fig|6666666.34159.peg.762	peg	NODE_3_length_285037_cov_42.248081_213457_214488	213457	2E+05	+	ABC transporter, periplasmic spermidine putrescine-binding protein PotD (TC 3.A.1.11.1)	FIG00005022	icw(3);Polyamine_Metabolism
NODE_3_length_285037_cov_42.248081	fig|6666666.34159.peg.763	peg	NODE_3_length_285037_cov_42.248081_214609_216372	214609	2E+05	+	ABC-type multidrug transport system, ATPase component		
NODE_3_length_285037_cov_42.248081	fig|6666666.34159.peg.764	peg	NODE_3_length_285037_cov_42.248081_217279_218376	217279	2E+05	+	Beta-lactamase (EC 3.5.2.6)	FIG00000747	isu;Beta-lactamase isu;Tn552
NODE_3_length_285037_cov_42.248081	fig|6666666.34159.peg.765	peg	NODE_3_length_285037_cov_42.248081_218556_219395	218556	2E+05	+	hypothetical protein		
NODE_3_length_285037_cov_42.248081	fig|6666666.34159.peg.766	peg	NODE_3_length_285037_cov_42.248081_221622_219748	221622	2E+05	-	Chaperone protein DnaJ	FIG00000070	icw(1);Heat_shock_dnaK_gene_cluster_extended icw(1);Protein_chaperones
NODE_3_length_285037_cov_42.248081	fig|6666666.34159.peg.767	peg	NODE_3_length_285037_cov_42.248081_222124_221708	222124	2E+05	-	Chaperone protein DnaJ	FIG00000070	icw(1);Heat_shock_dnaK_gene_cluster_extended icw(1);Protein_chaperones
NODE_3_length_285037_cov_42.248081	fig|6666666.34159.rna.13	rna	NODE_3_length_285037_cov_42.248081_222925_222841	222925	2E+05	-	tRNA-Ser-TGA		
NODE_3_length_285037_cov_42.248081	fig|6666666.34159.peg.768	peg	NODE_3_length_285037_cov_42.248081_223070_224464	223070	2E+05	+	unknown protein		
NODE_3_length_285037_cov_42.248081	fig|6666666.34159.peg.769	peg	NODE_3_length_285037_cov_42.248081_226423_224447	226423	2E+05	-	inversin protein alternative isoform, putative		
NODE_3_length_285037_cov_42.248081	fig|6666666.34159.peg.770	peg	NODE_3_length_285037_cov_42.248081_226678_227499	226678	2E+05	+	hypothetical protein		
NODE_3_length_285037_cov_42.248081	fig|6666666.34159.peg.771	peg	NODE_3_length_285037_cov_42.248081_227608_228396	227608	2E+05	+	hypothetical protein		
NODE_3_length_285037_cov_42.248081	fig|6666666.34159.peg.772	peg	NODE_3_length_285037_cov_42.248081_228393_229244	228393	2E+05	+	hypothetical protein	FIG00638284	ff
NODE_3_length_285037_cov_42.248081	fig|6666666.34159.peg.773	peg	NODE_3_length_285037_cov_42.248081_229253_231493	229253	2E+05	+	Helicase PriA essential for oriC/DnaA-independent DNA replication	FIG00080955	ff
NODE_3_length_285037_cov_42.248081	fig|6666666.34159.peg.774	peg	NODE_3_length_285037_cov_42.248081_231689_232300	231689	2E+05	+	uridine kinase(EC:2.7.1.48)		
NODE_3_length_285037_cov_42.248081	fig|6666666.34159.peg.775	peg	NODE_3_length_285037_cov_42.248081_233223_232333	233223	2E+05	-	hypothetical protein		
NODE_3_length_285037_cov_42.248081	fig|6666666.34159.peg.776	peg	NODE_3_length_285037_cov_42.248081_233707_234801	233707	2E+05	+	protein of unknown function DUF1504		
NODE_3_length_285037_cov_42.248081	fig|6666666.34159.peg.777	peg	NODE_3_length_285037_cov_42.248081_234816_235193	234816	2E+05	+	protein of unknown function DUF1504		
NODE_3_length_285037_cov_42.248081	fig|6666666.34159.peg.778	peg	NODE_3_length_285037_cov_42.248081_235589_235245	235589	2E+05	-	hypothetical protein		
NODE_3_length_285037_cov_42.248081	fig|6666666.34159.peg.779	peg	NODE_3_length_285037_cov_42.248081_236412_235714	236412	2E+05	-	putative periplasmic immunogenic protein		
NODE_3_length_285037_cov_42.248081	fig|6666666.34159.peg.780	peg	NODE_3_length_285037_cov_42.248081_236722_237387	236722	2E+05	+	hypothetical protein		
NODE_3_length_285037_cov_42.248081	fig|6666666.34159.peg.781	peg	NODE_3_length_285037_cov_42.248081_237403_238923	237403	2E+05	+	Thermostable carboxypeptidase 1 (EC 3.4.17.19)	FIG00229345	isu;Metallocarboxypeptidases_(EC_3.4.17.-)
NODE_3_length_285037_cov_42.248081	fig|6666666.34159.peg.782	peg	NODE_3_length_285037_cov_42.248081_241169_238920	241169	2E+05	-	hypothetical protein	FIG00638284	ff
NODE_3_length_285037_cov_42.248081	fig|6666666.34159.peg.783	peg	NODE_3_length_285037_cov_42.248081_241407_242780	241407	2E+05	+	hypothetical protein		
NODE_3_length_285037_cov_42.248081	fig|6666666.34159.peg.784	peg	NODE_3_length_285037_cov_42.248081_242847_243173	242847	2E+05	+	hypothetical protein		
NODE_3_length_285037_cov_42.248081	fig|6666666.34159.peg.785	peg	NODE_3_length_285037_cov_42.248081_244927_243170	244927	2E+05	-	hypothetical protein	FIG00638284	ff
NODE_3_length_285037_cov_42.248081	fig|6666666.34159.peg.786	peg	NODE_3_length_285037_cov_42.248081_245093_246658	245093	2E+05	+	4-alpha-glucanotransferase (amylomaltase) (EC 2.4.1.25)	FIG00028461	isu;Glycogen_metabolism isu;Maltose_and_Maltodextrin_Utilization isu;Glycogen_metabolism_cluster
NODE_3_length_285037_cov_42.248081	fig|6666666.34159.peg.787	peg	NODE_3_length_285037_cov_42.248081_246663_248189	246663	2E+05	+	hypothetical protein	FIG00638284	ff
NODE_3_length_285037_cov_42.248081	fig|6666666.34159.peg.788	peg	NODE_3_length_285037_cov_42.248081_248199_248759	248199	2E+05	+	Cob(I)alamin adenosyltransferase PduO (EC 2.5.1.17)	FIG00001045	isu;Glycerol_fermenation_to_1,3-propanediol isu;G3E_family_of_P-loop_GTPases_(metallocenter_biosynthesis)
NODE_3_length_285037_cov_42.248081	fig|6666666.34159.peg.789	peg	NODE_3_length_285037_cov_42.248081_248844_258566	248844	3E+05	+	hypothetical protein	FIG00638284	ff
NODE_3_length_285037_cov_42.248081	fig|6666666.34159.peg.790	peg	NODE_3_length_285037_cov_42.248081_259423_258569	259423	3E+05	-	endonuclease G		
NODE_3_length_285037_cov_42.248081	fig|6666666.34159.peg.791	peg	NODE_3_length_285037_cov_42.248081_259641_259970	259641	3E+05	+	hypothetical protein		
NODE_3_length_285037_cov_42.248081	fig|6666666.34159.peg.792	peg	NODE_3_length_285037_cov_42.248081_260221_261369	260221	3E+05	+	hypothetical protein		
NODE_3_length_285037_cov_42.248081	fig|6666666.34159.peg.793	peg	NODE_3_length_285037_cov_42.248081_261446_262582	261446	3E+05	+	1-deoxy-D-xylulose 5-phosphate reductoisomerase (EC 1.1.1.267)	FIG00000401	isu;Nonmevalonate_Branch_of_Isoprenoid_Biosynthesis isu;Isoprenoid_Biosynthesis
NODE_3_length_285037_cov_42.248081	fig|6666666.34159.peg.794	peg	NODE_3_length_285037_cov_42.248081_262749_264713	262749	3E+05	+	Membrane-associated zinc metalloprotease	FIG00002803	ff
NODE_3_length_285037_cov_42.248081	fig|6666666.34159.peg.795	peg	NODE_3_length_285037_cov_42.248081_264823_265527	264823	3E+05	+	Predicted nucleoside-diphosphate-sugar epimerase		
NODE_3_length_285037_cov_42.248081	fig|6666666.34159.peg.796	peg	NODE_3_length_285037_cov_42.248081_265660_266901	265660	3E+05	+	hypothetical protein	FIG00638284	ff
NODE_3_length_285037_cov_42.248081	fig|6666666.34159.peg.797	peg	NODE_3_length_285037_cov_42.248081_268554_266908	268554	3E+05	-	Pyruvate decarboxylase (EC 4.1.1.1); Alpha-keto-acid decarboxylase (EC 4.1.1.-)	FIG00001998	isu;Pyruvate_metabolism_II:_acetyl-CoA,_acetogenesis_from_pyruvate
NODE_3_length_285037_cov_42.248081	fig|6666666.34159.rna.14	rna	NODE_3_length_285037_cov_42.248081_268661_268733	268661	3E+05	+	tRNA-Arg-TCT		
NODE_3_length_285037_cov_42.248081	fig|6666666.34159.peg.798	peg	NODE_3_length_285037_cov_42.248081_269804_268773	269804	3E+05	-	hypothetical protein		
NODE_3_length_285037_cov_42.248081	fig|6666666.34159.peg.799	peg	NODE_3_length_285037_cov_42.248081_269918_272530	269918	3E+05	+	unknown protein	FIG00764722	ff
NODE_3_length_285037_cov_42.248081	fig|6666666.34159.peg.800	peg	NODE_3_length_285037_cov_42.248081_272815_272564	272815	3E+05	-	KH domain RNA binding protein YlqC	FIG00005628	idu(1);KH_domain_RNA_binding_protein_YlqC
NODE_3_length_285037_cov_42.248081	fig|6666666.34159.peg.801	peg	NODE_3_length_285037_cov_42.248081_273587_273150	273587	3E+05	-	D-tyrosyl-tRNA(Tyr) deacylase	FIG00000473	isu;D-tyrosyl-tRNA(Tyr)_deacylase isu;CBSS-342610.3.peg.283
NODE_3_length_285037_cov_42.248081	fig|6666666.34159.peg.802	peg	NODE_3_length_285037_cov_42.248081_274537_273578	274537	3E+05	-	Ribosomal large subunit pseudouridine synthase D (EC 4.2.1.70)	FIG00000124	idu(1);RNA_pseudouridine_syntheses
NODE_3_length_285037_cov_42.248081	fig|6666666.34159.peg.803	peg	NODE_3_length_285037_cov_42.248081_274928_274641	274928	3E+05	-	unknown protein	FIG00764722	ff
NODE_3_length_285037_cov_42.248081	fig|6666666.34159.peg.804	peg	NODE_3_length_285037_cov_42.248081_275241_274966	275241	3E+05	-	hypothetical protein		
NODE_3_length_285037_cov_42.248081	fig|6666666.34159.peg.805	peg	NODE_3_length_285037_cov_42.248081_275400_276203	275400	3E+05	+	Non-heme chloroperoxidase (EC 1.11.1.10)	FIG01225742	ff
NODE_3_length_285037_cov_42.248081	fig|6666666.34159.peg.806	peg	NODE_3_length_285037_cov_42.248081_276236_276877	276236	3E+05	+	DNA repair protein RecO		
NODE_3_length_285037_cov_42.248081	fig|6666666.34159.rna.15	rna	NODE_3_length_285037_cov_42.248081_276936_277019	276936	3E+05	+	tRNA-Leu-GAG		isu;tRNAs
NODE_3_length_285037_cov_42.248081	fig|6666666.34159.peg.807	peg	NODE_3_length_285037_cov_42.248081_277201_277893	277201	3E+05	+	hypothetical protein	FIG00638284	ff
NODE_3_length_285037_cov_42.248081	fig|6666666.34159.peg.808	peg	NODE_3_length_285037_cov_42.248081_277890_278099	277890	3E+05	+	Protein YidD	FIG00004843	isu;RNA_modification_cluster
NODE_3_length_285037_cov_42.248081	fig|6666666.34159.peg.809	peg	NODE_3_length_285037_cov_42.248081_278183_279385	278183	3E+05	+	oxidoreductase, 2OG-Fe(II) oxygenase family		
NODE_3_length_285037_cov_42.248081	fig|6666666.34159.peg.810	peg	NODE_3_length_285037_cov_42.248081_280363_279425	280363	3E+05	-	hypothetical protein		

NODE_3_length_285037_cov_42.248081	fig|6666666.34159.peg.811	peg	NODE_3_length_285037_cov_42.248081_280563_281360	280563	3E+05	+	ABC-type multidrug transport system, ATPase component	FIG00001005	ff
NODE_3_length_285037_cov_42.248081	fig|6666666.34159.peg.812	peg	NODE_3_length_285037_cov_42.248081_281317_282135	281317	3E+05	+	ABC-type drug export system, membrane protein	FIG00822310	ff
NODE_3_length_285037_cov_42.248081	fig|6666666.34159.peg.813	peg	NODE_3_length_285037_cov_42.248081_283403_282174	283403	3E+05	-	hypothetical protein		
NODE_3_length_285037_cov_42.248081	fig|6666666.34159.peg.814	peg	NODE_3_length_285037_cov_42.248081_284699_283419	284699	3E+05	-	hypothetical protein		
NODE_41_length_192097_cov_42.719078	fig|6666666.34159.peg.815	peg	NODE_41_length_192097_cov_42.719078_350_574	350	574	+	Mobile element protein	FIG01306568	ff
NODE_41_length_192097_cov_42.719078	fig|6666666.34159.peg.816	peg	NODE_41_length_192097_cov_42.719078_3039_874	3039	874	-	ATP-dependent DNA helicase UvrD/PcrA	FIG00000372	idu(1);CBSS-393121.3.peg.1913 idu(1);DNA_repair,_bacterial_UvrD_and_related_helicases
NODE_41_length_192097_cov_42.719078	fig|6666666.34159.peg.817	peg	NODE_41_length_192097_cov_42.719078_6717_3112	6717	3112	-	Proline dehydrogenase (EC 1.5.99.8) (Proline oxidase) / Delta-1-pyrroline-5-carboxylate dehydrogenase (EC 1.5.1.12)	FIG00001101	isu;Respiratory_dehydrogenases_1 isu;CBSS-196620.1.peg.2477 isu;Proline,_4-hydroxyproline_uptake_and_utilization
NODE_41_length_192097_cov_42.719078	fig|6666666.34159.peg.818	peg	NODE_41_length_192097_cov_42.719078_8173_6857	8173	6857	-	hypothetical protein		
NODE_41_length_192097_cov_42.719078	fig|6666666.34159.peg.819	peg	NODE_41_length_192097_cov_42.719078_8447_9070	8447	9070	+	Phosphoheptose isomerase (EC 5.3.1.-)	FIG00000620	isu;LOS_core_oligosaccharide_biosynthesis isu;Capsular_heptose_biosynthesis
NODE_41_length_192097_cov_42.719078	fig|6666666.34159.peg.820	peg	NODE_41_length_192097_cov_42.719078_9173_10291	9173	10291	+	Tetraacyldisaccharide 4'-kinase (EC 2.7.1.130)	FIG00138202	isu;KDO2-Lipid_A_biosynthesis
NODE_41_length_192097_cov_42.719078	fig|6666666.34159.peg.821	peg	NODE_41_length_192097_cov_42.719078_10324_11556	10324	11556	+	Proton/glutamate symport protein @ Sodium/glutamate symport protein	FIG00008055	idu(2);Glutamate_and_Aspartate_uptake_in_Bacteria
NODE_41_length_192097_cov_42.719078	fig|6666666.34159.peg.822	peg	NODE_41_length_192097_cov_42.719078_11645_11977	11645	11977	+	hypothetical protein		
NODE_41_length_192097_cov_42.719078	fig|6666666.34159.peg.823	peg	NODE_41_length_192097_cov_42.719078_12746_14581	12746	14581	+	Glutamate synthase [NADPH] small chain (EC 1.4.1.13)	FIG00007957	isu;Glutamine,_Glutamate,_Aspartate_and_Asparagine_Biosynthesis
NODE_41_length_192097_cov_42.719078	fig|6666666.34159.peg.824	peg	NODE_41_length_192097_cov_42.719078_14583_14714	14583	14714	+	hypothetical protein		
NODE_41_length_192097_cov_42.719078	fig|6666666.34159.peg.825	peg	NODE_41_length_192097_cov_42.719078_14720_15232	14720	15232	+	Ubiquinol-cytochrome C reductase iron-sulfur subunit (EC 1.10.2.2)	FIG00000797	isu;Ubiquinone_Menaquinone-cytochrome_c_reductase_complexes
NODE_41_length_192097_cov_42.719078	fig|6666666.34159.peg.826	peg	NODE_41_length_192097_cov_42.719078_15261_16586	15261	16586	+	Ubiquinol--cytochrome c reductase, cytochrome B subunit (EC 1.10.2.2)	FIG00000754	icw(1);Ubiquinone_Menaquinone-cytochrome_c_reductase_complexes
NODE_41_length_192097_cov_42.719078	fig|6666666.34159.peg.827	peg	NODE_41_length_192097_cov_42.719078_16605_20711	16605	20711	+	hypothetical protein	FIG00638284	ff
NODE_41_length_192097_cov_42.719078	fig|6666666.34159.peg.828	peg	NODE_41_length_192097_cov_42.719078_20708_22165	20708	22165	+	Cytochrome c oxidase subunit CcoN (EC 1.9.3.1)	FIG00103126	icw(1);Terminal_cytochrome_C_oxidases
NODE_41_length_192097_cov_42.719078	fig|6666666.34159.peg.829	peg	NODE_41_length_192097_cov_42.719078_22162_23442	22162	23442	+	Cytochrome c oxidase subunit CcoO (EC 1.9.3.1)	FIG00001108	isu;Terminal_cytochrome_C_oxidases
NODE_41_length_192097_cov_42.719078	fig|6666666.34159.peg.830	peg	NODE_41_length_192097_cov_42.719078_23439_23672	23439	23672	+	hypothetical protein		
NODE_41_length_192097_cov_42.719078	fig|6666666.34159.peg.831	peg	NODE_41_length_192097_cov_42.719078_23662_23937	23662	23937	+	hypothetical protein		
NODE_41_length_192097_cov_42.719078	fig|6666666.34159.peg.832	peg	NODE_41_length_192097_cov_42.719078_24435_23953	24435	23953	-	hypothetical protein		
NODE_41_length_192097_cov_42.719078	fig|6666666.34159.peg.833	peg	NODE_41_length_192097_cov_42.719078_24687_25712	24687	25712	+	hypothetical protein		
NODE_41_length_192097_cov_42.719078	fig|6666666.34159.peg.834	peg	NODE_41_length_192097_cov_42.719078_25895_26251	25895	26251	+	NADH ubiquinone oxidoreductase chain A (EC 1.6.5.3)	FIG00000558	icw(4);Respiratory_Complex_I icw(4);NADH_ubiquinone_oxidoreductase
NODE_41_length_192097_cov_42.719078	fig|6666666.34159.peg.835	peg	NODE_41_length_192097_cov_42.719078_26242_26715	26242	26715	+	NADH-ubiquinone oxidoreductase chain B (EC 1.6.5.3)	FIG00133539	icw(1);Respiratory_Complex_I icw(1);NADH_ubiquinone_oxidoreductase
NODE_41_length_192097_cov_42.719078	fig|6666666.34159.peg.836	peg	NODE_41_length_192097_cov_42.719078_26696_27223	26696	27223	+	NADH-ubiquinone oxidoreductase chain C (EC 1.6.5.3)	FIG00000785	icw(4);Respiratory_Complex_I icw(4);NADH_ubiquinone_oxidoreductase
NODE_41_length_192097_cov_42.719078	fig|6666666.34159.peg.837	peg	NODE_41_length_192097_cov_42.719078_27198_28403	27198	28403	+	NADH-ubiquinone oxidoreductase chain D (EC 1.6.5.3)	FIG00138449	icw(6);Respiratory_Complex_I icw(6);NADH_ubiquinone_oxidoreductase
NODE_41_length_192097_cov_42.719078	fig|6666666.34159.peg.838	peg	NODE_41_length_192097_cov_42.719078_28403_28918	28403	28918	+	NADH-ubiquinone oxidoreductase chain E (EC 1.6.5.3)	FIG00000705	icw(1);Respiratory_Complex_I icw(1);NADH_ubiquinone_oxidoreductase
NODE_41_length_192097_cov_42.719078	fig|6666666.34159.peg.839	peg	NODE_41_length_192097_cov_42.719078_28911_30209	28911	30209	+	NADH-ubiquinone oxidoreductase chain F (EC 1.6.5.3)	FIG00117890	icw(5);Respiratory_Complex_I icw(5);NADH_ubiquinone_oxidoreductase
NODE_41_length_192097_cov_42.719078	fig|6666666.34159.peg.840	peg	NODE_41_length_192097_cov_42.719078_30217_32532	30217	32532	+	NADH-ubiquinone oxidoreductase chain G (EC 1.6.5.3)	FIG00010922	icw(8);Respiratory_Complex_I icw(8);NADH_ubiquinone_oxidoreductase
NODE_41_length_192097_cov_42.719078	fig|6666666.34159.peg.841	peg	NODE_41_length_192097_cov_42.719078_32535_33512	32535	33512	+	NADH-ubiquinone oxidoreductase chain H (EC 1.6.5.3)	FIG00133041	icw(3);Respiratory_Complex_I icw(3);NADH_ubiquinone_oxidoreductase
NODE_41_length_192097_cov_42.719078	fig|6666666.34159.peg.842	peg	NODE_41_length_192097_cov_42.719078_33514_33987	33514	33987	+	NADH-ubiquinone oxidoreductase chain I (EC 1.6.5.3)	FIG00138210	icw(4);Respiratory_Complex_I icw(4);NADH_ubiquinone_oxidoreductase
NODE_41_length_192097_cov_42.719078	fig|6666666.34159.peg.843	peg	NODE_41_length_192097_cov_42.719078_33994_34500	33994	34500	+	NADH-ubiquinone oxidoreductase chain J (EC 1.6.5.3)	FIG00000569	icw(2);Respiratory_Complex_I icw(2);NADH_ubiquinone_oxidoreductase
NODE_41_length_192097_cov_42.719078	fig|6666666.34159.peg.844	peg	NODE_41_length_192097_cov_42.719078_34500_34799	34500	34799	+	NADH-ubiquinone oxidoreductase chain K (EC 1.6.5.3)	FIG00000559	isu;Respiratory_Complex_I isu;NADH_ubiquinone_oxidoreductase
NODE_41_length_192097_cov_42.719078	fig|6666666.34159.peg.845	peg	NODE_41_length_192097_cov_42.719078_34801_36672	34801	36672	+	NADH-ubiquinone oxidoreductase chain L (EC 1.6.5.3)	FIG00068727	icw(1);Respiratory_Complex_I icw(1);NADH_ubiquinone_oxidoreductase
NODE_41_length_192097_cov_42.719078	fig|6666666.34159.peg.846	peg	NODE_41_length_192097_cov_42.719078_36681_38138	36681	38138	+	NADH-ubiquinone oxidoreductase chain M (EC 1.6.5.3)	FIG00138227	icw(6);Respiratory_Complex_I icw(6);NADH_ubiquinone_oxidoreductase
NODE_41_length_192097_cov_42.719078	fig|6666666.34159.peg.847	peg	NODE_41_length_192097_cov_42.719078_38135_39574	38135	39574	+	NADH-ubiquinone oxidoreductase chain N (EC 1.6.5.3)	FIG00000512	icw(3);Respiratory_Complex_I icw(3);NADH_ubiquinone_oxidoreductase
NODE_41_length_192097_cov_42.719078	fig|6666666.34159.peg.848	peg	NODE_41_length_192097_cov_42.719078_39887_40744	39887	40744	+	hypothetical protein		
NODE_41_length_192097_cov_42.719078	fig|6666666.34159.peg.849	peg	NODE_41_length_192097_cov_42.719078_40794_40970	40794	40970	+	hypothetical protein		
NODE_41_length_192097_cov_42.719078	fig|6666666.34159.peg.850	peg	NODE_41_length_192097_cov_42.719078_41199_41471	41199	41471	+	hypothetical protein		
NODE_41_length_192097_cov_42.719078	fig|6666666.34159.peg.851	peg	NODE_41_length_192097_cov_42.719078_41663_42697	41663	42697	+	hypothetical protein		
NODE_41_length_192097_cov_42.719078	fig|6666666.34159.peg.852	peg	NODE_41_length_192097_cov_42.719078_42955_43701	42955	43701	+	hypothetical protein		
NODE_41_length_192097_cov_42.719078	fig|6666666.34159.peg.853	peg	NODE_41_length_192097_cov_42.719078_43784_43954	43784	43954	+	hypothetical protein		
NODE_41_length_192097_cov_42.719078	fig|6666666.34159.peg.854	peg	NODE_41_length_192097_cov_42.719078_44152_44535	44152	44535	+	hypothetical protein		
NODE_41_length_192097_cov_42.719078	fig|6666666.34159.peg.855	peg	NODE_41_length_192097_cov_42.719078_44635_45204	44635	45204	+	Translation elongation factor P	FIG00000177	icw(1);Translation_elongation_factor_P_lysylation isu;Translation_elongation_factors_bacterial isu;Modification_of_eukaryotic_initiation_factor
NODE_41_length_192097_cov_42.719078	fig|6666666.34159.peg.856	peg	NODE_41_length_192097_cov_42.719078_45214_46158	45214	46158	+	Translation elongation factor P Lys34:lysine transferase	FIG00587265	isu;Translation_elongation_factor_P_lysylation
NODE_41_length_192097_cov_42.719078	fig|6666666.34159.peg.857	peg	NODE_41_length_192097_cov_42.719078_46408_46130	46408	46130	-	hypothetical protein		
NODE_41_length_192097_cov_42.719078	fig|6666666.34159.peg.858	peg	NODE_41_length_192097_cov_42.719078_48093_46531	48093	46531	-	N-methylhydantoinase (ATP-hydrolyzing) (EC 3.5.2.14)	FIG00002273	isu;Creatine_and_Creatinine_Degradation isu;Hydantoin_metabolism
NODE_41_length_192097_cov_42.719078	fig|6666666.34159.peg.859	peg	NODE_41_length_192097_cov_42.719078_49189_48104	49189	48104	-	hypothetical protein		
NODE_41_length_192097_cov_42.719078	fig|6666666.34159.peg.860	peg	NODE_41_length_192097_cov_42.719078_50381_49170	50381	49170	-	Cytosine/purine/uracil/thiamine/allantoin permease family protein	FIG00003506	isu;Purine_Utilization
NODE_41_length_192097_cov_42.719078	fig|6666666.34159.peg.861	peg	NODE_41_length_192097_cov_42.719078_50775_51629	50775	51629	+	Glucose-1-phosphate cytidylyltransferase (EC 2.7.7.33)	FIG00132976	isu;dTDP-rhamnose_synthesis
NODE_41_length_192097_cov_42.719078	fig|6666666.34159.peg.862	peg	NODE_41_length_192097_cov_42.719078_51626_52759	51626	52759	+	Similar to CDP-glucose 4,6-dehydratase (EC 4.2.1.45)	FIG00473043	ff
NODE_41_length_192097_cov_42.719078	fig|6666666.34159.peg.863	peg	NODE_41_length_192097_cov_42.719078_52759_53316	52759	53316	+	dTDP-4-dehydrorhamnose 3,5-epimerase (EC 5.1.3.13)	FIG00000408	icw(1);dTDP-rhamnose_synthesis idu(1);Rhamnose_containing_glycans idu(1);Capsular_heptose_biosynthesis
NODE_41_length_192097_cov_42.719078	fig|6666666.34159.peg.864	peg	NODE_41_length_192097_cov_42.719078_53318_54262	53318	54262	+	hypothetical protein	FIG00638284	ff
NODE_41_length_192097_cov_42.719078	fig|6666666.34159.peg.865	peg	NODE_41_length_192097_cov_42.719078_55773_55892	55773	55892	+	hypothetical protein		
NODE_41_length_192097_cov_42.719078	fig|6666666.34159.peg.866	peg	NODE_41_length_192097_cov_42.719078_55906_56895	55906	56895	+	hypothetical protein		
NODE_41_length_192097_cov_42.719078	fig|6666666.34159.peg.867	peg	NODE_41_length_192097_cov_42.719078_56916_57614	56916	57614	+	peptidyl-prolyl cis-trans isomerase Mip		
NODE_41_length_192097_cov_42.719078	fig|6666666.34159.peg.868	peg	NODE_41_length_192097_cov_42.719078_57895_58089	57895	58089	+	hypothetical protein		
NODE_41_length_192097_cov_42.719078	fig|6666666.34159.peg.869	peg	NODE_41_length_192097_cov_42.719078_58086_58226	58086	58226	+	hypothetical protein		

NODE_41_length_192097_cov_42.719078	fig|6666666.34159.peg.870	peg	NODE_41_length_192097_cov_42.719078_58746_59480	58746	59480	+	hypothetical protein		
NODE_41_length_192097_cov_42.719078	fig|6666666.34159.peg.871	peg	NODE_41_length_192097_cov_42.719078_60210_59674	60210	59674	-	hypothetical protein		
NODE_41_length_192097_cov_42.719078	fig|6666666.34159.peg.872	peg	NODE_41_length_192097_cov_42.719078_63147_60400	63147	60400	-	hypothetical protein	FIG00638284	ff
NODE_41_length_192097_cov_42.719078	fig|6666666.34159.peg.873	peg	NODE_41_length_192097_cov_42.719078_63415_65106	63415	65106	+	Potassium-transporting ATPase A chain (EC 3.6.3.12) (TC 3.A.3.7.1)	FIG00002960	isu;Potassium_homeostasis
NODE_41_length_192097_cov_42.719078	fig|6666666.34159.peg.874	peg	NODE_41_length_192097_cov_42.719078_65116_67209	65116	67209	+	Potassium-transporting ATPase B chain (EC 3.6.3.12) (TC 3.A.3.7.1)	FIG00046742	icw(1);Potassium_homeostasis
NODE_41_length_192097_cov_42.719078	fig|6666666.34159.peg.875	peg	NODE_41_length_192097_cov_42.719078_67212_67802	67212	67802	+	Potassium-transporting ATPase C chain (EC 3.6.3.12) (TC 3.A.3.7.1)	FIG00007507	icw(2);Potassium_homeostasis
NODE_41_length_192097_cov_42.719078	fig|6666666.34159.peg.876	peg	NODE_41_length_192097_cov_42.719078_67795_70425	67795	70425	+	Osmosensitive K+ channel histidine kinase KdpD (EC 2.7.3.-)	FIG00000846	icw(3);Potassium_homeostasis
NODE_41_length_192097_cov_42.719078	fig|6666666.34159.peg.877	peg	NODE_41_length_192097_cov_42.719078_70516_71994	70516	71994	+	hypothetical protein	FIG00638284	ff
NODE_41_length_192097_cov_42.719078	fig|6666666.34159.peg.878	peg	NODE_41_length_192097_cov_42.719078_71991_73250	71991	73250	+	probable multidrug resistance protein, emrA		
NODE_41_length_192097_cov_42.719078	fig|6666666.34159.peg.879	peg	NODE_41_length_192097_cov_42.719078_73240_74787	73240	74787	+	Inner membrane component of tripartite multidrug resistance system	FIG00005204	ff
NODE_41_length_192097_cov_42.719078	fig|6666666.34159.peg.880	peg	NODE_41_length_192097_cov_42.719078_74886_76403	74886	76403	+	Prolyl-tRNA synthetase (EC 6.1.1.15)	FIG00000069	ff
NODE_41_length_192097_cov_42.719078	fig|6666666.34159.peg.881	peg	NODE_41_length_192097_cov_42.719078_76728_76405	76728	76405	-	hypothetical protein		
NODE_41_length_192097_cov_42.719078	fig|6666666.34159.peg.882	peg	NODE_41_length_192097_cov_42.719078_76801_77196	76801	77196	+	hypothetical protein		
NODE_41_length_192097_cov_42.719078	fig|6666666.34159.peg.883	peg	NODE_41_length_192097_cov_42.719078_77871_77215	77871	77215	-	hypothetical protein		
NODE_41_length_192097_cov_42.719078	fig|6666666.34159.peg.884	peg	NODE_41_length_192097_cov_42.719078_78101_88345	78101	88345	+	hypothetical protein	FIG00638284	ff
NODE_41_length_192097_cov_42.719078	fig|6666666.34159.peg.885	peg	NODE_41_length_192097_cov_42.719078_89625_88408	89625	88408	-	putative tyrosine/tryptophan transport protein		
NODE_41_length_192097_cov_42.719078	fig|6666666.34159.peg.886	peg	NODE_41_length_192097_cov_42.719078_91372_89924	91372	89924	-	Catalase (EC 1.11.1.6)	FIG00000313	isu;Oxidative_stress isu;Protection_from_Reactive_Oxygen_Species
NODE_41_length_192097_cov_42.719078	fig|6666666.34159.peg.887	peg	NODE_41_length_192097_cov_42.719078_93210_91450	93210	91450	-	Arginyl-tRNA synthetase (EC 6.1.1.19)	FIG00000088	isu;tRNA_aminoacylation,_Arg
NODE_41_length_192097_cov_42.719078	fig|6666666.34159.peg.888	peg	NODE_41_length_192097_cov_42.719078_94161_93229	94161	93229	-	hypothetical protein	FIG00638284	ff
NODE_41_length_192097_cov_42.719078	fig|6666666.34159.peg.889	peg	NODE_41_length_192097_cov_42.719078_95153_94167	95153	94167	-	Lipoate synthase	FIG00000362	isu;Lipoic_acid_metabolism
NODE_41_length_192097_cov_42.719078	fig|6666666.34159.peg.890	peg	NODE_41_length_192097_cov_42.719078_96569_95157	96569	95157	-	Dihydrolipoamide dehydrogenase of pyruvate dehydrogenase complex (EC 1.8.1.4)	FIG01303880	isu;Pyruvate_metabolism_II:_acetyl-CoA,_acetogenesis_from_pyruvate isu;TCA_Cycle
NODE_41_length_192097_cov_42.719078	fig|6666666.34159.peg.891	peg	NODE_41_length_192097_cov_42.719078_97358_96678	97358	96678	-	hypothetical protein		
NODE_41_length_192097_cov_42.719078	fig|6666666.34159.peg.892	peg	NODE_41_length_192097_cov_42.719078_97454_97924	97454	97924	+	hypothetical protein	FIG00638284	ff
NODE_41_length_192097_cov_42.719078	fig|6666666.34159.peg.893	peg	NODE_41_length_192097_cov_42.719078_98046_98597	98046	98597	+	hypothetical protein		
NODE_41_length_192097_cov_42.719078	fig|6666666.34159.peg.894	peg	NODE_41_length_192097_cov_42.719078_98713_99459	98713	99459	+	hypothetical protein		
NODE_41_length_192097_cov_42.719078	fig|6666666.34159.peg.895	peg	NODE_41_length_192097_cov_42.719078_100894_99512	100894	99512	-	Catalyzes the cleavage of p-aminobenzoyl-glutamate to p-aminobenzoate and glutamate, subunit A	FIG00001384	ff
NODE_41_length_192097_cov_42.719078	fig|6666666.34159.peg.896	peg	NODE_41_length_192097_cov_42.719078_102700_100895	102700	1E+05	-	hypothetical protein	FIG00638284	ff
NODE_41_length_192097_cov_42.719078	fig|6666666.34159.peg.897	peg	NODE_41_length_192097_cov_42.719078_105159_102754	105159	1E+05	-	hypothetical protein	FIG00638284	ff
NODE_41_length_192097_cov_42.719078	fig|6666666.34159.peg.898	peg	NODE_41_length_192097_cov_42.719078_106207_105230	106207	1E+05	-	MoxR-like ATPase in aerotolerance operon	FIG00002600	isu;Aerotolerance_operon_in_Bacteroides_and_potentially_orthologous_operons_in_other_organisms
NODE_41_length_192097_cov_42.719078	fig|6666666.34159.peg.899	peg	NODE_41_length_192097_cov_42.719078_106538_106302	106538	1E+05	-	hypothetical protein		
NODE_41_length_192097_cov_42.719078	fig|6666666.34159.peg.900	peg	NODE_41_length_192097_cov_42.719078_107723_106626	107723	1E+05	-	Fructose-bisphosphate aldolase class I (EC 4.1.2.13)	FIG01262685	isu;Calvin-Benson_cycle isu;Unknown_carbohydrate_utilization_(_cluster_Veg_) isu;Glycolysis_and_Gluconeogenesis
NODE_41_length_192097_cov_42.719078	fig|6666666.34159.peg.901	peg	NODE_41_length_192097_cov_42.719078_108339_109712	108339	1E+05	+	GTPase and tRNA-U34 5-formylation enzyme TrmE	FIG00000279	isu;RNA_modification_and_chromosome_partitioning_cluster isu;RNA_modification_cluster isu;tRNA_modification_Bacteria
NODE_41_length_192097_cov_42.719078	fig|6666666.34159.peg.902	peg	NODE_41_length_192097_cov_42.719078_109714_110334	109714	1E+05	+	Endonuclease III (EC 4.2.99.18)	FIG00000290	isu;DNA_Repair_Base_Excision isu;Control_of_cell_elongation_-_division_cycle_in_Bacilli
NODE_41_length_192097_cov_42.719078	fig|6666666.34159.peg.903	peg	NODE_41_length_192097_cov_42.719078_111191_110667	111191	1E+05	-	hypothetical protein		
NODE_41_length_192097_cov_42.719078	fig|6666666.34159.peg.904	peg	NODE_41_length_192097_cov_42.719078_111483_111193	111483	1E+05	-	hypothetical protein	FIG00638284	ff
NODE_41_length_192097_cov_42.719078	fig|6666666.34159.peg.905	peg	NODE_41_length_192097_cov_42.719078_112258_112404	112258	1E+05	+	hypothetical protein		
NODE_41_length_192097_cov_42.719078	fig|6666666.34159.peg.906	peg	NODE_41_length_192097_cov_42.719078_112419_112547	112419	1E+05	+	hypothetical protein		
NODE_41_length_192097_cov_42.719078	fig|6666666.34159.peg.907	peg	NODE_41_length_192097_cov_42.719078_112907_112608	112907	1E+05	-	hypothetical protein		
NODE_41_length_192097_cov_42.719078	fig|6666666.34159.peg.908	peg	NODE_41_length_192097_cov_42.719078_113220_114404	113220	1E+05	+	hypothetical protein		
NODE_41_length_192097_cov_42.719078	fig|6666666.34159.peg.909	peg	NODE_41_length_192097_cov_42.719078_114633_116012	114633	1E+05	+	Phosphoglucosamine mutase (EC 5.4.2.10)	FIG00009682	icw(1);Sialic_Acid_Metabolism icw(1);UDP-N-acetylmuramate_from_Fructose-6-phosphate_Biosynthesis
NODE_41_length_192097_cov_42.719078	fig|6666666.34159.peg.910	peg	NODE_41_length_192097_cov_42.719078_116022_117851	116022	1E+05	+	Glucosamine--fructose-6-phosphate aminotransferase [isomerizing] (EC 2.6.1.16)	FIG00000038	isu;Inteins isu;Sialic_Acid_Metabolism isu;UDP-N-acetylmuramate_from_Fructose-6-phosphate_Biosynthesis
NODE_41_length_192097_cov_42.719078	fig|6666666.34159.peg.911	peg	NODE_41_length_192097_cov_42.719078_118922_117987	118922	1E+05	-	hypothetical protein		
NODE_41_length_192097_cov_42.719078	fig|6666666.34159.peg.912	peg	NODE_41_length_192097_cov_42.719078_119118_119297	119118	1E+05	+	hypothetical protein		
NODE_41_length_192097_cov_42.719078	fig|6666666.34159.peg.913	peg	NODE_41_length_192097_cov_42.719078_119603_119731	119603	1E+05	+	hypothetical protein		
NODE_41_length_192097_cov_42.719078	fig|6666666.34159.peg.914	peg	NODE_41_length_192097_cov_42.719078_119948_121885	119948	1E+05	+	outer membrane protein RomA		
NODE_41_length_192097_cov_42.719078	fig|6666666.34159.peg.915	peg	NODE_41_length_192097_cov_42.719078_122439_121960	122439	1E+05	-	hypothetical protein		
NODE_41_length_192097_cov_42.719078	fig|6666666.34159.peg.916	peg	NODE_41_length_192097_cov_42.719078_123999_122548	123999	1E+05	-	Replicative DNA helicase (EC 3.6.1.-)	FIG00061313	ff
NODE_41_length_192097_cov_42.719078	fig|6666666.34159.peg.917	peg	NODE_41_length_192097_cov_42.719078_124425_124012	124425	1E+05	-	Endoribonuclease L-PSP	FIG00002122	isu;CBSS-176299.4.peg.1996A
NODE_41_length_192097_cov_42.719078	fig|6666666.34159.peg.918	peg	NODE_41_length_192097_cov_42.719078_126998_124428	126998	1E+05	-	hypothetical protein		
NODE_41_length_192097_cov_42.719078	fig|6666666.34159.peg.919	peg	NODE_41_length_192097_cov_42.719078_128236_127028	128236	1E+05	-	hypothetical protein	FIG00638284	ff
NODE_41_length_192097_cov_42.719078	fig|6666666.34159.peg.920	peg	NODE_41_length_192097_cov_42.719078_128513_130504	128513	1E+05	+	hypothetical protein	FIG00638284	ff
NODE_41_length_192097_cov_42.719078	fig|6666666.34159.peg.921	peg	NODE_41_length_192097_cov_42.719078_130966_130547	130966	1E+05	-	unknown protein	FIG00764722	ff
NODE_41_length_192097_cov_42.719078	fig|6666666.34159.peg.922	peg	NODE_41_length_192097_cov_42.719078_131184_132566	131184	1E+05	+	FIG00494291: hypothetical protein	FIG00493263	ff
NODE_41_length_192097_cov_42.719078	fig|6666666.34159.peg.923	peg	NODE_41_length_192097_cov_42.719078_132604_135162	132604	1E+05	+	General secretion pathway protein D	FIG00001810	ff
NODE_41_length_192097_cov_42.719078	fig|6666666.34159.peg.924	peg	NODE_41_length_192097_cov_42.719078_135152_136786	135152	1E+05	+	General secretion pathway protein E / Type II secretory pathway, ATPase PulE/Tfp pilus assembly pathway, ATPase PilB	FIG00229405	ff
NODE_41_length_192097_cov_42.719078	fig|6666666.34159.peg.925	peg	NODE_41_length_192097_cov_42.719078_136798_137982	136798	1E+05	+	General secretion pathway protein F		
NODE_41_length_192097_cov_42.719078	fig|6666666.34159.peg.926	peg	NODE_41_length_192097_cov_42.719078_137998_138372	137998	1E+05	+	FIG00899507: hypothetical protein	FIG00899506	ff
NODE_41_length_192097_cov_42.719078	fig|6666666.34159.peg.927	peg	NODE_41_length_192097_cov_42.719078_138490_138374	138490	1E+05	-	hypothetical protein		
NODE_41_length_192097_cov_42.719078	fig|6666666.34159.peg.928	peg	NODE_41_length_192097_cov_42.719078_138447_139079	138447	1E+05	+	hypothetical protein		

NODE_41_length_192097_cov_42.719078	fig|6666666.34159.peg.929	peg	NODE_41_length_192097_cov_42.719078_139083_139580	139083	1E+05	+	hypothetical protein		
NODE_41_length_192097_cov_42.719078	fig|6666666.34159.peg.930	peg	NODE_41_length_192097_cov_42.719078_139586_140320	139586	1E+05	+	hypothetical protein	FIG00638284	ff
NODE_41_length_192097_cov_42.719078	fig|6666666.34159.peg.931	peg	NODE_41_length_192097_cov_42.719078_140368_141195	140368	1E+05	+	hypothetical protein		
NODE_41_length_192097_cov_42.719078	fig|6666666.34159.peg.932	peg	NODE_41_length_192097_cov_42.719078_142119_141199	142119	1E+05	-	hypothetical protein		
NODE_41_length_192097_cov_42.719078	fig|6666666.34159.peg.933	peg	NODE_41_length_192097_cov_42.719078_142838_142969	142838	1E+05	+	hypothetical protein		
NODE_41_length_192097_cov_42.719078	fig|6666666.34159.peg.934	peg	NODE_41_length_192097_cov_42.719078_143031_143897	143031	1E+05	+	hypothetical protein		
NODE_41_length_192097_cov_42.719078	fig|6666666.34159.peg.935	peg	NODE_41_length_192097_cov_42.719078_143894_145129	143894	1E+05	+	hypothetical protein	FIG00638284	ff
NODE_41_length_192097_cov_42.719078	fig|6666666.34159.peg.936	peg	NODE_41_length_192097_cov_42.719078_146401_146282	146401	1E+05	-	hypothetical protein		
NODE_41_length_192097_cov_42.719078	fig|6666666.34159.peg.937	peg	NODE_41_length_192097_cov_42.719078_146763_146647	146763	1E+05	-	hypothetical protein		
NODE_41_length_192097_cov_42.719078	fig|6666666.34159.peg.938	peg	NODE_41_length_192097_cov_42.719078_146985_149009	146985	1E+05	+	Phage tail fiber protein	FIG00003520	idu(1);Phage_tail_fiber_proteins
NODE_41_length_192097_cov_42.719078	fig|6666666.34159.peg.939	peg	NODE_41_length_192097_cov_42.719078_150373_149006	150373	1E+05	-	N-acetylglucosamine-1-phosphate uridyltransferase eukaryotic (EC 2.7.7.23)	FIG00004620	isu;UDP-N-acetylmuramate_from_Fructose-6-phosphate_Biosynthesis
NODE_41_length_192097_cov_42.719078	fig|6666666.34159.peg.940	peg	NODE_41_length_192097_cov_42.719078_150859_150996	150859	2E+05	+	hypothetical protein		
NODE_41_length_192097_cov_42.719078	fig|6666666.34159.peg.941	peg	NODE_41_length_192097_cov_42.719078_151128_152582	151128	2E+05	+	RNA methyltransferase, TrmA family	FIG00004538	ff
NODE_41_length_192097_cov_42.719078	fig|6666666.34159.peg.942	peg	NODE_41_length_192097_cov_42.719078_152716_152591	152716	2E+05	-	hypothetical protein		
NODE_41_length_192097_cov_42.719078	fig|6666666.34159.peg.943	peg	NODE_41_length_192097_cov_42.719078_152772_153881	152772	2E+05	+	tRNA-guanine transglycosylase (EC 2.4.2.29)	FIG00000274	isu;tRNA_modification_Bacteria isu;Queuosine-Archaeosine_Biosynthesis icw(1);CBSS-211586.1.peg.2832
NODE_41_length_192097_cov_42.719078	fig|6666666.34159.peg.944	peg	NODE_41_length_192097_cov_42.719078_153906_154298	153906	2E+05	+	Preprotein translocase subunit YajC (TC 3.A.5.1.1)	FIG00051656	isu;CBSS-211586.1.peg.2832
NODE_41_length_192097_cov_42.719078	fig|6666666.34159.peg.945	peg	NODE_41_length_192097_cov_42.719078_154897_154295	154897	2E+05	-	Holliday junction DNA helicase RuvA	FIG00000314	icw(1);RuvABC_plus_a_hypothetical
NODE_41_length_192097_cov_42.719078	fig|6666666.34159.peg.946	peg	NODE_41_length_192097_cov_42.719078_155410_154916	155410	2E+05	-	Crossover junction endodeoxyribonuclease RuvC (EC 3.1.22.4)	FIG00095112	isu;RuvABC_plus_a_hypothetical
NODE_41_length_192097_cov_42.719078	fig|6666666.34159.peg.947	peg	NODE_41_length_192097_cov_42.719078_157049_155415	157049	2E+05	-	hypothetical protein	FIG00638284	ff
NODE_41_length_192097_cov_42.719078	fig|6666666.34159.peg.948	peg	NODE_41_length_192097_cov_42.719078_157196_158257	157196	2E+05	+	ADP-heptose--lipooligosaccharide heptosyltransferase II (EC 2.4.1.-)	FIG00013366	isu;LOS_core_oligosaccharide_biosynthesis
NODE_41_length_192097_cov_42.719078	fig|6666666.34159.peg.949	peg	NODE_41_length_192097_cov_42.719078_158264_159310	158264	2E+05	+	A/G-specific adenine glycosylase (EC 3.2.2.-)	FIG00000395	isu;DNA_repair,_bacterial isu;CBSS-176280.1.peg.1561
NODE_41_length_192097_cov_42.719078	fig|6666666.34159.peg.950	peg	NODE_41_length_192097_cov_42.719078_159716_159312	159716	2E+05	-	hypothetical protein		
NODE_41_length_192097_cov_42.719078	fig|6666666.34159.peg.951	peg	NODE_41_length_192097_cov_42.719078_160561_159758	160561	2E+05	-	SET domain protein		
NODE_41_length_192097_cov_42.719078	fig|6666666.34159.peg.952	peg	NODE_41_length_192097_cov_42.719078_161037_160636	161037	2E+05	-	probable histone H1-like protein	FIG01502818	ff
NODE_41_length_192097_cov_42.719078	fig|6666666.34159.peg.953	peg	NODE_41_length_192097_cov_42.719078_161248_161117	161248	2E+05	-	hypothetical protein		
NODE_41_length_192097_cov_42.719078	fig|6666666.34159.peg.954	peg	NODE_41_length_192097_cov_42.719078_161517_164336	161517	2E+05	+	hypothetical protein	FIG00638284	ff
NODE_41_length_192097_cov_42.719078	fig|6666666.34159.peg.955	peg	NODE_41_length_192097_cov_42.719078_164323_164979	164323	2E+05	+	MotA/TolQ/ExbB proton channel family protein	FIG00002161	idu(1);Ton_and_Tol_transport_systems
NODE_41_length_192097_cov_42.719078	fig|6666666.34159.peg.956	peg	NODE_41_length_192097_cov_42.719078_164993_165439	164993	2E+05	+	Biopolymer transport protein ExbD/TolR	FIG00017761	icw(1);Ton_and_Tol_transport_systems
NODE_41_length_192097_cov_42.719078	fig|6666666.34159.peg.957	peg	NODE_41_length_192097_cov_42.719078_165445_166218	165445	2E+05	+	hypothetical protein		
NODE_41_length_192097_cov_42.719078	fig|6666666.34159.peg.958	peg	NODE_41_length_192097_cov_42.719078_166503_168137	166503	2E+05	+	hypothetical protein		
NODE_41_length_192097_cov_42.719078	fig|6666666.34159.peg.959	peg	NODE_41_length_192097_cov_42.719078_168220_169047	168220	2E+05	+	hypothetical protein	FIG00638284	ff
NODE_41_length_192097_cov_42.719078	fig|6666666.34159.peg.960	peg	NODE_41_length_192097_cov_42.719078_171490_169070	171490	2E+05	-	hypothetical protein	FIG00638284	ff
NODE_41_length_192097_cov_42.719078	fig|6666666.34159.peg.961	peg	NODE_41_length_192097_cov_42.719078_172607_171660	172607	2E+05	-	Rhodanese domain protein UPF0176, Firmicutes subgroup	FIG00009035	isu;Single-Rhodanese-domain_proteins
NODE_41_length_192097_cov_42.719078	fig|6666666.34159.peg.962	peg	NODE_41_length_192097_cov_42.719078_173630_172668	173630	2E+05	-	PTP-like phytase		
NODE_41_length_192097_cov_42.719078	fig|6666666.34159.peg.963	peg	NODE_41_length_192097_cov_42.719078_173853_174173	173853	2E+05	+	Thioredoxin	FIG01318274	idu(1);CBSS-315749.4.peg.3658 idu(1);Glycine_reductase,_sarcosine_reductase_and_betaine_reductase
NODE_41_length_192097_cov_42.719078	fig|6666666.34159.peg.964	peg	NODE_41_length_192097_cov_42.719078_174670_174218	174670	2E+05	-	tRNA (cytosine34-2'-O-)-methyltransferase (EC 2.1.1.-)	FIG00001105	ff
NODE_41_length_192097_cov_42.719078	fig|6666666.34159.peg.965	peg	NODE_41_length_192097_cov_42.719078_175515_174736	175515	2E+05	-	putative mip (macrophage infectivity potentiator, fkbp-type peptidyl-prolyl cis-trans isomerase)	FIG00899491	ff
NODE_41_length_192097_cov_42.719078	fig|6666666.34159.peg.966	peg	NODE_41_length_192097_cov_42.719078_177373_175592	177373	2E+05	-	Aspartyl-tRNA synthetase (EC 6.1.1.12)	FIG00000750	idu(1);tRNA_aminoacylation,_Asp_and_Asn
NODE_41_length_192097_cov_42.719078	fig|6666666.34159.peg.967	peg	NODE_41_length_192097_cov_42.719078_178849_177404	178849	2E+05	-	Histidyl-tRNA synthetase (EC 6.1.1.21)	FIG00000087	isu;tRNA_aminoacylation,_His
NODE_41_length_192097_cov_42.719078	fig|6666666.34159.peg.968	peg	NODE_41_length_192097_cov_42.719078_179546_179409	179546	2E+05	-	hypothetical protein		
NODE_41_length_192097_cov_42.719078	fig|6666666.34159.peg.969	peg	NODE_41_length_192097_cov_42.719078_179648_181045	179648	2E+05	+	hypothetical protein	FIG00638284	ff
NODE_41_length_192097_cov_42.719078	fig|6666666.34159.peg.970	peg	NODE_41_length_192097_cov_42.719078_182231_181215	182231	2E+05	-	hypothetical protein		
NODE_41_length_192097_cov_42.719078	fig|6666666.34159.peg.971	peg	NODE_41_length_192097_cov_42.719078_183939_182416	183939	2E+05	-	Sodium-dependent transporter		
NODE_41_length_192097_cov_42.719078	fig|6666666.34159.peg.972	peg	NODE_41_length_192097_cov_42.719078_184138_185376	184138	2E+05	+	Heavy metal RND efflux outer membrane protein, CzcC family	FIG00049097	idu(1);Cobalt-zinc-cadmium_resistance
NODE_41_length_192097_cov_42.719078	fig|6666666.34159.peg.973	peg	NODE_41_length_192097_cov_42.719078_185378_186562	185378	2E+05	+	Cobalt/zinc/cadmium efflux RND transporter, membrane fusion protein, CzcB family	FIG00033787	icw(1);Cobalt-zinc-cadmium_resistance
NODE_41_length_192097_cov_42.719078	fig|6666666.34159.peg.974	peg	NODE_41_length_192097_cov_42.719078_186562_189771	186562	2E+05	+	Cobalt-zinc-cadmium resistance protein CzcA; Cation efflux system protein CusA	FIG00008299	icw(2);Cobalt-zinc-cadmium_resistance icw(2);Cobalt-zinc-cadmium_resistance
NODE_41_length_192097_cov_42.719078	fig|6666666.34159.peg.975	peg	NODE_41_length_192097_cov_42.719078_189784_190941	189784	2E+05	+	NADH:flavin oxidoreductase/NADH oxidase	FIG01602849	ff
NODE_41_length_192097_cov_42.719078	fig|6666666.34159.peg.976	peg	NODE_41_length_192097_cov_42.719078_191464_190982	191464	2E+05	-	NUDIX domain protein(EC:6.-)		
NODE_41_length_192097_cov_42.719078	fig|6666666.34159.peg.977	peg	NODE_41_length_192097_cov_42.719078_191443_191577	191443	2E+05	+	hypothetical protein		
NODE_41_length_192097_cov_42.719078	fig|6666666.34159.rna.16	rna	NODE_41_length_192097_cov_42.719078_191645_191731	191645	2E+05	+	tRNA-Ser-GCT		
NODE_41_length_192097_cov_42.719078	fig|6666666.34159.peg.978	peg	NODE_41_length_192097_cov_42.719078_192011_191895	192011	2E+05	-	hypothetical protein		
NODE_44_length_1683_cov_109.138451	fig|6666666.34159.rna.17	rna	NODE_44_length_1683_cov_109.138451_1618_85	1618	85	-	Small Subunit Ribosomal RNA; ssuRNA; SSU rRNA		
NODE_45_length_2423_cov_44.705452	fig|6666666.34159.peg.979	peg	NODE_45_length_2423_cov_44.705452_433_275	433	275	-	hypothetical protein		
NODE_45_length_2423_cov_44.705452	fig|6666666.34159.peg.980	peg	NODE_45_length_2423_cov_44.705452_862_725	862	725	-	hypothetical protein		
NODE_45_length_2423_cov_44.705452	fig|6666666.34159.peg.981	peg	NODE_45_length_2423_cov_44.705452_1568_1098	1568	1098	-	hypothetical protein	FIG00638284	ff
NODE_45_length_2423_cov_44.705452	fig|6666666.34159.peg.982	peg	NODE_45_length_2423_cov_44.705452_1732_1589	1732	1589	-	hypothetical protein		
NODE_48_length_26061_cov_41.566296	fig|6666666.34159.peg.983	peg	NODE_48_length_26061_cov_41.566296_390_1106	390	1106	+	Rhs family protein		
NODE_48_length_26061_cov_41.566296	fig|6666666.34159.peg.984	peg	NODE_48_length_26061_cov_41.566296_1205_1591	1205	1591	+	hypothetical protein		
NODE_48_length_26061_cov_41.566296	fig|6666666.34159.peg.985	peg	NODE_48_length_26061_cov_41.566296_1625_1765	1625	1765	+	hypothetical protein		

NODE_48_length_26061_cov_41.566296	fig|6666666.34159.peg.986	peg	NODE_48_length_26061_cov_41.566296_2046_2426	2046	2426	+	hypothetical protein		
NODE_48_length_26061_cov_41.566296	fig|6666666.34159.peg.987	peg	NODE_48_length_26061_cov_41.566296_2468_2653	2468	2653	+	hypothetical protein		
NODE_48_length_26061_cov_41.566296	fig|6666666.34159.peg.988	peg	NODE_48_length_26061_cov_41.566296_4512_2755	4512	2755	-	Oligoendopeptidase F	FIG00628107	ff
NODE_48_length_26061_cov_41.566296	fig|6666666.34159.peg.989	peg	NODE_48_length_26061_cov_41.566296_6012_4618	6012	4618	-	probable two-component response regulator		
NODE_48_length_26061_cov_41.566296	fig|6666666.34159.peg.990	peg	NODE_48_length_26061_cov_41.566296_7181_6042	7181	6042	-	putative two-component sensor histidine kinase		
NODE_48_length_26061_cov_41.566296	fig|6666666.34159.peg.991	peg	NODE_48_length_26061_cov_41.566296_7172_7330	7172	7330	+	hypothetical protein		
NODE_48_length_26061_cov_41.566296	fig|6666666.34159.peg.992	peg	NODE_48_length_26061_cov_41.566296_7364_7477	7364	7477	+	hypothetical protein		
NODE_48_length_26061_cov_41.566296	fig|6666666.34159.peg.993	peg	NODE_48_length_26061_cov_41.566296_7604_8224	7604	8224	+	SSU ribosomal protein S4p (S9e)	FIG00000148	isu;CBSS-393130.3.peg.794
NODE_48_length_26061_cov_41.566296	fig|6666666.34159.peg.994	peg	NODE_48_length_26061_cov_41.566296_8386_10002	8386	10002	+	hypothetical protein	FIG00638284	ff
NODE_48_length_26061_cov_41.566296	fig|6666666.34159.peg.995	peg	NODE_48_length_26061_cov_41.566296_10206_10586	10206	10586	+	hypothetical protein		
NODE_48_length_26061_cov_41.566296	fig|6666666.34159.peg.996	peg	NODE_48_length_26061_cov_41.566296_10636_10758	10636	10758	+	hypothetical protein		
NODE_48_length_26061_cov_41.566296	fig|6666666.34159.peg.997	peg	NODE_48_length_26061_cov_41.566296_11382_10867	11382	10867	-	hypothetical protein		
NODE_48_length_26061_cov_41.566296	fig|6666666.34159.peg.998	peg	NODE_48_length_26061_cov_41.566296_11878_11360	11878	11360	-	major royal jelly protein		
NODE_48_length_26061_cov_41.566296	fig|6666666.34159.peg.999	peg	NODE_48_length_26061_cov_41.566296_13689_12052	13689	12052	-	Virulence factor mviN homolog	FIG00874812	ff
NODE_48_length_26061_cov_41.566296	fig|6666666.34159.peg.1000	peg	NODE_48_length_26061_cov_41.566296_13664_13780	13664	13780	+	hypothetical protein		
NODE_48_length_26061_cov_41.566296	fig|6666666.34159.peg.1001	peg	NODE_48_length_26061_cov_41.566296_13780_14637	13780	14637	+	Endonuclease IV (EC 3.1.21.2)	FIG00000899	isu;DNA_repair,_bacterial
NODE_48_length_26061_cov_41.566296	fig|6666666.34159.peg.1002	peg	NODE_48_length_26061_cov_41.566296_14677_16080	14677	16080	+	Asparaginyl-tRNA synthetase (EC 6.1.1.22)	FIG00000636	isu;tRNA_aminoacylation,_Asp_and_Asn
NODE_48_length_26061_cov_41.566296	fig|6666666.34159.peg.1003	peg	NODE_48_length_26061_cov_41.566296_17966_16122	17966	16122	-	Signal peptidase I (EC 3.4.21.89)	FIG00000015	isu;Signal_peptidase
NODE_48_length_26061_cov_41.566296	fig|6666666.34159.peg.1004	peg	NODE_48_length_26061_cov_41.566296_19088_18354	19088	18354	-	hypothetical protein	FIG00638284	ff
NODE_48_length_26061_cov_41.566296	fig|6666666.34159.peg.1005	peg	NODE_48_length_26061_cov_41.566296_19208_20347	19208	20347	+	Glycerate kinase (EC 2.7.1.31)	FIG00000456	isu;Glycerate_metabolism isu;Glycine_and_Serine_Utilization isu;Allantoin_Utilization isu;D-galactarate,_D [illegible]
NODE_48_length_26061_cov_41.566296	fig|6666666.34159.peg.1006	peg	NODE_48_length_26061_cov_41.566296_20401_21921	20401	21921	+	Nicotinate phosphoribosyltransferase (EC 2.4.2.11)	FIG00000387	isu;NAD_and_NADP_cofactor_biosynthesis_global
NODE_48_length_26061_cov_41.566296	fig|6666666.34159.peg.1007	peg	NODE_48_length_26061_cov_41.566296_21923_22555	21923	22555	+	Nicotinamidase (EC 3.5.1.19)	FIG00067300	icw(1);NAD_and_NADP_cofactor_biosynthesis_global isu;Niacin-Choline_transport_and_metabolism [illegible]
NODE_48_length_26061_cov_41.566296	fig|6666666.34159.peg.1008	peg	NODE_48_length_26061_cov_41.566296_22565_22921	22565	22921	+	Arsenate reductase (EC 1.20.4.1)	FIG00000770	isu;CBSS-315749.4.peg.3658 isu;Arsenic_resistance isu;Anaerobic_respiratory_reductases [illegible]
NODE_48_length_26061_cov_41.566296	fig|6666666.34159.peg.1009	peg	NODE_48_length_26061_cov_41.566296_22928_23884	22928	23884	+	Cobalt-zinc-cadmium resistance protein	FIG00000207	isu;Cobalt-zinc-cadmium_resistance
NODE_48_length_26061_cov_41.566296	fig|6666666.34159.peg.1010	peg	NODE_48_length_26061_cov_41.566296_24289_24414	24289	24414	+	hypothetical protein		
NODE_48_length_26061_cov_41.566296	fig|6666666.34159.peg.1011	peg	NODE_48_length_26061_cov_41.566296_25043_24441	25043	24441	-	hypothetical protein		
NODE_48_length_26061_cov_41.566296	fig|6666666.34159.peg.1012	peg	NODE_48_length_26061_cov_41.566296_25520_25374	25520	25374	-	hypothetical protein		
NODE_4_length_25125_cov_44.165035	fig|6666666.34159.peg.1013	peg	NODE_4_length_25125_cov_44.165035_721_1209	721	1209	+	conserved protein		
NODE_4_length_25125_cov_44.165035	fig|6666666.34159.peg.1014	peg	NODE_4_length_25125_cov_44.165035_1206_2672	1206	2672	+	hypothetical protein		
NODE_4_length_25125_cov_44.165035	fig|6666666.34159.peg.1015	peg	NODE_4_length_25125_cov_44.165035_3807_2656	3807	2656	-	hypothetical protein	FIG00638284	ff
NODE_4_length_25125_cov_44.165035	fig|6666666.34159.peg.1016	peg	NODE_4_length_25125_cov_44.165035_4644_3823	4644	3823	-	hypothetical protein		
NODE_4_length_25125_cov_44.165035	fig|6666666.34159.peg.1017	peg	NODE_4_length_25125_cov_44.165035_4918_5655	4918	5655	+	hypothetical protein		
NODE_4_length_25125_cov_44.165035	fig|6666666.34159.peg.1018	peg	NODE_4_length_25125_cov_44.165035_5736_6884	5736	6884	+	hypothetical protein		
NODE_4_length_25125_cov_44.165035	fig|6666666.34159.peg.1019	peg	NODE_4_length_25125_cov_44.165035_7250_7756	7250	7756	+	TPR repeat		
NODE_4_length_25125_cov_44.165035	fig|6666666.34159.peg.1020	peg	NODE_4_length_25125_cov_44.165035_7780_8964	7780	8964	+	hypothetical protein		
NODE_4_length_25125_cov_44.165035	fig|6666666.34159.peg.1021	peg	NODE_4_length_25125_cov_44.165035_9336_11228	9336	11228	+	hypothetical protein	FIG00638284	ff
NODE_4_length_25125_cov_44.165035	fig|6666666.34159.peg.1022	peg	NODE_4_length_25125_cov_44.165035_11471_12088	11471	12088	+	hypothetical protein		
NODE_4_length_25125_cov_44.165035	fig|6666666.34159.peg.1023	peg	NODE_4_length_25125_cov_44.165035_12311_14203	12311	14203	+	hypothetical protein	FIG00638284	ff
NODE_4_length_25125_cov_44.165035	fig|6666666.34159.peg.1024	peg	NODE_4_length_25125_cov_44.165035_14298_14753	14298	14753	+	DoxX		
NODE_4_length_25125_cov_44.165035	fig|6666666.34159.peg.1025	peg	NODE_4_length_25125_cov_44.165035_15223_14759	15223	14759	-	6,7-dimethyl-8-ribityllumazine synthase (EC 2.5.1.78)	FIG00000231	isu;Riboflavin_synthesis_cluster icw(1);Riboflavin,_FMN_and_FAD_metabolism
NODE_4_length_25125_cov_44.165035	fig|6666666.34159.peg.1026	peg	NODE_4_length_25125_cov_44.165035_16445_15234	16445	15234	-	3,4-dihydroxy-2-butanone 4-phosphate synthase (EC 4.1.99.12) / GTP cyclohydrolase II (EC 3.5.4.25)	FIG00000378	icw(1);Riboflavin_synthesis_cluster icw(2);Riboflavin_synthesis_cluster icw(2);Riboflavin,_FMN_and_FAD_metabolism [illegible]
NODE_4_length_25125_cov_44.165035	fig|6666666.34159.peg.1027	peg	NODE_4_length_25125_cov_44.165035_17080_16442	17080	16442	-	Riboflavin synthase eubacterial/eukaryotic (EC 2.5.1.9)	FIG00000273	icw(2);Riboflavin_synthesis_cluster isu;Riboflavin,_FMN_and_FAD_metabolism isu;riboflavin_to_FAD [illegible]
NODE_4_length_25125_cov_44.165035	fig|6666666.34159.peg.1028	peg	NODE_4_length_25125_cov_44.165035_18942_17083	18942	17083	-	hypothetical protein	FIG00638284	ff
NODE_4_length_25125_cov_44.165035	fig|6666666.34159.peg.1029	peg	NODE_4_length_25125_cov_44.165035_19812_18958	19812	18958	-	hypothetical protein	FIG00638284	ff
NODE_4_length_25125_cov_44.165035	fig|6666666.34159.peg.1030	peg	NODE_4_length_25125_cov_44.165035_20653_19910	20653	19910	-	tRNA(Cytosine32)-2-thiocytidine synthetase	FIG00138938	isu;tRNA_modification_Bacteria isu;CBSS-326442.4.peg.1852
NODE_4_length_25125_cov_44.165035	fig|6666666.34159.peg.1031	peg	NODE_4_length_25125_cov_44.165035_21005_20865	21005	20865	-	hypothetical protein		
NODE_4_length_25125_cov_44.165035	fig|6666666.34159.peg.1032	peg	NODE_4_length_25125_cov_44.165035_21037_21153	21037	21153	+	hypothetical protein		
NODE_4_length_25125_cov_44.165035	fig|6666666.34159.peg.1033	peg	NODE_4_length_25125_cov_44.165035_21558_21893	21558	21893	+	RNP-1-like RNA-binding protein		
NODE_4_length_25125_cov_44.165035	fig|6666666.34159.peg.1034	peg	NODE_4_length_25125_cov_44.165035_22020_22178	22020	22178	+	hypothetical protein		
NODE_4_length_25125_cov_44.165035	fig|6666666.34159.peg.1035	peg	NODE_4_length_25125_cov_44.165035_22367_24034	22367	24034	+	hypothetical protein	FIG00638284	ff
NODE_4_length_25125_cov_44.165035	fig|6666666.34159.peg.1036	peg	NODE_4_length_25125_cov_44.165035_24562_24146	24562	24146	-	TnpA transposase	FIG01505217	ff
NODE_4_length_25125_cov_44.165035	fig|6666666.34159.peg.1037	peg	NODE_4_length_25125_cov_44.165035_24690_24863	24690	24863	+	hypothetical protein		
NODE_50_length_97022_cov_43.867477	fig|6666666.34159.peg.1038	peg	NODE_50_length_97022_cov_43.867477_357_1103	357	1103	+	Rhs protein		
NODE_50_length_97022_cov_43.867477	fig|6666666.34159.peg.1039	peg	NODE_50_length_97022_cov_43.867477_1116_1370	1116	1370	+	hypothetical protein		
NODE_50_length_97022_cov_43.867477	fig|6666666.34159.peg.1040	peg	NODE_50_length_97022_cov_43.867477_1479_1595	1479	1595	+	hypothetical protein		
NODE_50_length_97022_cov_43.867477	fig|6666666.34159.peg.1041	peg	NODE_50_length_97022_cov_43.867477_1973_2179	1973	2179	+	putative transposase		
NODE_50_length_97022_cov_43.867477	fig|6666666.34159.peg.1042	peg	NODE_50_length_97022_cov_43.867477_2823_2704	2823	2704	-	hypothetical protein		
NODE_50_length_97022_cov_43.867477	fig|6666666.34159.peg.1043	peg	NODE_50_length_97022_cov_43.867477_3243_8696	3243	8696	+	hypothetical protein	FIG00638284	ff
NODE_50_length_97022_cov_43.867477	fig|6666666.34159.peg.1044	peg	NODE_50_length_97022_cov_43.867477_8693_9226	8693	9226	+	hypothetical protein		

NODE_50_length_97022_cov_43.867477	fig|6666666.34159.peg.1045	peg	NODE_50_length_97022_cov_43.867477_9261_9524	9261	9524	+	hypothetical protein		
NODE_50_length_97022_cov_43.867477	fig|6666666.34159.peg.1046	peg	NODE_50_length_97022_cov_43.867477_9554_9739	9554	9739	+	hypothetical protein		
NODE_50_length_97022_cov_43.867477	fig|6666666.34159.peg.1047	peg	NODE_50_length_97022_cov_43.867477_9820_10356	9820	10356	+	hypothetical protein		
NODE_50_length_97022_cov_43.867477	fig|6666666.34159.peg.1048	peg	NODE_50_length_97022_cov_43.867477_10346_10480	10346	10480	+	hypothetical protein		
NODE_50_length_97022_cov_43.867477	fig|6666666.34159.peg.1049	peg	NODE_50_length_97022_cov_43.867477_10521_11048	10521	11048	+	hypothetical protein		
NODE_50_length_97022_cov_43.867477	fig|6666666.34159.peg.1050	peg	NODE_50_length_97022_cov_43.867477_11101_11676	11101	11676	+	hypothetical protein		
NODE_50_length_97022_cov_43.867477	fig|6666666.34159.peg.1051	peg	NODE_50_length_97022_cov_43.867477_11702_12226	11702	12226	+	hypothetical protein		
NODE_50_length_97022_cov_43.867477	fig|6666666.34159.peg.1052	peg	NODE_50_length_97022_cov_43.867477_12272_12823	12272	12823	+	hypothetical protein		
NODE_50_length_97022_cov_43.867477	fig|6666666.34159.peg.1053	peg	NODE_50_length_97022_cov_43.867477_14585_12849	14585	12849	-	FIG00493990: hypothetical protein	FIG00493401	ff
NODE_50_length_97022_cov_43.867477	fig|6666666.34159.peg.1054	peg	NODE_50_length_97022_cov_43.867477_15136_14582	15136	14582	-	Isochorismatase (EC 3.3.2.1)	FIG00009017	isu;Chorismate:_Intermediate_for_synthesis_of_Tryptophan,_PAPA_antibiotics,_PABA,_3-hydroxyanthranilate_and_more
NODE_50_length_97022_cov_43.867477	fig|6666666.34159.peg.1055	peg	NODE_50_length_97022_cov_43.867477_15490_15158	15490	15158	-	putative protein kinase C inhibitor 1		
NODE_50_length_97022_cov_43.867477	fig|6666666.34159.peg.1056	peg	NODE_50_length_97022_cov_43.867477_16377_15487	16377	15487	-	FIG00493905: hypothetical protein	FIG00493277	ff
NODE_50_length_97022_cov_43.867477	fig|6666666.34159.peg.1057	peg	NODE_50_length_97022_cov_43.867477_16947_19076	16947	19076	+	FIG00494344: hypothetical protein	FIG00493535	ff
NODE_50_length_97022_cov_43.867477	fig|6666666.34159.peg.1058	peg	NODE_50_length_97022_cov_43.867477_19928_19080	19928	19080	-	hypothetical protein	FIG00638284	ff
NODE_50_length_97022_cov_43.867477	fig|6666666.34159.peg.1059	peg	NODE_50_length_97022_cov_43.867477_21059_19932	21059	19932	-	hypothetical protein		
NODE_50_length_97022_cov_43.867477	fig|6666666.34159.peg.1060	peg	NODE_50_length_97022_cov_43.867477_21592_22623	21592	22623	+	hypothetical protein	FIG00638284	ff
NODE_50_length_97022_cov_43.867477	fig|6666666.34159.peg.1061	peg	NODE_50_length_97022_cov_43.867477_23409_22627	23409	22627	-	ABC-type multidrug transport system, permease component		
NODE_50_length_97022_cov_43.867477	fig|6666666.34159.peg.1062	peg	NODE_50_length_97022_cov_43.867477_24200_23412	24200	23412	-	ABC transporter permease protein		
NODE_50_length_97022_cov_43.867477	fig|6666666.34159.peg.1063	peg	NODE_50_length_97022_cov_43.867477_25182_24193	25182	24193	-	ABC transporter, ATP-binding protein	FIG00744535	ff
NODE_50_length_97022_cov_43.867477	fig|6666666.34159.peg.1064	peg	NODE_50_length_97022_cov_43.867477_25326_25931	25326	25931	+	hypothetical protein		
NODE_50_length_97022_cov_43.867477	fig|6666666.34159.peg.1065	peg	NODE_50_length_97022_cov_43.867477_28297_25928	28297	25928	-	FIG00495827: hypothetical protein	FIG00495824	ff
NODE_50_length_97022_cov_43.867477	fig|6666666.34159.peg.1066	peg	NODE_50_length_97022_cov_43.867477_28450_28325	28450	28325	-	hypothetical protein		
NODE_50_length_97022_cov_43.867477	fig|6666666.34159.peg.1067	peg	NODE_50_length_97022_cov_43.867477_29771_28404	29771	28404	-	putative sodium/pantothenate symporter (pantothenate permease)		
NODE_50_length_97022_cov_43.867477	fig|6666666.34159.peg.1068	peg	NODE_50_length_97022_cov_43.867477_29934_30098	29934	30098	+	hypothetical protein		
NODE_50_length_97022_cov_43.867477	fig|6666666.34159.peg.1069	peg	NODE_50_length_97022_cov_43.867477_30226_31854	30226	31854	+	hypothetical protein		
NODE_50_length_97022_cov_43.867477	fig|6666666.34159.peg.1070	peg	NODE_50_length_97022_cov_43.867477_31902_32492	31902	32492	+	hypothetical protein		
NODE_50_length_97022_cov_43.867477	fig|6666666.34159.peg.1071	peg	NODE_50_length_97022_cov_43.867477_32570_33130	32570	33130	+	hypothetical protein		
NODE_50_length_97022_cov_43.867477	fig|6666666.34159.peg.1072	peg	NODE_50_length_97022_cov_43.867477_34659_33127	34659	33127	-	ADP/ATP Translocase, NTT1	FIG01345036	ff
NODE_50_length_97022_cov_43.867477	fig|6666666.34159.peg.1073	peg	NODE_50_length_97022_cov_43.867477_36035_34923	36035	34923	-	FIG00758466: hypothetical protein	FIG00758465	ff
NODE_50_length_97022_cov_43.867477	fig|6666666.34159.peg.1074	peg	NODE_50_length_97022_cov_43.867477_36981_36046	36981	36046	-	hypothetical protein	FIG00638284	ff
NODE_50_length_97022_cov_43.867477	fig|6666666.34159.peg.1075	peg	NODE_50_length_97022_cov_43.867477_37086_37496	37086	37496	+	hypothetical protein		
NODE_50_length_97022_cov_43.867477	fig|6666666.34159.peg.1076	peg	NODE_50_length_97022_cov_43.867477_37919_37542	37919	37542	-	hypothetical protein		
NODE_50_length_97022_cov_43.867477	fig|6666666.34159.peg.1077	peg	NODE_50_length_97022_cov_43.867477_38171_38010	38171	38010	-	hypothetical protein		
NODE_50_length_97022_cov_43.867477	fig|6666666.34159.peg.1078	peg	NODE_50_length_97022_cov_43.867477_38628_38209	38628	38209	-	Acetyltransferase (EC 2.3.1.-)		
NODE_50_length_97022_cov_43.867477	fig|6666666.34159.peg.1079	peg	NODE_50_length_97022_cov_43.867477_40811_38634	40811	38634	-	hypothetical protein	FIG00638284	ff
NODE_50_length_97022_cov_43.867477	fig|6666666.34159.peg.1080	peg	NODE_50_length_97022_cov_43.867477_41151_41678	41151	41678	+	BclA protein		
NODE_50_length_97022_cov_43.867477	fig|6666666.34159.peg.1081	peg	NODE_50_length_97022_cov_43.867477_41685_42251	41685	42251	+	hypothetical protein		
NODE_50_length_97022_cov_43.867477	fig|6666666.34159.peg.1082	peg	NODE_50_length_97022_cov_43.867477_42513_43058	42513	43058	+	Phage tail fiber protein		idu(1);Phage_tail_fiber_proteins
NODE_50_length_97022_cov_43.867477	fig|6666666.34159.peg.1083	peg	NODE_50_length_97022_cov_43.867477_43078_43638	43078	43638	+	BclA protein		
NODE_50_length_97022_cov_43.867477	fig|6666666.34159.peg.1084	peg	NODE_50_length_97022_cov_43.867477_43821_45260	43821	45260	+	hypothetical protein	FIG00638284	ff
NODE_50_length_97022_cov_43.867477	fig|6666666.34159.peg.1085	peg	NODE_50_length_97022_cov_43.867477_46322_45264	46322	45264	-	Alcohol dehydrogenase (EC 1.1.1.1)	FIG01007502	idu(3);Fermentations:_Mixed_acid idu(3);Butanol_Biosynthesis
NODE_50_length_97022_cov_43.867477	fig|6666666.34159.peg.1086	peg	NODE_50_length_97022_cov_43.867477_46445_47053	46445	47053	+	hypothetical protein		
NODE_50_length_97022_cov_43.867477	fig|6666666.34159.peg.1087	peg	NODE_50_length_97022_cov_43.867477_47129_48535	47129	48535	+	hypothetical protein		
NODE_50_length_97022_cov_43.867477	fig|6666666.34159.peg.1088	peg	NODE_50_length_97022_cov_43.867477_48625_48864	48625	48864	+	hypothetical protein		
NODE_50_length_97022_cov_43.867477	fig|6666666.34159.peg.1089	peg	NODE_50_length_97022_cov_43.867477_50378_48903	50378	48903	-	RND efflux system, outer membrane lipoprotein CmeC	FIG00006235	icw(1);Multidrug_Resistance_Efflux_Pumps
NODE_50_length_97022_cov_43.867477	fig|6666666.34159.peg.1090	peg	NODE_50_length_97022_cov_43.867477_53494_50375	53494	50375	-	RND efflux system, inner membrane transporter CmeB	FIG00034851	icw(2);Multidrug_Resistance_Efflux_Pumps
NODE_50_length_97022_cov_43.867477	fig|6666666.34159.peg.1091	peg	NODE_50_length_97022_cov_43.867477_54683_53502	54683	53502	-	RND efflux system, membrane fusion protein CmeA	FIG01304998	isu;Multidrug_Resistance_Efflux_Pumps
NODE_50_length_97022_cov_43.867477	fig|6666666.34159.peg.1092	peg	NODE_50_length_97022_cov_43.867477_54682_54795	54682	54795	+	hypothetical protein		
NODE_50_length_97022_cov_43.867477	fig|6666666.34159.peg.1093	peg	NODE_50_length_97022_cov_43.867477_55341_54778	55341	54778	-	MutT domain containing protein		
NODE_50_length_97022_cov_43.867477	fig|6666666.34159.peg.1094	peg	NODE_50_length_97022_cov_43.867477_55548_56033	55548	56033	+	tRNA-specific adenosine-34 deaminase (EC 3.5.4.-)	FIG00000291	idu(1);tRNA_processing idu(1);tRNA_modification_Bacteria
NODE_50_length_97022_cov_43.867477	fig|6666666.34159.peg.1095	peg	NODE_50_length_97022_cov_43.867477_56035_57360	56035	57360	+	Amine oxidase (flavin-containing)(EC:1.4.3.4)		
NODE_50_length_97022_cov_43.867477	fig|6666666.34159.peg.1096	peg	NODE_50_length_97022_cov_43.867477_57347_58282	57347	58282	+	hypothetical protein		
NODE_50_length_97022_cov_43.867477	fig|6666666.34159.peg.1097	peg	NODE_50_length_97022_cov_43.867477_60281_58257	60281	58257	-	acetyltransferase, GNAT family	FIG01315364	ff
NODE_50_length_97022_cov_43.867477	fig|6666666.34159.peg.1098	peg	NODE_50_length_97022_cov_43.867477_61371_60262	61371	60262	-	3-dehydroquinate synthase (EC 4.2.3.4)	FIG00000224	icw(1);Common_Pathway_For_Synthesis_of_Aromatic_Compounds_(DAHP_synthase_to_chorismate)
NODE_50_length_97022_cov_43.867477	fig|6666666.34159.peg.1099	peg	NODE_50_length_97022_cov_43.867477_62459_61368	62459	61368	-	Chorismate synthase (EC 4.2.3.5)	FIG00000212	icw(2);Common_Pathway_For_Synthesis_of_Aromatic_Compounds_(DAHP_synthase_to_chorismate)
NODE_50_length_97022_cov_43.867477	fig|6666666.34159.peg.1100	peg	NODE_50_length_97022_cov_43.867477_62982_62449	62982	62449	-	Shikimate kinase(EC:2.7.1.71)		
NODE_50_length_97022_cov_43.867477	fig|6666666.34159.peg.1101	peg	NODE_50_length_97022_cov_43.867477_64265_62979	64265	62979	-	5-Enolpyruvylshikimate-3-phosphate synthase (EC 2.5.1.19)	FIG00075970	isu;Common_Pathway_For_Synthesis_of_Aromatic_Compounds_(DAHP_synthase_to_chorismate)
NODE_50_length_97022_cov_43.867477	fig|6666666.34159.peg.1102	peg	NODE_50_length_97022_cov_43.867477_64402_65073	64402	65073	+	Beta-phosphoglucomutase (EC 5.4.2.6)		isu;N-Acetyl-Galactosamine_and_Galactosamine_Utilization isu;Maltose_and_Maltodextrin_Utilization
NODE_50_length_97022_cov_43.867477	fig|6666666.34159.peg.1103	peg	NODE_50_length_97022_cov_43.867477_65494_65066	65494	65066	-	hypothetical protein	FIG00638284	ff

NODE_50_length_97022_cov_43.867477	fig|6666666.34159.peg.1104	peg	NODE_50_length_97022_cov_43.867477_65675_65505	65675	65505	-	hypothetical protein		
NODE_50_length_97022_cov_43.867477	fig|6666666.34159.peg.1105	peg	NODE_50_length_97022_cov_43.867477_65710_66153	65710	66153	+	hypothetical protein		
NODE_50_length_97022_cov_43.867477	fig|6666666.34159.peg.1106	peg	NODE_50_length_97022_cov_43.867477_66175_68703	66175	68703	+	Mg(2+) transport ATPase, P-type (EC 3.6.3.2)	FIG00112371 5	idu(2);Magnesium_transport
NODE_50_length_97022_cov_43.867477	fig|6666666.34159.peg.1107	peg	NODE_50_length_97022_cov_43.867477_68700_70598	68700	70598	+	Cadmium-transporting ATPase (EC 3.6.3.3)	FIG00503691	isu;Cobalt-zinc-cadmium_resistance
NODE_50_length_97022_cov_43.867477	fig|6666666.34159.peg.1108	peg	NODE_50_length_97022_cov_43.867477_70615_71253	70615	71253	+	hypothetical protein	FIG00638284	ff
NODE_50_length_97022_cov_43.867477	fig|6666666.34159.peg.1109	peg	NODE_50_length_97022_cov_43.867477_71784_71344	71784	71344	-	small heat shock protein (class I)		
NODE_50_length_97022_cov_43.867477	fig|6666666.34159.peg.1110	peg	NODE_50_length_97022_cov_43.867477_72861_71923	72861	71923	-	hypothetical protein		
NODE_50_length_97022_cov_43.867477	fig|6666666.34159.peg.1111	peg	NODE_50_length_97022_cov_43.867477_74179_72977	74179	72977	-	protein of unknown function DUF214		
NODE_50_length_97022_cov_43.867477	fig|6666666.34159.peg.1112	peg	NODE_50_length_97022_cov_43.867477_74880_74176	74880	74176	-	ABC transporter, ATP-binding protein		
NODE_50_length_97022_cov_43.867477	fig|6666666.34159.peg.1113	peg	NODE_50_length_97022_cov_43.867477_76817_74955	76817	74955	-	glycosyl transferase, family 2		
NODE_50_length_97022_cov_43.867477	fig|6666666.34159.peg.1114	peg	NODE_50_length_97022_cov_43.867477_76912_77589	76912	77589	+	hypothetical protein		
NODE_50_length_97022_cov_43.867477	fig|6666666.34159.peg.1115	peg	NODE_50_length_97022_cov_43.867477_78821_77586	78821	77586	-	tetracycline resistance protein		
NODE_50_length_97022_cov_43.867477	fig|6666666.34159.peg.1116	peg	NODE_50_length_97022_cov_43.867477_79534_78983	79534	78983	-	hypothetical protein		
NODE_50_length_97022_cov_43.867477	fig|6666666.34159.peg.1117	peg	NODE_50_length_97022_cov_43.867477_79716_79552	79716	79552	-	Short-chain dehydrogenase/reductase SDR(EC:1.1.1.100)		
NODE_50_length_97022_cov_43.867477	fig|6666666.34159.peg.1118	peg	NODE_50_length_97022_cov_43.867477_80303_80016	80303	80016	-	3-oxoacyl-[acyl-carrier protein] reductase (EC 1.1.1.100)	FIG00621114	idu(12);CBSS-246196.1.peg.364 idu(12);Fatty_Acid_Biosynthesis_FASII
NODE_50_length_97022_cov_43.867477	fig|6666666.34159.peg.1119	peg	NODE_50_length_97022_cov_43.867477_80458_81564	80458	81564	+	hypothetical protein		
NODE_50_length_97022_cov_43.867477	fig|6666666.34159.peg.1120	peg	NODE_50_length_97022_cov_43.867477_81685_81885	81685	81885	+	hypothetical protein		
NODE_50_length_97022_cov_43.867477	fig|6666666.34159.peg.1121	peg	NODE_50_length_97022_cov_43.867477_82664_82320	82664	82320	-	hypothetical protein		
NODE_50_length_97022_cov_43.867477	fig|6666666.34159.peg.1122	peg	NODE_50_length_97022_cov_43.867477_82789_82902	82789	82902	+	hypothetical protein		
NODE_50_length_97022_cov_43.867477	fig|6666666.34159.peg.1123	peg	NODE_50_length_97022_cov_43.867477_83548_83667	83548	83667	+	hypothetical protein		
NODE_50_length_97022_cov_43.867477	fig|6666666.34159.peg.1124	peg	NODE_50_length_97022_cov_43.867477_84390_83677	84390	83677	-	hypothetical protein		
NODE_50_length_97022_cov_43.867477	fig|6666666.34159.peg.1125	peg	NODE_50_length_97022_cov_43.867477_84767_84519	84767	84519	-	hypothetical protein		
NODE_50_length_97022_cov_43.867477	fig|6666666.34159.peg.1126	peg	NODE_50_length_97022_cov_43.867477_84847_84975	84847	84975	+	hypothetical protein		
NODE_50_length_97022_cov_43.867477	fig|6666666.34159.peg.1127	peg	NODE_50_length_97022_cov_43.867477_85707_84985	85707	84985	-	hypothetical protein		
NODE_50_length_97022_cov_43.867477	fig|6666666.34159.peg.1128	peg	NODE_50_length_97022_cov_43.867477_87204_85882	87204	85882	-	acyl-CoA thioester hydrolase/bile acid-CoA amino acid N-acetyltransferase		
NODE_50_length_97022_cov_43.867477	fig|6666666.34159.peg.1129	peg	NODE_50_length_97022_cov_43.867477_87379_87561	87379	87561	+	hypothetical protein		
NODE_50_length_97022_cov_43.867477	fig|6666666.34159.peg.1130	peg	NODE_50_length_97022_cov_43.867477_88276_87764	88276	87764	-	hypothetical protein		
NODE_50_length_97022_cov_43.867477	fig|6666666.34159.peg.1131	peg	NODE_50_length_97022_cov_43.867477_88691_88419	88691	88419	-	hypothetical protein		
NODE_50_length_97022_cov_43.867477	fig|6666666.34159.peg.1132	peg	NODE_50_length_97022_cov_43.867477_90374_88824	90374	88824	-	Glucose-methanol-choline (GMC) oxidoreductase:NAD binding site	FIG00001629	isu;Respiratory_dehydrogenases_1
NODE_50_length_97022_cov_43.867477	fig|6666666.34159.peg.1133	peg	NODE_50_length_97022_cov_43.867477_93114_90412	93114	90412	-	hypothetical protein	FIG00638284	ff
NODE_50_length_97022_cov_43.867477	fig|6666666.34159.peg.1134	peg	NODE_50_length_97022_cov_43.867477_93883_93116	93883	93116	-	3-oxoacyl-[acyl-carrier protein] reductase (EC 1.1.1.100)	FIG00621114	idu(12);CBSS-246196.1.peg.364 idu(12);Fatty_Acid_Biosynthesis_FASII
NODE_50_length_97022_cov_43.867477	fig|6666666.34159.peg.1135	peg	NODE_50_length_97022_cov_43.867477_94800_93883	94800	93883	-	major facilitator superfamily MFS_1		
NODE_50_length_97022_cov_43.867477	fig|6666666.34159.peg.1136	peg	NODE_50_length_97022_cov_43.867477_96140_95079	96140	95079	-	Gluconolactonase (EC 3.1.1.17)	FIG00127296	ff
NODE_50_length_97022_cov_43.867477	fig|6666666.34159.peg.1137	peg	NODE_50_length_97022_cov_43.867477_96117_96239	96117	96239	+	hypothetical protein		
NODE_50_length_97022_cov_43.867477	fig|6666666.34159.peg.1138	peg	NODE_50_length_97022_cov_43.867477_96397_96930	96397	96930	+	hypothetical protein		
NODE_51_length_1973_cov_46.585419	fig|6666666.34159.peg.1139	peg	NODE_51_length_1973_cov_46.585419_869_219	869	219	-	hypothetical protein	FIG00638284	ff
NODE_51_length_1973_cov_46.585419	fig|6666666.34159.peg.1140	peg	NODE_51_length_1973_cov_46.585419_1912_866	1912	866	-	hypothetical protein		
NODE_55_length_368_cov_85.843063	fig|6666666.34159.peg.1141	peg	NODE_55_length_368_cov_85.843063_363_214	363	214	-	Mobile element protein	FIG01306568	ff
NODE_5_length_258648_cov_42.850559	fig|6666666.34159.peg.1142	peg	NODE_5_length_258648_cov_42.850559_266_138	266	138	-	hypothetical protein		
NODE_5_length_258648_cov_42.850559	fig|6666666.34159.peg.1143	peg	NODE_5_length_258648_cov_42.850559_2060_345	2060	345	-	hypothetical protein	FIG00638284	ff
NODE_5_length_258648_cov_42.850559	fig|6666666.34159.peg.1144	peg	NODE_5_length_258648_cov_42.850559_2661_2807	2661	2807	+	hypothetical protein		
NODE_5_length_258648_cov_42.850559	fig|6666666.34159.peg.1145	peg	NODE_5_length_258648_cov_42.850559_3125_2913	3125	2913	-	putative Mitomycin resistance protein mcrB		
NODE_5_length_258648_cov_42.850559	fig|6666666.34159.peg.1146	peg	NODE_5_length_258648_cov_42.850559_3615_3229	3615	3229	-	Lactoylglutathione lyase (EC 4.4.1.5)	FIG00004743	isu;Glutathione:_Non-redox_reactions isu;Methylglyoxal_Metabolism
NODE_5_length_258648_cov_42.850559	fig|6666666.34159.peg.1147	peg	NODE_5_length_258648_cov_42.850559_4247_3657	4247	3657	-	putative integrase/recombinase		
NODE_5_length_258648_cov_42.850559	fig|6666666.34159.peg.1148	peg	NODE_5_length_258648_cov_42.850559_4397_4281	4397	4281	-	hypothetical protein		
NODE_5_length_258648_cov_42.850559	fig|6666666.34159.peg.1149	peg	NODE_5_length_258648_cov_42.850559_7227_4456	7227	4456	-	hypothetical protein	FIG00638284	ff
NODE_5_length_258648_cov_42.850559	fig|6666666.34159.peg.1150	peg	NODE_5_length_258648_cov_42.850559_8604_7327	8604	7327	-	Ribonuclease BN (EC 3.1.-.-)	FIG00136522	ff
NODE_5_length_258648_cov_42.850559	fig|6666666.34159.peg.1151	peg	NODE_5_length_258648_cov_42.850559_8777_8664	8777	8664	-	hypothetical protein		
NODE_5_length_258648_cov_42.850559	fig|6666666.34159.peg.1152	peg	NODE_5_length_258648_cov_42.850559_8743_9810	8743	9810	+	hypothetical protein		
NODE_5_length_258648_cov_42.850559	fig|6666666.34159.peg.1153	peg	NODE_5_length_258648_cov_42.850559_10094_9822	10094	9822	-	FIG016027: protein of unknown function YeaO		
NODE_5_length_258648_cov_42.850559	fig|6666666.34159.peg.1154	peg	NODE_5_length_258648_cov_42.850559_10168_10046	10168	10046	-	hypothetical protein		
NODE_5_length_258648_cov_42.850559	fig|6666666.34159.peg.1155	peg	NODE_5_length_258648_cov_42.850559_10865_10221	10865	10221	-	hypothetical protein		
NODE_5_length_258648_cov_42.850559	fig|6666666.34159.peg.1156	peg	NODE_5_length_258648_cov_42.850559_11083_11301	11083	11301	+	hypothetical protein		
NODE_5_length_258648_cov_42.850559	fig|6666666.34159.peg.1157	peg	NODE_5_length_258648_cov_42.850559_11415_12449	11415	12449	+	hypothetical protein		
NODE_5_length_258648_cov_42.850559	fig|6666666.34159.peg.1158	peg	NODE_5_length_258648_cov_42.850559_14768_12660	14768	12660	-	hypothetical protein		
NODE_5_length_258648_cov_42.850559	fig|6666666.34159.peg.1159	peg	NODE_5_length_258648_cov_42.850559_15008_15517	15008	15517	+	exported protein		
NODE_5_length_258648_cov_42.850559	fig|6666666.34159.peg.1160	peg	NODE_5_length_258648_cov_42.850559_15726_16649	15726	16649	+	hypothetical protein		
NODE_5_length_258648_cov_42.850559	fig|6666666.34159.peg.1161	peg	NODE_5_length_258648_cov_42.850559_16612_16725	16612	16725	+	hypothetical protein		
NODE_5_length_258648_cov_42.850559	fig|6666666.34159.peg.1162	peg	NODE_5_length_258648_cov_42.850559_16809_17720	16809	17720	+	hypothetical protein		

NODE_5_length_258648_cov_42.850559	fig|6666666.34159.peg.1163	peg	NODE_5_length_258648_cov_42.850559_17912_19624	17912	19624	+	hypothetical protein		
NODE_5_length_258648_cov_42.850559	fig|6666666.34159.peg.1164	peg	NODE_5_length_258648_cov_42.850559_20427_19954	20427	19954	-	hypothetical protein		
NODE_5_length_258648_cov_42.850559	fig|6666666.34159.peg.1165	peg	NODE_5_length_258648_cov_42.850559_20755_20636	20755	20636	-	hypothetical protein		
NODE_5_length_258648_cov_42.850559	fig|6666666.34159.peg.1166	peg	NODE_5_length_258648_cov_42.850559_20733_21569	20733	21569	+	hypothetical protein	FIG00638284	ff
NODE_5_length_258648_cov_42.850559	fig|6666666.34159.peg.1167	peg	NODE_5_length_258648_cov_42.850559_21581_22723	21581	22723	+	hypothetical protein		
NODE_5_length_258648_cov_42.850559	fig|6666666.34159.peg.1168	peg	NODE_5_length_258648_cov_42.850559_22720_23574	22720	23574	+	putative alpha-1,2-fucosyltransferase		
NODE_5_length_258648_cov_42.850559	fig|6666666.34159.peg.1169	peg	NODE_5_length_258648_cov_42.850559_23726_24985	23726	24985	+	Na+/H+ antiporter NhaD type	FIG01261528	ff
NODE_5_length_258648_cov_42.850559	fig|6666666.34159.peg.1170	peg	NODE_5_length_258648_cov_42.850559_25053_25649	25053	25649	+	LemA protein	FIG00001666	icw(1);CBSS-393011.11.peg.386
NODE_5_length_258648_cov_42.850559	fig|6666666.34159.peg.1171	peg	NODE_5_length_258648_cov_42.850559_25665_26684	25665	26684	+	Heat shock protein HtpX (EC 3.4.24.-)	FIG00005571	isu;CBSS-393011.11.peg.386
NODE_5_length_258648_cov_42.850559	fig|6666666.34159.peg.1172	peg	NODE_5_length_258648_cov_42.850559_27958_27206	27958	27206	-	hypothetical protein		
NODE_5_length_258648_cov_42.850559	fig|6666666.34159.peg.1173	peg	NODE_5_length_258648_cov_42.850559_28586_27975	28586	27975	-	hypothetical protein		
NODE_5_length_258648_cov_42.850559	fig|6666666.34159.peg.1174	peg	NODE_5_length_258648_cov_42.850559_30390_28696	30390	28696	-	Pyrophosphate--fructose 6-phosphate 1-phosphotransferase, beta subunit (EC 2.7.1.90)	FIG00003732	ff
NODE_5_length_258648_cov_42.850559	fig|6666666.34159.peg.1175	peg	NODE_5_length_258648_cov_42.850559_31201_30410	31201	30410	-	hypothetical protein		
NODE_5_length_258648_cov_42.850559	fig|6666666.34159.peg.1176	peg	NODE_5_length_258648_cov_42.850559_31351_31235	31351	31235	-	hypothetical protein		
NODE_5_length_258648_cov_42.850559	fig|6666666.34159.peg.1177	peg	NODE_5_length_258648_cov_42.850559_31368_31559	31368	31559	+	hypothetical protein		
NODE_5_length_258648_cov_42.850559	fig|6666666.34159.peg.1178	peg	NODE_5_length_258648_cov_42.850559_31572_33494	31572	33494	+	unknown protein		
NODE_5_length_258648_cov_42.850559	fig|6666666.34159.peg.1179	peg	NODE_5_length_258648_cov_42.850559_33604_33491	33604	33491	-	hypothetical protein		
NODE_5_length_258648_cov_42.850559	fig|6666666.34159.peg.1180	peg	NODE_5_length_258648_cov_42.850559_33599_34633	33599	34633	+	hypothetical protein		
NODE_5_length_258648_cov_42.850559	fig|6666666.34159.peg.1181	peg	NODE_5_length_258648_cov_42.850559_36016_34664	36016	34664	-	hypothetical protein	FIG00638284	ff
NODE_5_length_258648_cov_42.850559	fig|6666666.34159.peg.1182	peg	NODE_5_length_258648_cov_42.850559_36187_37776	36187	37776	+	hypothetical protein	FIG00638284	ff
NODE_5_length_258648_cov_42.850559	fig|6666666.34159.peg.1183	peg	NODE_5_length_258648_cov_42.850559_39097_37784	39097	37784	-	ADP-heptose synthase (EC 2.7.-.-) / D-glycero-beta-D-manno-heptose 7-phosphate kinase	FIG00014339	icw(1);LOS_core_oligosaccharide_biosynthesis icw(1);LOS_core_oligosaccharide_biosynthesis
NODE_5_length_258648_cov_42.850559	fig|6666666.34159.peg.1184	peg	NODE_5_length_258648_cov_42.850559_40074_39091	40074	39091	-	ADP-L-glycero-D-manno-heptose-6-epimerase (EC 5.1.3.20)	FIG00001052	isu;LOS_core_oligosaccharide_biosynthesis
NODE_5_length_258648_cov_42.850559	fig|6666666.34159.peg.1185	peg	NODE_5_length_258648_cov_42.850559_40135_40251	40135	40251	+	hypothetical protein		
NODE_5_length_258648_cov_42.850559	fig|6666666.34159.peg.1186	peg	NODE_5_length_258648_cov_42.850559_41999_40257	41999	40257	-	hypothetical protein	FIG00638284	ff
NODE_5_length_258648_cov_42.850559	fig|6666666.34159.peg.1187	peg	NODE_5_length_258648_cov_42.850559_42310_43374	42310	43374	+	hypothetical protein	FIG00638284	ff
NODE_5_length_258648_cov_42.850559	fig|6666666.34159.peg.1188	peg	NODE_5_length_258648_cov_42.850559_44009_43443	44009	43443	-	flavin reductase like domain, putative		
NODE_5_length_258648_cov_42.850559	fig|6666666.34159.peg.1189	peg	NODE_5_length_258648_cov_42.850559_45452_44163	45452	44163	-	Adenosylmethionine-8-amino-7-oxononanoate aminotransferase (EC 2.6.1.62)	FIG00012547	icw(2);Biotin_biosynthesis_Experimental isu;Biotin_synthesis_cluster isu;Biotin_biosynthesis
NODE_5_length_258648_cov_42.850559	fig|6666666.34159.peg.1190	peg	NODE_5_length_258648_cov_42.850559_46085_45462	46085	45462	-	Dethiobiotin synthetase (EC 6.3.3.3)	FIG00000423	isu;Biotin_biosynthesis_Experimental icw(2);Biotin_synthesis_cluster icw(2);Biotin_biosynthesis
NODE_5_length_258648_cov_42.850559	fig|6666666.34159.peg.1191	peg	NODE_5_length_258648_cov_42.850559_46307_46101	46307	46101	-	hypothetical protein		
NODE_5_length_258648_cov_42.850559	fig|6666666.34159.peg.1192	peg	NODE_5_length_258648_cov_42.850559_46326_46487	46326	46487	+	hypothetical protein		
NODE_5_length_258648_cov_42.850559	fig|6666666.34159.peg.1193	peg	NODE_5_length_258648_cov_42.850559_46629_46745	46629	46745	+	hypothetical protein		
NODE_5_length_258648_cov_42.850559	fig|6666666.34159.peg.1194	peg	NODE_5_length_258648_cov_42.850559_47611_46859	47611	46859	-	Biotin synthesis protein BioC	FIG00033705	icw(1);Biotin_biosynthesis_Experimental icw(3);Biotin_synthesis_cluster icw(3);Biotin_biosynthesis
NODE_5_length_258648_cov_42.850559	fig|6666666.34159.peg.1195	peg	NODE_5_length_258648_cov_42.850559_48753_47590	48753	47590	-	8-amino-7-oxononanoate synthase (EC 2.3.1.47)	FIG00051001	icw(3);Biotin_biosynthesis_Experimental icw(1);Biotin_synthesis_cluster icw(1);Biotin_biosynthesis
NODE_5_length_258648_cov_42.850559	fig|6666666.34159.peg.1196	peg	NODE_5_length_258648_cov_42.850559_49190_48762	49190	48762	-	MutT/Nudix family protein	FIG01307837	ff
NODE_5_length_258648_cov_42.850559	fig|6666666.34159.peg.1197	peg	NODE_5_length_258648_cov_42.850559_50442_49183	50442	49183	-	3-oxoacyl-[acyl-carrier-protein] synthase, KASII (EC 2.3.1.41)	FIG00002246	isu;Fatty_Acid_Biosynthesis_FASII
NODE_5_length_258648_cov_42.850559	fig|6666666.34159.peg.1198	peg	NODE_5_length_258648_cov_42.850559_50839_50468	50839	50468	-	Iojap protein	FIG00135315	ff
NODE_5_length_258648_cov_42.850559	fig|6666666.34159.peg.1199	peg	NODE_5_length_258648_cov_42.850559_51594_50977	51594	50977	-	Nicotinate-nucleotide adenylyltransferase (EC 2.7.7.18)	FIG01956071	isu;NAD_and_NADP_cofactor_biosynthesis_global
NODE_5_length_258648_cov_42.850559	fig|6666666.34159.peg.1200	peg	NODE_5_length_258648_cov_42.850559_53784_51598	53784	51598	-	Glutamine synthetase type III, GlnN (EC 6.3.1.2)	FIG00132839	isu;Glutamine,_Glutamate,_Aspartate_and_Asparagine_Biosynthesis isu;Glutamine_synthetases
NODE_5_length_258648_cov_42.850559	fig|6666666.34159.peg.1201	peg	NODE_5_length_258648_cov_42.850559_54459_53803	54459	53803	-	Phosphoserine phosphatase (EC 3.1.3.3)	FIG00000149	idu(1);Glycine_and_Serine_Utilization idu(1);Serine_Biosynthesis
NODE_5_length_258648_cov_42.850559	fig|6666666.34159.peg.1202	peg	NODE_5_length_258648_cov_42.850559_56278_54560	56278	54560	-	hypothetical protein		
NODE_5_length_258648_cov_42.850559	fig|6666666.34159.peg.1203	peg	NODE_5_length_258648_cov_42.850559_57019_56537	57019	56537	-	NADPH-dependent FMN reductase		
NODE_5_length_258648_cov_42.850559	fig|6666666.34159.peg.1204	peg	NODE_5_length_258648_cov_42.850559_58164_57067	58164	57067	-	tRNA delta(2)-isopentenylpyrophosphate transferase (EC 2.5.1.8)	FIG00000196	ff
NODE_5_length_258648_cov_42.850559	fig|6666666.34159.peg.1205	peg	NODE_5_length_258648_cov_42.850559_58580_58224	58580	58224	-	anti-sigma F factor antagonist (spoIIAA-2); anti sigma b factor antagonist RsbV	FIG00103613	ff
NODE_5_length_258648_cov_42.850559	fig|6666666.34159.peg.1206	peg	NODE_5_length_258648_cov_42.850559_58991_58608	58991	58608	-	Single-stranded DNA-binding protein	FIG00077620	idu(1);DNA_repair,_bacterial idu(1);pVir_Plasmid_of_Campylobacter
NODE_5_length_258648_cov_42.850559	fig|6666666.34159.peg.1207	peg	NODE_5_length_258648_cov_42.850559_59016_59129	59016	59129	+	hypothetical protein		
NODE_5_length_258648_cov_42.850559	fig|6666666.34159.peg.1208	peg	NODE_5_length_258648_cov_42.850559_59348_59118	59348	59118	-	KH domain RNA binding protein YlqC	FIG00005628	idu(1);KH_domain_RNA_binding_protein_YlqC
NODE_5_length_258648_cov_42.850559	fig|6666666.34159.peg.1209	peg	NODE_5_length_258648_cov_42.850559_60014_59658	60014	59658	-	Periplasmic divalent cation tolerance protein CutA	FIG01955810	isu;Copper_homeostasis:_copper_tolerance
NODE_5_length_258648_cov_42.850559	fig|6666666.34159.peg.1210	peg	NODE_5_length_258648_cov_42.850559_60934_60029	60934	60029	-	hypothetical protein	FIG00638284	ff
NODE_5_length_258648_cov_42.850559	fig|6666666.34159.peg.1211	peg	NODE_5_length_258648_cov_42.850559_61689_60931	61689	60931	-	possible Glycosyl transferase	FIG01424684	ff
NODE_5_length_258648_cov_42.850559	fig|6666666.34159.peg.1212	peg	NODE_5_length_258648_cov_42.850559_64076_61710	64076	61710	-	UDP-N-acetylmuramate--alanine ligase (EC 6.3.2.8)	FIG00135554	ff
NODE_5_length_258648_cov_42.850559	fig|6666666.34159.peg.1213	peg	NODE_5_length_258648_cov_42.850559_65176_64073	65176	64073	-	UDP-N-acetylglucosamine--N-acetylmuramyl-(pentapeptide) pyrophosphoryl-undecaprenol N-acetylglucosamine transferase (EC 2.4.1.227)	FIG00047056	ff
NODE_5_length_258648_cov_42.850559	fig|6666666.34159.peg.1214	peg	NODE_5_length_258648_cov_42.850559_66830_65454	66830	65454	-	hypothetical protein		
NODE_5_length_258648_cov_42.850559	fig|6666666.34159.peg.1215	peg	NODE_5_length_258648_cov_42.850559_67470_66985	67470	66985	-	Methylated-DNA--protein-cysteine methyltransferase (EC 2.1.1.63)	FIG00000328	idu(1);DNA_repair,_bacterial idu(1);CBSS-393124.3.peg.2657
NODE_5_length_258648_cov_42.850559	fig|6666666.34159.peg.1216	peg	NODE_5_length_258648_cov_42.850559_68148_68783	68148	68783	+	hypothetical protein	FIG00638284	ff
NODE_5_length_258648_cov_42.850559	fig|6666666.34159.peg.1217	peg	NODE_5_length_258648_cov_42.850559_68945_69118	68945	69118	+	hypothetical protein		
NODE_5_length_258648_cov_42.850559	fig|6666666.34159.peg.1218	peg	NODE_5_length_258648_cov_42.850559_70350_69115	70350	69115	-	hypothetical protein		
NODE_5_length_258648_cov_42.850559	fig|6666666.34159.peg.1219	peg	NODE_5_length_258648_cov_42.850559_71506_70541	71506	70541	-	hypothetical protein		
NODE_5_length_258648_cov_42.850559	fig|6666666.34159.peg.1220	peg	NODE_5_length_258648_cov_42.850559_71701_72000	71701	72000	+	Aspartyl-tRNA(Asn) amidotransferase subunit C (EC 6.3.5.6) @ Glutamyl-tRNA(Gln) amidotransferase subunit C (EC 6.3.5.7)	FIG00000432	isu;tRNA_aminoacylation,_Asp_and_Asn icw(2);tRNA_aminoacylation,_Glu_and_Gln
NODE_5_length_258648_cov_42.850559	fig|6666666.34159.peg.1221	peg	NODE_5_length_258648_cov_42.850559_72012_73466	72012	73466	+	Aspartyl-tRNA(Asn) amidotransferase subunit A (EC 6.3.5.6) @ Glutamyl-tRNA(Gln) amidotransferase subunit A (EC 6.3.5.7)	FIG00000359	icw(2);tRNA_aminoacylation,_Asp_and_Asn isu;tRNA_aminoacylation,_Glu_and_Gln

NODE_5_length_258648_cov_42.850559	fig|6666666.34159.peg.1222	peg	NODE_5_length_258648_cov_42.850559_73477_74934	73477	74934	+	Aspartyl-tRNA(Asn) amidotransferase subunit B (EC 6.3.5.6) @ Glutamyl-tRNA(Gln) amidotransferase subunit B (EC 6.3.5.7)	FIG00000399	icw(1);tRNA_aminoacylation,_Asp_and_Asn icw(1);tRNA_aminoacylation,_Glu_and_Gln
NODE_5_length_258648_cov_42.850559	fig|6666666.34159.peg.1223	peg	NODE_5_length_258648_cov_42.850559_75047_76039	75047	76039	+	hypothetical protein		
NODE_5_length_258648_cov_42.850559	fig|6666666.34159.peg.1224	peg	NODE_5_length_258648_cov_42.850559_77891_76014	77891	76014	-	Lipid A export ATP-binding/permease protein MsbA	FIG00017059	ff
NODE_5_length_258648_cov_42.850559	fig|6666666.34159.peg.1225	peg	NODE_5_length_258648_cov_42.850559_78949_78002	78949	78002	-	Acetyl-coenzyme A carboxyl transferase alpha chain (EC 6.4.1.2)	FIG00000442	isu;Fatty_Acid_Biosynthesis_FASII
NODE_5_length_258648_cov_42.850559	fig|6666666.34159.peg.1226	peg	NODE_5_length_258648_cov_42.850559_79962_78973	79962	78973	-	FIG00899523: hypothetical protein	FIG00899522	ff
NODE_5_length_258648_cov_42.850559	fig|6666666.34159.peg.1227	peg	NODE_5_length_258648_cov_42.850559_81803_80289	81803	80289	-	FIG00899511: hypothetical protein	FIG00899510	ff
NODE_5_length_258648_cov_42.850559	fig|6666666.34159.peg.1228	peg	NODE_5_length_258648_cov_42.850559_82303_81995	82303	81995	-	Integration host factor alpha/beta	FIG00797231	isu;DNA_structural_proteins,_bacterial
NODE_5_length_258648_cov_42.850559	fig|6666666.34159.peg.1229	peg	NODE_5_length_258648_cov_42.850559_83903_82611	83903	82611	-	Tyrosyl-tRNA synthetase (EC 6.1.1.1)		isu;tRNA_aminoacylation,_Tyr
NODE_5_length_258648_cov_42.850559	fig|6666666.34159.peg.1230	peg	NODE_5_length_258648_cov_42.850559_84101_84253	84101	84253	+	hypothetical protein		
NODE_5_length_258648_cov_42.850559	fig|6666666.34159.peg.1231	peg	NODE_5_length_258648_cov_42.850559_84596_84712	84596	84712	+	hypothetical protein		
NODE_5_length_258648_cov_42.850559	fig|6666666.34159.peg.1232	peg	NODE_5_length_258648_cov_42.850559_85442_84687	85442	84687	-	ACR family	FIG01385081	ff
NODE_5_length_258648_cov_42.850559	fig|6666666.34159.peg.1233	peg	NODE_5_length_258648_cov_42.850559_85516_86199	85516	86199	+	Ribose 5-phosphate isomerase A (EC 5.3.1.6)	FIG00000415	isu;D-ribose_utilization isu;Calvin-Benson_cycle isu;Pentose_phosphate_pathway
NODE_5_length_258648_cov_42.850559	fig|6666666.34159.peg.1234	peg	NODE_5_length_258648_cov_42.850559_86256_86669	86256	86669	+	FIG00494004: hypothetical protein	FIG00493433	ff
NODE_5_length_258648_cov_42.850559	fig|6666666.34159.peg.1235	peg	NODE_5_length_258648_cov_42.850559_87022_86723	87022	86723	-	Phosphocarrier protein of PTS system	FIG01954533	ff
NODE_5_length_258648_cov_42.850559	fig|6666666.34159.peg.1236	peg	NODE_5_length_258648_cov_42.850559_87070_87183	87070	87183	+	hypothetical protein		
NODE_5_length_258648_cov_42.850559	fig|6666666.34159.peg.1237	peg	NODE_5_length_258648_cov_42.850559_87267_89078	87267	89078	+	Oligoendopeptidase F	FIG00628107	ff
NODE_5_length_258648_cov_42.850559	fig|6666666.34159.peg.1238	peg	NODE_5_length_258648_cov_42.850559_89202_89531	89202	89531	+	Heat shock protein 60 family co-chaperone GroES	FIG00009229	idu(1);GroEL_GroES
NODE_5_length_258648_cov_42.850559	fig|6666666.34159.peg.1239	peg	NODE_5_length_258648_cov_42.850559_89568_91190	89568	91190	+	Heat shock protein 60 family chaperone GroEL	FIG00000056	idu(2);Staphylococcal_pathogenicity_islands_SaPI icw(1);GroEL_GroES
NODE_5_length_258648_cov_42.850559	fig|6666666.34159.peg.1240	peg	NODE_5_length_258648_cov_42.850559_92002_91229	92002	91229	-	hypothetical protein		
NODE_5_length_258648_cov_42.850559	fig|6666666.34159.peg.1241	peg	NODE_5_length_258648_cov_42.850559_92508_92017	92508	92017	-	hypothetical protein		
NODE_5_length_258648_cov_42.850559	fig|6666666.34159.peg.1242	peg	NODE_5_length_258648_cov_42.850559_92627_93718	92627	93718	+	hypothetical protein		
NODE_5_length_258648_cov_42.850559	fig|6666666.34159.rna.18	rna	NODE_5_length_258648_cov_42.850559_93794_93865	93794	93865	+	tRNA-Asn-GTT		
NODE_5_length_258648_cov_42.850559	fig|6666666.34159.peg.1243	peg	NODE_5_length_258648_cov_42.850559_94698_94829	94698	94829	+	hypothetical protein		
NODE_5_length_258648_cov_42.850559	fig|6666666.34159.peg.1244	peg	NODE_5_length_258648_cov_42.850559_94874_95185	94874	95185	+	hypothetical protein		
NODE_5_length_258648_cov_42.850559	fig|6666666.34159.peg.1245	peg	NODE_5_length_258648_cov_42.850559_96699_95359	96699	95359	-	ATP-dependent DNA helicase		
NODE_5_length_258648_cov_42.850559	fig|6666666.34159.peg.1246	peg	NODE_5_length_258648_cov_42.850559_97136_97312	97136	97312	+	hypothetical protein		
NODE_5_length_258648_cov_42.850559	fig|6666666.34159.peg.1247	peg	NODE_5_length_258648_cov_42.850559_98703_97822	98703	97822	-	Ribosomal RNA large subunit methyltransferase F (EC 2.1.1.51)	FIG00005454	isu;RNA_methylation
NODE_5_length_258648_cov_42.850559	fig|6666666.34159.peg.1248	peg	NODE_5_length_258648_cov_42.850559_99006_98782	99006	98782	-	hypothetical protein		
NODE_5_length_258648_cov_42.850559	fig|6666666.34159.peg.1249	peg	NODE_5_length_258648_cov_42.850559_100291_99203	100291	99203	-	hypothetical protein		
NODE_5_length_258648_cov_42.850559	fig|6666666.34159.peg.1250	peg	NODE_5_length_258648_cov_42.850559_100423_100578	100423	1E+05	+	hypothetical protein	FIG00638284	ff
NODE_5_length_258648_cov_42.850559	fig|6666666.34159.peg.1251	peg	NODE_5_length_258648_cov_42.850559_100856_101404	100856	1E+05	+	probable yciF protein		
NODE_5_length_258648_cov_42.850559	fig|6666666.34159.peg.1252	peg	NODE_5_length_258648_cov_42.850559_101370_101507	101370	1E+05	+	hypothetical protein		
NODE_5_length_258648_cov_42.850559	fig|6666666.34159.peg.1253	peg	NODE_5_length_258648_cov_42.850559_101641_102141	101641	1E+05	+	hypothetical protein	FIG00638284	ff
NODE_5_length_258648_cov_42.850559	fig|6666666.34159.peg.1254	peg	NODE_5_length_258648_cov_42.850559_102207_103334	102207	1E+05	+	hypothetical protein		
NODE_5_length_258648_cov_42.850559	fig|6666666.34159.peg.1255	peg	NODE_5_length_258648_cov_42.850559_103345_103743	103345	1E+05	+	MazG (nucleoside triphosphate pyrophosphohydrolase)		
NODE_5_length_258648_cov_42.850559	fig|6666666.34159.peg.1256	peg	NODE_5_length_258648_cov_42.850559_104363_103740	104363	1E+05	-	hypothetical protein		
NODE_5_length_258648_cov_42.850559	fig|6666666.34159.peg.1257	peg	NODE_5_length_258648_cov_42.850559_104531_106321	104531	1E+05	+	hypothetical protein	FIG00638284	ff
NODE_5_length_258648_cov_42.850559	fig|6666666.34159.peg.1258	peg	NODE_5_length_258648_cov_42.850559_106787_106401	106787	1E+05	-	Ribosomal-protein-S5p-alanine acetyltransferase	FIG00000756	isu;Ribosomal_protein_S5p_acylation
NODE_5_length_258648_cov_42.850559	fig|6666666.34159.peg.1259	peg	NODE_5_length_258648_cov_42.850559_108436_107009	108436	1E+05	-	hypothetical protein		
NODE_5_length_258648_cov_42.850559	fig|6666666.34159.peg.1260	peg	NODE_5_length_258648_cov_42.850559_109720_108677	109720	1E+05	-	UDP-glucose 4-epimerase (EC 5.1.3.2)	FIG00022300	idu(1);Rhamnose_containing_glycans idu(1);CBSS-296591.1.peg.2330 idu(1);Lactose_and_Galactose_Uptake_and_Utili
NODE_5_length_258648_cov_42.850559	fig|6666666.34159.peg.1261	peg	NODE_5_length_258648_cov_42.850559_110836_109844	110836	1E+05	-	putative endonuclease/exonuclease/phosphatase family protein		
NODE_5_length_258648_cov_42.850559	fig|6666666.34159.peg.1262	peg	NODE_5_length_258648_cov_42.850559_111126_110947	111126	1E+05	-	hypothetical protein		
NODE_5_length_258648_cov_42.850559	fig|6666666.34159.peg.1263	peg	NODE_5_length_258648_cov_42.850559_111284_111141	111284	1E+05	-	hypothetical protein		
NODE_5_length_258648_cov_42.850559	fig|6666666.34159.peg.1264	peg	NODE_5_length_258648_cov_42.850559_112912_111710	112912	1E+05	-	Cyclopropane-fatty-acyl-phospholipid synthase (EC 2.1.1.79)	FIG00001555	ff
NODE_5_length_258648_cov_42.850559	fig|6666666.34159.peg.1265	peg	NODE_5_length_258648_cov_42.850559_113107_122196	113107	1E+05	+	Cyclic beta-1,2-glucan synthase (EC 2.4.1.-)	FIG00031723	isu;Synthesis_of_osmoregulated_periplasmic_glucans
NODE_5_length_258648_cov_42.850559	fig|6666666.34159.peg.1266	peg	NODE_5_length_258648_cov_42.850559_122366_122980	122366	1E+05	+	hypothetical protein		
NODE_5_length_258648_cov_42.850559	fig|6666666.34159.peg.1267	peg	NODE_5_length_258648_cov_42.850559_123289_125958	123289	1E+05	+	hypothetical protein	FIG00638284	ff
NODE_5_length_258648_cov_42.850559	fig|6666666.34159.peg.1268	peg	NODE_5_length_258648_cov_42.850559_128232_125965	128232	1E+05	-	Lead, cadmium, zinc and mercury transporting ATPase (EC 3.6.3.3) (EC 3.6.3.5); Copper-translocating P-type ATPase (EC 3.6.3.4)	FIG00658111	idu(3);Copper_Transport_System idu(3);CBSS-196620.1.peg.2477 idu(3);Copper_homeostasis
NODE_5_length_258648_cov_42.850559	fig|6666666.34159.peg.1269	peg	NODE_5_length_258648_cov_42.850559_128333_128866	128333	1E+05	+	Peptide deformylase (EC 3.5.1.88)	FIG00000017	idu(1);CBSS-89187.3.peg.2957 idu(1);Translation_termination_factors_bacterial
NODE_5_length_258648_cov_42.850559	fig|6666666.34159.peg.1270	peg	NODE_5_length_258648_cov_42.850559_128933_130282	128933	1E+05	+	Mg/Co/Ni transporter MgtE / CBS domain	FIG00437800	idu(2);Magnesium_transport
NODE_5_length_258648_cov_42.850559	fig|6666666.34159.peg.1271	peg	NODE_5_length_258648_cov_42.850559_130318_130641	130318	1E+05	+	hypothetical protein		
NODE_5_length_258648_cov_42.850559	fig|6666666.34159.peg.1272	peg	NODE_5_length_258648_cov_42.850559_133115_130680	133115	1E+05	-	hypothetical protein		
NODE_5_length_258648_cov_42.850559	fig|6666666.34159.peg.1273	peg	NODE_5_length_258648_cov_42.850559_134247_135167	134247	1E+05	+	hypothetical protein		
NODE_5_length_258648_cov_42.850559	fig|6666666.34159.peg.1274	peg	NODE_5_length_258648_cov_42.850559_138335_135210	138335	1E+05	-	Isoleucyl-tRNA synthetase (EC 6.1.1.5)	FIG00000085	isu;tRNA_aminoacylation,_Ile
NODE_5_length_258648_cov_42.850559	fig|6666666.34159.peg.1275	peg	NODE_5_length_258648_cov_42.850559_138572_140476	138572	1E+05	+	Asparagine synthetase [glutamine-hydrolyzing] (EC 6.3.5.4)	FIG00035175	idu(1);Glutamine,_Glutamate,_Aspartate_and_Asparagine_Biosynthesis idu(1);Glutamate_and_Aspartate_uptake_in_Bact
NODE_5_length_258648_cov_42.850559	fig|6666666.34159.peg.1276	peg	NODE_5_length_258648_cov_42.850559_140499_141467	140499	1E+05	+	hypothetical protein		
NODE_5_length_258648_cov_42.850559	fig|6666666.34159.peg.1277	peg	NODE_5_length_258648_cov_42.850559_141649_144714	141649	1E+05	+	hypothetical protein	FIG00638284	ff
NODE_5_length_258648_cov_42.850559	fig|6666666.34159.peg.1278	peg	NODE_5_length_258648_cov_42.850559_145871_145368	145871	1E+05	-	hypothetical protein	FIG00638284	ff
NODE_5_length_258648_cov_42.850559	fig|6666666.34159.peg.1279	peg	NODE_5_length_258648_cov_42.850559_146533_145868	146533	1E+05	-	Nucleoside triphosphate pyrophosphohydrolase MazG (EC 3.6.1.8)	FIG00002974	icw(1);Nucleoside_triphosphate_pyrophosphohydrolase_MazG

NODE_5_length_258648_cov_42.850559	fig|6666666.34159.peg.1280	peg	NODE_5_length_258648_cov_42.850559_146646_146530	146646	1E+05	-	Nucleoside triphosphate pyrophosphohydrolase MazG (EC 3.6.1.8)	FIG00002974	icw(1);Nucleoside_triphosphate_pyrophosphohydrolase_MazG
NODE_5_length_258648_cov_42.850559	fig|6666666.34159.peg.1281	peg	NODE_5_length_258648_cov_42.850559_146658_147773	146658	1E+05	+	BioD-like N-terminal domain of phosphotransacetylase		
NODE_5_length_258648_cov_42.850559	fig|6666666.34159.peg.1282	peg	NODE_5_length_258648_cov_42.850559_149014_147770	149014	1E+05	-	hypothetical protein		
NODE_5_length_258648_cov_42.850559	fig|6666666.34159.peg.1283	peg	NODE_5_length_258648_cov_42.850559_150099_149050	150099	1E+05	-	Lipopolysaccharide heptosyltransferase I (EC 2.4.1.-)	FIG00133231	isu;LOS_core_oligosaccharide_biosynthesis
NODE_5_length_258648_cov_42.850559	fig|6666666.34159.peg.1284	peg	NODE_5_length_258648_cov_42.850559_150983_150096	150983	2E+05	-	Ribosomal large subunit pseudouridine synthase D (EC 4.2.1.70)	FIG00000124	idu(1);RNA_pseudouridine_syntheses
NODE_5_length_258648_cov_42.850559	fig|6666666.34159.peg.1285	peg	NODE_5_length_258648_cov_42.850559_152015_150987	152015	2E+05	-	Phenylalanyl-tRNA synthetase alpha chain (EC 6.1.1.20)	FIG00000098	isu;tRNA_aminoacylation,_Phe
NODE_5_length_258648_cov_42.850559	fig|6666666.34159.peg.1286	peg	NODE_5_length_258648_cov_42.850559_152504_152148	152504	2E+05	-	LSU ribosomal protein L20p	FIG00000216	icw(2);Mycobacterium_virulence_operon_involved_in_protein_synthesis_(LSU_ribosomal_proteins)
NODE_5_length_258648_cov_42.850559	fig|6666666.34159.peg.1287	peg	NODE_5_length_258648_cov_42.850559_152776_152582	152776	2E+05	-	LSU ribosomal protein L35p	FIG00000259	isu;Mycobacterium_virulence_operon_involved_in_protein_synthesis_(LSU_ribosomal_proteins)
NODE_5_length_258648_cov_42.850559	fig|6666666.34159.peg.1288	peg	NODE_5_length_258648_cov_42.850559_153380_152811	153380	2E+05	-	Translation initiation factor 3	FIG00000206	isu;Translation_initiation_factors_bacterial icw(1);Mycobacterium_virulence_operon_involved_in_protein_synthesis_(LSU_ribosomal_proteins
NODE_5_length_258648_cov_42.850559	fig|6666666.34159.peg.1289	peg	NODE_5_length_258648_cov_42.850559_155017_153401	155017	2E+05	-	Threonyl-tRNA synthetase (EC 6.1.1.3)	FIG00000101	idu(1);tRNA_aminoacylation,_Thr
NODE_5_length_258648_cov_42.850559	fig|6666666.34159.rna.19	rna	NODE_5_length_258648_cov_42.850559_155218_155145	155218	2E+05	-	tRNA-Val-GAC		isu;tRNAs
NODE_5_length_258648_cov_42.850559	fig|6666666.34159.peg.1290	peg	NODE_5_length_258648_cov_42.850559_155964_155356	155964	2E+05	-	hypothetical protein		
NODE_5_length_258648_cov_42.850559	fig|6666666.34159.peg.1291	peg	NODE_5_length_258648_cov_42.850559_156415_157035	156415	2E+05	+	hypothetical protein		
NODE_5_length_258648_cov_42.850559	fig|6666666.34159.peg.1292	peg	NODE_5_length_258648_cov_42.850559_157217_157351	157217	2E+05	+	hypothetical protein		
NODE_5_length_258648_cov_42.850559	fig|6666666.34159.peg.1293	peg	NODE_5_length_258648_cov_42.850559_157576_157439	157576	2E+05	-	hypothetical protein		
NODE_5_length_258648_cov_42.850559	fig|6666666.34159.peg.1294	peg	NODE_5_length_258648_cov_42.850559_161882_157689	161882	2E+05	-	hypothetical protein	FIG00638284	ff
NODE_5_length_258648_cov_42.850559	fig|6666666.34159.peg.1295	peg	NODE_5_length_258648_cov_42.850559_163467_162148	163467	2E+05	-	Glutamate-1-semialdehyde aminotransferase (EC 5.4.3.8)	FIG00000338	isu;CBSS-196164.1.peg.461 isu;Heme_and_Siroheme_Biosynthesis
NODE_5_length_258648_cov_42.850559	fig|6666666.34159.peg.1296	peg	NODE_5_length_258648_cov_42.850559_163948_163472	163948	2E+05	-	Low molecular weight protein tyrosine phosphatase (EC 3.1.3.48)	FIG00059124	isu;Protein_deglycation isu;LMPTP_YfkJ_cluster isu;CBSS-176280.1.peg.1561
NODE_5_length_258648_cov_42.850559	fig|6666666.34159.peg.1297	peg	NODE_5_length_258648_cov_42.850559_165958_163967	165958	2E+05	-	Serine/threonine kinase	FIG00659720	ff
NODE_5_length_258648_cov_42.850559	fig|6666666.34159.peg.1298	peg	NODE_5_length_258648_cov_42.850559_166829_165915	166829	2E+05	-	hypothetical protein		
NODE_5_length_258648_cov_42.850559	fig|6666666.34159.peg.1299	peg	NODE_5_length_258648_cov_42.850559_167045_169567	167045	2E+05	+	Inner membrane protein translocase component YidC, long form / Inner membrane protein translocase component YidC, short form OxaI-like	FIG00229531	isu;CTP_synthase_(EC_6.3.4.2)_cluster isu;RNA_modification_cluster isu;RNA_modification_cluster
NODE_5_length_258648_cov_42.850559	fig|6666666.34159.peg.1300	peg	NODE_5_length_258648_cov_42.850559_169797_171191	169797	2E+05	+	Chromosomal replication initiator protein DnaA	FIG00000441	idu(1);DNA_replication_cluster_1
NODE_5_length_258648_cov_42.850559	fig|6666666.34159.peg.1301	peg	NODE_5_length_258648_cov_42.850559_171200_172528	171200	2E+05	+	Adenosylhomocysteinase (EC 3.3.1.1)	FIG00000858	isu;Methionine_Degradation
NODE_5_length_258648_cov_42.850559	fig|6666666.34159.rna.20	rna	NODE_5_length_258648_cov_42.850559_172786_172860	172786	2E+05	+	tRNA-Pro-TGG		
NODE_5_length_258648_cov_42.850559	fig|6666666.34159.peg.1302	peg	NODE_5_length_258648_cov_42.850559_172927_174051	172927	2E+05	+	Prolipoprotein diacylglyceryl transferase (EC 2.4.99.-)	FIG00000985	isu;Lipoprotein_Biosynthesis
NODE_5_length_258648_cov_42.850559	fig|6666666.34159.peg.1303	peg	NODE_5_length_258648_cov_42.850559_174139_174411	174139	2E+05	+	Ferredoxin	FIG00001676	isu;Soluble_cytochromes_and_functionally_related_electron_carriers isu;Inorganic_Sulfur_Assimilation
NODE_5_length_258648_cov_42.850559	fig|6666666.34159.peg.1304	peg	NODE_5_length_258648_cov_42.850559_174443_175030	174443	2E+05	+	hypothetical protein	FIG00638284	ff
NODE_5_length_258648_cov_42.850559	fig|6666666.34159.peg.1305	peg	NODE_5_length_258648_cov_42.850559_176160_175027	176160	2E+05	-	hypothetical protein		
NODE_5_length_258648_cov_42.850559	fig|6666666.34159.peg.1306	peg	NODE_5_length_258648_cov_42.850559_176971_176177	176971	2E+05	-	3-oxoacyl-[acyl-carrier protein] reductase (EC 1.1.1.100)	FIG00621114	idu(12);CBSS-246196.1.peg.364 idu(12);Fatty_Acid_Biosynthesis_FASII
NODE_5_length_258648_cov_42.850559	fig|6666666.34159.peg.1307	peg	NODE_5_length_258648_cov_42.850559_177265_176999	177265	2E+05	-	hypothetical protein		
NODE_5_length_258648_cov_42.850559	fig|6666666.34159.peg.1308	peg	NODE_5_length_258648_cov_42.850559_177846_177358	177846	2E+05	-	protein of unknown function DUF296		
NODE_5_length_258648_cov_42.850559	fig|6666666.34159.peg.1309	peg	NODE_5_length_258648_cov_42.850559_178202_177912	178202	2E+05	-	hypothetical protein		
NODE_5_length_258648_cov_42.850559	fig|6666666.34159.peg.1310	peg	NODE_5_length_258648_cov_42.850559_179595_178213	179595	2E+05	-	Succinate-semialdehyde dehydrogenase [NAD] (EC 1.2.1.24); Succinate-semialdehyde dehydrogenase [NADP+] (EC 1.2.1.16)	FIG01959002	ff
NODE_5_length_258648_cov_42.850559	fig|6666666.34159.peg.1311	peg	NODE_5_length_258648_cov_42.850559_180647_180402	180647	2E+05	-	putative transposase	FIG01331389	ff
NODE_5_length_258648_cov_42.850559	fig|6666666.34159.peg.1312	peg	NODE_5_length_258648_cov_42.850559_182230_180743	182230	2E+05	-	hypothetical protein		
NODE_5_length_258648_cov_42.850559	fig|6666666.34159.peg.1313	peg	NODE_5_length_258648_cov_42.850559_182677_182555	182677	2E+05	-	hypothetical protein		
NODE_5_length_258648_cov_42.850559	fig|6666666.34159.peg.1314	peg	NODE_5_length_258648_cov_42.850559_182963_182709	182963	2E+05	-	hypothetical protein		
NODE_5_length_258648_cov_42.850559	fig|6666666.34159.peg.1315	peg	NODE_5_length_258648_cov_42.850559_183991_183449	183991	2E+05	-	hypothetical protein		
NODE_5_length_258648_cov_42.850559	fig|6666666.34159.peg.1316	peg	NODE_5_length_258648_cov_42.850559_185472_184069	185472	2E+05	-	Dihydrolipoamide dehydrogenase of 2-oxoglutarate dehydrogenase (EC 1.8.1.4)	FIG00013010	isu;TCA_Cycle isu;Dehydrogenase_complexes
NODE_5_length_258648_cov_42.850559	fig|6666666.34159.peg.1317	peg	NODE_5_length_258648_cov_42.850559_186655_185474	186655	2E+05	-	Dihydrolipoamide succinyltransferase component (E2) of 2-oxoglutarate dehydrogenase complex (EC 2.3.1.61)	FIG00127747	icw(1);TCA_Cycle icw(1);Dehydrogenase_complexes
NODE_5_length_258648_cov_42.850559	fig|6666666.34159.peg.1318	peg	NODE_5_length_258648_cov_42.850559_189422_186675	189422	2E+05	-	2-oxoglutarate dehydrogenase E1 component (EC 1.2.4.2)	FIG00000541	icw(2);TCA_Cycle icw(2);Dehydrogenase_complexes
NODE_5_length_258648_cov_42.850559	fig|6666666.34159.peg.1319	peg	NODE_5_length_258648_cov_42.850559_189605_190258	189605	2E+05	+	unknown protein	FIG00764722	ff
NODE_5_length_258648_cov_42.850559	fig|6666666.34159.peg.1320	peg	NODE_5_length_258648_cov_42.850559_190255_192888	190255	2E+05	+	hypothetical protein	FIG00638284	ff
NODE_5_length_258648_cov_42.850559	fig|6666666.34159.peg.1321	peg	NODE_5_length_258648_cov_42.850559_192968_193705	192968	2E+05	+	hypothetical protein		
NODE_5_length_258648_cov_42.850559	fig|6666666.34159.peg.1322	peg	NODE_5_length_258648_cov_42.850559_195530_193686	195530	2E+05	-	Excinuclease ABC subunit C	FIG00000185	isu;DNA_repair,_UvrABC_system
NODE_5_length_258648_cov_42.850559	fig|6666666.34159.peg.1323	peg	NODE_5_length_258648_cov_42.850559_196014_195532	196014	2E+05	-	hypothetical protein		
NODE_5_length_258648_cov_42.850559	fig|6666666.34159.peg.1324	peg	NODE_5_length_258648_cov_42.850559_197130_196078	197130	2E+05	-	Ribosomal RNA large subunit methyltransferase N (EC 2.1.1.-)	FIG00000293	idu(1);RNA_methylation
NODE_5_length_258648_cov_42.850559	fig|6666666.34159.peg.1325	peg	NODE_5_length_258648_cov_42.850559_197517_197630	197517	2E+05	+	hypothetical protein		
NODE_5_length_258648_cov_42.850559	fig|6666666.34159.peg.1326	peg	NODE_5_length_258648_cov_42.850559_197680_198330	197680	2E+05	+	hypothetical protein		
NODE_5_length_258648_cov_42.850559	fig|6666666.34159.peg.1327	peg	NODE_5_length_258648_cov_42.850559_199238_198381	199238	2E+05	-	putative sugar transferase		
NODE_5_length_258648_cov_42.850559	fig|6666666.34159.peg.1328	peg	NODE_5_length_258648_cov_42.850559_201067_199265	201067	2E+05	-	DNA primase (EC 2.7.7.-)	FIG00019251	isu;CBSS-349161.4.peg.2417
NODE_5_length_258648_cov_42.850559	fig|6666666.34159.peg.1329	peg	NODE_5_length_258648_cov_42.850559_201206_201069	201206	2E+05	-	hypothetical protein		
NODE_5_length_258648_cov_42.850559	fig|6666666.34159.peg.1330	peg	NODE_5_length_258648_cov_42.850559_208064_201360	208064	2E+05	-	Long-chain-fatty-acid--CoA ligase (EC 6.2.1.3)	FIG00018699	idu(5);n-Phenylalkanoic_acid_degradation idu(5);Fatty_acid_metabolism_cluster idu(5);Biotin_synthesis_cluster
NODE_5_length_258648_cov_42.850559	fig|6666666.34159.peg.1331	peg	NODE_5_length_258648_cov_42.850559_217414_208061	217414	2E+05	-	Long-chain-fatty-acid--CoA ligase (EC 6.2.1.3)	FIG00018699	idu(5);n-Phenylalkanoic_acid_degradation idu(5);Fatty_acid_metabolism_cluster idu(5);Biotin_synthesis_cluster
NODE_5_length_258648_cov_42.850559	fig|6666666.34159.peg.1332	peg	NODE_5_length_258648_cov_42.850559_218412_217405	218412	2E+05	-	O-methyltransferase, family 2		
NODE_5_length_258648_cov_42.850559	fig|6666666.34159.peg.1333	peg	NODE_5_length_258648_cov_42.850559_218520_218825	218520	2E+05	+	hypothetical protein		
NODE_5_length_258648_cov_42.850559	fig|6666666.34159.peg.1334	peg	NODE_5_length_258648_cov_42.850559_218874_219827	218874	2E+05	+	SyrP-like protein	FIG00461509	ff
NODE_5_length_258648_cov_42.850559	fig|6666666.34159.peg.1335	peg	NODE_5_length_258648_cov_42.850559_219831_221192	219831	2E+05	+	kynurenine 3-monooxygenase(EC:1.14.13.9)		
NODE_5_length_258648_cov_42.850559	fig|6666666.34159.peg.1336	peg	NODE_5_length_258648_cov_42.850559_221189_221968	221189	2E+05	+	hypothetical protein		

NODE_5_length_258648_cov_42.850559	fig|6666666.34159.peg.1337	peg	NODE_5_length_258648_cov_42.850559_221982_222557	221982	2E+05	+	FIG006220: Hypothetical MbtH-like protein		
NODE_5_length_258648_cov_42.850559	fig|6666666.34159.peg.1338	peg	NODE_5_length_258648_cov_42.850559_223254_222547	223254	2E+05	-	4'-phosphopantetheinyl transferase (EC 2.7.8.-)		isu;Fatty_Acid_Biosynthesis_FASII
NODE_5_length_258648_cov_42.850559	fig|6666666.34159.peg.1339	peg	NODE_5_length_258648_cov_42.850559_224310_223270	224310	2E+05	-	Low-specificity L-threonine aldolase (EC 4.1.2.5)	FIG00000679	isu;Threonine_degradation isu;Glycine_Biosynthesis
NODE_5_length_258648_cov_42.850559	fig|6666666.34159.peg.1340	peg	NODE_5_length_258648_cov_42.850559_224952_224323	224952	2E+05	-	putative transporter		
NODE_5_length_258648_cov_42.850559	fig|6666666.34159.peg.1341	peg	NODE_5_length_258648_cov_42.850559_226705_224969	226705	2E+05	-	ABC transporter, ATP-binding/permease protein		
NODE_5_length_258648_cov_42.850559	fig|6666666.34159.peg.1342	peg	NODE_5_length_258648_cov_42.850559_229359_226690	229359	2E+05	-	Argininosuccinate lyase (EC 4.3.2.1)		
NODE_5_length_258648_cov_42.850559	fig|6666666.34159.peg.1343	peg	NODE_5_length_258648_cov_42.850559_230368_229352	230368	2E+05	-	Cysteine synthase (EC 2.5.1.47)	FIG00064110	isu;Cysteine_Biosynthesis
NODE_5_length_258648_cov_42.850559	fig|6666666.34159.peg.1344	peg	NODE_5_length_258648_cov_42.850559_231232_230384	231232	2E+05	-	Threonine kinase in B12 biosynthesis	FIG00002129	isu;Propanediol_utilization
NODE_5_length_258648_cov_42.850559	fig|6666666.34159.peg.1345	peg	NODE_5_length_258648_cov_42.850559_234837_231238	234837	2E+05	-	Long-chain-fatty-acid--CoA ligase (EC 6.2.1.3)	FIG00018699	idu(5);n-Phenylalkanoic_acid_degradation idu(5);Fatty_acid_metabolism_cluster idu(5);Biotin_synthesis_cluster
NODE_5_length_258648_cov_42.850559	fig|6666666.34159.peg.1346	peg	NODE_5_length_258648_cov_42.850559_234856_235077	234856	2E+05	+	hypothetical protein		
NODE_5_length_258648_cov_42.850559	fig|6666666.34159.peg.1347	peg	NODE_5_length_258648_cov_42.850559_239145_235102	239145	2E+05	-	hypothetical protein	FIG00638284	ff
NODE_5_length_258648_cov_42.850559	fig|6666666.34159.peg.1348	peg	NODE_5_length_258648_cov_42.850559_239613_239179	239613	2E+05	-	hypothetical protein		
NODE_5_length_258648_cov_42.850559	fig|6666666.34159.peg.1349	peg	NODE_5_length_258648_cov_42.850559_240046_239633	240046	2E+05	-	hypothetical protein		
NODE_5_length_258648_cov_42.850559	fig|6666666.34159.peg.1350	peg	NODE_5_length_258648_cov_42.850559_242836_240056	242836	2E+05	-	Single-stranded DNA-binding protein	FIG00077620	idu(1);DNA_repair,_bacterial idu(1);pVir_Plasmid_of_Campylobacter
NODE_5_length_258648_cov_42.850559	fig|6666666.34159.peg.1351	peg	NODE_5_length_258648_cov_42.850559_243447_242833	243447	2E+05	-	Chemotaxis signal transduction protein		
NODE_5_length_258648_cov_42.850559	fig|6666666.34159.peg.1352	peg	NODE_5_length_258648_cov_42.850559_244712_243471	244712	2E+05	-	Chemotaxis protein methyltransferase CheR (EC 2.1.1.80)		
NODE_5_length_258648_cov_42.850559	fig|6666666.34159.peg.1353	peg	NODE_5_length_258648_cov_42.850559_245171_244734	245171	2E+05	-	COG0835: Chemotaxis signal transduction protein		
NODE_5_length_258648_cov_42.850559	fig|6666666.34159.peg.1354	peg	NODE_5_length_258648_cov_42.850559_245455_245225	245455	2E+05	-	hypothetical protein		
NODE_5_length_258648_cov_42.850559	fig|6666666.34159.peg.1355	peg	NODE_5_length_258648_cov_42.850559_245734_246954	245734	2E+05	+	hypothetical protein		
NODE_5_length_258648_cov_42.850559	fig|6666666.34159.peg.1356	peg	NODE_5_length_258648_cov_42.850559_246951_248333	246951	2E+05	+	Ornithine aminotransferase (EC 2.6.1.13); Succinylornithine transaminase (EC 2.6.1.81); Acetylornithine aminotransferase (EC 2.6.1.11); N-succinyl-L,L-diaminopimelate	FIG01303969	isu;Lysine_Biosynthesis_DAP_Pathway,_GJO_scratch isu;Dimethylarginine_metabolism isu;Lysine_Biosynthesis_DAP_Pathway
NODE_5_length_258648_cov_42.850559	fig|6666666.34159.peg.1357	peg	NODE_5_length_258648_cov_42.850559_248337_249353	248337	2E+05	+	Arginine N-succinyltransferase (EC 2.3.1.109)		
NODE_5_length_258648_cov_42.850559	fig|6666666.34159.peg.1358	peg	NODE_5_length_258648_cov_42.850559_249367_250839	249367	3E+05	+	Succinylglutamic semialdehyde dehydrogenase (EC 1.2.1.71)	FIG00014703	ff
NODE_5_length_258648_cov_42.850559	fig|6666666.34159.peg.1359	peg	NODE_5_length_258648_cov_42.850559_250848_252203	250848	3E+05	+	Succinylarginine dihydrolase (EC 3.5.3.23)	FIG00003226	ff
NODE_5_length_258648_cov_42.850559	fig|6666666.34159.peg.1360	peg	NODE_5_length_258648_cov_42.850559_253666_252200	253666	3E+05	-	Mercuric ion reductase (EC 1.16.1.1)	FIG00002773	isu;Mercuric_reductase isu;Mercury_resistance_operon
NODE_5_length_258648_cov_42.850559	fig|6666666.34159.peg.1361	peg	NODE_5_length_258648_cov_42.850559_253845_254267	253845	3E+05	+	Osmotically inducible protein C	FIG00510640	ff
NODE_5_length_258648_cov_42.850559	fig|6666666.34159.peg.1362	peg	NODE_5_length_258648_cov_42.850559_254371_257238	254371	3E+05	+	Fe-S protein, homolog of lactate dehydrogenase SO1521	FIG00001220	ff
NODE_5_length_258648_cov_42.850559	fig|6666666.34159.peg.1363	peg	NODE_5_length_258648_cov_42.850559_257377_258300	257377	3E+05	+	hypothetical protein		
NODE_64_length_203_cov_92.477066	fig|6666666.34159.peg.1364	peg	NODE_64_length_203_cov_92.477066_139_2	139	2	-	hypothetical protein		
NODE_6_length_1611_cov_42.456821	fig|6666666.34159.peg.1365	peg	NODE_6_length_1611_cov_42.456821_719_342	719	342	-	hypothetical protein		
NODE_6_length_1611_cov_42.456821	fig|6666666.34159.peg.1366	peg	NODE_6_length_1611_cov_42.456821_906_787	906	787	-	hypothetical protein		
NODE_6_length_1611_cov_42.456821	fig|6666666.34159.peg.1367	peg	NODE_6_length_1611_cov_42.456821_1583_882	1583	882	-	COG3293: Transposase and inactivated derivatives		
NODE_71_length_58179_cov_42.447174	fig|6666666.34159.peg.1368	peg	NODE_71_length_58179_cov_42.447174_16_1386	16	1386	+	Rhs family protein-like		
NODE_71_length_58179_cov_42.447174	fig|6666666.34159.peg.1369	peg	NODE_71_length_58179_cov_42.447174_1386_1619	1386	1619	+	hypothetical protein		
NODE_71_length_58179_cov_42.447174	fig|6666666.34159.peg.1370	peg	NODE_71_length_58179_cov_42.447174_1957_2601	1957	2601	+	hypothetical protein		
NODE_71_length_58179_cov_42.447174	fig|6666666.34159.peg.1371	peg	NODE_71_length_58179_cov_42.447174_3084_3554	3084	3554	+	hypothetical protein		
NODE_71_length_58179_cov_42.447174	fig|6666666.34159.peg.1372	peg	NODE_71_length_58179_cov_42.447174_3544_4020	3544	4020	+	hypothetical protein		
NODE_71_length_58179_cov_42.447174	fig|6666666.34159.peg.1373	peg	NODE_71_length_58179_cov_42.447174_4870_4127	4870	4127	-	transposase, Mutator family		
NODE_71_length_58179_cov_42.447174	fig|6666666.34159.peg.1374	peg	NODE_71_length_58179_cov_42.447174_5355_5134	5355	5134	-	Transposase, mutator type		
NODE_71_length_58179_cov_42.447174	fig|6666666.34159.peg.1375	peg	NODE_71_length_58179_cov_42.447174_5803_5462	5803	5462	-	hypothetical protein		
NODE_71_length_58179_cov_42.447174	fig|6666666.34159.peg.1376	peg	NODE_71_length_58179_cov_42.447174_6814_6263	6814	6263	-	transposase		
NODE_71_length_58179_cov_42.447174	fig|6666666.34159.peg.1377	peg	NODE_71_length_58179_cov_42.447174_7156_7317	7156	7317	+	hypothetical protein		
NODE_71_length_58179_cov_42.447174	fig|6666666.34159.peg.1378	peg	NODE_71_length_58179_cov_42.447174_7417_7536	7417	7536	+	hypothetical protein		
NODE_71_length_58179_cov_42.447174	fig|6666666.34159.peg.1379	peg	NODE_71_length_58179_cov_42.447174_8421_7558	8421	7558	-	hypothetical protein		
NODE_71_length_58179_cov_42.447174	fig|6666666.34159.peg.1380	peg	NODE_71_length_58179_cov_42.447174_8638_8390	8638	8390	-	hypothetical protein		
NODE_71_length_58179_cov_42.447174	fig|6666666.34159.peg.1381	peg	NODE_71_length_58179_cov_42.447174_8741_9304	8741	9304	+	aminoglycoside N(6')acetyltransferase		
NODE_71_length_58179_cov_42.447174	fig|6666666.34159.peg.1382	peg	NODE_71_length_58179_cov_42.447174_9557_9306	9557	9306	-	ATP synthase epsilon chain (EC 3.6.3.14)	FIG00000249	icw(5);F0F1-type_ATP_synthase
NODE_71_length_58179_cov_42.447174	fig|6666666.34159.peg.1383	peg	NODE_71_length_58179_cov_42.447174_10959_9550	10959	9550	-	ATP synthase beta chain (EC 3.6.3.14)	FIG00040241	icw(3);F0F1-type_ATP_synthase
NODE_71_length_58179_cov_42.447174	fig|6666666.34159.peg.1384	peg	NODE_71_length_58179_cov_42.447174_11835_10975	11835	10975	-	ATP synthase gamma chain (EC 3.6.3.14)	FIG00023994	icw(5);F0F1-type_ATP_synthase
NODE_71_length_58179_cov_42.447174	fig|6666666.34159.peg.1385	peg	NODE_71_length_58179_cov_42.447174_13333_11819	13333	11819	-	ATP synthase alpha chain (EC 3.6.3.14)	FIG00000082	icw(6);F0F1-type_ATP_synthase
NODE_71_length_58179_cov_42.447174	fig|6666666.34159.peg.1386	peg	NODE_71_length_58179_cov_42.447174_13881_13330	13881	13330	-	ATP synthase delta chain (EC 3.6.3.14)	FIG00000262	icw(1);F0F1-type_ATP_synthase
NODE_71_length_58179_cov_42.447174	fig|6666666.34159.peg.1387	peg	NODE_71_length_58179_cov_42.447174_14363_13878	14363	13878	-	ATP synthase B chain (EC 3.6.3.14)	FIG00000186	icw(2);F0F1-type_ATP_synthase
NODE_71_length_58179_cov_42.447174	fig|6666666.34159.peg.1388	peg	NODE_71_length_58179_cov_42.447174_14601_14368	14601	14368	-	ATP synthase C chain (EC 3.6.3.14)	FIG00017607	isu;F0F1-type_ATP_synthase
NODE_71_length_58179_cov_42.447174	fig|6666666.34159.peg.1389	peg	NODE_71_length_58179_cov_42.447174_15374_14616	15374	14616	-	ATP synthase A chain (EC 3.6.3.14)	FIG00000138	icw(7);F0F1-type_ATP_synthase
NODE_71_length_58179_cov_42.447174	fig|6666666.34159.peg.1390	peg	NODE_71_length_58179_cov_42.447174_15752_15381	15752	15381	-	unknown protein	FIG00764722	ff
NODE_71_length_58179_cov_42.447174	fig|6666666.34159.peg.1391	peg	NODE_71_length_58179_cov_42.447174_15966_15742	15966	15742	-	ATP synthase protein I		icw(3);F0F1-type_ATP_synthase
NODE_71_length_58179_cov_42.447174	fig|6666666.34159.peg.1392	peg	NODE_71_length_58179_cov_42.447174_16285_16842	16285	16842	+	hypothetical protein		
NODE_71_length_58179_cov_42.447174	fig|6666666.34159.peg.1393	peg	NODE_71_length_58179_cov_42.447174_17040_17285	17040	17285	+	hypothetical protein		
NODE_71_length_58179_cov_42.447174	fig|6666666.34159.peg.1394	peg	NODE_71_length_58179_cov_42.447174_17287_17784	17287	17784	+	hypothetical protein		
NODE_71_length_58179_cov_42.447174	fig|6666666.34159.peg.1395	peg	NODE_71_length_58179_cov_42.447174_19770_17773	19770	17773	-	hypothetical protein	FIG00638284	ff

NODE_71_length_58179_cov_42.447174	fig|6666666.34159.peg.1396	peg	NODE_71_length_58179_cov_42.447174_20006_20380	20006	20380	+	hypothetical protein	FIG00638284	ff
NODE_71_length_58179_cov_42.447174	fig|6666666.34159.peg.1397	peg	NODE_71_length_58179_cov_42.447174_20396_21352	20396	21352	+	Transaldolase (EC 2.2.1.2)	FIG00000023	isu;Fructose_utilization isu;Pentose_phosphate_pathway
NODE_71_length_58179_cov_42.447174	fig|6666666.34159.peg.1398	peg	NODE_71_length_58179_cov_42.447174_24169_21422	24169	21422	-	Cell division protein FtsH (EC 3.4.24.-)	FIG00018369	icw(1);Cell_division-ribosomal_stress_proteins_cluster icw(1);Folate_biosynthesis_cluster
NODE_71_length_58179_cov_42.447174	fig|6666666.34159.peg.1399	peg	NODE_71_length_58179_cov_42.447174_25967_24687	25967	24687	-	tRNA(Ile)-lysidine synthetase	FIG00000257	isu;tRNA_processing isu;tRNA_modification_Bacteria isu;Cell_division
NODE_71_length_58179_cov_42.447174	fig|6666666.34159.peg.1400	peg	NODE_71_length_58179_cov_42.447174_26372_25977	26372	25977	-	hypothetical protein		
NODE_71_length_58179_cov_42.447174	fig|6666666.34159.peg.1401	peg	NODE_71_length_58179_cov_42.447174_26526_27665	26526	27665	+	hypothetical protein		
NODE_71_length_58179_cov_42.447174	fig|6666666.34159.peg.1402	peg	NODE_71_length_58179_cov_42.447174_27665_28756	27665	28756	+	FIG00899446: hypothetical protein	FIG00899445	ff
NODE_71_length_58179_cov_42.447174	fig|6666666.34159.peg.1403	peg	NODE_71_length_58179_cov_42.447174_28788_31298	28788	31298	+	UDP-N-acetylmuramoylalanyl-D-glutamyl-2,6-diaminopimelate--D-alanyl-D-alanine ligase (EC 6.3.2.10)	FIG00024401	idu(1);Methicillin_resistance_in_Staphylococci
NODE_71_length_58179_cov_42.447174	fig|6666666.34159.rna.21	rna	NODE_71_length_58179_cov_42.447174_31371_31453	31371	31453	+	tRNA-Leu-CAA		isu;tRNAs
NODE_71_length_58179_cov_42.447174	fig|6666666.34159.peg.1405	peg	NODE_71_length_58179_cov_42.447174_32173_31946	32173	31946	-	hypothetical protein		
NODE_71_length_58179_cov_42.447174	fig|6666666.34159.peg.1406	peg	NODE_71_length_58179_cov_42.447174_32556_32293	32556	32293	-	hypothetical protein		
NODE_71_length_58179_cov_42.447174	fig|6666666.34159.peg.1407	peg	NODE_71_length_58179_cov_42.447174_33776_32553	33776	32553	-	hypothetical protein		
NODE_71_length_58179_cov_42.447174	fig|6666666.34159.peg.1408	peg	NODE_71_length_58179_cov_42.447174_34350_34568	34350	34568	+	hypothetical protein		
NODE_71_length_58179_cov_42.447174	fig|6666666.34159.peg.1409	peg	NODE_71_length_58179_cov_42.447174_35034_34897	35034	34897	-	hypothetical protein		
NODE_71_length_58179_cov_42.447174	fig|6666666.34159.peg.1410	peg	NODE_71_length_58179_cov_42.447174_35144_35917	35144	35917	+	hypothetical protein		
NODE_71_length_58179_cov_42.447174	fig|6666666.34159.peg.1411	peg	NODE_71_length_58179_cov_42.447174_36043_36945	36043	36945	+	hypothetical protein		
NODE_71_length_58179_cov_42.447174	fig|6666666.34159.peg.1412	peg	NODE_71_length_58179_cov_42.447174_36987_37445	36987	37445	+	hypothetical protein		
NODE_71_length_58179_cov_42.447174	fig|6666666.34159.peg.1413	peg	NODE_71_length_58179_cov_42.447174_37762_40158	37762	40158	+	hypothetical protein		
NODE_71_length_58179_cov_42.447174	fig|6666666.34159.peg.1414	peg	NODE_71_length_58179_cov_42.447174_41073_42038	41073	42038	+	hypothetical protein		
NODE_71_length_58179_cov_42.447174	fig|6666666.34159.peg.1415	peg	NODE_71_length_58179_cov_42.447174_42255_42464	42255	42464	+	Alkylphosphonate utilization operon protein PhnA	FIG00000968	isu;Alkylphosphonate_utilization
NODE_71_length_58179_cov_42.447174	fig|6666666.34159.peg.1416	peg	NODE_71_length_58179_cov_42.447174_42569_43516	42569	43516	+	hypothetical protein	FIG00638284	ff
NODE_71_length_58179_cov_42.447174	fig|6666666.34159.peg.1417	peg	NODE_71_length_58179_cov_42.447174_44370_43519	44370	43519	-	3-oxoacyl-[acyl-carrier protein] reductase (EC 1.1.1.100)	FIG00621114	idu(12);CBSS-246196.1.peg.364 idu(12);Fatty_Acid_Biosynthesis_FASII
NODE_71_length_58179_cov_42.447174	fig|6666666.34159.peg.1418	peg	NODE_71_length_58179_cov_42.447174_44552_45907	44552	45907	+	Protein of unknown function DUF1597		
NODE_71_length_58179_cov_42.447174	fig|6666666.34159.peg.1419	peg	NODE_71_length_58179_cov_42.447174_46367_47380	46367	47380	+	hypothetical protein		
NODE_71_length_58179_cov_42.447174	fig|6666666.34159.peg.1420	peg	NODE_71_length_58179_cov_42.447174_47365_47484	47365	47484	+	hypothetical protein		
NODE_71_length_58179_cov_42.447174	fig|6666666.34159.peg.1421	peg	NODE_71_length_58179_cov_42.447174_47459_48448	47459	48448	+	Quinone oxidoreductase (EC 1.6.5.5)	FIG00002982	idu(1);Quinone_oxidoreductase_family
NODE_71_length_58179_cov_42.447174	fig|6666666.34159.peg.1422	peg	NODE_71_length_58179_cov_42.447174_48571_49011	48571	49011	+	transposase, IS605 OrfB family		
NODE_71_length_58179_cov_42.447174	fig|6666666.34159.peg.1423	peg	NODE_71_length_58179_cov_42.447174_49494_50072	49494	50072	+	transposase, IS605 OrfB family		
NODE_71_length_58179_cov_42.447174	fig|6666666.34159.peg.1424	peg	NODE_71_length_58179_cov_42.447174_51220_50186	51220	50186	-	Enoyl-[acyl-carrier-protein] reductase [FMN] (EC 1.3.1.9)	FIG00007641	icw(1);Fatty_Acid_Biosynthesis_FASII
NODE_71_length_58179_cov_42.447174	fig|6666666.34159.peg.1425	peg	NODE_71_length_58179_cov_42.447174_52133_51213	52133	51213	-	COG0451: Nucleoside-diphosphate-sugar epimerases		
NODE_71_length_58179_cov_42.447174	fig|6666666.34159.peg.1426	peg	NODE_71_length_58179_cov_42.447174_52219_52914	52219	52914	+	Pirin	FIG01319925	ff
NODE_71_length_58179_cov_42.447174	fig|6666666.34159.peg.1427	peg	NODE_71_length_58179_cov_42.447174_53025_54029	53025	54029	+	Cupin 2, conserved barrel domain protein		
NODE_71_length_58179_cov_42.447174	fig|6666666.34159.peg.1428	peg	NODE_71_length_58179_cov_42.447174_54201_54956	54201	54956	+	3-oxoacyl-[acyl-carrier protein] reductase (EC 1.1.1.100)	FIG00621114	idu(12);CBSS-246196.1.peg.364 idu(12);Fatty_Acid_Biosynthesis_FASII
NODE_71_length_58179_cov_42.447174	fig|6666666.34159.peg.1429	peg	NODE_71_length_58179_cov_42.447174_55071_55301	55071	55301	+	hypothetical protein		
NODE_71_length_58179_cov_42.447174	fig|6666666.34159.peg.1430	peg	NODE_71_length_58179_cov_42.447174_55295_55414	55295	55414	+	hypothetical protein		
NODE_71_length_58179_cov_42.447174	fig|6666666.34159.peg.1431	peg	NODE_71_length_58179_cov_42.447174_55662_55387	55662	55387	-	hypothetical protein		
NODE_71_length_58179_cov_42.447174	fig|6666666.34159.peg.1432	peg	NODE_71_length_58179_cov_42.447174_56919_55726	56919	55726	-	conserved hypothetical protein		
NODE_71_length_58179_cov_42.447174	fig|6666666.34159.peg.1433	peg	NODE_71_length_58179_cov_42.447174_57747_57013	57747	57013	-	Replicative DNA helicase (EC 3.6.1.-)	FIG00061313	ff
NODE_71_length_58179_cov_42.447174	fig|6666666.34159.peg.1434	peg	NODE_71_length_58179_cov_42.447174_57877_57722	57877	57722	-	hypothetical protein		
NODE_75_length_93224_cov_42.572952	fig|6666666.34159.peg.1435	peg	NODE_75_length_93224_cov_42.572952_385_257	385	257	-	hypothetical protein		
NODE_75_length_93224_cov_42.572952	fig|6666666.34159.peg.1436	peg	NODE_75_length_93224_cov_42.572952_2264_741	2264	741	-	hypothetical protein		
NODE_75_length_93224_cov_42.572952	fig|6666666.34159.peg.1437	peg	NODE_75_length_93224_cov_42.572952_2885_2721	2885	2721	-	hypothetical protein		
NODE_75_length_93224_cov_42.572952	fig|6666666.34159.peg.1438	peg	NODE_75_length_93224_cov_42.572952_2874_3737	2874	3737	+	hypothetical protein	FIG00638284	ff
NODE_75_length_93224_cov_42.572952	fig|6666666.34159.peg.1439	peg	NODE_75_length_93224_cov_42.572952_5197_3725	5197	3725	-	hypothetical protein		
NODE_75_length_93224_cov_42.572952	fig|6666666.34159.peg.1440	peg	NODE_75_length_93224_cov_42.572952_6124_5279	6124	5279	-	LysR-family regulatory protein		
NODE_75_length_93224_cov_42.572952	fig|6666666.34159.peg.1441	peg	NODE_75_length_93224_cov_42.572952_6238_6678	6238	6678	+	hypothetical protein		
NODE_75_length_93224_cov_42.572952	fig|6666666.34159.peg.1442	peg	NODE_75_length_93224_cov_42.572952_6725_7042	6725	7042	+	hypothetical protein		
NODE_75_length_93224_cov_42.572952	fig|6666666.34159.peg.1443	peg	NODE_75_length_93224_cov_42.572952_7044_8657	7044	8657	+	hypothetical protein	FIG00638284	ff
NODE_75_length_93224_cov_42.572952	fig|6666666.34159.peg.1444	peg	NODE_75_length_93224_cov_42.572952_9387_8665	9387	8665	-	ABC transporter related		
NODE_75_length_93224_cov_42.572952	fig|6666666.34159.peg.1445	peg	NODE_75_length_93224_cov_42.572952_9827_9384	9827	9384	-	amino acid ABC transporter, permease protein		
NODE_75_length_93224_cov_42.572952	fig|6666666.34159.peg.1446	peg	NODE_75_length_93224_cov_42.572952_10840_10040	10840	10040	-	amino acid ABC transporter, periplasmic amino acid-binding protein		
NODE_75_length_93224_cov_42.572952	fig|6666666.34159.peg.1447	peg	NODE_75_length_93224_cov_42.572952_12529_11579	12529	11579	-	hypothetical protein	FIG00638284	ff
NODE_75_length_93224_cov_42.572952	fig|6666666.34159.peg.1448	peg	NODE_75_length_93224_cov_42.572952_13424_12552	13424	12552	-	hypothetical protein		
NODE_75_length_93224_cov_42.572952	fig|6666666.34159.peg.1449	peg	NODE_75_length_93224_cov_42.572952_13541_14401	13541	14401	+	LysR family transcriptional regulator		
NODE_75_length_93224_cov_42.572952	fig|6666666.34159.peg.1450	peg	NODE_75_length_93224_cov_42.572952_16996_14396	16996	14396	-	hypothetical protein	FIG00638284	ff
NODE_75_length_93224_cov_42.572952	fig|6666666.34159.peg.1451	peg	NODE_75_length_93224_cov_42.572952_17244_17125	17244	17125	-	hypothetical protein		
NODE_75_length_93224_cov_42.572952	fig|6666666.34159.peg.1452	peg	NODE_75_length_93224_cov_42.572952_17243_17386	17243	17386	+	hypothetical protein		
NODE_75_length_93224_cov_42.572952	fig|6666666.34159.peg.1453	peg	NODE_75_length_93224_cov_42.572952_17683_17387	17683	17387	-	hypothetical protein		
NODE_75_length_93224_cov_42.572952	fig|6666666.34159.peg.1454	peg	NODE_75_length_93224_cov_42.572952_17657_17779	17657	17779	+	hypothetical protein		

NODE_75_length_93224_cov_42.572952	fig|6666666.34159.peg.1455	peg	NODE_75_length_93224_cov_42.572952_17853_18059	17853	18059	+	hypothetical protein		
NODE_75_length_93224_cov_42.572952	fig|6666666.34159.peg.1456	peg	NODE_75_length_93224_cov_42.572952_18092_18646	18092	18646	+	hypothetical protein		
NODE_75_length_93224_cov_42.572952	fig|6666666.34159.peg.1457	peg	NODE_75_length_93224_cov_42.572952_18659_19297	18659	19297	+	hypothetical protein		
NODE_75_length_93224_cov_42.572952	fig|6666666.34159.peg.1458	peg	NODE_75_length_93224_cov_42.572952_19362_19475	19362	19475	+	hypothetical protein		
NODE_75_length_93224_cov_42.572952	fig|6666666.34159.peg.1459	peg	NODE_75_length_93224_cov_42.572952_19746_19982	19746	19982	+	hypothetical protein		
NODE_75_length_93224_cov_42.572952	fig|6666666.34159.peg.1460	peg	NODE_75_length_93224_cov_42.572952_20062_20211	20062	20211	+	hypothetical protein		
NODE_75_length_93224_cov_42.572952	fig|6666666.34159.peg.1461	peg	NODE_75_length_93224_cov_42.572952_21431_20208	21431	20208	-	putative glutathione-regulated potassium-efflux system protein		
NODE_75_length_93224_cov_42.572952	fig|6666666.34159.peg.1462	peg	NODE_75_length_93224_cov_42.572952_21847_23208	21847	23208	+	sodium/alanine symporter family protein	FIG01316725	ff
NODE_75_length_93224_cov_42.572952	fig|6666666.34159.peg.1463	peg	NODE_75_length_93224_cov_42.572952_24008_23205	24008	23205	-	Inositol-1-monophosphatase (EC 3.1.3.25)	FIG00002332	idu(1);Di-Inositol-Phosphate_biosynthesis
NODE_75_length_93224_cov_42.572952	fig|6666666.34159.peg.1464	peg	NODE_75_length_93224_cov_42.572952_24163_26034	24163	26034	+	Acyl-CoA dehydrogenase, short-chain specific (EC 1.3.99.2)	FIG00009849	isu;Isoleucine_degradation
NODE_75_length_93224_cov_42.572952	fig|6666666.34159.peg.1465	peg	NODE_75_length_93224_cov_42.572952_26509_26072	26509	26072	-	hypothetical protein		
NODE_75_length_93224_cov_42.572952	fig|6666666.34159.peg.1466	peg	NODE_75_length_93224_cov_42.572952_26694_28241	26694	28241	+	Glycyl-tRNA synthetase (EC 6.1.1.14)	FIG00001189	isu;tRNA_aminoacylation,_Gly
NODE_75_length_93224_cov_42.572952	fig|6666666.34159.peg.1467	peg	NODE_75_length_93224_cov_42.572952_29158_28280	29158	28280	-	hypothetical protein		
NODE_75_length_93224_cov_42.572952	fig|6666666.34159.peg.1468	peg	NODE_75_length_93224_cov_42.572952_30246_29185	30246	29185	-	Magnesium and cobalt transport protein CorA	FIG00004180	isu;Magnesium_transport isu;Campylobacter_Iron_Metabolism
NODE_75_length_93224_cov_42.572952	fig|6666666.34159.peg.1469	peg	NODE_75_length_93224_cov_42.572952_30284_30397	30284	30397	+	hypothetical protein		
NODE_75_length_93224_cov_42.572952	fig|6666666.34159.peg.1470	peg	NODE_75_length_93224_cov_42.572952_30381_31055	30381	31055	+	Phosphate transport regulator (distant homolog of PhoU)	FIG00022740	ff
NODE_75_length_93224_cov_42.572952	fig|6666666.34159.peg.1471	peg	NODE_75_length_93224_cov_42.572952_31055_32467	31055	32467	+	Probable low-affinity inorganic phosphate transporter	FIG00009601	ff
NODE_75_length_93224_cov_42.572952	fig|6666666.34159.peg.1472	peg	NODE_75_length_93224_cov_42.572952_32460_33032	32460	33032	+	hypothetical protein		
NODE_75_length_93224_cov_42.572952	fig|6666666.34159.peg.1473	peg	NODE_75_length_93224_cov_42.572952_33249_33458	33249	33458	+	hypothetical protein		
NODE_75_length_93224_cov_42.572952	fig|6666666.34159.peg.1474	peg	NODE_75_length_93224_cov_42.572952_33500_33637	33500	33637	+	hypothetical protein		
NODE_75_length_93224_cov_42.572952	fig|6666666.34159.peg.1475	peg	NODE_75_length_93224_cov_42.572952_33746_33859	33746	33859	+	hypothetical protein		
NODE_75_length_93224_cov_42.572952	fig|6666666.34159.peg.1476	peg	NODE_75_length_93224_cov_42.572952_33926_34627	33926	34627	+	hypothetical protein		
NODE_75_length_93224_cov_42.572952	fig|6666666.34159.peg.1477	peg	NODE_75_length_93224_cov_42.572952_35017_36246	35017	36246	+	hypothetical protein		
NODE_75_length_93224_cov_42.572952	fig|6666666.34159.peg.1478	peg	NODE_75_length_93224_cov_42.572952_36547_36918	36547	36918	+	SSU ribosomal protein S12p (S23e)	FIG00000203	isu;Ribosomal_protein_S12p_Asp_methylthiotransferase icw(2);Mycobacterium_virulence_operon_involve
NODE_75_length_93224_cov_42.572952	fig|6666666.34159.peg.1479	peg	NODE_75_length_93224_cov_42.572952_37088_37561	37088	37561	+	SSU ribosomal protein S7p (S5e)	FIG00000209	isu;Mycobacterium_virulence_operon_involved_in_protein_synthesis_(SSU_ribosomal_proteins)
NODE_75_length_93224_cov_42.572952	fig|6666666.34159.peg.1480	peg	NODE_75_length_93224_cov_42.572952_37576_39663	37576	39663	+	Translation elongation factor G	FIG00063189	isu;Tetracycline_resistance,_ribosome_protection_type,_too isu;Translation_elongation_factor_G_family
NODE_75_length_93224_cov_42.572952	fig|6666666.34159.peg.1481	peg	NODE_75_length_93224_cov_42.572952_39676_40011	39676	40011	+	SSU ribosomal protein S10p (S20e)	FIG00001107	ff
NODE_75_length_93224_cov_42.572952	fig|6666666.34159.peg.1482	peg	NODE_75_length_93224_cov_42.572952_41128_40055	41128	40055	-	hypothetical protein		
NODE_75_length_93224_cov_42.572952	fig|6666666.34159.peg.1483	peg	NODE_75_length_93224_cov_42.572952_41632_41195	41632	41195	-	hypothetical protein	FIG00638284	ff
NODE_75_length_93224_cov_42.572952	fig|6666666.34159.peg.1484	peg	NODE_75_length_93224_cov_42.572952_42774_41632	42774	41632	-	Cell division protein FtsW	FIG00000025	ff
NODE_75_length_93224_cov_42.572952	fig|6666666.34159.peg.1485	peg	NODE_75_length_93224_cov_42.572952_42867_43598	42867	43598	+	Biotin-protein ligase (EC 6.3.4.15) / Biotin operon repressor	FIG00000692	isu;Biotin_synthesis_cluster isu;Biotin_synthesis_cluster isu;Biotin_biosynthesis isu;Biotin_biosynthesis
NODE_75_length_93224_cov_42.572952	fig|6666666.34159.peg.1486	peg	NODE_75_length_93224_cov_42.572952_46386_43603	46386	43603	-	Pyruvate,phosphate dikinase (EC 2.7.9.1)	FIG00001076	isu;Glycolysis_and_Gluconeogenesis isu;Pyruvate_metabolism_I:_anaplerotic_reactions,_PEP
NODE_75_length_93224_cov_42.572952	fig|6666666.34159.peg.1487	peg	NODE_75_length_93224_cov_42.572952_46982_46395	46982	46395	-	hypothetical protein		
NODE_75_length_93224_cov_42.572952	fig|6666666.34159.peg.1488	peg	NODE_75_length_93224_cov_42.572952_47889_47014	47889	47014	-	hypothetical protein		
NODE_75_length_93224_cov_42.572952	fig|6666666.34159.peg.1489	peg	NODE_75_length_93224_cov_42.572952_48000_48125	48000	48125	+	hypothetical protein		
NODE_75_length_93224_cov_42.572952	fig|6666666.34159.peg.1490	peg	NODE_75_length_93224_cov_42.572952_48109_49440	48109	49440	+	conserved hypothetical protein, putative ATPase		
NODE_75_length_93224_cov_42.572952	fig|6666666.34159.peg.1491	peg	NODE_75_length_93224_cov_42.572952_49708_49412	49708	49412	-	hypothetical protein		
NODE_75_length_93224_cov_42.572952	fig|6666666.34159.peg.1492	peg	NODE_75_length_93224_cov_42.572952_49815_49943	49815	49943	+	hypothetical protein		
NODE_75_length_93224_cov_42.572952	fig|6666666.34159.peg.1493	peg	NODE_75_length_93224_cov_42.572952_49959_50330	49959	50330	+	hypothetical protein		
NODE_75_length_93224_cov_42.572952	fig|6666666.34159.peg.1494	peg	NODE_75_length_93224_cov_42.572952_51386_50346	51386	50346	-	hypothetical protein		
NODE_75_length_93224_cov_42.572952	fig|6666666.34159.peg.1495	peg	NODE_75_length_93224_cov_42.572952_51618_52652	51618	52652	+	Protease	FIG00493420	ff
NODE_75_length_93224_cov_42.572952	fig|6666666.34159.peg.1496	peg	NODE_75_length_93224_cov_42.572952_52721_55393	52721	55393	+	DNA polymerase I (EC 2.7.7.7)	FIG00000404	isu;DNA_Repair_Base_Excision
NODE_75_length_93224_cov_42.572952	fig|6666666.34159.peg.1497	peg	NODE_75_length_93224_cov_42.572952_55387_55989	55387	55989	+	Dephospho-CoA kinase (EC 2.7.1.24)	FIG00000267	isu;Coenzyme_A_Biosynthesis
NODE_75_length_93224_cov_42.572952	fig|6666666.34159.peg.1498	peg	NODE_75_length_93224_cov_42.572952_56017_57396	56017	57396	+	Transcription termination factor Rho	FIG00000392	isu;Transcription_factors_bacterial
NODE_75_length_93224_cov_42.572952	fig|6666666.34159.peg.1499	peg	NODE_75_length_93224_cov_42.572952_57528_58370	57528	58370	+	FIG00899468: hypothetical protein	FIG00899467	ff
NODE_75_length_93224_cov_42.572952	fig|6666666.34159.peg.1500	peg	NODE_75_length_93224_cov_42.572952_60966_58405	60966	58405	-	hypothetical protein	FIG00638284	ff
NODE_75_length_93224_cov_42.572952	fig|6666666.34159.peg.1501	peg	NODE_75_length_93224_cov_42.572952_61508_61059	61508	61059	-	thioredoxin		
NODE_75_length_93224_cov_42.572952	fig|6666666.34159.peg.1502	peg	NODE_75_length_93224_cov_42.572952_64154_61644	64154	61644	-	Protein tyrosine phosphatase, non-receptor type 13 (EC 3.1.3.48)		
NODE_75_length_93224_cov_42.572952	fig|6666666.34159.peg.1503	peg	NODE_75_length_93224_cov_42.572952_64762_64364	64762	64364	-	Thioredoxin	FIG01318274	idu(1);CBSS-315749.4.peg.3658 idu(1);Glycine_reductase,_sarcosine_reductase_and_betaine_reductase
NODE_75_length_93224_cov_42.572952	fig|6666666.34159.peg.1504	peg	NODE_75_length_93224_cov_42.572952_65941_64931	65941	64931	-	COG0536: GTP-binding protein Obg	FIG00000169	ff
NODE_75_length_93224_cov_42.572952	fig|6666666.34159.peg.1505	peg	NODE_75_length_93224_cov_42.572952_66333_66082	66333	66082	-	LSU ribosomal protein L27p	FIG00000189	icw(1);CBSS-176279.3.peg.868
NODE_75_length_93224_cov_42.572952	fig|6666666.34159.peg.1506	peg	NODE_75_length_93224_cov_42.572952_66659_66345	66659	66345	-	LSU ribosomal protein L21p	FIG00000276	isu;CBSS-176279.3.peg.868
NODE_75_length_93224_cov_42.572952	fig|6666666.34159.peg.1507	peg	NODE_75_length_93224_cov_42.572952_66877_67860	66877	67860	+	Phosphoglycerate dehydrogenase and related dehydrogenase		
NODE_75_length_93224_cov_42.572952	fig|6666666.34159.peg.1508	peg	NODE_75_length_93224_cov_42.572952_68154_67990	68154	67990	-	hypothetical protein		
NODE_75_length_93224_cov_42.572952	fig|6666666.34159.peg.1509	peg	NODE_75_length_93224_cov_42.572952_68380_68261	68380	68261	-	hypothetical protein		
NODE_75_length_93224_cov_42.572952	fig|6666666.34159.peg.1510	peg	NODE_75_length_93224_cov_42.572952_68581_68700	68581	68700	+	hypothetical protein		
NODE_75_length_93224_cov_42.572952	fig|6666666.34159.peg.1511	peg	NODE_75_length_93224_cov_42.572952_68851_69450	68851	69450	+	hypothetical protein		
NODE_75_length_93224_cov_42.572952	fig|6666666.34159.rna.22	rna	NODE_75_length_93224_cov_42.572952_69585_69513	69585	69513	-	tRNA-Phe-GAA		isu;tRNAs
NODE_75_length_93224_cov_42.572952	fig|6666666.34159.peg.1512	peg	NODE_75_length_93224_cov_42.572952_71745_69706	71745	69706	-	Peptidase, S41 family	FIG01369989	ff

NODE_75_length_93224_cov_42.572952	fig|6666666.34159.peg.1513	peg	NODE_75_length_93224_cov_42.572952_71951_72679	71951	72679	+	Segregation and condensation protein A	FIG00019652	ff
NODE_75_length_93224_cov_42.572952	fig|6666666.34159.peg.1514	peg	NODE_75_length_93224_cov_42.572952_72683_73330	72683	73330	+	Segregation and condensation protein B	FIG00133569	isu;CBSS-203122.12.peg.188
NODE_75_length_93224_cov_42.572952	fig|6666666.34159.peg.1515	peg	NODE_75_length_93224_cov_42.572952_73334_74377	73334	74377	+	Oligopeptide transport ATP-binding protein OppD (TC 3.A.1.5.1)	FIG00002049	icw(1);ABC_transporter_oligopeptide_(TC_3.A.1.5.1)
NODE_75_length_93224_cov_42.572952	fig|6666666.34159.peg.1516	peg	NODE_75_length_93224_cov_42.572952_74374_75357	74374	75357	+	Oligopeptide transport ATP-binding protein OppF (TC 3.A.1.5.1)	FIG00018865	isu;ABC_transporter_oligopeptide_(TC_3.A.1.5.1)
NODE_75_length_93224_cov_42.572952	fig|6666666.34159.peg.1517	peg	NODE_75_length_93224_cov_42.572952_75440_76690	75440	76690	+	hypothetical protein	FIG00638284	ff
NODE_75_length_93224_cov_42.572952	fig|6666666.34159.peg.1518	peg	NODE_75_length_93224_cov_42.572952_78519_76687	78519	76687	-	hypothetical protein		
NODE_75_length_93224_cov_42.572952	fig|6666666.34159.peg.1519	peg	NODE_75_length_93224_cov_42.572952_79759_78599	79759	78599	-	hypothetical protein	FIG00638284	ff
NODE_75_length_93224_cov_42.572952	fig|6666666.34159.peg.1520	peg	NODE_75_length_93224_cov_42.572952_80482_79778	80482	79778	-	Ribosomal small subunit pseudouridine synthase A (EC 4.2.1.70)	FIG00002716	isu;RNA_pseudouridine_syntheses
NODE_75_length_93224_cov_42.572952	fig|6666666.34159.peg.1521	peg	NODE_75_length_93224_cov_42.572952_80646_80951	80646	80951	+	hypothetical protein		
NODE_75_length_93224_cov_42.572952	fig|6666666.34159.peg.1522	peg	NODE_75_length_93224_cov_42.572952_81443_81090	81443	81090	-	hypothetical protein		
NODE_75_length_93224_cov_42.572952	fig|6666666.34159.peg.1523	peg	NODE_75_length_93224_cov_42.572952_81720_81406	81720	81406	-	hypothetical protein		
NODE_75_length_93224_cov_42.572952	fig|6666666.34159.peg.1524	peg	NODE_75_length_93224_cov_42.572952_81851_82528	81851	82528	+	Phosphoglycerate mutase (EC 5.4.2.1)	FIG00005840	isu;Phosphoglycerate_mutase_protein_family isu;Glycolysis_and_Gluconeogenesis
NODE_75_length_93224_cov_42.572952	fig|6666666.34159.peg.1525	peg	NODE_75_length_93224_cov_42.572952_82529_83692	82529	83692	+	Cysteine desulfurase (EC 2.8.1.7)	FIG00000001	idu(1);Iron-sulfur_cluster_assembly idu(1);CBSS-84588.1.peg.1247 idu(1);CBSS-393130.3.peg.794 idu(1);tRNA_modification_Bacteria
NODE_75_length_93224_cov_42.572952	fig|6666666.34159.peg.1526	peg	NODE_75_length_93224_cov_42.572952_83703_84488	83703	84488	+	nifU protein, putative	FIG01955269	ff
NODE_75_length_93224_cov_42.572952	fig|6666666.34159.peg.1527	peg	NODE_75_length_93224_cov_42.572952_84533_85768	84533	85768	+	COG2110, Macro domain, possibly ADP-ribose binding module	FIG00004221	ff
NODE_75_length_93224_cov_42.572952	fig|6666666.34159.peg.1528	peg	NODE_75_length_93224_cov_42.572952_85811_85942	85811	85942	+	hypothetical protein		
NODE_75_length_93224_cov_42.572952	fig|6666666.34159.peg.1529	peg	NODE_75_length_93224_cov_42.572952_87528_88274	87528	88274	+	hypothetical protein		
NODE_75_length_93224_cov_42.572952	fig|6666666.34159.peg.1530	peg	NODE_75_length_93224_cov_42.572952_88664_90745	88664	90745	+	Pyrophosphate-energized proton pump (EC 3.6.1.1)	FIG00031504	ff
NODE_75_length_93224_cov_42.572952	fig|6666666.34159.peg.1531	peg	NODE_75_length_93224_cov_42.572952_92709_90784	92709	90784	-	hypothetical protein	FIG00638284	ff
NODE_75_length_93224_cov_42.572952	fig|6666666.34159.peg.1532	peg	NODE_75_length_93224_cov_42.572952_92940_93104	92940	93104	+	hypothetical protein		
NODE_79_length_22744_cov_41.467903	fig|6666666.34159.peg.1533	peg	NODE_79_length_22744_cov_41.467903_488_682	488	682	+	hypothetical protein		
NODE_79_length_22744_cov_41.467903	fig|6666666.34159.peg.1534	peg	NODE_79_length_22744_cov_41.467903_792_679	792	679	-	hypothetical protein		
NODE_79_length_22744_cov_41.467903	fig|6666666.34159.peg.1535	peg	NODE_79_length_22744_cov_41.467903_1456_881	1456	881	-	hypothetical protein		
NODE_79_length_22744_cov_41.467903	fig|6666666.34159.peg.1536	peg	NODE_79_length_22744_cov_41.467903_2396_1524	2396	1524	-	Zinc ABC transporter, inner membrane permease protein ZnuB	FIG00005113	ff
NODE_79_length_22744_cov_41.467903	fig|6666666.34159.peg.1537	peg	NODE_79_length_22744_cov_41.467903_3119_2400	3119	2400	-	Zinc ABC transporter, ATP-binding protein ZnuC	FIG00059280	ff
NODE_79_length_22744_cov_41.467903	fig|6666666.34159.peg.1538	peg	NODE_79_length_22744_cov_41.467903_4018_3158	4018	3158	-	Zinc ABC transporter, periplasmic-binding protein ZnuA	FIG00021555	ff
NODE_79_length_22744_cov_41.467903	fig|6666666.34159.peg.1539	peg	NODE_79_length_22744_cov_41.467903_4262_6118	4262	6118	+	Chaperone protein HtpG	FIG00000594	isu;Protein_chaperones
NODE_79_length_22744_cov_41.467903	fig|6666666.34159.peg.1540	peg	NODE_79_length_22744_cov_41.467903_6160_7359	6160	7359	+	hypothetical protein	FIG00638284	ff
NODE_79_length_22744_cov_41.467903	fig|6666666.34159.peg.1541	peg	NODE_79_length_22744_cov_41.467903_7572_7444	7572	7444	-	hypothetical protein		
NODE_79_length_22744_cov_41.467903	fig|6666666.34159.peg.1542	peg	NODE_79_length_22744_cov_41.467903_7752_7639	7752	7639	-	hypothetical protein		
NODE_79_length_22744_cov_41.467903	fig|6666666.34159.peg.1543	peg	NODE_79_length_22744_cov_41.467903_8347_10278	8347	10278	+	hypothetical protein	FIG00638284	ff
NODE_79_length_22744_cov_41.467903	fig|6666666.34159.peg.1544	peg	NODE_79_length_22744_cov_41.467903_10275_10640	10275	10640	+	hypothetical protein		
NODE_79_length_22744_cov_41.467903	fig|6666666.34159.peg.1545	peg	NODE_79_length_22744_cov_41.467903_10650_11120	10650	11120	+	hypothetical protein		
NODE_79_length_22744_cov_41.467903	fig|6666666.34159.peg.1546	peg	NODE_79_length_22744_cov_41.467903_11136_11660	11136	11660	+	hypothetical protein		
NODE_79_length_22744_cov_41.467903	fig|6666666.34159.peg.1547	peg	NODE_79_length_22744_cov_41.467903_11667_13904	11667	13904	+	hypothetical protein	FIG00638284	ff
NODE_79_length_22744_cov_41.467903	fig|6666666.34159.peg.1548	peg	NODE_79_length_22744_cov_41.467903_13914_16151	13914	16151	+	hypothetical protein	FIG00638284	ff
NODE_79_length_22744_cov_41.467903	fig|6666666.34159.peg.1549	peg	NODE_79_length_22744_cov_41.467903_16154_16990	16154	16990	+	oxidoreductase, short chain dehydrogenase/reductase family		
NODE_79_length_22744_cov_41.467903	fig|6666666.34159.peg.1550	peg	NODE_79_length_22744_cov_41.467903_19273_17036	19273	17036	-	Nitric-oxide reductase (EC 1.7.99.7), quinol-dependent	FIG00033697	isu;Nitrosative_stress isu;Flavohaemoglobin isu;Denitrification
NODE_79_length_22744_cov_41.467903	fig|6666666.34159.peg.1551	peg	NODE_79_length_22744_cov_41.467903_19783_19899	19783	19899	+	hypothetical protein		
NODE_79_length_22744_cov_41.467903	fig|6666666.34159.peg.1552	peg	NODE_79_length_22744_cov_41.467903_20939_20280	20939	20280	-	hypothetical protein	FIG00638284	ff
NODE_79_length_22744_cov_41.467903	fig|6666666.34159.peg.1553	peg	NODE_79_length_22744_cov_41.467903_21809_21159	21809	21159	-	hypothetical protein	FIG00638284	ff
NODE_79_length_22744_cov_41.467903	fig|6666666.34159.peg.1554	peg	NODE_79_length_22744_cov_41.467903_21912_21793	21912	21793	-	hypothetical protein		
NODE_79_length_22744_cov_41.467903	fig|6666666.34159.peg.1555	peg	NODE_79_length_22744_cov_41.467903_21911_22165	21911	22165	+	hypothetical protein		
NODE_7_length_126357_cov_42.436516	fig|6666666.34159.peg.1556	peg	NODE_7_length_126357_cov_42.436516_524_7762	524	7762	+	hypothetical protein	FIG00638284	ff
NODE_7_length_126357_cov_42.436516	fig|6666666.34159.peg.1557	peg	NODE_7_length_126357_cov_42.436516_7972_8103	7972	8103	+	hypothetical protein		
NODE_7_length_126357_cov_42.436516	fig|6666666.34159.peg.1558	peg	NODE_7_length_126357_cov_42.436516_8344_9108	8344	9108	+	hypothetical protein	FIG00638284	ff
NODE_7_length_126357_cov_42.436516	fig|6666666.34159.peg.1559	peg	NODE_7_length_126357_cov_42.436516_9408_9271	9408	9271	-	hypothetical protein		
NODE_7_length_126357_cov_42.436516	fig|6666666.34159.peg.1560	peg	NODE_7_length_126357_cov_42.436516_10632_9724	10632	9724	-	hypothetical protein		
NODE_7_length_126357_cov_42.436516	fig|6666666.34159.peg.1561	peg	NODE_7_length_126357_cov_42.436516_11518_10697	11518	10697	-	hypothetical protein		
NODE_7_length_126357_cov_42.436516	fig|6666666.34159.peg.1562	peg	NODE_7_length_126357_cov_42.436516_11670_13172	11670	13172	+	hypothetical protein	FIG00638284	ff
NODE_7_length_126357_cov_42.436516	fig|6666666.34159.peg.1563	peg	NODE_7_length_126357_cov_42.436516_13214_14149	13214	14149	+	hypothetical protein	FIG00638284	ff
NODE_7_length_126357_cov_42.436516	fig|6666666.34159.peg.1564	peg	NODE_7_length_126357_cov_42.436516_14156_15415	14156	15415	+	FIG065221: Holliday junction DNA helicase	FIG00150395	ff
NODE_7_length_126357_cov_42.436516	fig|6666666.34159.peg.1565	peg	NODE_7_length_126357_cov_42.436516_16398_15412	16398	15412	-	hypothetical protein	FIG00638284	ff
NODE_7_length_126357_cov_42.436516	fig|6666666.34159.peg.1566	peg	NODE_7_length_126357_cov_42.436516_17949_16501	17949	16501	-	hypothetical protein	FIG00638284	ff
NODE_7_length_126357_cov_42.436516	fig|6666666.34159.peg.1567	peg	NODE_7_length_126357_cov_42.436516_20014_18185	20014	18185	-	hypothetical protein	FIG00638284	ff
NODE_7_length_126357_cov_42.436516	fig|6666666.34159.peg.1568	peg	NODE_7_length_126357_cov_42.436516_20974_20246	20974	20246	-	hypothetical protein	FIG00638284	ff
NODE_7_length_126357_cov_42.436516	fig|6666666.34159.peg.1569	peg	NODE_7_length_126357_cov_42.436516_22427_20985	22427	20985	-	hypothetical protein		
NODE_7_length_126357_cov_42.436516	fig|6666666.34159.peg.1570	peg	NODE_7_length_126357_cov_42.436516_23140_22427	23140	22427	-	hypothetical protein		
NODE_7_length_126357_cov_42.436516	fig|6666666.34159.peg.1571	peg	NODE_7_length_126357_cov_42.436516_23975_23133	23975	23133	-	2-Keto-3-deoxy-D-manno-octulosonate-8-phosphate synthase (EC 2.5.1.55)	FIG00000556	isu;KDO2-Lipid_A_biosynthesis

NODE_7_length_126357_cov_42.436516	fig|6666666.34159.peg.1572	peg	NODE_7_length_126357_cov_42.436516_24511_23987	24511	23987	-	2-amino-4-hydroxy-6-hydroxymethyldihydropteridine pyrophosphokinase (EC 2.7.6.3)	FIG00000171	isu;Folate_Biosynthesis isu;Folate_biosynthesis_cluster
NODE_7_length_126357_cov_42.436516	fig|6666666.34159.peg.1573	peg	NODE_7_length_126357_cov_42.436516_24721_25005	24721	25005	+	hypothetical protein		
NODE_7_length_126357_cov_42.436516	fig|6666666.34159.peg.1574	peg	NODE_7_length_126357_cov_42.436516_27548_25164	27548	25164	-	ATP-dependent DNA ligase (EC 6.5.1.1) clustered with Ku protein, LigD	FIG00002268	icw(1);DNA_Repair_Base_Excision icw(1);DNA_ligases icw(1);Nonhomologous_End_Joining_in_Bacteria
NODE_7_length_126357_cov_42.436516	fig|6666666.34159.peg.1575	peg	NODE_7_length_126357_cov_42.436516_28364_27552	28364	27552	-	Ku domain protein	FIG00001754	isu;DNA_Repair_Base_Excision isu;DNA_ligases isu;Nonhomologous_End-Joining_in_Bacteria
NODE_7_length_126357_cov_42.436516	fig|6666666.34159.peg.1576	peg	NODE_7_length_126357_cov_42.436516_28895_28368	28895	28368	-	ThiJ/PfpI family protein	FIG00015923	isu;CBSS-176280.1.peg.1561
NODE_7_length_126357_cov_42.436516	fig|6666666.34159.peg.1577	peg	NODE_7_length_126357_cov_42.436516_29863_28958	29863	28958	-	hypothetical protein		
NODE_7_length_126357_cov_42.436516	fig|6666666.34159.peg.1578	peg	NODE_7_length_126357_cov_42.436516_29989_30552	29989	30552	+	hypothetical protein		
NODE_7_length_126357_cov_42.436516	fig|6666666.34159.peg.1579	peg	NODE_7_length_126357_cov_42.436516_31841_30594	31841	30594	-	ATP-dependent Clp protease ATP-binding subunit ClpX	FIG00000241	isu;Proteolysis_in_bacteria,_ATP-dependent
NODE_7_length_126357_cov_42.436516	fig|6666666.34159.peg.1580	peg	NODE_7_length_126357_cov_42.436516_32486_31878	32486	31878	-	ATP-dependent Clp protease proteolytic subunit (EC 3.4.21.92)	FIG00000028	icw(1);Proteolysis_in_bacteria,_ATP-dependent idu(1);cAMP_signaling_in_bacteria
NODE_7_length_126357_cov_42.436516	fig|6666666.34159.peg.1581	peg	NODE_7_length_126357_cov_42.436516_33886_32603	33886	32603	-	Cell division trigger factor (EC 5.2.1.8)	FIG00000286	ff
NODE_7_length_126357_cov_42.436516	fig|6666666.34159.rna.23	rna	NODE_7_length_126357_cov_42.436516_34032_33961	34032	33961	-	tRNA-Gly-GCC		idu(1);tRNAs
NODE_7_length_126357_cov_42.436516	fig|6666666.34159.rna.24	rna	NODE_7_length_126357_cov_42.436516_34120_34049	34120	34049	-	tRNA-Gly-GCC		idu(1);tRNAs
NODE_7_length_126357_cov_42.436516	fig|6666666.34159.rna.25	rna	NODE_7_length_126357_cov_42.436516_34230_34158	34230	34158	-	tRNA-Gly-CCC		isu;tRNAs
NODE_7_length_126357_cov_42.436516	fig|6666666.34159.peg.1582	peg	NODE_7_length_126357_cov_42.436516_34349_34483	34349	34483	+	hypothetical protein		
NODE_7_length_126357_cov_42.436516	fig|6666666.34159.peg.1583	peg	NODE_7_length_126357_cov_42.436516_34689_34853	34689	34853	+	hypothetical protein		
NODE_7_length_126357_cov_42.436516	fig|6666666.34159.peg.1584	peg	NODE_7_length_126357_cov_42.436516_34896_38396	34896	38396	+	FIG00899436: hypothetical protein	FIG00899434	ff
NODE_7_length_126357_cov_42.436516	fig|6666666.34159.peg.1585	peg	NODE_7_length_126357_cov_42.436516_38427_39527	38427	39527	+	Rod shape-determining protein MreB	FIG00065314	ff
NODE_7_length_126357_cov_42.436516	fig|6666666.34159.peg.1586	peg	NODE_7_length_126357_cov_42.436516_39571_41388	39571	41388	+	Phosphoenolpyruvate carboxykinase [GTP] (EC 4.1.1.32)	FIG00001643	isu;Pyruvate_metabolism_I:_anaplerotic_reactions,_PEP
NODE_7_length_126357_cov_42.436516	fig|6666666.34159.peg.1587	peg	NODE_7_length_126357_cov_42.436516_41419_42768	41419	42768	+	hypothetical protein	FIG00638284	ff
NODE_7_length_126357_cov_42.436516	fig|6666666.34159.peg.1588	peg	NODE_7_length_126357_cov_42.436516_42946_44703	42946	44703	+	hypothetical protein		
NODE_7_length_126357_cov_42.436516	fig|6666666.34159.peg.1589	peg	NODE_7_length_126357_cov_42.436516_45464_44700	45464	44700	-	hypothetical protein		
NODE_7_length_126357_cov_42.436516	fig|6666666.34159.peg.1590	peg	NODE_7_length_126357_cov_42.436516_45547_47478	45547	47478	+	hypothetical protein		
NODE_7_length_126357_cov_42.436516	fig|6666666.34159.peg.1591	peg	NODE_7_length_126357_cov_42.436516_48008_47475	48008	47475	-	hypothetical protein		
NODE_7_length_126357_cov_42.436516	fig|6666666.34159.peg.1592	peg	NODE_7_length_126357_cov_42.436516_48087_48203	48087	48203	+	hypothetical protein		
NODE_7_length_126357_cov_42.436516	fig|6666666.34159.peg.1593	peg	NODE_7_length_126357_cov_42.436516_48203_49279	48203	49279	+	hypothetical protein		
NODE_7_length_126357_cov_42.436516	fig|6666666.34159.peg.1594	peg	NODE_7_length_126357_cov_42.436516_50055_49309	50055	49309	-	Adenylate kinase (EC 2.7.4.3)	FIG00000089	isu;Purine_conversions
NODE_7_length_126357_cov_42.436516	fig|6666666.34159.peg.1595	peg	NODE_7_length_126357_cov_42.436516_50205_51881	50205	51881	+	hypothetical protein		
NODE_7_length_126357_cov_42.436516	fig|6666666.34159.peg.1596	peg	NODE_7_length_126357_cov_42.436516_51951_52694	51951	52694	+	hypothetical protein		
NODE_7_length_126357_cov_42.436516	fig|6666666.34159.peg.1597	peg	NODE_7_length_126357_cov_42.436516_52927_52691	52927	52691	-	hypothetical protein		
NODE_7_length_126357_cov_42.436516	fig|6666666.34159.peg.1598	peg	NODE_7_length_126357_cov_42.436516_53438_52908	53438	52908	-	tRNA-specific adenosine-34 deaminase (EC 3.5.4.-)	FIG00000291	idu(1);tRNA_processing idu(1);tRNA_modification_Bacteria
NODE_7_length_126357_cov_42.436516	fig|6666666.34159.peg.1599	peg	NODE_7_length_126357_cov_42.436516_54128_53445	54128	53445	-	hypothetical protein		
NODE_7_length_126357_cov_42.436516	fig|6666666.34159.peg.1600	peg	NODE_7_length_126357_cov_42.436516_54203_54457	54203	54457	+	hypothetical protein		
NODE_7_length_126357_cov_42.436516	fig|6666666.34159.peg.1601	peg	NODE_7_length_126357_cov_42.436516_54579_57017	54579	57017	+	hypothetical protein	FIG00638284	ff
NODE_7_length_126357_cov_42.436516	fig|6666666.34159.peg.1602	peg	NODE_7_length_126357_cov_42.436516_57175_57348	57175	57348	+	hypothetical protein		
NODE_7_length_126357_cov_42.436516	fig|6666666.34159.peg.1603	peg	NODE_7_length_126357_cov_42.436516_57384_57545	57384	57545	+	hypothetical protein		
NODE_7_length_126357_cov_42.436516	fig|6666666.34159.peg.1604	peg	NODE_7_length_126357_cov_42.436516_57716_58174	57716	58174	+	FIG000233: metal-dependent hydrolase	FIG01954455	ff
NODE_7_length_126357_cov_42.436516	fig|6666666.34159.peg.1605	peg	NODE_7_length_126357_cov_42.436516_58191_59546	58191	59546	+	Magnesium and cobalt efflux protein CorC	FIG00042627	isu;Magnesium_transport isu;Copper_homeostasis:_copper_tolerance
NODE_7_length_126357_cov_42.436516	fig|6666666.34159.peg.1606	peg	NODE_7_length_126357_cov_42.436516_59641_61203	59641	61203	+	Hydrolase (HAD superfamily)	FIG00510207	ff
NODE_7_length_126357_cov_42.436516	fig|6666666.34159.peg.1607	peg	NODE_7_length_126357_cov_42.436516_61305_63314	61305	63314	+	FIG00899452: hypothetical protein	FIG01339080	ff
NODE_7_length_126357_cov_42.436516	fig|6666666.34159.peg.1608	peg	NODE_7_length_126357_cov_42.436516_63339_63545	63339	63545	+	hypothetical protein		
NODE_7_length_126357_cov_42.436516	fig|6666666.34159.peg.1609	peg	NODE_7_length_126357_cov_42.436516_63542_64285	63542	64285	+	Ubiquinone/menaquinone biosynthesis methyltransferase UbiE (EC 2.1.1.-) @ 2-heptaprenyl-1,4-naphthoquinone methyltransferase (EC 2.1.1.163)	FIG00006851	isu;Menaquinone_and_Phylloquinone_Biosynthesis_--_gjo isu;Menaquinone_and_Phylloquinone_Biosynthes
NODE_7_length_126357_cov_42.436516	fig|6666666.34159.peg.1610	peg	NODE_7_length_126357_cov_42.436516_66155_64290	66155	64290	-	hypothetical protein	FIG00638284	ff
NODE_7_length_126357_cov_42.436516	fig|6666666.34159.peg.1611	peg	NODE_7_length_126357_cov_42.436516_67387_66257	67387	66257	-	O-succinylbenzoic acid--CoA ligase (EC 6.2.1.26)	FIG00000860	icw(1);Menaquinone_and_Phylloquinone_Biosynthesis_--_gjo icw(1);Menaquinone_and_Phylloquinone_Biosyn
NODE_7_length_126357_cov_42.436516	fig|6666666.34159.peg.1612	peg	NODE_7_length_126357_cov_42.436516_67517_69289	67517	69289	+	Pyruvate kinase (EC 2.7.1.40)	FIG00000043	isu;Glycerate_metabolism isu;Glycolysis_and_Gluconeogenesis isu;Pyruvate_metabolism_I:_anaplerotic_reaction
NODE_7_length_126357_cov_42.436516	fig|6666666.34159.peg.1613	peg	NODE_7_length_126357_cov_42.436516_70003_69473	70003	69473	-	hypothetical protein		
NODE_7_length_126357_cov_42.436516	fig|6666666.34159.peg.1614	peg	NODE_7_length_126357_cov_42.436516_70752_70027	70752	70027	-	Ribosomal RNA small subunit methyltransferase E (EC 2.1.1.-)	FIG00000248	isu;Heat_shock_dnaK_gene_cluster_extended isu;RNA_methylation
NODE_7_length_126357_cov_42.436516	fig|6666666.34159.peg.1615	peg	NODE_7_length_126357_cov_42.436516_72001_70754	72001	70754	-	FIG00493852: hypothetical protein	FIG00493226	ff
NODE_7_length_126357_cov_42.436516	fig|6666666.34159.peg.1616	peg	NODE_7_length_126357_cov_42.436516_72696_71989	72696	71989	-	Hypothetical protein YbbP, contains nucleotide-binding domain of DisA bacterial checkpoint controller	FIG00006310	ff
NODE_7_length_126357_cov_42.436516	fig|6666666.34159.peg.1617	peg	NODE_7_length_126357_cov_42.436516_73599_72808	73599	72808	-	Dihydropteroate synthase (EC 2.5.1.15)	FIG00000307	isu;Folate_Biosynthesis isu;Folate_biosynthesis_cluster
NODE_7_length_126357_cov_42.436516	fig|6666666.34159.peg.1618	peg	NODE_7_length_126357_cov_42.436516_75274_73592	75274	73592	-	hypothetical protein	FIG00638284	ff
NODE_7_length_126357_cov_42.436516	fig|6666666.34159.peg.1619	peg	NODE_7_length_126357_cov_42.436516_75602_76972	75602	76972	+	Cytochrome d ubiquinol oxidase subunit I (EC 1.10.3.-)	FIG00096593	isu;Terminal_cytochrome_d_ubiquinol_oxidases isu;Terminal_cytochrome_oxidases
NODE_7_length_126357_cov_42.436516	fig|6666666.34159.peg.1620	peg	NODE_7_length_126357_cov_42.436516_76974_78002	76974	78002	+	Cytochrome d ubiquinol oxidase subunit II (EC 1.10.3.-)	FIG00000390	icw(1);Terminal_cytochrome_d_ubiquinol_oxidases icw(1);Terminal_cytochrome_oxidases
NODE_7_length_126357_cov_42.436516	fig|6666666.34159.peg.1621	peg	NODE_7_length_126357_cov_42.436516_78017_79111	78017	79111	+	Linoleoyl-CoA desaturase (EC 1.14.19.3)	FIG00904516	ff
NODE_7_length_126357_cov_42.436516	fig|6666666.34159.peg.1622	peg	NODE_7_length_126357_cov_42.436516_80291_79083	80291	79083	-	Dihydrofolate synthase (EC 6.3.2.12) / Folylpolyglutamate synthase (EC 6.3.2.17)	FIG00000106	isu;Folate_Biosynthesis isu;Folate_Biosynthesis
NODE_7_length_126357_cov_42.436516	fig|6666666.34159.peg.1623	peg	NODE_7_length_126357_cov_42.436516_81130_80288	81130	80288	-	COG0613, Predicted metal-dependent phosphoesterases (PHP family)	FIG00031715	isu;tRNA_modification_Bacteria
NODE_7_length_126357_cov_42.436516	fig|6666666.34159.peg.1624	peg	NODE_7_length_126357_cov_42.436516_82023_81142	82023	81142	-	UDP-N-acetylenolpyruvoylglucosamine reductase (EC 1.1.1.158)	FIG00000163	isu;UDP-N-acetylmuramate_from_Fructose-6-phosphate_Biosynthesis
NODE_7_length_126357_cov_42.436516	fig|6666666.34159.peg.1625	peg	NODE_7_length_126357_cov_42.436516_82586_82023	82586	82023	-	Transcription termination protein NusB	FIG00016836	isu;Riboflavin_synthesis_cluster isu;Transcription_factors_bacterial
NODE_7_length_126357_cov_42.436516	fig|6666666.34159.peg.1626	peg	NODE_7_length_126357_cov_42.436516_82892_82719	82892	82719	-	hypothetical protein		
NODE_7_length_126357_cov_42.436516	fig|6666666.34159.peg.1627	peg	NODE_7_length_126357_cov_42.436516_83000_83404	83000	83404	+	hypothetical protein		

NODE_7_length_126357_cov_42.436516	fig|6666666.34159.peg.1628	peg	NODE_7_length_126357_cov_42.436516_84482_83439	84482	83439	-	Threonine dehydrogenase and related Zn-dependent dehydrogenases	FIG00053044	isu;Threonine_degradation
NODE_7_length_126357_cov_42.436516	fig|6666666.34159.peg.1629	peg	NODE_7_length_126357_cov_42.436516_86311_84581	86311	84581	-	hypothetical protein	FIG00638284	ff
NODE_7_length_126357_cov_42.436516	fig|6666666.34159.peg.1630	peg	NODE_7_length_126357_cov_42.436516_86657_86499	86657	86499	-	hypothetical protein		
NODE_7_length_126357_cov_42.436516	fig|6666666.34159.peg.1631	peg	NODE_7_length_126357_cov_42.436516_87223_87056	87223	87056	-	hypothetical protein		
NODE_7_length_126357_cov_42.436516	fig|6666666.34159.peg.1632	peg	NODE_7_length_126357_cov_42.436516_88261_87419	88261	87419	-	NAD kinase (EC 2.7.1.23)	FIG00000075	isu;NAD_and_NADP_cofactor_biosynthesis_global
NODE_7_length_126357_cov_42.436516	fig|6666666.34159.peg.1633	peg	NODE_7_length_126357_cov_42.436516_90230_88302	90230	88302	-	1-deoxy-D-xylulose 5-phosphate synthase (EC 2.2.1.7)	FIG00000343	isu;Thiamin_biosynthesis isu;Nonmevalonate_Branch_of_Isoprenoid_Biosynthesis isu;Pyridoxin_(Vitamin_B6)_Biosynthesis [illegible]
NODE_7_length_126357_cov_42.436516	fig|6666666.34159.peg.1634	peg	NODE_7_length_126357_cov_42.436516_90538_90230	90538	90230	-	putative exodeoxyribonuclease VII, small chain		
NODE_7_length_126357_cov_42.436516	fig|6666666.34159.peg.1635	peg	NODE_7_length_126357_cov_42.436516_91963_90548	91963	90548	-	Exodeoxyribonuclease VII large subunit (EC 3.1.11.6)	FIG00000323	isu;DNA_repair,_bacterial
NODE_7_length_126357_cov_42.436516	fig|6666666.34159.peg.1636	peg	NODE_7_length_126357_cov_42.436516_94266_91972	94266	91972	-	3'-to-5' exoribonuclease RNase R	FIG00000308	idu(1);RNA_processing_and_degradation,_bacterial
NODE_7_length_126357_cov_42.436516	fig|6666666.34159.peg.1637	peg	NODE_7_length_126357_cov_42.436516_95153_94341	95153	94341	-	hypothetical protein		
NODE_7_length_126357_cov_42.436516	fig|6666666.34159.peg.1638	peg	NODE_7_length_126357_cov_42.436516_95334_96515	95334	96515	+	Na+/H+ antiporter	FIG00008246	ff
NODE_7_length_126357_cov_42.436516	fig|6666666.34159.peg.1639	peg	NODE_7_length_126357_cov_42.436516_96538_96969	96538	96969	+	UspA domain protein		
NODE_7_length_126357_cov_42.436516	fig|6666666.34159.peg.1640	peg	NODE_7_length_126357_cov_42.436516_97182_99752	97182	99752	+	Leucyl-tRNA synthetase (EC 6.1.1.4)	FIG00000114	isu;tRNA_aminoacylation,_Leu
NODE_7_length_126357_cov_42.436516	fig|6666666.34159.peg.1641	peg	NODE_7_length_126357_cov_42.436516_99836_100663	99836	1E+05	+	Formamidopyrimidine-DNA glycosylase (EC 3.2.2.23)	FIG00035634	isu;DNA_Repair_Base_Excision
NODE_7_length_126357_cov_42.436516	fig|6666666.34159.peg.1642	peg	NODE_7_length_126357_cov_42.436516_100741_101520	100741	1E+05	+	hypothetical protein		
NODE_7_length_126357_cov_42.436516	fig|6666666.34159.peg.1643	peg	NODE_7_length_126357_cov_42.436516_102525_101890	102525	1E+05	-	hypothetical protein	FIG00638284	ff
NODE_7_length_126357_cov_42.436516	fig|6666666.34159.peg.1644	peg	NODE_7_length_126357_cov_42.436516_103175_103041	103175	1E+05	-	hypothetical protein		
NODE_7_length_126357_cov_42.436516	fig|6666666.34159.peg.1645	peg	NODE_7_length_126357_cov_42.436516_103422_105980	103422	1E+05	+	hypothetical protein		
NODE_7_length_126357_cov_42.436516	fig|6666666.34159.peg.1646	peg	NODE_7_length_126357_cov_42.436516_106631_106311	106631	1E+05	-	hypothetical protein		
NODE_7_length_126357_cov_42.436516	fig|6666666.34159.peg.1647	peg	NODE_7_length_126357_cov_42.436516_106835_106653	106835	1E+05	-	hypothetical protein		
NODE_7_length_126357_cov_42.436516	fig|6666666.34159.peg.1648	peg	NODE_7_length_126357_cov_42.436516_107092_107769	107092	1E+05	+	hypothetical protein	FIG00638284	ff
NODE_7_length_126357_cov_42.436516	fig|6666666.34159.peg.1649	peg	NODE_7_length_126357_cov_42.436516_107766_109028	107766	1E+05	+	Trehalose synthase, nucleoside diphosphate glucose dependent	FIG00159273	ff
NODE_7_length_126357_cov_42.436516	fig|6666666.34159.peg.1650	peg	NODE_7_length_126357_cov_42.436516_109025_109792	109025	1E+05	+	Trehalose-6-phosphate phosphatase (EC 3.1.3.12)	FIG00001349	isu;Trehalose_Biosynthesis
NODE_7_length_126357_cov_42.436516	fig|6666666.34159.peg.1651	peg	NODE_7_length_126357_cov_42.436516_109800_111296	109800	1E+05	+	Alpha,alpha-trehalose-phosphate synthase [UDP-forming] (EC 2.4.1.15)	FIG00001100	icw(1);Trehalose_Biosynthesis
NODE_7_length_126357_cov_42.436516	fig|6666666.34159.peg.1652	peg	NODE_7_length_126357_cov_42.436516_111548_111426	111548	1E+05	-	hypothetical protein		
NODE_7_length_126357_cov_42.436516	fig|6666666.34159.peg.1653	peg	NODE_7_length_126357_cov_42.436516_111811_112617	111811	1E+05	+	Flagellar hook-length control protein FliK	FIG00001074	idu(1);Flagellum
NODE_7_length_126357_cov_42.436516	fig|6666666.34159.peg.1654	peg	NODE_7_length_126357_cov_42.436516_112640_113617	112640	1E+05	+	hypothetical protein		
NODE_7_length_126357_cov_42.436516	fig|6666666.34159.peg.1655	peg	NODE_7_length_126357_cov_42.436516_114837_113764	114837	1E+05	-	UDP-3-O-[3-hydroxymyristoyl] glucosamine N-acyltransferase (EC 2.3.1.-)	FIG00138404	isu;Llipid_A_biosynthesis_cluster isu;KDO2-Lipid_A_biosynthesis
NODE_7_length_126357_cov_42.436516	fig|6666666.34159.peg.1656	peg	NODE_7_length_126357_cov_42.436516_115505_114912	115505	1E+05	-	Outer membrane protein H precursor	FIG00089707	isu;Periplasmic_Stress_Response icw(2);Llipid_A_biosynthesis_cluster
NODE_7_length_126357_cov_42.436516	fig|6666666.34159.peg.1657	peg	NODE_7_length_126357_cov_42.436516_117890_115539	117890	1E+05	-	Outer membrane protein assembly factor YaeT precursor		icw(1);Llipid_A_biosynthesis_cluster
NODE_7_length_126357_cov_42.436516	fig|6666666.34159.peg.1658	peg	NODE_7_length_126357_cov_42.436516_118647_118045	118647	1E+05	-	Recombination protein RecR	FIG00000190	isu;DNA_processing_cluster
NODE_7_length_126357_cov_42.436516	fig|6666666.34159.peg.1659	peg	NODE_7_length_126357_cov_42.436516_118791_119786	118791	1E+05	+	3-oxoacyl-[acyl-carrier-protein] synthase, KASIII (EC 2.3.1.41)	FIG00015100	isu;Fatty_Acid_Biosynthesis_FASII
NODE_7_length_126357_cov_42.436516	fig|6666666.34159.peg.1660	peg	NODE_7_length_126357_cov_42.436516_119807_120739	119807	1E+05	+	Malonyl CoA-acyl carrier protein transacylase (EC 2.3.1.39)	FIG00000319	icw(3);Fatty_Acid_Biosynthesis_FASII
NODE_7_length_126357_cov_42.436516	fig|6666666.34159.peg.1661	peg	NODE_7_length_126357_cov_42.436516_120754_121506	120754	1E+05	+	3-oxoacyl-[acyl-carrier protein] reductase (EC 1.1.1.100)	FIG00621114	idu(12);CBSS-246196.1.peg.364 icw(1);Fatty_Acid_Biosynthesis_FASII
NODE_7_length_126357_cov_42.436516	fig|6666666.34159.peg.1662	peg	NODE_7_length_126357_cov_42.436516_121546_121800	121546	1E+05	+	Acyl carrier protein	FIG00002328	icw(2);Fatty_Acid_Biosynthesis_FASII
NODE_7_length_126357_cov_42.436516	fig|6666666.34159.peg.1663	peg	NODE_7_length_126357_cov_42.436516_121951_121832	121951	1E+05	-	hypothetical protein		
NODE_7_length_126357_cov_42.436516	fig|6666666.34159.peg.1664	peg	NODE_7_length_126357_cov_42.436516_122021_122533	122021	1E+05	+	hypothetical protein		
NODE_7_length_126357_cov_42.436516	fig|6666666.34159.peg.1665	peg	NODE_7_length_126357_cov_42.436516_122540_123235	122540	1E+05	+	DNA polymerase III epsilon subunit (EC 2.7.7.7)	FIG00008411	isu;CBSS-228410.1.peg.134 isu;CBSS-342610.3.peg.1536
NODE_7_length_126357_cov_42.436516	fig|6666666.34159.peg.1666	peg	NODE_7_length_126357_cov_42.436516_123225_124040	123225	1E+05	+	Nucleoside-diphosphate-sugar epimerases	FIG00002924	isu;CBSS-296591.1.peg.2330
NODE_7_length_126357_cov_42.436516	fig|6666666.34159.peg.1667	peg	NODE_7_length_126357_cov_42.436516_124056_124739	124056	1E+05	+	Probable 2-phosphosulfolactate phosphatase (EC 3.1.3.71)		
NODE_7_length_126357_cov_42.436516	fig|6666666.34159.peg.1668	peg	NODE_7_length_126357_cov_42.436516_124754_125722	124754	1E+05	+	hypothetical protein	FIG00638284	ff
NODE_7_length_126357_cov_42.436516	fig|6666666.34159.peg.1669	peg	NODE_7_length_126357_cov_42.436516_125883_125719	125883	1E+05	-	Protein of unknown function DUF1328		
NODE_7_length_126357_cov_42.436516	fig|6666666.34159.peg.1670	peg	NODE_7_length_126357_cov_42.436516_126033_126227	126033	1E+05	+	hypothetical protein		
NODE_87_length_41778_cov_41.815182	fig|6666666.34159.peg.1671	peg	NODE_87_length_41778_cov_41.815182_401_72	401	72	-	hypothetical protein		
NODE_87_length_41778_cov_41.815182	fig|6666666.34159.peg.1672	peg	NODE_87_length_41778_cov_41.815182_662_841	662	841	+	hypothetical protein		
NODE_87_length_41778_cov_41.815182	fig|6666666.34159.peg.1673	peg	NODE_87_length_41778_cov_41.815182_829_1056	829	1056	+	Short chain dehydrogenase family protein		
NODE_87_length_41778_cov_41.815182	fig|6666666.34159.peg.1674	peg	NODE_87_length_41778_cov_41.815182_2367_1066	2367	1066	-	hypothetical protein		
NODE_87_length_41778_cov_41.815182	fig|6666666.34159.peg.1675	peg	NODE_87_length_41778_cov_41.815182_3299_2490	3299	2490	-	hypothetical protein		
NODE_87_length_41778_cov_41.815182	fig|6666666.34159.peg.1676	peg	NODE_87_length_41778_cov_41.815182_3667_3317	3667	3317	-	hypothetical protein		
NODE_87_length_41778_cov_41.815182	fig|6666666.34159.peg.1677	peg	NODE_87_length_41778_cov_41.815182_4417_4031	4417	4031	-	glycine-rich protein		
NODE_87_length_41778_cov_41.815182	fig|6666666.34159.peg.1678	peg	NODE_87_length_41778_cov_41.815182_5683_4754	5683	4754	-	Signal recognition particle receptor protein FtsY (=alpha subunit) (TC 3.A.5.1.1)	FIG00000095	isu;Bacterial_signal_recognition_particle_(SRP)
NODE_87_length_41778_cov_41.815182	fig|6666666.34159.peg.1679	peg	NODE_87_length_41778_cov_41.815182_5772_6935	5772	6935	+	Succinyl-CoA ligase [ADP-forming] beta chain (EC 6.2.1.5)	FIG00000337	icw(1);TCA_Cycle
NODE_87_length_41778_cov_41.815182	fig|6666666.34159.peg.1680	peg	NODE_87_length_41778_cov_41.815182_6950_7828	6950	7828	+	Succinyl-CoA ligase [ADP-forming] alpha chain (EC 6.2.1.5)	FIG00038611	isu;TCA_Cycle
NODE_87_length_41778_cov_41.815182	fig|6666666.34159.peg.1681	peg	NODE_87_length_41778_cov_41.815182_7825_8994	7825	8994	+	hypothetical protein	FIG00638284	ff
NODE_87_length_41778_cov_41.815182	fig|6666666.34159.peg.1682	peg	NODE_87_length_41778_cov_41.815182_10383_9172	10383	9172	-	Arsenic efflux pump protein	FIG00081580	idu(1);Arsenic_resistance
NODE_87_length_41778_cov_41.815182	fig|6666666.34159.peg.1683	peg	NODE_87_length_41778_cov_41.815182_10823_10386	10823	10386	-	Integral membrane transport protein		
NODE_87_length_41778_cov_41.815182	fig|6666666.34159.peg.1684	peg	NODE_87_length_41778_cov_41.815182_11596_11108	11596	11108	-	major facilitator superfamily MFS_1		
NODE_87_length_41778_cov_41.815182	fig|6666666.34159.peg.1685	peg	NODE_87_length_41778_cov_41.815182_12367_11687	12367	11687	-	Thiaminase II (EC 3.5.99.2)	FIG00034727	isu;Thiamin_biosynthesis
NODE_87_length_41778_cov_41.815182	fig|6666666.34159.peg.1686	peg	NODE_87_length_41778_cov_41.815182_13424_12423	13424	12423	-	Thiamine-monophosphate kinase (EC 2.7.4.16)	FIG00000570	isu;Riboflavin_synthesis_cluster icw(1);Thiamin_biosynthesis

NODE_87_length_41778_cov_41.815182	fig|6666666.34159.peg.1687	peg	NODE_87_length_41778_cov_41.815182_13595_15403	13595	15403	+	Aspartyl-tRNA synthetase (EC 6.1.1.12) @ Aspartyl-tRNA(Asn) synthetase (EC 6.1.1.23)	FIG00000656	isu;tRNA_aminoacylation,_Asp_and_Asn isu;tRNA_aminoacylation,_Asp_and_Asn
NODE_87_length_41778_cov_41.815182	fig|6666666.34159.peg.1688	peg	NODE_87_length_41778_cov_41.815182_16282_15455	16282	15455	-	hypothetical protein		
NODE_87_length_41778_cov_41.815182	fig|6666666.34159.peg.1689	peg	NODE_87_length_41778_cov_41.815182_16492_16370	16492	16370	-	hypothetical protein		
NODE_87_length_41778_cov_41.815182	fig|6666666.34159.peg.1690	peg	NODE_87_length_41778_cov_41.815182_16647_16763	16647	16763	+	hypothetical protein		
NODE_87_length_41778_cov_41.815182	fig|6666666.34159.peg.1691	peg	NODE_87_length_41778_cov_41.815182_18301_16745	18301	16745	-	ADP/ATP Translocase, NTT1	FIG01345036	ff
NODE_87_length_41778_cov_41.815182	fig|6666666.34159.peg.1692	peg	NODE_87_length_41778_cov_41.815182_18455_18330	18455	18330	-	hypothetical protein		
NODE_87_length_41778_cov_41.815182	fig|6666666.34159.peg.1693	peg	NODE_87_length_41778_cov_41.815182_19992_18463	19992	18463	-	ADP/ATP Translocase, NTT1	FIG01345036	ff
NODE_87_length_41778_cov_41.815182	fig|6666666.34159.peg.1694	peg	NODE_87_length_41778_cov_41.815182_20043_20174	20043	20174	+	hypothetical protein		
NODE_87_length_41778_cov_41.815182	fig|6666666.34159.peg.1695	peg	NODE_87_length_41778_cov_41.815182_21503_20298	21503	20298	-	Phosphoglycerate kinase (EC 2.7.2.3)	FIG00054459	isu;Calvin-Benson_cycle isu;Glycolysis_and_Gluconeogenesis
NODE_87_length_41778_cov_41.815182	fig|6666666.34159.peg.1696	peg	NODE_87_length_41778_cov_41.815182_21590_22237	21590	22237	+	Pyridoxamine 5'-phosphate oxidase (EC 1.4.3.5)	FIG00133002	isu;Pyridoxin_(Vitamin_B6)_Biosynthesis
NODE_87_length_41778_cov_41.815182	fig|6666666.34159.peg.1697	peg	NODE_87_length_41778_cov_41.815182_25806_22234	25806	22234	-	hypothetical protein	FIG00638284	ff
NODE_87_length_41778_cov_41.815182	fig|6666666.34159.peg.1698	peg	NODE_87_length_41778_cov_41.815182_29200_25943	29200	25943	-	hypothetical protein		
NODE_87_length_41778_cov_41.815182	fig|6666666.34159.peg.1699	peg	NODE_87_length_41778_cov_41.815182_29840_30205	29840	30205	+	DnaK Suppressor	FIG00493161	ff
NODE_87_length_41778_cov_41.815182	fig|6666666.34159.peg.1700	peg	NODE_87_length_41778_cov_41.815182_30208_30726	30208	30726	+	Lipoprotein signal peptidase (EC 3.4.23.36)	FIG00000081	isu;Signal_peptidase isu;Sex_pheromones_in_Enterococcus_faecalis_and_other_Firmicutes isu;CBSS-36873.1.peg.4752
NODE_87_length_41778_cov_41.815182	fig|6666666.34159.peg.1701	peg	NODE_87_length_41778_cov_41.815182_30723_31418	30723	31418	+	hypothetical protein	FIG00638284	ff
NODE_87_length_41778_cov_41.815182	fig|6666666.34159.peg.1702	peg	NODE_87_length_41778_cov_41.815182_31444_32208	31444	32208	+	Glycerophosphoryl diester phosphodiesterase (EC 3.1.4.46)	FIG00002239	isu;Glycerol_and_Glycerol-3-phosphate_Uptake_and_Utilization isu;CBSS-176299.4.peg.1996A
NODE_87_length_41778_cov_41.815182	fig|6666666.34159.peg.1703	peg	NODE_87_length_41778_cov_41.815182_33509_32205	33509	32205	-	Enolase (EC 4.2.1.11)	FIG00000118	isu;Glycolysis_and_Gluconeogenesis
NODE_87_length_41778_cov_41.815182	fig|6666666.34159.peg.1704	peg	NODE_87_length_41778_cov_41.815182_34688_33537	34688	33537	-	Ribosome small subunit-stimulated GTPase EngC	FIG00000464	ff
NODE_87_length_41778_cov_41.815182	fig|6666666.34159.peg.1705	peg	NODE_87_length_41778_cov_41.815182_35456_34821	35456	34821	-	hypothetical protein		
NODE_87_length_41778_cov_41.815182	fig|6666666.34159.peg.1706	peg	NODE_87_length_41778_cov_41.815182_35522_37156	35522	37156	+	Beta-hexosaminidase (EC 3.2.1.52)	FIG00001088	isu;N-Acetyl-Galactosamine_and_Galactosamine_Utilization isu;Chitin_and_N-acetylglucosamine_utilization
NODE_87_length_41778_cov_41.815182	fig|6666666.34159.peg.1707	peg	NODE_87_length_41778_cov_41.815182_37135_37932	37135	37932	+	FIG00441953: hypothetical protein	FIG00441951	ff
NODE_87_length_41778_cov_41.815182	fig|6666666.34159.peg.1708	peg	NODE_87_length_41778_cov_41.815182_38104_38283	38104	38283	+	SSU ribosomal protein S21p	FIG00000178	ff
NODE_87_length_41778_cov_41.815182	fig|6666666.34159.peg.1709	peg	NODE_87_length_41778_cov_41.815182_38488_39639	38488	39639	+	Chaperone protein DnaJ	FIG00000070	idu(2);Heat_shock_dnaK_gene_cluster_extended idu(2);Protein_chaperones
NODE_87_length_41778_cov_41.815182	fig|6666666.34159.peg.1710	peg	NODE_87_length_41778_cov_41.815182_40507_39710	40507	39710	-	metal dependent hydrolase, putative	FIG01338207	ff
NODE_8_length_386799_cov_42.541500	fig|6666666.34159.peg.1711	peg	NODE_8_length_386799_cov_42.541500_680_147	680	147	-	hypothetical protein		
NODE_8_length_386799_cov_42.541500	fig|6666666.34159.peg.1712	peg	NODE_8_length_386799_cov_42.541500_940_1059	940	1059	+	hypothetical protein		
NODE_8_length_386799_cov_42.541500	fig|6666666.34159.peg.1713	peg	NODE_8_length_386799_cov_42.541500_2203_1166	2203	1166	-	S-adenosylmethionine:tRNA ribosyltransferase-isomerase (EC 5.-.-.-)	FIG00000334	isu;tRNA_modification_Bacteria isu;Queuosine-Archaeosine_Biosynthesis isu;CBSS-211586.1.peg.2832
NODE_8_length_386799_cov_42.541500	fig|6666666.34159.peg.1714	peg	NODE_8_length_386799_cov_42.541500_3122_2262	3122	2262	-	hypothetical protein		
NODE_8_length_386799_cov_42.541500	fig|6666666.34159.peg.1715	peg	NODE_8_length_386799_cov_42.541500_4135_3122	4135	3122	-	Holliday junction DNA helicase RuvB	FIG00000352	isu;RuvABC_plus_a_hypothetical
NODE_8_length_386799_cov_42.541500	fig|6666666.34159.peg.1716	peg	NODE_8_length_386799_cov_42.541500_5507_4137	5507	4137	-	Sodium:neurotransmitter symporter		
NODE_8_length_386799_cov_42.541500	fig|6666666.34159.peg.1717	peg	NODE_8_length_386799_cov_42.541500_5674_6168	5674	6168	+	hypothetical protein		
NODE_8_length_386799_cov_42.541500	fig|6666666.34159.peg.1718	peg	NODE_8_length_386799_cov_42.541500_6372_7352	6372	7352	+	hypothetical protein		
NODE_8_length_386799_cov_42.541500	fig|6666666.34159.peg.1719	peg	NODE_8_length_386799_cov_42.541500_7990_7349	7990	7349	-	ribosomal RNA adenine dimethylase domain protein		
NODE_8_length_386799_cov_42.541500	fig|6666666.34159.peg.1720	peg	NODE_8_length_386799_cov_42.541500_8328_8008	8328	8008	-	hypothetical protein		
NODE_8_length_386799_cov_42.541500	fig|6666666.34159.peg.1721	peg	NODE_8_length_386799_cov_42.541500_9077_8346	9077	8346	-	dienelactone hydrolase family protein		
NODE_8_length_386799_cov_42.541500	fig|6666666.34159.peg.1722	peg	NODE_8_length_386799_cov_42.541500_9212_9649	9212	9649	+	hypothetical protein	FIG00638284	ff
NODE_8_length_386799_cov_42.541500	fig|6666666.34159.peg.1723	peg	NODE_8_length_386799_cov_42.541500_9712_10962	9712	10962	+	hypothetical protein		
NODE_8_length_386799_cov_42.541500	fig|6666666.34159.peg.1724	peg	NODE_8_length_386799_cov_42.541500_11148_12428	11148	12428	+	hypothetical protein		
NODE_8_length_386799_cov_42.541500	fig|6666666.34159.peg.1725	peg	NODE_8_length_386799_cov_42.541500_13688_12477	13688	12477	-	hypothetical protein		
NODE_8_length_386799_cov_42.541500	fig|6666666.34159.peg.1726	peg	NODE_8_length_386799_cov_42.541500_21247_13841	21247	13841	-	hypothetical protein	FIG00638284	ff
NODE_8_length_386799_cov_42.541500	fig|6666666.34159.peg.1727	peg	NODE_8_length_386799_cov_42.541500_21585_22877	21585	22877	+	MiaB family protein, possibly involved in tRNA or rRNA modification	FIG00002029	ff
NODE_8_length_386799_cov_42.541500	fig|6666666.34159.peg.1728	peg	NODE_8_length_386799_cov_42.541500_22882_23949	22882	23949	+	FIG00494467: hypothetical protein	FIG00494465	ff
NODE_8_length_386799_cov_42.541500	fig|6666666.34159.peg.1729	peg	NODE_8_length_386799_cov_42.541500_23987_26155	23987	26155	+	1,4-alpha-glucan (glycogen) branching enzyme, GH-13-type (EC 2.4.1.18)	FIG00024930	isu;Trehalose_Biosynthesis isu;Glycogen_metabolism isu;Glycogen_metabolism_cluster
NODE_8_length_386799_cov_42.541500	fig|6666666.34159.peg.1730	peg	NODE_8_length_386799_cov_42.541500_26349_27965	26349	27965	+	Cold-shock DEAD-box protein A	FIG00001381	idu(1);ATP-dependent_RNA_helicases,_bacterial
NODE_8_length_386799_cov_42.541500	fig|6666666.34159.peg.1731	peg	NODE_8_length_386799_cov_42.541500_28865_28026	28865	28026	-	hypothetical protein		
NODE_8_length_386799_cov_42.541500	fig|6666666.34159.peg.1732	peg	NODE_8_length_386799_cov_42.541500_30431_28977	30431	28977	-	ADA regulatory protein	FIG00006424	isu;DNA_repair,_bacterial isu;CBSS-393124.3.peg.2657
NODE_8_length_386799_cov_42.541500	fig|6666666.34159.peg.1733	peg	NODE_8_length_386799_cov_42.541500_31979_30591	31979	30591	-	Multi antimicrobial extrusion protein (Na(+)/drug antiporter), MATE family of MDR efflux pumps	FIG00000402	isu;Multidrug_Resistance_Efflux_Pumps
NODE_8_length_386799_cov_42.541500	fig|6666666.34159.peg.1734	peg	NODE_8_length_386799_cov_42.541500_32779_32225	32779	32225	-	hypothetical protein		
NODE_8_length_386799_cov_42.541500	fig|6666666.34159.peg.1735	peg	NODE_8_length_386799_cov_42.541500_34641_32779	34641	32779	-	hypothetical protein	FIG00638284	ff
NODE_8_length_386799_cov_42.541500	fig|6666666.34159.peg.1736	peg	NODE_8_length_386799_cov_42.541500_35675_34638	35675	34638	-	hypothetical protein		
NODE_8_length_386799_cov_42.541500	fig|6666666.34159.peg.1737	peg	NODE_8_length_386799_cov_42.541500_38248_35672	38248	35672	-	hypothetical protein	FIG00638284	ff
NODE_8_length_386799_cov_42.541500	fig|6666666.34159.peg.1738	peg	NODE_8_length_386799_cov_42.541500_41335_38312	41335	38312	-	hypothetical protein	FIG00638284	ff
NODE_8_length_386799_cov_42.541500	fig|6666666.34159.peg.1739	peg	NODE_8_length_386799_cov_42.541500_41768_41352	41768	41352	-	hypothetical protein		
NODE_8_length_386799_cov_42.541500	fig|6666666.34159.peg.1740	peg	NODE_8_length_386799_cov_42.541500_41847_41731	41847	41731	-	hypothetical protein		
NODE_8_length_386799_cov_42.541500	fig|6666666.34159.peg.1741	peg	NODE_8_length_386799_cov_42.541500_43655_41898	43655	41898	-	hypothetical protein	FIG00638284	ff
NODE_8_length_386799_cov_42.541500	fig|6666666.34159.peg.1742	peg	NODE_8_length_386799_cov_42.541500_44858_43770	44858	43770	-	hypothetical protein		
NODE_8_length_386799_cov_42.541500	fig|6666666.34159.peg.1743	peg	NODE_8_length_386799_cov_42.541500_45244_46827	45244	46827	+	hypothetical protein	FIG00638284	ff
NODE_8_length_386799_cov_42.541500	fig|6666666.34159.peg.1744	peg	NODE_8_length_386799_cov_42.541500_46909_47100	46909	47100	+	hypothetical protein		
NODE_8_length_386799_cov_42.541500	fig|6666666.34159.peg.1745	peg	NODE_8_length_386799_cov_42.541500_47061_47237	47061	47237	+	hypothetical protein		

NODE_8_length_386799_cov_42.541500	fig|6666666.34159.peg.1746	peg	NODE_8_length_386799_cov_42.541500_47694_47218	47694	47218	-	hypothetical protein		
NODE_8_length_386799_cov_42.541500	fig|6666666.34159.peg.1747	peg	NODE_8_length_386799_cov_42.541500_48338_47700	48338	47700	-	Ribosomal subunit interface protein	FIG00001306	isu;Ribosome_activity_modulation
NODE_8_length_386799_cov_42.541500	fig|6666666.34159.peg.1748	peg	NODE_8_length_386799_cov_42.541500_48353_48484	48353	48484	+	hypothetical protein		
NODE_8_length_386799_cov_42.541500	fig|6666666.34159.peg.1749	peg	NODE_8_length_386799_cov_42.541500_48651_48917	48651	48917	+	hypothetical protein		
NODE_8_length_386799_cov_42.541500	fig|6666666.34159.peg.1750	peg	NODE_8_length_386799_cov_42.541500_49314_48922	49314	48922	-	hypothetical protein		
NODE_8_length_386799_cov_42.541500	fig|6666666.34159.peg.1751	peg	NODE_8_length_386799_cov_42.541500_50853_49759	50853	49759	-	Ribosomal RNA large subunit methyltransferase N (EC 2.1.1.-)	FIG00000293	idu(1);RNA_methylation
NODE_8_length_386799_cov_42.541500	fig|6666666.34159.rna.26	rna	NODE_8_length_386799_cov_42.541500_50947_50874	50947	50874	-	tRNA-Arg-TCG		
NODE_8_length_386799_cov_42.541500	fig|6666666.34159.peg.1752	peg	NODE_8_length_386799_cov_42.541500_51081_51266	51081	51266	+	hypothetical protein		
NODE_8_length_386799_cov_42.541500	fig|6666666.34159.peg.1753	peg	NODE_8_length_386799_cov_42.541500_51282_52847	51282	52847	+	Lysyl-tRNA synthetase (class II) (EC 6.1.1.6)	FIG00000300	isu;tRNA_aminoacylation,_Lys
NODE_8_length_386799_cov_42.541500	fig|6666666.34159.peg.1754	peg	NODE_8_length_386799_cov_42.541500_54225_52906	54225	52906	-	MORN repeat protein		
NODE_8_length_386799_cov_42.541500	fig|6666666.34159.peg.1755	peg	NODE_8_length_386799_cov_42.541500_55892_54321	55892	54321	-	MORN repeat protein		
NODE_8_length_386799_cov_42.541500	fig|6666666.34159.peg.1756	peg	NODE_8_length_386799_cov_42.541500_57459_56026	57459	56026	-	MORN repeat protein		
NODE_8_length_386799_cov_42.541500	fig|6666666.34159.peg.1757	peg	NODE_8_length_386799_cov_42.541500_57675_57794	57675	57794	+	hypothetical protein		
NODE_8_length_386799_cov_42.541500	fig|6666666.34159.peg.1758	peg	NODE_8_length_386799_cov_42.541500_58764_57850	58764	57850	-	putative type III secretion protein SctJ		
NODE_8_length_386799_cov_42.541500	fig|6666666.34159.peg.1759	peg	NODE_8_length_386799_cov_42.541500_59270_59139	59270	59139	-	hypothetical protein		
NODE_8_length_386799_cov_42.541500	fig|6666666.34159.peg.1760	peg	NODE_8_length_386799_cov_42.541500_59533_60099	59533	60099	+	hypothetical protein		
NODE_8_length_386799_cov_42.541500	fig|6666666.34159.peg.1761	peg	NODE_8_length_386799_cov_42.541500_60316_61560	60316	61560	+	hypothetical protein		
NODE_8_length_386799_cov_42.541500	fig|6666666.34159.peg.1762	peg	NODE_8_length_386799_cov_42.541500_61923_61561	61923	61561	-	hypothetical protein		
NODE_8_length_386799_cov_42.541500	fig|6666666.34159.peg.1763	peg	NODE_8_length_386799_cov_42.541500_62168_61926	62168	61926	-	hypothetical protein		
NODE_8_length_386799_cov_42.541500	fig|6666666.34159.peg.1764	peg	NODE_8_length_386799_cov_42.541500_63634_62165	63634	62165	-	Cysteinyl-tRNA synthetase (EC 6.1.1.16)	FIG00000086	isu;tRNA_aminoacylation,_Cys isu;Conserved_gene_cluster_possibly_involved_in_RNA_metabolism
NODE_8_length_386799_cov_42.541500	fig|6666666.34159.peg.1765	peg	NODE_8_length_386799_cov_42.541500_66378_63640	66378	63640	-	Long-chain-fatty-acid--CoA ligase (EC 6.2.1.3)	FIG00018699	idu(5);n-Phenylalkanoic_acid_degradation idu(5);Fatty_acid_metabolism_cluster idu(5);Biotin_synthesis_cluster
NODE_8_length_386799_cov_42.541500	fig|6666666.34159.peg.1766	peg	NODE_8_length_386799_cov_42.541500_66904_66575	66904	66575	-	hypothetical protein		
NODE_8_length_386799_cov_42.541500	fig|6666666.34159.peg.1767	peg	NODE_8_length_386799_cov_42.541500_68346_67051	68346	67051	-	Predicted ATPase related to phosphate starvation-inducible protein PhoH	FIG00075426	ff
NODE_8_length_386799_cov_42.541500	fig|6666666.34159.peg.1768	peg	NODE_8_length_386799_cov_42.541500_68578_69294	68578	69294	+	hypothetical protein		
NODE_8_length_386799_cov_42.541500	fig|6666666.34159.peg.1769	peg	NODE_8_length_386799_cov_42.541500_69485_69354	69485	69354	-	hypothetical protein		
NODE_8_length_386799_cov_42.541500	fig|6666666.34159.peg.1770	peg	NODE_8_length_386799_cov_42.541500_69852_71090	69852	71090	+	hypothetical protein	FIG00638284	ff
NODE_8_length_386799_cov_42.541500	fig|6666666.34159.peg.1771	peg	NODE_8_length_386799_cov_42.541500_71117_71779	71117	71779	+	Putative preQ0 transporter	FIG00002792	isu;Queuosine-Archaeosine_Biosynthesis
NODE_8_length_386799_cov_42.541500	fig|6666666.34159.peg.1772	peg	NODE_8_length_386799_cov_42.541500_71784_72470	71784	72470	+	hypothetical protein		
NODE_8_length_386799_cov_42.541500	fig|6666666.34159.peg.1773	peg	NODE_8_length_386799_cov_42.541500_72454_73074	72454	73074	+	SOS-response repressor and protease LexA (EC 3.4.21.88)	FIG00000510	isu;DNA_repair,_bacterial_UmuCD_system isu;DNA_repair,_bacterial
NODE_8_length_386799_cov_42.541500	fig|6666666.34159.peg.1774	peg	NODE_8_length_386799_cov_42.541500_73933_73088	73933	73088	-	CDP-diacylglycerol--serine O-phosphatidyltransferase (EC 2.7.8.8)	FIG00000775	ff
NODE_8_length_386799_cov_42.541500	fig|6666666.34159.peg.1775	peg	NODE_8_length_386799_cov_42.541500_74801_74049	74801	74049	-	ABC transporter, ATP-binding component		
NODE_8_length_386799_cov_42.541500	fig|6666666.34159.peg.1776	peg	NODE_8_length_386799_cov_42.541500_74908_76107	74908	76107	+	Aminopeptidase YpdF (MP-, MA-, MS-, AP-, NP- specific)	FIG00135469	idu(1);Protein_degradation
NODE_8_length_386799_cov_42.541500	fig|6666666.34159.peg.1777	peg	NODE_8_length_386799_cov_42.541500_76191_77468	76191	77468	+	Tyrosine-specific transport protein	FIG00004484	ff
NODE_8_length_386799_cov_42.541500	fig|6666666.34159.peg.1778	peg	NODE_8_length_386799_cov_42.541500_77484_77984	77484	77984	+	hypothetical protein		
NODE_8_length_386799_cov_42.541500	fig|6666666.34159.peg.1779	peg	NODE_8_length_386799_cov_42.541500_77996_79822	77996	79822	+	DnaK-related protein	FIG00454936	ff
NODE_8_length_386799_cov_42.541500	fig|6666666.34159.peg.1780	peg	NODE_8_length_386799_cov_42.541500_79895_80911	79895	80911	+	cell envelope integrity inner membrane protein TolA		
NODE_8_length_386799_cov_42.541500	fig|6666666.34159.peg.1781	peg	NODE_8_length_386799_cov_42.541500_82000_80957	82000	80957	-	hypothetical protein		
NODE_8_length_386799_cov_42.541500	fig|6666666.34159.peg.1782	peg	NODE_8_length_386799_cov_42.541500_82185_82943	82185	82943	+	FIG00551148: hypothetical protein	FIG00551144	ff
NODE_8_length_386799_cov_42.541500	fig|6666666.34159.peg.1783	peg	NODE_8_length_386799_cov_42.541500_82940_83917	82940	83917	+	Positive regulator of CheA protein activity (CheW)		isu;Two-component_regulatory_systems_in_Campylobacter
NODE_8_length_386799_cov_42.541500	fig|6666666.34159.peg.1784	peg	NODE_8_length_386799_cov_42.541500_83927_85408	83927	85408	+	Methyl-accepting chemotaxis protein		
NODE_8_length_386799_cov_42.541500	fig|6666666.34159.peg.1785	peg	NODE_8_length_386799_cov_42.541500_85414_87618	85414	87618	+	Signal transduction histidine kinase CheA (EC 2.7.3.-)	FIG00000571	isu;Flagellar_motility icw(1);Two-component_regulatory_systems_in_Campylobacter
NODE_8_length_386799_cov_42.541500	fig|6666666.34159.peg.1786	peg	NODE_8_length_386799_cov_42.541500_87615_88847	87615	88847	+	Adenylate cyclase (EC 4.6.1.1)	FIG00001294	isu;cAMP_signaling_in_bacteria
NODE_8_length_386799_cov_42.541500	fig|6666666.34159.peg.1787	peg	NODE_8_length_386799_cov_42.541500_89023_88850	89023	88850	-	hypothetical protein		
NODE_8_length_386799_cov_42.541500	fig|6666666.34159.peg.1788	peg	NODE_8_length_386799_cov_42.541500_90457_89084	90457	89084	-	putative outer membrane protein		
NODE_8_length_386799_cov_42.541500	fig|6666666.34159.peg.1789	peg	NODE_8_length_386799_cov_42.541500_91601_90483	91601	90483	-	hypothetical protein		
NODE_8_length_386799_cov_42.541500	fig|6666666.34159.peg.1790	peg	NODE_8_length_386799_cov_42.541500_91851_91720	91851	91720	-	hypothetical protein		
NODE_8_length_386799_cov_42.541500	fig|6666666.34159.peg.1791	peg	NODE_8_length_386799_cov_42.541500_91810_91944	91810	91944	+	hypothetical protein		
NODE_8_length_386799_cov_42.541500	fig|6666666.34159.peg.1792	peg	NODE_8_length_386799_cov_42.541500_92421_91954	92421	91954	-	MutT/nudix family protein	FIG01323436	ff
NODE_8_length_386799_cov_42.541500	fig|6666666.34159.peg.1793	peg	NODE_8_length_386799_cov_42.541500_92580_93479	92580	93479	+	hypothetical protein	FIG00638284	ff
NODE_8_length_386799_cov_42.541500	fig|6666666.34159.peg.1794	peg	NODE_8_length_386799_cov_42.541500_93628_93482	93628	93482	-	hypothetical protein		
NODE_8_length_386799_cov_42.541500	fig|6666666.34159.peg.1795	peg	NODE_8_length_386799_cov_42.541500_93685_94413	93685	94413	+	hypothetical protein		
NODE_8_length_386799_cov_42.541500	fig|6666666.34159.peg.1796	peg	NODE_8_length_386799_cov_42.541500_94889_94422	94889	94422	-	putative chloramphenicol 3-O phosphotransferase		
NODE_8_length_386799_cov_42.541500	fig|6666666.34159.peg.1797	peg	NODE_8_length_386799_cov_42.541500_95624_95043	95624	95043	-	hypothetical protein		
NODE_8_length_386799_cov_42.541500	fig|6666666.34159.peg.1798	peg	NODE_8_length_386799_cov_42.541500_95803_96570	95803	96570	+	hypothetical protein	FIG00638284	ff
NODE_8_length_386799_cov_42.541500	fig|6666666.34159.peg.1799	peg	NODE_8_length_386799_cov_42.541500_96578_97651	96578	97651	+	FIG00947689: hypothetical protein	FIG00947688	ff
NODE_8_length_386799_cov_42.541500	fig|6666666.34159.peg.1800	peg	NODE_8_length_386799_cov_42.541500_97686_98651	97686	98651	+	hypothetical protein	FIG00638284	ff
NODE_8_length_386799_cov_42.541500	fig|6666666.34159.peg.1801	peg	NODE_8_length_386799_cov_42.541500_98805_99149	98805	99149	+	Possible carboxymuconolactone decarboxylase family protein (EC 4.1.1.44)	FIG01384312	ff
NODE_8_length_386799_cov_42.541500	fig|6666666.34159.peg.1802	peg	NODE_8_length_386799_cov_42.541500_99375_100043	99375	1E+05	+	hypothetical protein		
NODE_8_length_386799_cov_42.541500	fig|6666666.34159.peg.1803	peg	NODE_8_length_386799_cov_42.541500_100075_101025	100075	1E+05	+	hypothetical protein		

NODE_8_length_386799_cov_42.541500	fig|6666666.34159.peg.1804	peg	NODE_8_length_386799_cov_42.541500_101268_102668	101268	1E+05	+	hypothetical protein	FIG00638284	ff
NODE_8_length_386799_cov_42.541500	fig|6666666.34159.peg.1805	peg	NODE_8_length_386799_cov_42.541500_103724_102768	103724	1E+05	-	hypothetical protein		
NODE_8_length_386799_cov_42.541500	fig|6666666.34159.peg.1806	peg	NODE_8_length_386799_cov_42.541500_104005_103874	104005	1E+05	-	hypothetical protein		
NODE_8_length_386799_cov_42.541500	fig|6666666.34159.peg.1807	peg	NODE_8_length_386799_cov_42.541500_104286_104143	104286	1E+05	-	hypothetical protein		
NODE_8_length_386799_cov_42.541500	fig|6666666.34159.peg.1808	peg	NODE_8_length_386799_cov_42.541500_104399_104836	104399	1E+05	+	Transamidase GatB domain protein		
NODE_8_length_386799_cov_42.541500	fig|6666666.34159.peg.1809	peg	NODE_8_length_386799_cov_42.541500_106337_105216	106337	1E+05	-	ABC-type multidrug transport system, permease component	FIG00001785	ff
NODE_8_length_386799_cov_42.541500	fig|6666666.34159.peg.1810	peg	NODE_8_length_386799_cov_42.541500_109123_106340	109123	1E+05	-	ABC-type multidrug transport system, permease component	FIG00001785	ff
NODE_8_length_386799_cov_42.541500	fig|6666666.34159.peg.1811	peg	NODE_8_length_386799_cov_42.541500_110216_109128	110216	1E+05	-	HlyD family secretion protein	FIG01364763	ff
NODE_8_length_386799_cov_42.541500	fig|6666666.34159.peg.1812	peg	NODE_8_length_386799_cov_42.541500_111281_110268	111281	1E+05	-	RND efflux system, outer membrane lipoprotein CmeC	FIG00006235	idu(2);Multidrug_Resistance_Efflux_Pumps
NODE_8_length_386799_cov_42.541500	fig|6666666.34159.peg.1813	peg	NODE_8_length_386799_cov_42.541500_113282_112728	113282	1E+05	-	Oxidoreductase (putative)		
NODE_8_length_386799_cov_42.541500	fig|6666666.34159.peg.1814	peg	NODE_8_length_386799_cov_42.541500_114356_113304	114356	1E+05	-	Alcohol dehydrogenase (EC 1.1.1.1)	FIG01007502	idu(3);Fermentations:_Mixed_acid idu(3);Butanol_Biosynthesis
NODE_8_length_386799_cov_42.541500	fig|6666666.34159.peg.1815	peg	NODE_8_length_386799_cov_42.541500_115588_114404	115588	1E+05	-	4-carboxymuconolactone decarboxylase (EC 4.1.1.44)	FIG01304850	isu;Protocatechuate_branch_of_beta-ketoadipate_pathway isu;Pyrene_degradation
NODE_8_length_386799_cov_42.541500	fig|6666666.34159.peg.1816	peg	NODE_8_length_386799_cov_42.541500_116589_115606	116589	1E+05	-	Aldo-keto reductase	FIG00639501	ff
NODE_8_length_386799_cov_42.541500	fig|6666666.34159.peg.1817	peg	NODE_8_length_386799_cov_42.541500_116950_116813	116950	1E+05	-	hypothetical protein		
NODE_8_length_386799_cov_42.541500	fig|6666666.34159.peg.1818	peg	NODE_8_length_386799_cov_42.541500_117147_117010	117147	1E+05	-	hypothetical protein		
NODE_8_length_386799_cov_42.541500	fig|6666666.34159.peg.1819	peg	NODE_8_length_386799_cov_42.541500_117344_117195	117344	1E+05	-	hypothetical protein		
NODE_8_length_386799_cov_42.541500	fig|6666666.34159.peg.1820	peg	NODE_8_length_386799_cov_42.541500_117506_117354	117506	1E+05	-	hypothetical protein		
NODE_8_length_386799_cov_42.541500	fig|6666666.34159.peg.1821	peg	NODE_8_length_386799_cov_42.541500_118432_117809	118432	1E+05	-	Methyltransferase		
NODE_8_length_386799_cov_42.541500	fig|6666666.34159.peg.1822	peg	NODE_8_length_386799_cov_42.541500_118556_118690	118556	1E+05	+	hypothetical protein		
NODE_8_length_386799_cov_42.541500	fig|6666666.34159.peg.1823	peg	NODE_8_length_386799_cov_42.541500_118731_119180	118731	1E+05	+	GTP cyclohydrolase II		
NODE_8_length_386799_cov_42.541500	fig|6666666.34159.peg.1824	peg	NODE_8_length_386799_cov_42.541500_120450_119443	120450	1E+05	-	predicted LicA		
NODE_8_length_386799_cov_42.541500	fig|6666666.34159.peg.1825	peg	NODE_8_length_386799_cov_42.541500_120584_120721	120584	1E+05	+	hypothetical protein		
NODE_8_length_386799_cov_42.541500	fig|6666666.34159.rna.27	rna	NODE_8_length_386799_cov_42.541500_120884_120803	120884	1E+05	-	tRNA-Tyr-GTA		
NODE_8_length_386799_cov_42.541500	fig|6666666.34159.rna.28	rna	NODE_8_length_386799_cov_42.541500_120965_120893	120965	1E+05	-	tRNA-Thr-TGT		
NODE_8_length_386799_cov_42.541500	fig|6666666.34159.peg.1826	peg	NODE_8_length_386799_cov_42.541500_122112_121060	122112	1E+05	-	hypothetical protein	FIG00638284	ff
NODE_8_length_386799_cov_42.541500	fig|6666666.34159.peg.1827	peg	NODE_8_length_386799_cov_42.541500_124292_122109	124292	1E+05	-	RecD-like DNA helicase YrrC	FIG00059211	isu;DNA_repair,_bacterial_RecBCD_pathway
NODE_8_length_386799_cov_42.541500	fig|6666666.34159.peg.1828	peg	NODE_8_length_386799_cov_42.541500_125425_124463	125425	1E+05	-	tolA protein		
NODE_8_length_386799_cov_42.541500	fig|6666666.34159.peg.1829	peg	NODE_8_length_386799_cov_42.541500_125491_125604	125491	1E+05	+	hypothetical protein		
NODE_8_length_386799_cov_42.541500	fig|6666666.34159.peg.1830	peg	NODE_8_length_386799_cov_42.541500_126827_125601	126827	1E+05	-	Bicyclomycin resistance protein		
NODE_8_length_386799_cov_42.541500	fig|6666666.34159.peg.1831	peg	NODE_8_length_386799_cov_42.541500_126932_127837	126932	1E+05	+	transcriptional regulator		
NODE_8_length_386799_cov_42.541500	fig|6666666.34159.peg.1832	peg	NODE_8_length_386799_cov_42.541500_127929_128996	127929	1E+05	+	hypothetical protein		
NODE_8_length_386799_cov_42.541500	fig|6666666.34159.peg.1833	peg	NODE_8_length_386799_cov_42.541500_128993_129412	128993	1E+05	+	Mn-dependent transcriptional regulator MntR		
NODE_8_length_386799_cov_42.541500	fig|6666666.34159.peg.1834	peg	NODE_8_length_386799_cov_42.541500_130508_129447	130508	1E+05	-	hypothetical protein	FIG00638284	ff
NODE_8_length_386799_cov_42.541500	fig|6666666.34159.peg.1835	peg	NODE_8_length_386799_cov_42.541500_131317_130610	131317	1E+05	-	Lipoprotein releasing system ATP-binding protein LolD	FIG00000771	isu;Lipoprotein_sorting_system
NODE_8_length_386799_cov_42.541500	fig|6666666.34159.peg.1836	peg	NODE_8_length_386799_cov_42.541500_133489_131330	133489	1E+05	-	Lipoprotein releasing system transmembrane protein LolE	FIG00023187	icw(1);Lipoprotein_sorting_system
NODE_8_length_386799_cov_42.541500	fig|6666666.34159.peg.1837	peg	NODE_8_length_386799_cov_42.541500_133730_133575	133730	1E+05	-	LSU ribosomal protein L33p	FIG00000053	ff
NODE_8_length_386799_cov_42.541500	fig|6666666.34159.peg.1838	peg	NODE_8_length_386799_cov_42.541500_134985_133957	134985	1E+05	-	YgjD/Kae1/Qri7 family, required for threonylcarbamoyladenosine (t(6)A) formation in tRNA	FIG00134348	ff
NODE_8_length_386799_cov_42.541500	fig|6666666.34159.peg.1839	peg	NODE_8_length_386799_cov_42.541500_135042_135557	135042	1E+05	+	hypothetical protein		
NODE_8_length_386799_cov_42.541500	fig|6666666.34159.peg.1840	peg	NODE_8_length_386799_cov_42.541500_135570_136172	135570	1E+05	+	FIG00899458: hypothetical protein	FIG00899457	ff
NODE_8_length_386799_cov_42.541500	fig|6666666.34159.peg.1841	peg	NODE_8_length_386799_cov_42.541500_136180_136785	136180	1E+05	+	FIG00899512: hypothetical protein	FIG00899511	ff
NODE_8_length_386799_cov_42.541500	fig|6666666.34159.peg.1842	peg	NODE_8_length_386799_cov_42.541500_136782_137546	136782	1E+05	+	tRNA pseudouridine synthase A (EC 4.2.1.70)	FIG00000093	idu(1);tRNA_processing idu(1);tRNA_modification_Bacteria [illegible]
NODE_8_length_386799_cov_42.541500	fig|6666666.34159.peg.1843	peg	NODE_8_length_386799_cov_42.541500_138749_137514	138749	1E+05	-	FIG00899465: hypothetical protein	FIG00899463	ff
NODE_8_length_386799_cov_42.541500	fig|6666666.34159.peg.1844	peg	NODE_8_length_386799_cov_42.541500_139153_138749	139153	1E+05	-	FIG00899448: hypothetical protein	FIG00899446	ff
NODE_8_length_386799_cov_42.541500	fig|6666666.34159.peg.1845	peg	NODE_8_length_386799_cov_42.541500_139387_140301	139387	1E+05	+	Ribonuclease HIII (EC 3.1.26.4)	FIG00001474	isu;Ribonucleases_in_Bacillus isu;Ribonuclease_H
NODE_8_length_386799_cov_42.541500	fig|6666666.34159.peg.1846	peg	NODE_8_length_386799_cov_42.541500_140349_140789	140349	1E+05	+	unknown protein	FIG00764722	ff
NODE_8_length_386799_cov_42.541500	fig|6666666.34159.peg.1847	peg	NODE_8_length_386799_cov_42.541500_140805_142451	140805	1E+05	+	DNA repair protein RecN	FIG00000317	isu;DNA_repair,_bacterial
NODE_8_length_386799_cov_42.541500	fig|6666666.34159.peg.1848	peg	NODE_8_length_386799_cov_42.541500_143992_142448	143992	1E+05	-	hypothetical protein		
NODE_8_length_386799_cov_42.541500	fig|6666666.34159.peg.1849	peg	NODE_8_length_386799_cov_42.541500_144232_144486	144232	1E+05	+	putative hydrolase		
NODE_8_length_386799_cov_42.541500	fig|6666666.34159.peg.1850	peg	NODE_8_length_386799_cov_42.541500_145481_145182	145481	1E+05	-	hypothetical protein		
NODE_8_length_386799_cov_42.541500	fig|6666666.34159.peg.1851	peg	NODE_8_length_386799_cov_42.541500_145675_145562	145675	1E+05	-	hypothetical protein		
NODE_8_length_386799_cov_42.541500	fig|6666666.34159.peg.1852	peg	NODE_8_length_386799_cov_42.541500_145956_145834	145956	1E+05	-	hypothetical protein		
NODE_8_length_386799_cov_42.541500	fig|6666666.34159.peg.1853	peg	NODE_8_length_386799_cov_42.541500_146014_146895	146014	1E+05	+	hypothetical protein		
NODE_8_length_386799_cov_42.541500	fig|6666666.34159.peg.1854	peg	NODE_8_length_386799_cov_42.541500_147595_146900	147595	1E+05	-	3-oxoacyl-[acyl-carrier protein] reductase (EC 1.1.1.100)	FIG00621114	idu(12);CBSS-246196.1.peg.364 idu(12);Fatty_Acid_Biosynthesis_FASII
NODE_8_length_386799_cov_42.541500	fig|6666666.34159.peg.1855	peg	NODE_8_length_386799_cov_42.541500_148563_147622	148563	1E+05	-	Voltage-gated potassium channel subunit beta-1 (K(+) channel subunit beta-1) (Kv-beta-1)	FIG01758522	ff
NODE_8_length_386799_cov_42.541500	fig|6666666.34159.peg.1856	peg	NODE_8_length_386799_cov_42.541500_148720_149934	148720	1E+05	+	Heavy metal RND efflux outer membrane protein, CzcC family		idu(1);Cobalt-zinc-cadmium_resistance
NODE_8_length_386799_cov_42.541500	fig|6666666.34159.peg.1857	peg	NODE_8_length_386799_cov_42.541500_149913_151163	149913	2E+05	+	Cobalt/zinc/cadmium efflux RND transporter, membrane fusion protein, CzcB family		icw(1);Cobalt-zinc-cadmium_resistance
NODE_8_length_386799_cov_42.541500	fig|6666666.34159.peg.1858	peg	NODE_8_length_386799_cov_42.541500_151182_154391	151182	2E+05	+	Cobalt-zinc-cadmium resistance protein CzcA; Cation efflux system protein CusA	FIG00008299	icw(2);Cobalt-zinc-cadmium_resistance icw(2);Cobalt-zinc-cadmium_resistance
NODE_8_length_386799_cov_42.541500	fig|6666666.34159.peg.1859	peg	NODE_8_length_386799_cov_42.541500_154552_155088	154552	2E+05	+	NTP pyrophosphohydrolase including oxidative damage repair enzyme		
NODE_8_length_386799_cov_42.541500	fig|6666666.34159.peg.1860	peg	NODE_8_length_386799_cov_42.541500_155618_155478	155618	2E+05	-	hypothetical protein		

NODE_8_length_386799_cov_42.541500	fig|6666666.34159.peg.1861	peg	NODE_8_length_386799_cov_42.541500_155702_155818	155702	2E+05	+	hypothetical protein		
NODE_8_length_386799_cov_42.541500	fig|6666666.34159.peg.1862	peg	NODE_8_length_386799_cov_42.541500_156119_157207	156119	2E+05	+	hypothetical protein		
NODE_8_length_386799_cov_42.541500	fig|6666666.34159.peg.1863	peg	NODE_8_length_386799_cov_42.541500_157218_157907	157218	2E+05	+	hypothetical protein		
NODE_8_length_386799_cov_42.541500	fig|6666666.34159.peg.1864	peg	NODE_8_length_386799_cov_42.541500_158032_158349	158032	2E+05	+	hypothetical protein		
NODE_8_length_386799_cov_42.541500	fig|6666666.34159.peg.1865	peg	NODE_8_length_386799_cov_42.541500_158360_158908	158360	2E+05	+	hypothetical protein		
NODE_8_length_386799_cov_42.541500	fig|6666666.34159.peg.1866	peg	NODE_8_length_386799_cov_42.541500_159963_159046	159963	2E+05	-	Cell division inhibitor	FIG00002048	isu;CBSS-83333.1.peg.946 isu;Persister_Cells
NODE_8_length_386799_cov_42.541500	fig|6666666.34159.peg.1867	peg	NODE_8_length_386799_cov_42.541500_160567_159986	160567	2E+05	-	PPO candidate 1	FIG01307617	ff
NODE_8_length_386799_cov_42.541500	fig|6666666.34159.peg.1868	peg	NODE_8_length_386799_cov_42.541500_160808_161446	160808	2E+05	+	hypothetical protein		
NODE_8_length_386799_cov_42.541500	fig|6666666.34159.peg.1869	peg	NODE_8_length_386799_cov_42.541500_162916_161492	162916	2E+05	-	Deoxyribodipyrimidine photolyase (EC 4.1.99.3)	FIG00000633	isu;DNA_repair,_bacterial_photolyase
NODE_8_length_386799_cov_42.541500	fig|6666666.34159.peg.1870	peg	NODE_8_length_386799_cov_42.541500_164776_162926	164776	2E+05	-	hypothetical protein	FIG00638284	ff
NODE_8_length_386799_cov_42.541500	fig|6666666.34159.peg.1871	peg	NODE_8_length_386799_cov_42.541500_164963_165859	164963	2E+05	+	Methionine aminopeptidase (EC 3.4.11.18)	FIG00000036	isu;CBSS-312309.3.peg.1965 isu;Translation_termination_factors_bacterial
NODE_8_length_386799_cov_42.541500	fig|6666666.34159.peg.1872	peg	NODE_8_length_386799_cov_42.541500_166389_165856	166389	2E+05	-	hypothetical protein		
NODE_8_length_386799_cov_42.541500	fig|6666666.34159.peg.1873	peg	NODE_8_length_386799_cov_42.541500_167828_166425	167828	2E+05	-	Soluble pyridine nucleotide transhydrogenase (EC 1.6.1.1)	FIG00001482	ff
NODE_8_length_386799_cov_42.541500	fig|6666666.34159.peg.1874	peg	NODE_8_length_386799_cov_42.541500_168016_168447	168016	2E+05	+	hypothetical protein		
NODE_8_length_386799_cov_42.541500	fig|6666666.34159.peg.1875	peg	NODE_8_length_386799_cov_42.541500_168758_168474	168758	2E+05	-	LSU ribosomal protein L28p	FIG00000266	ff
NODE_8_length_386799_cov_42.541500	fig|6666666.34159.peg.1876	peg	NODE_8_length_386799_cov_42.541500_171095_169011	171095	2E+05	-	hypothetical protein	FIG00638284	ff
NODE_8_length_386799_cov_42.541500	fig|6666666.34159.peg.1877	peg	NODE_8_length_386799_cov_42.541500_173728_171686	173728	2E+05	-	Methionyl-tRNA synthetase (EC 6.1.1.10)	FIG01265301	isu;tRNA_aminoacylation,_Met
NODE_8_length_386799_cov_42.541500	fig|6666666.34159.peg.1878	peg	NODE_8_length_386799_cov_42.541500_174396_173731	174396	2E+05	-	unknown protein	FIG00764722	ff
NODE_8_length_386799_cov_42.541500	fig|6666666.34159.peg.1879	peg	NODE_8_length_386799_cov_42.541500_174695_174408	174695	2E+05	-	FIG00899503: hypothetical protein	FIG00899502	ff
NODE_8_length_386799_cov_42.541500	fig|6666666.34159.peg.1880	peg	NODE_8_length_386799_cov_42.541500_175375_174752	175375	2E+05	-	Guanylate kinase (EC 2.7.4.8)	FIG00000131	isu;Purine_conversions
NODE_8_length_386799_cov_42.541500	fig|6666666.34159.peg.1881	peg	NODE_8_length_386799_cov_42.541500_176249_175365	176249	2E+05	-	hypothetical protein	FIG00638284	ff
NODE_8_length_386799_cov_42.541500	fig|6666666.34159.peg.1882	peg	NODE_8_length_386799_cov_42.541500_176421_177407	176421	2E+05	+	Cystathionine beta-synthase (EC 4.2.1.22)		icw(1);Glycine_and_Serine_Utilization icw(1);Methionine_Degradation [illegible]
NODE_8_length_386799_cov_42.541500	fig|6666666.34159.peg.1883	peg	NODE_8_length_386799_cov_42.541500_177404_178549	177404	2E+05	+	Cystathionine gamma-lyase (EC 4.4.1.1)	FIG00001346	isu;Glycine_and_Serine_Utilization isu;Methionine_Degradation [illegible]
NODE_8_length_386799_cov_42.541500	fig|6666666.34159.peg.1884	peg	NODE_8_length_386799_cov_42.541500_180151_178550	180151	2E+05	-	hypothetical protein	FIG00638284	ff
NODE_8_length_386799_cov_42.541500	fig|6666666.34159.peg.1885	peg	NODE_8_length_386799_cov_42.541500_181632_180478	181632	2E+05	-	hypothetical protein		
NODE_8_length_386799_cov_42.541500	fig|6666666.34159.peg.1886	peg	NODE_8_length_386799_cov_42.541500_181772_182074	181772	2E+05	+	hypothetical protein		
NODE_8_length_386799_cov_42.541500	fig|6666666.34159.peg.1887	peg	NODE_8_length_386799_cov_42.541500_182532_182113	182532	2E+05	-	NADPH:quinone oxidoreductase		
NODE_8_length_386799_cov_42.541500	fig|6666666.34159.peg.1888	peg	NODE_8_length_386799_cov_42.541500_183814_182780	183814	2E+05	-	Mg/Co/Ni transporter MgtE / CBS domain		idu(2);Magnesium_transport
NODE_8_length_386799_cov_42.541500	fig|6666666.34159.peg.1889	peg	NODE_8_length_386799_cov_42.541500_184474_183848	184474	2E+05	-	Ribonuclease HII (EC 3.1.26.4)	FIG00000126	isu;Ribonucleases_in_Bacillus isu;DNA_replication,_archaeal [illegible]
NODE_8_length_386799_cov_42.541500	fig|6666666.34159.peg.1890	peg	NODE_8_length_386799_cov_42.541500_184958_184524	184958	2E+05	-	LSU ribosomal protein L19p	FIG00000214	ff
NODE_8_length_386799_cov_42.541500	fig|6666666.34159.peg.1891	peg	NODE_8_length_386799_cov_42.541500_185666_184983	185666	2E+05	-	tRNA (Guanine37-N1) -methyltransferase (EC 2.1.1.31)	FIG00000230	isu;tRNA_modification_Bacteria isu;RNA_methylation
NODE_8_length_386799_cov_42.541500	fig|6666666.34159.peg.1892	peg	NODE_8_length_386799_cov_42.541500_186001_185669	186001	2E+05	-	SSU ribosomal protein S16p	FIG00000193	isu;KH_domain_RNA_binding_protein_YlqC
NODE_8_length_386799_cov_42.541500	fig|6666666.34159.peg.1893	peg	NODE_8_length_386799_cov_42.541500_187320_185992	187320	2E+05	-	Signal recognition particle, subunit Ffh SRP54 (TC 3.A.5.1.1)	FIG00000113	isu;Bacterial_signal_recognition_particle_(SRP)
NODE_8_length_386799_cov_42.541500	fig|6666666.34159.peg.1894	peg	NODE_8_length_386799_cov_42.541500_188232_187375	188232	2E+05	-	Methylase of polypeptide chain release factors	FIG00003217	ff
NODE_8_length_386799_cov_42.541500	fig|6666666.34159.peg.1895	peg	NODE_8_length_386799_cov_42.541500_189304_188240	189304	2E+05	-	Peptide chain release factor 1	FIG00000188	isu;Translation_termination_factors_bacterial
NODE_8_length_386799_cov_42.541500	fig|6666666.34159.peg.1896	peg	NODE_8_length_386799_cov_42.541500_189654_189367	189654	2E+05	-	LSU ribosomal protein L31p	FIG00000092	ff
NODE_8_length_386799_cov_42.541500	fig|6666666.34159.peg.1897	peg	NODE_8_length_386799_cov_42.541500_190023_192104	190023	2E+05	+	hypothetical protein		
NODE_8_length_386799_cov_42.541500	fig|6666666.34159.peg.1898	peg	NODE_8_length_386799_cov_42.541500_192122_193057	192122	2E+05	+	hypothetical protein	FIG00638284	ff
NODE_8_length_386799_cov_42.541500	fig|6666666.34159.peg.1899	peg	NODE_8_length_386799_cov_42.541500_193441_195270	193441	2E+05	+	hypothetical protein	FIG00638284	ff
NODE_8_length_386799_cov_42.541500	fig|6666666.34159.peg.1900	peg	NODE_8_length_386799_cov_42.541500_195442_196032	195442	2E+05	+	hypothetical protein		
NODE_8_length_386799_cov_42.541500	fig|6666666.34159.peg.1901	peg	NODE_8_length_386799_cov_42.541500_197039_196095	197039	2E+05	-	MORN motif		
NODE_8_length_386799_cov_42.541500	fig|6666666.34159.peg.1902	peg	NODE_8_length_386799_cov_42.541500_198277_197234	198277	2E+05	-	hypothetical protein	FIG00638284	ff
NODE_8_length_386799_cov_42.541500	fig|6666666.34159.peg.1903	peg	NODE_8_length_386799_cov_42.541500_198575_198384	198575	2E+05	-	hypothetical protein		
NODE_8_length_386799_cov_42.541500	fig|6666666.34159.peg.1904	peg	NODE_8_length_386799_cov_42.541500_200084_198636	200084	2E+05	-	truncated monoxygenase		
NODE_8_length_386799_cov_42.541500	fig|6666666.34159.peg.1905	peg	NODE_8_length_386799_cov_42.541500_200305_200916	200305	2E+05	+	BclA protein		
NODE_8_length_386799_cov_42.541500	fig|6666666.34159.peg.1906	peg	NODE_8_length_386799_cov_42.541500_201131_201256	201131	2E+05	+	hypothetical protein		
NODE_8_length_386799_cov_42.541500	fig|6666666.34159.peg.1907	peg	NODE_8_length_386799_cov_42.541500_201271_201525	201271	2E+05	+	hypothetical protein		
NODE_8_length_386799_cov_42.541500	fig|6666666.34159.peg.1908	peg	NODE_8_length_386799_cov_42.541500_201649_203448	201649	2E+05	+	COG0488: ATPase components of ABC transporters with duplicated ATPase domains	FIG00005836	ff
NODE_8_length_386799_cov_42.541500	fig|6666666.34159.peg.1909	peg	NODE_8_length_386799_cov_42.541500_203635_204408	203635	2E+05	+	Protein serine/threonine phosphatase PrpC, regulation of stationary phase	FIG00134722	ff
NODE_8_length_386799_cov_42.541500	fig|6666666.34159.peg.1910	peg	NODE_8_length_386799_cov_42.541500_204383_205522	204383	2E+05	+	Cysteine desulfurase (EC 2.8.1.7)	FIG00000001	idu(1);Iron-sulfur_cluster_assembly idu(1);CBSS-84588.1.peg.1247 idu(1);CBSS-393130.3.peg.794 [illegible]
NODE_8_length_386799_cov_42.541500	fig|6666666.34159.peg.1911	peg	NODE_8_length_386799_cov_42.541500_205602_207098	205602	2E+05	+	Glycogen synthase, ADP-glucose transglucosylase (EC 2.4.1.21)	FIG00134135	isu;Glycogen_metabolism isu;Glycogen_metabolism_cluster
NODE_8_length_386799_cov_42.541500	fig|6666666.34159.peg.1912	peg	NODE_8_length_386799_cov_42.541500_207663_207538	207663	2E+05	-	hypothetical protein		
NODE_8_length_386799_cov_42.541500	fig|6666666.34159.peg.1913	peg	NODE_8_length_386799_cov_42.541500_207883_207767	207883	2E+05	-	hypothetical protein		
NODE_8_length_386799_cov_42.541500	fig|6666666.34159.peg.1914	peg	NODE_8_length_386799_cov_42.541500_207937_208548	207937	2E+05	+	CDP-diacylglycerol--glycerol-3-phosphate 3-phosphatidyltransferase (EC 2.7.8.5)	FIG00001550	ff
NODE_8_length_386799_cov_42.541500	fig|6666666.34159.peg.1915	peg	NODE_8_length_386799_cov_42.541500_208556_209515	208556	2E+05	+	Ribokinase (EC 2.7.1.15)		isu;D-ribose_utilization isu;Deoxyribose_and_Deoxynucleoside_Catabolis[illegible]
NODE_8_length_386799_cov_42.541500	fig|6666666.34159.peg.1916	peg	NODE_8_length_386799_cov_42.541500_210869_209517	210869	2E+05	-	sodium/alanine symporter family protein	FIG01316725	ff
NODE_8_length_386799_cov_42.541500	fig|6666666.34159.peg.1917	peg	NODE_8_length_386799_cov_42.541500_211428_210949	211428	2E+05	-	hypothetical protein	FIG00638284	ff
NODE_8_length_386799_cov_42.541500	fig|6666666.34159.peg.1918	peg	NODE_8_length_386799_cov_42.541500_211553_212089	211553	2E+05	+	hypothetical protein	FIG00638284	ff
NODE_8_length_386799_cov_42.541500	fig|6666666.34159.peg.1919	peg	NODE_8_length_386799_cov_42.541500_214159_212126	214159	2E+05	-	ATP-dependent DNA helicase UvrD/PcrA	FIG00000372	idu(1);CBSS-393121.3.peg.1913 idu(1);DNA_repair,_bacterial_UvrD_and_related [illegible]

NODE_8_length_386799_cov_42.541500	fig|6666666.34159.peg.1920	peg	NODE_8_length_386799_cov_42.541500_214472_214212	214472	2E+05	-	hypothetical protein		
NODE_8_length_386799_cov_42.541500	fig|6666666.34159.peg.1921	peg	NODE_8_length_386799_cov_42.541500_214715_214578	214715	2E+05	-	LSU ribosomal protein L34p	FIG00000471	isu;RNA_modification_cluster
NODE_8_length_386799_cov_42.541500	fig|6666666.34159.peg.1922	peg	NODE_8_length_386799_cov_42.541500_214957_216654	214957	2E+05	+	hypothetical protein		
NODE_8_length_386799_cov_42.541500	fig|6666666.34159.peg.1923	peg	NODE_8_length_386799_cov_42.541500_216766_216623	216766	2E+05	-	hypothetical protein		
NODE_8_length_386799_cov_42.541500	fig|6666666.34159.peg.1924	peg	NODE_8_length_386799_cov_42.541500_216825_218705	216825	2E+05	+	hypothetical protein	FIG00638284	ff
NODE_8_length_386799_cov_42.541500	fig|6666666.34159.peg.1925	peg	NODE_8_length_386799_cov_42.541500_218939_219076	218939	2E+05	+	LSU ribosomal protein L36p	FIG00000229	ff
NODE_8_length_386799_cov_42.541500	fig|6666666.34159.peg.1926	peg	NODE_8_length_386799_cov_42.541500_219089_219394	219089	2E+05	+	SSU ribosomal protein S14p (S29e)	FIG01955777	ff
NODE_8_length_386799_cov_42.541500	fig|6666666.34159.peg.1927	peg	NODE_8_length_386799_cov_42.541500_219878_219453	219878	2E+05	-	hypothetical protein		
NODE_8_length_386799_cov_42.541500	fig|6666666.34159.peg.1928	peg	NODE_8_length_386799_cov_42.541500_220090_220560	220090	2E+05	+	hypothetical protein	FIG00638284	ff
NODE_8_length_386799_cov_42.541500	fig|6666666.34159.peg.1929	peg	NODE_8_length_386799_cov_42.541500_220570_221094	220570	2E+05	+	hypothetical protein		
NODE_8_length_386799_cov_42.541500	fig|6666666.34159.peg.1930	peg	NODE_8_length_386799_cov_42.541500_221544_221125	221544	2E+05	-	hypothetical protein		
NODE_8_length_386799_cov_42.541500	fig|6666666.34159.peg.1931	peg	NODE_8_length_386799_cov_42.541500_221727_223208	221727	2E+05	+	hypothetical protein		
NODE_8_length_386799_cov_42.541500	fig|6666666.34159.peg.1932	peg	NODE_8_length_386799_cov_42.541500_224372_223776	224372	2E+05	-	hypothetical protein		
NODE_8_length_386799_cov_42.541500	fig|6666666.34159.peg.1933	peg	NODE_8_length_386799_cov_42.541500_224590_224988	224590	2E+05	+	hypothetical protein		
NODE_8_length_386799_cov_42.541500	fig|6666666.34159.peg.1934	peg	NODE_8_length_386799_cov_42.541500_225119_225439	225119	2E+05	+	hypothetical protein		
NODE_8_length_386799_cov_42.541500	fig|6666666.34159.peg.1935	peg	NODE_8_length_386799_cov_42.541500_225463_226044	225463	2E+05	+	Septum formation protein Maf	FIG00021845	ff
NODE_8_length_386799_cov_42.541500	fig|6666666.34159.peg.1936	peg	NODE_8_length_386799_cov_42.541500_226041_227729	226041	2E+05	+	hypothetical protein	FIG00638284	ff
NODE_8_length_386799_cov_42.541500	fig|6666666.34159.peg.1937	peg	NODE_8_length_386799_cov_42.541500_227840_229948	227840	2E+05	+	hypothetical protein	FIG00638284	ff
NODE_8_length_386799_cov_42.541500	fig|6666666.34159.peg.1938	peg	NODE_8_length_386799_cov_42.541500_229954_230475	229954	2E+05	+	hypothetical protein		
NODE_8_length_386799_cov_42.541500	fig|6666666.34159.peg.1939	peg	NODE_8_length_386799_cov_42.541500_230528_231952	230528	2E+05	+	UDP-glucose dehydrogenase (EC 1.1.1.22)	FIG00000532	idu(1);Inteins idu(1);Lipid_A-Ara4N_pathway_(_Polymyxin_resistance_)
NODE_8_length_386799_cov_42.541500	fig|6666666.34159.peg.1940	peg	NODE_8_length_386799_cov_42.541500_231933_232850	231933	2E+05	+	Octaprenyl-diphosphate synthase (EC 2.5.1.-) / Dimethylallyltransferase (EC 2.5.1.1) / Geranyltranstransferase (farnesyldiphosphate synthase) (EC 2.5.1.10) / Geranylgeranyl	FIG00000019	idu(1);Isoprenoid_Biosynthesis
NODE_8_length_386799_cov_42.541500	fig|6666666.34159.peg.1941	peg	NODE_8_length_386799_cov_42.541500_232936_233601	232936	2E+05	+	Undecaprenyl-phosphate galactosephosphotransferase (EC 2.7.8.6)	FIG00002968	isu;Exopolysaccharide_Biosynthesis isu;CBSS-258594.1.peg.3339
NODE_8_length_386799_cov_42.541500	fig|6666666.34159.peg.1942	peg	NODE_8_length_386799_cov_42.541500_233604_234137	233604	2E+05	+	hypothetical protein		
NODE_8_length_386799_cov_42.541500	fig|6666666.34159.peg.1943	peg	NODE_8_length_386799_cov_42.541500_234764_234186	234764	2E+05	-	hypothetical protein		
NODE_8_length_386799_cov_42.541500	fig|6666666.34159.peg.1944	peg	NODE_8_length_386799_cov_42.541500_235306_234797	235306	2E+05	-	hypothetical protein		
NODE_8_length_386799_cov_42.541500	fig|6666666.34159.peg.1945	peg	NODE_8_length_386799_cov_42.541500_235541_237730	235541	2E+05	+	hypothetical protein	FIG00638284	ff
NODE_8_length_386799_cov_42.541500	fig|6666666.34159.peg.1946	peg	NODE_8_length_386799_cov_42.541500_237723_238526	237723	2E+05	+	Aminodeoxychorismate lyase (EC 4.1.3.38)	FIG00000708	isu;Chorismate:_Intermediate_for_synthesis_of_Tryptophan,_PAPA_antibiotics,_PABA,_3-hydroxyanthranilate_and_more
NODE_8_length_386799_cov_42.541500	fig|6666666.34159.peg.1947	peg	NODE_8_length_386799_cov_42.541500_238892_238509	238892	2E+05	-	hypothetical protein		
NODE_8_length_386799_cov_42.541500	fig|6666666.34159.peg.1948	peg	NODE_8_length_386799_cov_42.541500_238956_239744	238956	2E+05	+	5-nucleotidase SurE (EC 3.1.3.5)	FIG00000480	isu;Housecleaning_nucleoside_triphosphate_pyrophosphatases isu;Stationary_phase_repair_cluster
NODE_8_length_386799_cov_42.541500	fig|6666666.34159.peg.1949	peg	NODE_8_length_386799_cov_42.541500_239817_239704	239817	2E+05	-	hypothetical protein		
NODE_8_length_386799_cov_42.541500	fig|6666666.34159.rna.29	rna	NODE_8_length_386799_cov_42.541500_239987_239914	239987	2E+05	-	tRNA-Asp-GTC		
NODE_8_length_386799_cov_42.541500	fig|6666666.34159.rna.30	rna	NODE_8_length_386799_cov_42.541500_240077_240005	240077	2E+05	-	tRNA-Val-TAC		
NODE_8_length_386799_cov_42.541500	fig|6666666.34159.peg.1950	peg	NODE_8_length_386799_cov_42.541500_240296_241246	240296	2E+05	+	Uncharacterized membrane protein Bsu2508 (YqfU)	FIG01312674	ff
NODE_8_length_386799_cov_42.541500	fig|6666666.34159.peg.1951	peg	NODE_8_length_386799_cov_42.541500_241236_242174	241236	2E+05	+	Epoxyqueuosine (oQ) reductase QueG		isu;Queuosine-Archaeosine_Biosynthesis
NODE_8_length_386799_cov_42.541500	fig|6666666.34159.peg.1952	peg	NODE_8_length_386799_cov_42.541500_242167_242838	242167	2E+05	+	hypothetical protein	FIG00638284	ff
NODE_8_length_386799_cov_42.541500	fig|6666666.34159.peg.1953	peg	NODE_8_length_386799_cov_42.541500_243175_242891	243175	2E+05	-	hypothetical protein		
NODE_8_length_386799_cov_42.541500	fig|6666666.34159.peg.1954	peg	NODE_8_length_386799_cov_42.541500_243996_243190	243996	2E+05	-	hypothetical protein		
NODE_8_length_386799_cov_42.541500	fig|6666666.34159.peg.1955	peg	NODE_8_length_386799_cov_42.541500_244692_244042	244692	2E+05	-	hypothetical protein		
NODE_8_length_386799_cov_42.541500	fig|6666666.34159.peg.1956	peg	NODE_8_length_386799_cov_42.541500_245550_244762	245550	2E+05	-	hypothetical protein		
NODE_8_length_386799_cov_42.541500	fig|6666666.34159.peg.1957	peg	NODE_8_length_386799_cov_42.541500_247107_245743	247107	2E+05	-	Biotin carboxylase of acetyl-CoA carboxylase (EC 6.3.4.14)	FIG00000420	icw(1);Fatty_Acid_Biosynthesis_FASII
NODE_8_length_386799_cov_42.541500	fig|6666666.34159.peg.1958	peg	NODE_8_length_386799_cov_42.541500_247623_247123	247623	2E+05	-	Biotin carboxyl carrier protein of acetyl-CoA carboxylase	FIG00000411	isu;Fatty_Acid_Biosynthesis_FASII
NODE_8_length_386799_cov_42.541500	fig|6666666.34159.peg.1959	peg	NODE_8_length_386799_cov_42.541500_248199_247645	248199	2E+05	-	Translation elongation factor P-related protein	FIG00133360	isu;Translation_elongation_factors_bacterial
NODE_8_length_386799_cov_42.541500	fig|6666666.34159.peg.1960	peg	NODE_8_length_386799_cov_42.541500_248962_248249	248962	2E+05	-	Ribulose-phosphate 3-epimerase (EC 5.1.3.1)	FIG00000047	isu;Riboflavin_synthesis_cluster isu;Calvin-Benson_cycle isu;Pentose_phosphate_pathway
NODE_8_length_386799_cov_42.541500	fig|6666666.34159.peg.1961	peg	NODE_8_length_386799_cov_42.541500_249479_250645	249479	3E+05	+	Branched-chain amino acid transport system carrier protein	FIG00905542	ff
NODE_8_length_386799_cov_42.541500	fig|6666666.34159.peg.1962	peg	NODE_8_length_386799_cov_42.541500_252613_250634	252613	3E+05	-	unknown protein	FIG00764722	ff
NODE_8_length_386799_cov_42.541500	fig|6666666.34159.peg.1963	peg	NODE_8_length_386799_cov_42.541500_253449_252613	253449	3E+05	-	Aspartokinase (EC 2.7.2.4)	FIG00000132	isu;Lysine_Biosynthesis_DAP_Pathway,_GJO_scratch isu;CBSS-216591.1.peg.168 isu;Threonine_and_Homoserine_Biosynthesis
NODE_8_length_386799_cov_42.541500	fig|6666666.34159.peg.1964	peg	NODE_8_length_386799_cov_42.541500_255967_253682	255967	3E+05	-	hypothetical protein	FIG00638284	ff
NODE_8_length_386799_cov_42.541500	fig|6666666.34159.peg.1965	peg	NODE_8_length_386799_cov_42.541500_257039_256116	257039	3E+05	-	Riboflavin kinase (EC 2.7.1.26) / FMN adenylyltransferase (EC 2.7.7.2)	FIG00000220	isu;Riboflavin,_FMN_and_FAD_metabolism isu;Riboflavin,_FMN_and_FAD_metabolism isu;riboflavin_to_FAD isu;riboflavin_to_FAD
NODE_8_length_386799_cov_42.541500	fig|6666666.34159.peg.1966	peg	NODE_8_length_386799_cov_42.541500_257752_257036	257752	3E+05	-	tRNA pseudouridine synthase B (EC 4.2.1.70)	FIG01016270	icw(2);CBSS-138119.3.peg.2719 isu;tRNA_processing isu;tRNA_modification_Bacteria
NODE_8_length_386799_cov_42.541500	fig|6666666.34159.peg.1967	peg	NODE_8_length_386799_cov_42.541500_258128_257730	258128	3E+05	-	Ribosome-binding factor A	FIG00000164	icw(1);CBSS-138119.3.peg.2719 icw(2);NusA-TFII_Cluster icw(1);Translation_initiation_factors_bacterial
NODE_8_length_386799_cov_42.541500	fig|6666666.34159.peg.1968	peg	NODE_8_length_386799_cov_42.541500_260874_258133	260874	3E+05	-	Translation initiation factor 2	FIG00000102	isu;CBSS-138119.3.peg.2719 isu;Inteins isu;NusA-TFII_Cluster isu;Translation_initiation_factors_bacterial
NODE_8_length_386799_cov_42.541500	fig|6666666.34159.peg.1969	peg	NODE_8_length_386799_cov_42.541500_262153_260879	262153	3E+05	-	Transcription termination protein NusA	FIG00000168	icw(1);NusA-TFII_Cluster isu;Transcription_factors_bacterial
NODE_8_length_386799_cov_42.541500	fig|6666666.34159.peg.1970	peg	NODE_8_length_386799_cov_42.541500_262379_262266	262379	3E+05	-	hypothetical protein		
NODE_8_length_386799_cov_42.541500	fig|6666666.34159.peg.1971	peg	NODE_8_length_386799_cov_42.541500_264223_262451	264223	3E+05	-	SSU ribosomal protein S1p	FIG00000210	isu;Cell_division-ribosomal_stress_proteins_cluster
NODE_8_length_386799_cov_42.541500	fig|6666666.34159.peg.1972	peg	NODE_8_length_386799_cov_42.541500_264723_264421	264723	3E+05	-	hypothetical protein		
NODE_8_length_386799_cov_42.541500	fig|6666666.34159.peg.1973	peg	NODE_8_length_386799_cov_42.541500_265652_264720	265652	3E+05	-	rRNA small subunit methyltransferase H	FIG00000172	isu;16S_rRNA_modification_within_P_site_of_ribosome
NODE_8_length_386799_cov_42.541500	fig|6666666.34159.peg.1974	peg	NODE_8_length_386799_cov_42.541500_265953_266402	265953	3E+05	+	hypothetical protein		
NODE_8_length_386799_cov_42.541500	fig|6666666.34159.peg.1975	peg	NODE_8_length_386799_cov_42.541500_266406_266801	266406	3E+05	+	type III secretion chaperone, putative		
NODE_8_length_386799_cov_42.541500	fig|6666666.34159.peg.1976	peg	NODE_8_length_386799_cov_42.541500_267976_266798	267976	3E+05	-	Bacillosamine/Legionaminic acid biosynthesis aminotransferase PglE; 4-keto-6-deoxy-N-Acetyl-D-hexosaminyl-(Lipid carrier) aminotransferase	FIG00150184	isu;N-linked_Glycosylation_in_Bacteria

NODE_8_length_386799_cov_42.541500	fig|6666666.34159.peg.1977	peg	NODE_8_length_386799_cov_42.541500_268370_270415	268370	3E+05	+	hypothetical protein	FIG00638284	ff
NODE_8_length_386799_cov_42.541500	fig|6666666.34159.peg.1978	peg	NODE_8_length_386799_cov_42.541500_270415_272427	270415	3E+05	+	chemotaxis transducer		
NODE_8_length_386799_cov_42.541500	fig|6666666.34159.peg.1979	peg	NODE_8_length_386799_cov_42.541500_281578_272429	281578	3E+05	-	hypothetical protein	FIG00638284	ff
NODE_8_length_386799_cov_42.541500	fig|6666666.34159.peg.1980	peg	NODE_8_length_386799_cov_42.541500_281838_283868	281838	3E+05	+	Cell division protein FtsI [Peptidoglycan synthetase] (EC 2.4.1.129)	FIG00046929	isu;16S_rRNA_modification_within_P_site_of_ribosome isu;Flagellum_in_Campylobacter
NODE_8_length_386799_cov_42.541500	fig|6666666.34159.peg.1981	peg	NODE_8_length_386799_cov_42.541500_283908_285389	283908	3E+05	+	UDP-N-acetylmuramoylalanyl-D-glutamate--2,6-diaminopimelate ligase (EC 6.3.2.13)	FIG00000243	isu;Methicillin_resistance_in_Staphylococci
NODE_8_length_386799_cov_42.541500	fig|6666666.34159.peg.1982	peg	NODE_8_length_386799_cov_42.541500_285393_286130	285393	3E+05	+	N-acetylmuramoyl-L-alanine amidase (EC 3.5.1.28)	FIG00001385	isu;Murein_Hydrolases isu;Recycling_of_Peptidoglycan_Amino_Acids
NODE_8_length_386799_cov_42.541500	fig|6666666.34159.rna.31	rna	NODE_8_length_386799_cov_42.541500_286205_286132	286205	3E+05	-	tRNA-Arg-CCT		
NODE_8_length_386799_cov_42.541500	fig|6666666.34159.peg.1983	peg	NODE_8_length_386799_cov_42.541500_286440_289064	286440	3E+05	+	hypothetical protein	FIG00638284	ff
NODE_8_length_386799_cov_42.541500	fig|6666666.34159.peg.1984	peg	NODE_8_length_386799_cov_42.541500_289219_290670	289219	3E+05	+	6-phosphogluconate dehydrogenase, decarboxylating (EC 1.1.1.44)	FIG00000405	isu;Pentose_phosphate_pathway isu;D-gluconate_and_ketogluconates_metabolism
NODE_8_length_386799_cov_42.541500	fig|6666666.34159.peg.1985	peg	NODE_8_length_386799_cov_42.541500_290737_293334	290737	3E+05	+	ClpB protein	FIG00025216	isu;Proteolysis_in_bacteria,_ATP-dependent isu;USS-DB-7 isu;Protein_chaperones isu;Type_VI_secretion_systems
NODE_8_length_386799_cov_42.541500	fig|6666666.34159.peg.1986	peg	NODE_8_length_386799_cov_42.541500_295547_293355	295547	3E+05	-	hypothetical protein	FIG00638284	ff
NODE_8_length_386799_cov_42.541500	fig|6666666.34159.peg.1987	peg	NODE_8_length_386799_cov_42.541500_296909_295704	296909	3E+05	-	Serine phosphatase RsbU, regulator of sigma subunit	FIG00017431	isu;SigmaB_stress_responce_regulation
NODE_8_length_386799_cov_42.541500	fig|6666666.34159.peg.1988	peg	NODE_8_length_386799_cov_42.541500_297942_296902	297942	3E+05	-	Chemotaxis response regulator protein-glutamate methylesterase CheB (EC 3.1.1.61)	FIG00017306	ff
NODE_8_length_386799_cov_42.541500	fig|6666666.34159.peg.1989	peg	NODE_8_length_386799_cov_42.541500_298138_299682	298138	3E+05	+	hypothetical protein		
NODE_8_length_386799_cov_42.541500	fig|6666666.34159.peg.1990	peg	NODE_8_length_386799_cov_42.541500_303908_299763	303908	3E+05	-	DNA-directed RNA polymerase beta' subunit (EC 2.7.7.6)	FIG00000242	isu;Mycobacterium_virulence_operon_involved_in_DNA_transcription isu;RNA_polymerase_bacterial
NODE_8_length_386799_cov_42.541500	fig|6666666.34159.peg.1991	peg	NODE_8_length_386799_cov_42.541500_307874_304119	307874	3E+05	-	DNA-directed RNA polymerase beta subunit (EC 2.7.7.6)	FIG00000156	icw(1);Mycobacterium_virulence_operon_involved_in_DNA_transcription icw(1);RNA_polymerase_bacterial
NODE_8_length_386799_cov_42.541500	fig|6666666.34159.peg.1992	peg	NODE_8_length_386799_cov_42.541500_308539_308153	308539	3E+05	-	LSU ribosomal protein L7/L12 (P1/P2)	FIG00000141	icw(1);LSU_ribosomal_proteins_cluster
NODE_8_length_386799_cov_42.541500	fig|6666666.34159.peg.1993	peg	NODE_8_length_386799_cov_42.541500_309099_308560	309099	3E+05	-	LSU ribosomal protein L10p (P0)	FIG00000159	icw(5);LSU_ribosomal_proteins_cluster
NODE_8_length_386799_cov_42.541500	fig|6666666.34159.peg.1994	peg	NODE_8_length_386799_cov_42.541500_309816_309115	309816	3E+05	-	LSU ribosomal protein L1p (L10Ae)	FIG00000264	icw(4);LSU_ribosomal_proteins_cluster
NODE_8_length_386799_cov_42.541500	fig|6666666.34159.peg.1995	peg	NODE_8_length_386799_cov_42.541500_310150_309827	310150	3E+05	-	LSU ribosomal protein L11p (L12e)	FIG00000182	icw(2);LSU_ribosomal_proteins_cluster
NODE_8_length_386799_cov_42.541500	fig|6666666.34159.peg.1996	peg	NODE_8_length_386799_cov_42.541500_310861_310313	310861	3E+05	-	Transcription antitermination protein NusG	FIG00000269	icw(3);LSU_ribosomal_proteins_cluster isu;Transcription_factors_bacterial
NODE_8_length_386799_cov_42.541500	fig|6666666.34159.peg.1997	peg	NODE_8_length_386799_cov_42.541500_311174_310911	311174	3E+05	-	Preprotein translocase subunit SecE (TC 3.A.5.1.1)	FIG00001219	isu;LSU_ribosomal_proteins_cluster
NODE_8_length_386799_cov_42.541500	fig|6666666.34159.rna.32	rna	NODE_8_length_386799_cov_42.541500_311421_311349	311421	3E+05	-	tRNA-Trp-CCA		isu;tRNAs
NODE_8_length_386799_cov_42.541500	fig|6666666.34159.peg.1998	peg	NODE_8_length_386799_cov_42.541500_312679_311492	312679	3E+05	-	Translation elongation factor Tu	FIG00000039	isu;Translation_elongation_factors_bacterial isu;Mycobacterium_virulence_operon_involved_in_protein_synthesis_(SSU_ribosomal_proteins)
NODE_8_length_386799_cov_42.541500	fig|6666666.34159.rna.33	rna	NODE_8_length_386799_cov_42.541500_312802_312731	312802	3E+05	-	tRNA-Thr-GGT		
NODE_8_length_386799_cov_42.541500	fig|6666666.34159.peg.1999	peg	NODE_8_length_386799_cov_42.541500_313120_312902	313120	3E+05	-	Translation initiation factor 1	FIG00000143	isu;Translation_initiation_factors_bacterial
NODE_8_length_386799_cov_42.541500	fig|6666666.34159.peg.2000	peg	NODE_8_length_386799_cov_42.541500_313296_313643	313296	3E+05	+	hypothetical protein		
NODE_8_length_386799_cov_42.541500	fig|6666666.34159.peg.2001	peg	NODE_8_length_386799_cov_42.541500_313913_313743	313913	3E+05	-	hypothetical protein		
NODE_8_length_386799_cov_42.541500	fig|6666666.34159.peg.2002	peg	NODE_8_length_386799_cov_42.541500_313876_314022	313876	3E+05	+	hypothetical protein		
NODE_8_length_386799_cov_42.541500	fig|6666666.34159.peg.2003	peg	NODE_8_length_386799_cov_42.541500_314534_314025	314534	3E+05	-	hypothetical protein		
NODE_8_length_386799_cov_42.541500	fig|6666666.34159.peg.2004	peg	NODE_8_length_386799_cov_42.541500_315830_314811	315830	3E+05	-	Alcohol dehydrogenase (EC 1.1.1.1)	FIG01007502	idu(3);Fermentations:_Mixed_acid idu(3);Butanol_Biosynthesis
NODE_8_length_386799_cov_42.541500	fig|6666666.34159.peg.2005	peg	NODE_8_length_386799_cov_42.541500_317224_315932	317224	3E+05	-	Dihydrolipoamide acetyltransferase component of pyruvate dehydrogenase complex (EC 2.3.1.12)	FIG00136675	icw(2);Pyruvate_metabolism_II:_acetyl-CoA,_acetogenesis_from_pyruvate icw(2);Dehydrogenase_complexes
NODE_8_length_386799_cov_42.541500	fig|6666666.34159.peg.2006	peg	NODE_8_length_386799_cov_42.541500_318231_317239	318231	3E+05	-	Pyruvate dehydrogenase E1 component beta subunit (EC 1.2.4.1)	FIG00025527	icw(1);Pyruvate_metabolism_II:_acetyl-CoA,_acetogenesis_from_pyruvate icw(1);Dehydrogenase_complexes
NODE_8_length_386799_cov_42.541500	fig|6666666.34159.peg.2007	peg	NODE_8_length_386799_cov_42.541500_319287_318256	319287	3E+05	-	Pyruvate dehydrogenase E1 component alpha subunit (EC 1.2.4.1)	FIG00053889	isu;Pyruvate_metabolism_II:_acetyl-CoA,_acetogenesis_from_pyruvate isu;Dehydrogenase_complexes
NODE_8_length_386799_cov_42.541500	fig|6666666.34159.peg.2008	peg	NODE_8_length_386799_cov_42.541500_320664_319411	320664	3E+05	-	Proton/glutamate symport protein @ Sodium/glutamate symport protein	FIG00008055	idu(2);Glutamate_and_Aspartate_uptake_in_Bacteria
NODE_8_length_386799_cov_42.541500	fig|6666666.34159.peg.2009	peg	NODE_8_length_386799_cov_42.541500_321876_321625	321876	3E+05	-	hypothetical protein		
NODE_8_length_386799_cov_42.541500	fig|6666666.34159.peg.2010	peg	NODE_8_length_386799_cov_42.541500_322450_321881	322450	3E+05	-	Membrane-associated phospholipid phosphatase		
NODE_8_length_386799_cov_42.541500	fig|6666666.34159.peg.2011	peg	NODE_8_length_386799_cov_42.541500_323158_322481	323158	3E+05	-	Hypothetical protein YggS, proline synthase co-transcribed bacterial homolog PROSC	FIG00003955	isu;A_Hypothetical_Protein_Related_to_Proline_Metabolism
NODE_8_length_386799_cov_42.541500	fig|6666666.34159.peg.2012	peg	NODE_8_length_386799_cov_42.541500_323886_323335	323886	3E+05	-	hypothetical protein		
NODE_8_length_386799_cov_42.541500	fig|6666666.34159.peg.2013	peg	NODE_8_length_386799_cov_42.541500_324582_324166	324582	3E+05	-	unknown protein	FIG00764722	ff
NODE_8_length_386799_cov_42.541500	fig|6666666.34159.peg.2014	peg	NODE_8_length_386799_cov_42.541500_325605_324586	325605	3E+05	-	DUF1432 domain-containing protein	FIG00648509	ff
NODE_8_length_386799_cov_42.541500	fig|6666666.34159.peg.2015	peg	NODE_8_length_386799_cov_42.541500_326108_325608	326108	3E+05	-	hypothetical protein	FIG00638284	ff
NODE_8_length_386799_cov_42.541500	fig|6666666.34159.peg.2016	peg	NODE_8_length_386799_cov_42.541500_328366_326105	328366	3E+05	-	hypothetical protein	FIG00638284	ff
NODE_8_length_386799_cov_42.541500	fig|6666666.34159.peg.2017	peg	NODE_8_length_386799_cov_42.541500_328368_328517	328368	3E+05	+	hypothetical protein		
NODE_8_length_386799_cov_42.541500	fig|6666666.34159.peg.2018	peg	NODE_8_length_386799_cov_42.541500_328885_328622	328885	3E+05	-	hypothetical protein		
NODE_8_length_386799_cov_42.541500	fig|6666666.34159.peg.2019	peg	NODE_8_length_386799_cov_42.541500_329013_330974	329013	3E+05	+	hypothetical protein	FIG00638284	ff
NODE_8_length_386799_cov_42.541500	fig|6666666.34159.peg.2020	peg	NODE_8_length_386799_cov_42.541500_331460_330966	331460	3E+05	-	putative regulatory protein, involved in type III secretion lcrH, sycD		
NODE_8_length_386799_cov_42.541500	fig|6666666.34159.peg.2021	peg	NODE_8_length_386799_cov_42.541500_331726_331481	331726	3E+05	-	unknown protein	FIG00764722	ff
NODE_8_length_386799_cov_42.541500	fig|6666666.34159.peg.2022	peg	NODE_8_length_386799_cov_42.541500_332116_333510	332116	3E+05	+	unknown protein	FIG00764722	ff
NODE_8_length_386799_cov_42.541500	fig|6666666.34159.peg.2023	peg	NODE_8_length_386799_cov_42.541500_334577_333507	334577	3E+05	-	DNA recombination and repair protein RecF	FIG00000412	icw(1);DNA_replication_cluster_1
NODE_8_length_386799_cov_42.541500	fig|6666666.34159.peg.2024	peg	NODE_8_length_386799_cov_42.541500_335713_334583	335713	3E+05	-	DNA polymerase III beta subunit (EC 2.7.7.7)	FIG00066425	isu;DNA_replication_cluster_1
NODE_8_length_386799_cov_42.541500	fig|6666666.34159.peg.2025	peg	NODE_8_length_386799_cov_42.541500_335955_336413	335955	3E+05	+	tmRNA-binding protein SmpB	FIG00000194	isu;Staphylococcal_pathogenicity_islands_SaPI isu;Heat_shock_dnaK_gene_cluster_extended isu;Translation_termination_factors_bacterial
NODE_8_length_386799_cov_42.541500	fig|6666666.34159.peg.2026	peg	NODE_8_length_386799_cov_42.541500_336421_336843	336421	3E+05	+	LSU m3Psi1915 methyltransferase RlmH	FIG01280345	isu;tRNA_modification_Bacteria isu;RNA_methylation
NODE_8_length_386799_cov_42.541500	fig|6666666.34159.peg.2027	peg	NODE_8_length_386799_cov_42.541500_337335_336844	337335	3E+05	-	unknown protein	FIG00764722	ff
NODE_8_length_386799_cov_42.541500	fig|6666666.34159.peg.2028	peg	NODE_8_length_386799_cov_42.541500_337910_339001	337910	3E+05	+	Pili retraction protein pilT	FIG00346989	ff
NODE_8_length_386799_cov_42.541500	fig|6666666.34159.peg.2029	peg	NODE_8_length_386799_cov_42.541500_339028_339432	339028	3E+05	+	Holo-[acyl-carrier protein] synthase (EC 2.7.8.7)	FIG00000540	isu;Fatty_Acid_Biosynthesis_FASII
NODE_8_length_386799_cov_42.541500	fig|6666666.34159.peg.2030	peg	NODE_8_length_386799_cov_42.541500_340262_339438	340262	3E+05	-	unknown protein	FIG00764722	ff
NODE_8_length_386799_cov_42.541500	fig|6666666.34159.peg.2031	peg	NODE_8_length_386799_cov_42.541500_341215_340265	341215	3E+05	-	Thioredoxin reductase (EC 1.8.1.9)	FIG00018396	isu;pyrimidine_conversions isu;Thioredoxin-disulfide_reductase isu;Glycine_reductase,_sarcosine_reductase_and [illegible]
NODE_8_length_386799_cov_42.541500	fig|6666666.34159.peg.2032	peg	NODE_8_length_386799_cov_42.541500_341422_341258	341422	3E+05	-	hypothetical protein		

NODE_8_length_386799_cov_42.541500	fig|6666666.34159.peg.2033	peg	NODE_8_length_386799_cov_42.541500_341997_341542	341997	3E+05	-	hypothetical protein		
NODE_8_length_386799_cov_42.541500	fig|6666666.34159.peg.2034	peg	NODE_8_length_386799_cov_42.541500_342439_343890	342439	3E+05	+	Proline/sodium symporter PutP (TC 2.A.21.2.1) @ Propionate/sodium symporter	FIG00034981	isu;Proline,_4-hydroxyproline_uptake_and_utilization
NODE_8_length_386799_cov_42.541500	fig|6666666.34159.peg.2035	peg	NODE_8_length_386799_cov_42.541500_344795_343887	344795	3E+05	-	1,4-dihydroxy-2-naphthoate octaprenyltransferase (EC 2.5.1.74)	FIG00085608	ff
NODE_8_length_386799_cov_42.541500	fig|6666666.34159.peg.2036	peg	NODE_8_length_386799_cov_42.541500_345679_344849	345679	3E+05	-	Naphthoate synthase (EC 4.1.3.36)	FIG00071623	isu;Menaquinone_and_Phylloquinone_Biosynthesis_--_gjo
NODE_8_length_386799_cov_42.541500	fig|6666666.34159.peg.2037	peg	NODE_8_length_386799_cov_42.541500_346418_345693	346418	3E+05	-	2-succinyl-6-hydroxy-2,4-cyclohexadiene-1-carboxylate synthase (EC 4.2.99.20)	FIG00016466	icw(2);Menaquinone_and_Phylloquinone_Biosynthesis_--_gjo
NODE_8_length_386799_cov_42.541500	fig|6666666.34159.peg.2038	peg	NODE_8_length_386799_cov_42.541500_348008_346425	348008	3E+05	-	2-succinyl-5-enolpyruvyl-6-hydroxy-3-cyclohexene-1-carboxylic-acid synthase (EC 2.2.1.9)	FIG00000866	icw(3);Menaquinone_and_Phylloquinone_Biosynthesis_--_gjo
NODE_8_length_386799_cov_42.541500	fig|6666666.34159.peg.2039	peg	NODE_8_length_386799_cov_42.541500_349048_348005	349048	3E+05	-	Isochorismate synthase (EC 5.4.4.2) @ Menaquinone-specific isochorismate synthase (EC 5.4.4.2)	FIG01304872	isu;Chorismate:_Intermediate_for_synthesis_of_Tryptophan,_PAPA_antibiotics,_PABA,_3-
NODE_8_length_386799_cov_42.541500	fig|6666666.34159.peg.2040	peg	NODE_8_length_386799_cov_42.541500_350798_349074	350798	3E+05	-	hypothetical protein	FIG00638284	ff
NODE_8_length_386799_cov_42.541500	fig|6666666.34159.peg.2041	peg	NODE_8_length_386799_cov_42.541500_350896_352245	350896	4E+05	+	RNA methyltransferase, TrmA family	FIG00004538	ff
NODE_8_length_386799_cov_42.541500	fig|6666666.34159.peg.2042	peg	NODE_8_length_386799_cov_42.541500_353418_352228	353418	4E+05	-	Acetate kinase (EC 2.7.2.1)	FIG00067248	isu;Pyruvate_metabolism_II:_acetyl-CoA,_acetogenesis_from_pyruvate
NODE_8_length_386799_cov_42.541500	fig|6666666.34159.peg.2043	peg	NODE_8_length_386799_cov_42.541500_353660_354958	353660	4E+05	+	hypothetical protein		
NODE_8_length_386799_cov_42.541500	fig|6666666.34159.rna.34	rna	NODE_8_length_386799_cov_42.541500_355062_355148	355062	4E+05	+	tRNA-Ser-CGA		isu;tRNAs
NODE_8_length_386799_cov_42.541500	fig|6666666.34159.peg.2044	peg	NODE_8_length_386799_cov_42.541500_355812_355168	355812	4E+05	-	conserved hypothetical protein		
NODE_8_length_386799_cov_42.541500	fig|6666666.34159.peg.2045	peg	NODE_8_length_386799_cov_42.541500_356569_355886	356569	4E+05	-	hypothetical protein	FIG00638284	ff
NODE_8_length_386799_cov_42.541500	fig|6666666.34159.peg.2046	peg	NODE_8_length_386799_cov_42.541500_356816_357268	356816	4E+05	+	hypothetical protein		
NODE_8_length_386799_cov_42.541500	fig|6666666.34159.peg.2047	peg	NODE_8_length_386799_cov_42.541500_357642_357265	357642	4E+05	-	hypothetical protein		
NODE_8_length_386799_cov_42.541500	fig|6666666.34159.peg.2048	peg	NODE_8_length_386799_cov_42.541500_357748_358641	357748	4E+05	+	hypothetical protein		
NODE_8_length_386799_cov_42.541500	fig|6666666.34159.peg.2049	peg	NODE_8_length_386799_cov_42.541500_360518_358638	360518	4E+05	-	hypothetical protein		
NODE_8_length_386799_cov_42.541500	fig|6666666.34159.peg.2050	peg	NODE_8_length_386799_cov_42.541500_360547_360678	360547	4E+05	+	hypothetical protein		
NODE_8_length_386799_cov_42.541500	fig|6666666.34159.peg.2051	peg	NODE_8_length_386799_cov_42.541500_360739_366522	360739	4E+05	+	Excinuclease ABC subunit A, dimeric form	FIG00133288	isu;DNA_repair,_UvrABC_system
NODE_8_length_386799_cov_42.541500	fig|6666666.34159.peg.2052	peg	NODE_8_length_386799_cov_42.541500_366688_368229	366688	4E+05	+	hypothetical protein		
NODE_8_length_386799_cov_42.541500	fig|6666666.34159.peg.2053	peg	NODE_8_length_386799_cov_42.541500_368871_368242	368871	4E+05	-	hypothetical protein		
NODE_8_length_386799_cov_42.541500	fig|6666666.34159.peg.2054	peg	NODE_8_length_386799_cov_42.541500_370790_368874	370790	4E+05	-	Topoisomerase IV subunit A (EC 5.99.1.-)	FIG00000425	icw(1);DNA_topoisomerases,_Type_II,_ATP-dependent icw(1);Resistance_to_fluoroquinolones
NODE_8_length_386799_cov_42.541500	fig|6666666.34159.peg.2055	peg	NODE_8_length_386799_cov_42.541500_372632_370794	372632	4E+05	-	Topoisomerase IV subunit B (EC 5.99.1.-)	FIG00000439	isu;DNA_topoisomerases,_Type_II,_ATP-dependent isu;Resistance_to_fluoroquinolones
NODE_8_length_386799_cov_42.541500	fig|6666666.34159.peg.2056	peg	NODE_8_length_386799_cov_42.541500_372729_373751	372729	4E+05	+	UDP-N-acetylglucosamine 4,6-dehydratase (EC 4.2.1.-)	FIG00003715	isu;CBSS-296591.1.peg.2330 isu;N-linked_Glycosylation_in_Bacteria
NODE_8_length_386799_cov_42.541500	fig|6666666.34159.peg.2057	peg	NODE_8_length_386799_cov_42.541500_374709_373741	374709	4E+05	-	hypothetical protein-signal peptide prediction	FIG01367919	ff
NODE_8_length_386799_cov_42.541500	fig|6666666.34159.peg.2058	peg	NODE_8_length_386799_cov_42.541500_374868_375899	374868	4E+05	+	Glutamyl-tRNA reductase (EC 1.2.1.70)	FIG00004795	isu;Heme_and_Siroheme_Biosynthesis
NODE_8_length_386799_cov_42.541500	fig|6666666.34159.peg.2059	peg	NODE_8_length_386799_cov_42.541500_376395_378944	376395	4E+05	+	DNA mismatch repair protein MutS	FIG00000356	isu;DNA_repair_system_including_RecA,_MutS_and_a_hypothetical_protein
NODE_8_length_386799_cov_42.541500	fig|6666666.34159.peg.2060	peg	NODE_8_length_386799_cov_42.541500_378966_379802	378966	4E+05	+	hydrolase HAD superfamily		
NODE_8_length_386799_cov_42.541500	fig|6666666.34159.peg.2061	peg	NODE_8_length_386799_cov_42.541500_379825_380625	379825	4E+05	+	Dihydroneopterin triphosphate pyrophosphohydolase / Dihydroneopterin aldolase (EC 4.1.2.25)	FIG00899409	icw(1);Folate_Biosynthesis icw(1);Folate_Biosynthesis isu;Queuosine-
NODE_8_length_386799_cov_42.541500	fig|6666666.34159.peg.2062	peg	NODE_8_length_386799_cov_42.541500_380627_381337	380627	4E+05	+	FolM Alternative dihydrofolate reductase 1	FIG00138402	isu;Folate_Biosynthesis
NODE_8_length_386799_cov_42.541500	fig|6666666.34159.peg.2063	peg	NODE_8_length_386799_cov_42.541500_381330_382808	381330	4E+05	+	unknown protein	FIG00764722	ff
NODE_8_length_386799_cov_42.541500	fig|6666666.34159.peg.2064	peg	NODE_8_length_386799_cov_42.541500_382814_383704	382814	4E+05	+	hypothetical protein	FIG00638284	ff
NODE_8_length_386799_cov_42.541500	fig|6666666.34159.peg.2065	peg	NODE_8_length_386799_cov_42.541500_384618_383701	384618	4E+05	-	Enoyl-[acyl-carrier-protein] reductase [NADH] (EC 1.3.1.9)	FIG00000524	isu;Fatty_Acid_Biosynthesis_FASII
NODE_8_length_386799_cov_42.541500	fig|6666666.34159.peg.2066	peg	NODE_8_length_386799_cov_42.541500_386347_384689	386347	4E+05	-	hypothetical protein	FIG00638284	ff
NODE_96_length_748272_cov_42.517338	fig|6666666.34159.peg.2067	peg	NODE_96_length_748272_cov_42.517338_2910_1504	2910	1504	-	ATP-dependent DNA helicase	FIG01349536	ff
NODE_96_length_748272_cov_42.517338	fig|6666666.34159.peg.2068	peg	NODE_96_length_748272_cov_42.517338_4058_3060	4058	3060	-	Proline iminopeptidase (EC 3.4.11.5)	FIG00000951	isu;Proline,_4-hydroxyproline_uptake_and_utilization
NODE_96_length_748272_cov_42.517338	fig|6666666.34159.peg.2069	peg	NODE_96_length_748272_cov_42.517338_4618_4127	4618	4127	-	putative acetyltransferase		
NODE_96_length_748272_cov_42.517338	fig|6666666.34159.peg.2070	peg	NODE_96_length_748272_cov_42.517338_5745_4684	5745	4684	-	aminoglycoside phosphotransferase		
NODE_96_length_748272_cov_42.517338	fig|6666666.34159.peg.2071	peg	NODE_96_length_748272_cov_42.517338_5878_5762	5878	5762	-	hypothetical protein		
NODE_96_length_748272_cov_42.517338	fig|6666666.34159.peg.2072	peg	NODE_96_length_748272_cov_42.517338_7052_6024	7052	6024	-	hypothetical protein		
NODE_96_length_748272_cov_42.517338	fig|6666666.34159.peg.2073	peg	NODE_96_length_748272_cov_42.517338_7375_7262	7375	7262	-	hypothetical protein		
NODE_96_length_748272_cov_42.517338	fig|6666666.34159.peg.2074	peg	NODE_96_length_748272_cov_42.517338_7893_7453	7893	7453	-	hypothetical protein		
NODE_96_length_748272_cov_42.517338	fig|6666666.34159.peg.2075	peg	NODE_96_length_748272_cov_42.517338_8476_7910	8476	7910	-	hypothetical protein		
NODE_96_length_748272_cov_42.517338	fig|6666666.34159.peg.2076	peg	NODE_96_length_748272_cov_42.517338_9554_8469	9554	8469	-	Phage integrase		
NODE_96_length_748272_cov_42.517338	fig|6666666.34159.rna.35	rna	NODE_96_length_748272_cov_42.517338_9744_9662	9744	9662	-	tRNA-Leu-TAA		
NODE_96_length_748272_cov_42.517338	fig|6666666.34159.peg.2077	peg	NODE_96_length_748272_cov_42.517338_12826_9857	12826	9857	-	metalloprotease, insulinase family		
NODE_96_length_748272_cov_42.517338	fig|6666666.34159.peg.2078	peg	NODE_96_length_748272_cov_42.517338_13149_14174	13149	14174	+	NAD-dependent glyceraldehyde-3-phosphate dehydrogenase (EC 1.2.1.12)	FIG00132586	isu;Calvin-Benson_cycle isu;Pyridoxin_(Vitamin_B6)_Biosynthesis
NODE_96_length_748272_cov_42.517338	fig|6666666.34159.peg.2079	peg	NODE_96_length_748272_cov_42.517338_14309_15463	14309	15463	+	FIG00494136: hypothetical protein	FIG00493445	ff
NODE_96_length_748272_cov_42.517338	fig|6666666.34159.peg.2080	peg	NODE_96_length_748272_cov_42.517338_16147_15515	16147	15515	-	Carbonic anhydrase (EC 4.2.1.1)	FIG00023019	idu(2);Cyanate_hydrolysis
NODE_96_length_748272_cov_42.517338	fig|6666666.34159.peg.2081	peg	NODE_96_length_748272_cov_42.517338_18599_16620	18599	16620	-	Chaperone protein DnaK	FIG00023369	icw(1);Heat_shock_dnaK_gene_cluster_extended icw(1);Protein_chaperones
NODE_96_length_748272_cov_42.517338	fig|6666666.34159.peg.2082	peg	NODE_96_length_748272_cov_42.517338_19271_18627	19271	18627	-	Heat shock protein GrpE	FIG00000103	icw(2);Heat_shock_dnaK_gene_cluster_extended isu;Protein_chaperones
NODE_96_length_748272_cov_42.517338	fig|6666666.34159.peg.2083	peg	NODE_96_length_748272_cov_42.517338_20460_19315	20460	19315	-	Heat-inducible transcription repressor HrcA	FIG00000544	isu;Heat_shock_dnaK_gene_cluster_extended
NODE_96_length_748272_cov_42.517338	fig|6666666.34159.peg.2084	peg	NODE_96_length_748272_cov_42.517338_23659_20540	23659	20540	-	Glu/Leu/Phe/Val dehydrogenase family protein	FIG01323562	ff
NODE_96_length_748272_cov_42.517338	fig|6666666.34159.peg.2085	peg	NODE_96_length_748272_cov_42.517338_24194_23829	24194	23829	-	hypothetical protein		
NODE_96_length_748272_cov_42.517338	fig|6666666.34159.peg.2086	peg	NODE_96_length_748272_cov_42.517338_26279_24267	26279	24267	-	Lead, cadmium, zinc and mercury transporting ATPase (EC 3.6.3.3) (EC 3.6.3.5); Copper-translocating P-type ATPase (EC	FIG00658111	idu(3);Copper_Transport_System idu(3);CBSS-196620.1.peg.2477 idu(3);Copper_homeostasis
NODE_96_length_748272_cov_42.517338	fig|6666666.34159.peg.2087	peg	NODE_96_length_748272_cov_42.517338_27045_26287	27045	26287	-	hypothetical protein		
NODE_96_length_748272_cov_42.517338	fig|6666666.34159.peg.2088	peg	NODE_96_length_748272_cov_42.517338_27081_27209	27081	27209	+	hypothetical protein		
NODE_96_length_748272_cov_42.517338	fig|6666666.34159.peg.2089	peg	NODE_96_length_748272_cov_42.517338_27264_28400	27264	28400	+	hypothetical protein		

NODE_96_length_748272_cov_42.517338	fig|6666666.34159.peg.2090	peg	NODE_96_length_748272_cov_42.517338_28397_29059	28397	29059	+	polysaccharide deacetylase		
NODE_96_length_748272_cov_42.517338	fig|6666666.34159.peg.2091	peg	NODE_96_length_748272_cov_42.517338_29056_30654	29056	30654	+	hypothetical protein		
NODE_96_length_748272_cov_42.517338	fig|6666666.34159.peg.2092	peg	NODE_96_length_748272_cov_42.517338_30671_31792	30671	31792	+	putative protein, slightly similar to UDP-N-acetylglucosamine-N-acetylmuramyl-(pentapeptide) pyrophosphoryl-undecaprenol N-acetylglucosamine transferase		
NODE_96_length_748272_cov_42.517338	fig|6666666.34159.peg.2093	peg	NODE_96_length_748272_cov_42.517338_32654_31782	32654	31782	-	hypothetical protein		
NODE_96_length_748272_cov_42.517338	fig|6666666.34159.peg.2094	peg	NODE_96_length_748272_cov_42.517338_32864_34867	32864	34867	+	Transketolase (EC 2.2.1.1)	FIG00020289	isu;Calvin-Benson_cycle isu;Pentose_phosphate_pathway
NODE_96_length_748272_cov_42.517338	fig|6666666.34159.peg.2095	peg	NODE_96_length_748272_cov_42.517338_34881_35900	34881	35900	+	Uroporphyrinogen III decarboxylase (EC 4.1.1.37)	FIG00000428	icw(1);Heme_and_Siroheme_Biosynthesis
NODE_96_length_748272_cov_42.517338	fig|6666666.34159.peg.2096	peg	NODE_96_length_748272_cov_42.517338_35890_37287	35890	37287	+	Protoporphyrinogen IX oxidase, aerobic, HemY (EC 1.3.3.4)	FIG00068302	isu;Heme_and_Siroheme_Biosynthesis
NODE_96_length_748272_cov_42.517338	fig|6666666.34159.peg.2097	peg	NODE_96_length_748272_cov_42.517338_37703_41851	37703	41851	+	hypothetical protein		
NODE_96_length_748272_cov_42.517338	fig|6666666.34159.peg.2098	peg	NODE_96_length_748272_cov_42.517338_41865_42974	41865	42974	+	Glutamate 5-kinase (EC 2.7.2.11)	FIG00000351	icw(2);Proline_Synthesis
NODE_96_length_748272_cov_42.517338	fig|6666666.34159.peg.2099	peg	NODE_96_length_748272_cov_42.517338_42993_44267	42993	44267	+	Gamma-glutamyl phosphate reductase (EC 1.2.1.41)	FIG00000365	icw(1);Proline_Synthesis
NODE_96_length_748272_cov_42.517338	fig|6666666.34159.peg.2100	peg	NODE_96_length_748272_cov_42.517338_44269_45075	44269	45075	+	Pyrroline-5-carboxylate reductase (EC 1.5.1.2)	FIG00133325	isu;Proline_Synthesis isu;A_Hypothetical_Protein_Related_to_Proline_Metabolism
NODE_96_length_748272_cov_42.517338	fig|6666666.34159.peg.2101	peg	NODE_96_length_748272_cov_42.517338_46205_45438	46205	45438	-	FIG137478: Hypothetical protein	FIG01303938	isu;tRNA_modification_Bacteria
NODE_96_length_748272_cov_42.517338	fig|6666666.34159.peg.2102	peg	NODE_96_length_748272_cov_42.517338_47389_48639	47389	48639	+	Collagen triple helix repeat protein		
NODE_96_length_748272_cov_42.517338	fig|6666666.34159.peg.2103	peg	NODE_96_length_748272_cov_42.517338_48657_49184	48657	49184	+	hypothetical protein		
NODE_96_length_748272_cov_42.517338	fig|6666666.34159.peg.2104	peg	NODE_96_length_748272_cov_42.517338_49242_49847	49242	49847	+	hypothetical protein		
NODE_96_length_748272_cov_42.517338	fig|6666666.34159.peg.2105	peg	NODE_96_length_748272_cov_42.517338_50283_50657	50283	50657	+	hypothetical protein	FIG00638284	ff
NODE_96_length_748272_cov_42.517338	fig|6666666.34159.peg.2106	peg	NODE_96_length_748272_cov_42.517338_50785_52257	50785	52257	+	Serine hydroxymethyltransferase (EC 2.1.2.1)	FIG00000040	isu;Glycine_and_Serine_Utilization isu;Serine_Biosynthesis isu;Glycine_Biosynthesis
NODE_96_length_748272_cov_42.517338	fig|6666666.34159.peg.2107	peg	NODE_96_length_748272_cov_42.517338_52314_53312	52314	53312	+	Glycerol-3-phosphate dehydrogenase [NAD(P)+] (EC 1.1.1.94)	FIG00000309	isu;Glycerol_and_Glycerol-3-phosphate_Uptake_and_Utilization
NODE_96_length_748272_cov_42.517338	fig|6666666.34159.peg.2108	peg	NODE_96_length_748272_cov_42.517338_53353_54861	53353	54861	+	NAD(P)HX epimerase / NAD(P)HX dehydratase	FIG00000592	isu;YjeE isu;YjeE
NODE_96_length_748272_cov_42.517338	fig|6666666.34159.peg.2109	peg	NODE_96_length_748272_cov_42.517338_55211_55579	55211	55579	+	hypothetical protein		
NODE_96_length_748272_cov_42.517338	fig|6666666.34159.peg.2110	peg	NODE_96_length_748272_cov_42.517338_56045_55584	56045	55584	-	hypothetical protein		
NODE_96_length_748272_cov_42.517338	fig|6666666.34159.peg.2111	peg	NODE_96_length_748272_cov_42.517338_56658_56014	56658	56014	-	hypothetical protein		
NODE_96_length_748272_cov_42.517338	fig|6666666.34159.peg.2112	peg	NODE_96_length_748272_cov_42.517338_56785_56898	56785	56898	+	hypothetical protein		
NODE_96_length_748272_cov_42.517338	fig|6666666.34159.peg.2113	peg	NODE_96_length_748272_cov_42.517338_57041_57766	57041	57766	+	hypothetical protein		
NODE_96_length_748272_cov_42.517338	fig|6666666.34159.peg.2114	peg	NODE_96_length_748272_cov_42.517338_58356_57769	58356	57769	-	hypothetical protein		
NODE_96_length_748272_cov_42.517338	fig|6666666.34159.peg.2115	peg	NODE_96_length_748272_cov_42.517338_58428_59147	58428	59147	+	Lipoate-protein ligase A	FIG00000465	isu;Lipoic_acid_metabolism
NODE_96_length_748272_cov_42.517338	fig|6666666.34159.peg.2116	peg	NODE_96_length_748272_cov_42.517338_59676_59296	59676	59296	-	conserved hypothetical protein, partial length	FIG01617469	ff
NODE_96_length_748272_cov_42.517338	fig|6666666.34159.peg.2117	peg	NODE_96_length_748272_cov_42.517338_62543_59676	62543	59676	-	putative component D of type II secretion pathway	FIG01584795	ff
NODE_96_length_748272_cov_42.517338	fig|6666666.34159.peg.2118	peg	NODE_96_length_748272_cov_42.517338_63538_62552	63538	62552	-	hypothetical protein	FIG00638284	ff
NODE_96_length_748272_cov_42.517338	fig|6666666.34159.peg.2119	peg	NODE_96_length_748272_cov_42.517338_66315_63541	66315	63541	-	Type III secretion protein SctC		
NODE_96_length_748272_cov_42.517338	fig|6666666.34159.peg.2120	peg	NODE_96_length_748272_cov_42.517338_68187_66586	68187	66586	-	FIG047302: Type III secretion S/T Protein Kinase	FIG00493683	ff
NODE_96_length_748272_cov_42.517338	fig|6666666.34159.peg.2121	peg	NODE_96_length_748272_cov_42.517338_69654_68266	69654	68266	-	hypothetical protein		
NODE_96_length_748272_cov_42.517338	fig|6666666.34159.peg.2122	peg	NODE_96_length_748272_cov_42.517338_70758_69754	70758	69754	-	FIG046930: Type III secretion protein	FIG00493130	ff
NODE_96_length_748272_cov_42.517338	fig|6666666.34159.peg.2123	peg	NODE_96_length_748272_cov_42.517338_71276_70785	71276	70785	-	FIG016940: Type III secretion protein	FIG00430439	ff
NODE_96_length_748272_cov_42.517338	fig|6666666.34159.peg.2124	peg	NODE_96_length_748272_cov_42.517338_72612_71296	72612	71296	-	Type III secretion cytoplasmic ATP synthase (EC 3.6.3.14, YscN,SpaL,MxiB,HrcN,EscN)	FIG01304599	ff
NODE_96_length_748272_cov_42.517338	fig|6666666.34159.peg.2125	peg	NODE_96_length_748272_cov_42.517338_73390_72722	73390	72722	-	hypothetical protein	FIG00638284	ff
NODE_96_length_748272_cov_42.517338	fig|6666666.34159.peg.2126	peg	NODE_96_length_748272_cov_42.517338_73954_73487	73954	73487	-	FIG016921: Type III secretion protein	FIG00493250	ff
NODE_96_length_748272_cov_42.517338	fig|6666666.34159.peg.2127	peg	NODE_96_length_748272_cov_42.517338_74354_74079	74354	74079	-	unknown protein	FIG00764722	ff
NODE_96_length_748272_cov_42.517338	fig|6666666.34159.peg.2128	peg	NODE_96_length_748272_cov_42.517338_74828_74583	74828	74583	-	FIG016943: Type III secretion	FIG00493537	ff
NODE_96_length_748272_cov_42.517338	fig|6666666.34159.peg.2129	peg	NODE_96_length_748272_cov_42.517338_77286_74959	77286	74959	-	hypothetical protein		
NODE_96_length_748272_cov_42.517338	fig|6666666.34159.peg.2130	peg	NODE_96_length_748272_cov_42.517338_77642_79123	77642	79123	+	hypothetical protein	FIG00638284	ff
NODE_96_length_748272_cov_42.517338	fig|6666666.34159.peg.2131	peg	NODE_96_length_748272_cov_42.517338_79209_80432	79209	80432	+	hypothetical protein		
NODE_96_length_748272_cov_42.517338	fig|6666666.34159.peg.2132	peg	NODE_96_length_748272_cov_42.517338_81269_80457	81269	80457	-	ABC transporter, permease protein		
NODE_96_length_748272_cov_42.517338	fig|6666666.34159.peg.2133	peg	NODE_96_length_748272_cov_42.517338_82038_81250	82038	81250	-	hypothetical protein		
NODE_96_length_748272_cov_42.517338	fig|6666666.34159.peg.2134	peg	NODE_96_length_748272_cov_42.517338_82766_82041	82766	82041	-	ABC transporter, ATP-binding protein	FIG00744535	ff
NODE_96_length_748272_cov_42.517338	fig|6666666.34159.peg.2135	peg	NODE_96_length_748272_cov_42.517338_83110_82934	83110	82934	-	hypothetical protein		
NODE_96_length_748272_cov_42.517338	fig|6666666.34159.peg.2136	peg	NODE_96_length_748272_cov_42.517338_83252_84892	83252	84892	+	hypothetical protein		
NODE_96_length_748272_cov_42.517338	fig|6666666.34159.peg.2137	peg	NODE_96_length_748272_cov_42.517338_85291_84926	85291	84926	-	transcriptional coactivator/pterin dehydratase		
NODE_96_length_748272_cov_42.517338	fig|6666666.34159.peg.2138	peg	NODE_96_length_748272_cov_42.517338_85518_85270	85518	85270	-	hypothetical protein		
NODE_96_length_748272_cov_42.517338	fig|6666666.34159.peg.2139	peg	NODE_96_length_748272_cov_42.517338_86891_85515	86891	85515	-	hypothetical protein		
NODE_96_length_748272_cov_42.517338	fig|6666666.34159.peg.2140	peg	NODE_96_length_748272_cov_42.517338_87436_87005	87436	87005	-	Queuosine biosynthesis QueD, PTPS-I	FIG00138171	idu(1);tRNA_modification_Bacteria idu(1);Queuosine-Archaeosine_Biosynthesis
NODE_96_length_748272_cov_42.517338	fig|6666666.34159.peg.2141	peg	NODE_96_length_748272_cov_42.517338_88076_87450	88076	87450	-	hypothetical protein		
NODE_96_length_748272_cov_42.517338	fig|6666666.34159.peg.2142	peg	NODE_96_length_748272_cov_42.517338_89163_88399	89163	88399	-	hypothetical protein		
NODE_96_length_748272_cov_42.517338	fig|6666666.34159.peg.2143	peg	NODE_96_length_748272_cov_42.517338_89432_91597	89432	91597	+	hypothetical protein	FIG00638284	ff
NODE_96_length_748272_cov_42.517338	fig|6666666.34159.peg.2144	peg	NODE_96_length_748272_cov_42.517338_91864_91745	91864	91745	-	hypothetical protein		
NODE_96_length_748272_cov_42.517338	fig|6666666.34159.peg.2145	peg	NODE_96_length_748272_cov_42.517338_92179_93792	92179	93792	+	hypothetical protein		
NODE_96_length_748272_cov_42.517338	fig|6666666.34159.peg.2146	peg	NODE_96_length_748272_cov_42.517338_94152_94039	94152	94039	-	hypothetical protein		
NODE_96_length_748272_cov_42.517338	fig|6666666.34159.peg.2147	peg	NODE_96_length_748272_cov_42.517338_94131_96938	94131	96938	+	hypothetical protein	FIG00638284	ff
NODE_96_length_748272_cov_42.517338	fig|6666666.34159.peg.2148	peg	NODE_96_length_748272_cov_42.517338_99799_97010	99799	97010	-	hypothetical protein	FIG00638284	ff

NODE_96_length_748272_cov_42.517338	fig|6666666.34159.peg.2149	peg	NODE_96_length_748272_cov_42.517338_100420_100235	100420	1E+05	-	hypothetical protein		
NODE_96_length_748272_cov_42.517338	fig|6666666.34159.peg.2150	peg	NODE_96_length_748272_cov_42.517338_100592_100443	100592	1E+05	-	hypothetical protein		
NODE_96_length_748272_cov_42.517338	fig|6666666.34159.peg.2151	peg	NODE_96_length_748272_cov_42.517338_100638_101708	100638	1E+05	+	putative rhs core protein with extension		
NODE_96_length_748272_cov_42.517338	fig|6666666.34159.peg.2152	peg	NODE_96_length_748272_cov_42.517338_101813_102376	101813	1E+05	+	hypothetical protein		
NODE_96_length_748272_cov_42.517338	fig|6666666.34159.peg.2153	peg	NODE_96_length_748272_cov_42.517338_102386_102697	102386	1E+05	+	hypothetical protein		
NODE_96_length_748272_cov_42.517338	fig|6666666.34159.peg.2154	peg	NODE_96_length_748272_cov_42.517338_102809_102967	102809	1E+05	+	hypothetical protein		
NODE_96_length_748272_cov_42.517338	fig|6666666.34159.peg.2155	peg	NODE_96_length_748272_cov_42.517338_103265_103149	103265	1E+05	-	hypothetical protein		
NODE_96_length_748272_cov_42.517338	fig|6666666.34159.peg.2156	peg	NODE_96_length_748272_cov_42.517338_104029_103799	104029	1E+05	-	transposase		
NODE_96_length_748272_cov_42.517338	fig|6666666.34159.peg.2157	peg	NODE_96_length_748272_cov_42.517338_104481_104642	104481	1E+05	+	hypothetical protein		
NODE_96_length_748272_cov_42.517338	fig|6666666.34159.peg.2158	peg	NODE_96_length_748272_cov_42.517338_106894_105086	106894	1E+05	-	hypothetical protein		
NODE_96_length_748272_cov_42.517338	fig|6666666.34159.peg.2159	peg	NODE_96_length_748272_cov_42.517338_108754_107063	108754	1E+05	-	hypothetical protein		
NODE_96_length_748272_cov_42.517338	fig|6666666.34159.peg.2160	peg	NODE_96_length_748272_cov_42.517338_109397_109008	109397	1E+05	-	hypothetical protein		
NODE_96_length_748272_cov_42.517338	fig|6666666.34159.peg.2161	peg	NODE_96_length_748272_cov_42.517338_110652_109456	110652	1E+05	-	hypothetical protein	FIG00638284	ff
NODE_96_length_748272_cov_42.517338	fig|6666666.34159.peg.2162	peg	NODE_96_length_748272_cov_42.517338_112311_110704	112311	1E+05	-	hypothetical protein		
NODE_96_length_748272_cov_42.517338	fig|6666666.34159.peg.2163	peg	NODE_96_length_748272_cov_42.517338_113157_112378	113157	1E+05	-	type III secretion system chaperone, LcrH/SycD family		
NODE_96_length_748272_cov_42.517338	fig|6666666.34159.peg.2164	peg	NODE_96_length_748272_cov_42.517338_115750_113192	115750	1E+05	-	hypothetical protein	FIG00638284	ff
NODE_96_length_748272_cov_42.517338	fig|6666666.34159.peg.2165	peg	NODE_96_length_748272_cov_42.517338_116373_115804	116373	1E+05	-	type III secretion chaperone	FIG00493336	ff
NODE_96_length_748272_cov_42.517338	fig|6666666.34159.peg.2166	peg	NODE_96_length_748272_cov_42.517338_117688_116504	117688	1E+05	-	unknown protein	FIG00764722	ff
NODE_96_length_748272_cov_42.517338	fig|6666666.34159.peg.2167	peg	NODE_96_length_748272_cov_42.517338_119069_117822	119069	1E+05	-	unknown protein	FIG00764722	ff
NODE_96_length_748272_cov_42.517338	fig|6666666.34159.peg.2168	peg	NODE_96_length_748272_cov_42.517338_122137_119264	122137	1E+05	-	hypothetical protein	FIG00638284	ff
NODE_96_length_748272_cov_42.517338	fig|6666666.34159.peg.2169	peg	NODE_96_length_748272_cov_42.517338_122900_123640	122900	1E+05	+	hypothetical protein		
NODE_96_length_748272_cov_42.517338	fig|6666666.34159.peg.2170	peg	NODE_96_length_748272_cov_42.517338_123997_124134	123997	1E+05	+	hypothetical protein		
NODE_96_length_748272_cov_42.517338	fig|6666666.34159.peg.2171	peg	NODE_96_length_748272_cov_42.517338_124169_124573	124169	1E+05	+	hypothetical protein	FIG00638284	ff
NODE_96_length_748272_cov_42.517338	fig|6666666.34159.peg.2172	peg	NODE_96_length_748272_cov_42.517338_124982_124836	124982	1E+05	-	hypothetical protein		
NODE_96_length_748272_cov_42.517338	fig|6666666.34159.peg.2173	peg	NODE_96_length_748272_cov_42.517338_125084_125368	125084	1E+05	+	hypothetical protein		
NODE_96_length_748272_cov_42.517338	fig|6666666.34159.peg.2174	peg	NODE_96_length_748272_cov_42.517338_126188_125940	126188	1E+05	-	hypothetical protein		
NODE_96_length_748272_cov_42.517338	fig|6666666.34159.peg.2175	peg	NODE_96_length_748272_cov_42.517338_126521_128095	126521	1E+05	+	hypothetical protein		
NODE_96_length_748272_cov_42.517338	fig|6666666.34159.peg.2176	peg	NODE_96_length_748272_cov_42.517338_128250_129869	128250	1E+05	+	hypothetical protein		
NODE_96_length_748272_cov_42.517338	fig|6666666.34159.peg.2177	peg	NODE_96_length_748272_cov_42.517338_130780_129902	130780	1E+05	-	hypothetical protein	FIG00638284	ff
NODE_96_length_748272_cov_42.517338	fig|6666666.34159.peg.2178	peg	NODE_96_length_748272_cov_42.517338_130775_130948	130775	1E+05	+	hypothetical protein		
NODE_96_length_748272_cov_42.517338	fig|6666666.34159.peg.2179	peg	NODE_96_length_748272_cov_42.517338_131378_130905	131378	1E+05	-	hypothetical protein		
NODE_96_length_748272_cov_42.517338	fig|6666666.34159.peg.2180	peg	NODE_96_length_748272_cov_42.517338_132222_131371	132222	1E+05	-	Pseudouridylate synthase, 23S RNA-specific(EC:4.2.1.70)		
NODE_96_length_748272_cov_42.517338	fig|6666666.34159.peg.2181	peg	NODE_96_length_748272_cov_42.517338_132730_134166	132730	1E+05	+	Iron-sulfur cluster assembly protein SufB	FIG00000451	icw(1);Iron-sulfur_cluster_assembly icw(4);tRNA_modification_Bacteria [illegible]
NODE_96_length_748272_cov_42.517338	fig|6666666.34159.peg.2182	peg	NODE_96_length_748272_cov_42.517338_134183_134944	134183	1E+05	+	Iron-sulfur cluster assembly ATPase protein SufC	FIG00018727	icw(3);Iron-sulfur_cluster_assembly isu;tRNA_modification_Bacteria
NODE_96_length_748272_cov_42.517338	fig|6666666.34159.peg.2183	peg	NODE_96_length_748272_cov_42.517338_134941_136245	134941	1E+05	+	Iron-sulfur cluster assembly protein SufD	FIG00072797	icw(2);Iron-sulfur_cluster_assembly icw(2);tRNA_modification_Bacteria
NODE_96_length_748272_cov_42.517338	fig|6666666.34159.peg.2184	peg	NODE_96_length_748272_cov_42.517338_136258_137493	136258	1E+05	+	Cysteine desulfurase (EC 2.8.1.7), SufS subfamily	FIG00229199	isu;Iron-sulfur_cluster_assembly icw(3);tRNA_modification_Bacteria [illegible]
NODE_96_length_748272_cov_42.517338	fig|6666666.34159.peg.2185	peg	NODE_96_length_748272_cov_42.517338_137503_138447	137503	1E+05	+	YrdC/Sua5 family protein, required for threonylcarbamoyladenosine (t(6)A) formation in tRNA	FIG00012971	icw(1);tRNA_modification_Bacteria
NODE_96_length_748272_cov_42.517338	fig|6666666.34159.peg.2186	peg	NODE_96_length_748272_cov_42.517338_138603_141158	138603	1E+05	+	ATP-dependent Clp protease, ATP-binding subunit ClpC / Negative regulator of genetic competence clcC/mecB	FIG00011902	isu;Proteolysis_in_bacteria,_ATP-dependent
NODE_96_length_748272_cov_42.517338	fig|6666666.34159.peg.2187	peg	NODE_96_length_748272_cov_42.517338_141511_141200	141511	1E+05	-	hypothetical protein		
NODE_96_length_748272_cov_42.517338	fig|6666666.34159.peg.2188	peg	NODE_96_length_748272_cov_42.517338_142367_141621	142367	1E+05	-	PTS system, IIA component	FIG01317636	ff
NODE_96_length_748272_cov_42.517338	fig|6666666.34159.peg.2189	peg	NODE_96_length_748272_cov_42.517338_142842_142393	142842	1E+05	-	PTS IIA Protein		
NODE_96_length_748272_cov_42.517338	fig|6666666.34159.peg.2190	peg	NODE_96_length_748272_cov_42.517338_143335_142889	143335	1E+05	-	Deoxyuridine 5'-triphosphate nucleotidohydrolase (EC 3.6.1.23)	FIG00000388	isu;Nudix_proteins_(nucleoside_triphosphate_hydrolases) [illegible]
NODE_96_length_748272_cov_42.517338	fig|6666666.34159.peg.2191	peg	NODE_96_length_748272_cov_42.517338_144262_143357	144262	1E+05	-	Acetyl-coenzyme A carboxyl transferase beta chain (EC 6.4.1.2)	FIG00000437	isu;Fatty_Acid_Biosynthesis_FASII
NODE_96_length_748272_cov_42.517338	fig|6666666.34159.peg.2192	peg	NODE_96_length_748272_cov_42.517338_145082_144468	145082	1E+05	-	Superoxide dismutase [Mn] (EC 1.15.1.1)	FIG00050592	isu;Oxidative_stress
NODE_96_length_748272_cov_42.517338	fig|6666666.34159.peg.2193	peg	NODE_96_length_748272_cov_42.517338_146113_145325	146113	1E+05	-	putative phosphoprotein phosphatase		
NODE_96_length_748272_cov_42.517338	fig|6666666.34159.peg.2194	peg	NODE_96_length_748272_cov_42.517338_146453_146325	146453	1E+05	-	hypothetical protein		
NODE_96_length_748272_cov_42.517338	fig|6666666.34159.peg.2195	peg	NODE_96_length_748272_cov_42.517338_147428_146721	147428	1E+05	-	hypothetical protein	FIG00638284	ff
NODE_96_length_748272_cov_42.517338	fig|6666666.34159.peg.2196	peg	NODE_96_length_748272_cov_42.517338_148121_147930	148121	1E+05	-	hypothetical protein		
NODE_96_length_748272_cov_42.517338	fig|6666666.34159.peg.2197	peg	NODE_96_length_748272_cov_42.517338_148771_148136	148771	1E+05	-	Uroporphyrinogen-III synthase (EC 4.2.1.75)	FIG00056694	isu;Heme_and_Siroheme_Biosynthesis
NODE_96_length_748272_cov_42.517338	fig|6666666.34159.peg.2198	peg	NODE_96_length_748272_cov_42.517338_149442_148750	149442	1E+05	-	Porphobilinogen deaminase (EC 2.5.1.61)	FIG00000363	icw(1);Heme_and_Siroheme_Biosynthesis
NODE_96_length_748272_cov_42.517338	fig|6666666.34159.peg.2199	peg	NODE_96_length_748272_cov_42.517338_150799_149426	150799	1E+05	-	DNA repair protein RadA	FIG00000283	isu;Proteolysis_in_bacteria,_ATP-dependent isu;DNA_repair,_bacterial
NODE_96_length_748272_cov_42.517338	fig|6666666.34159.peg.2200	peg	NODE_96_length_748272_cov_42.517338_151522_150812	151522	2E+05	-	Ribonuclease III (EC 3.1.26.3)	FIG00000208	isu;RNA_processing_and_degradation,_bacterial
NODE_96_length_748272_cov_42.517338	fig|6666666.34159.peg.2201	peg	NODE_96_length_748272_cov_42.517338_152212_151586	152212	2E+05	-	hypothetical protein		
NODE_96_length_748272_cov_42.517338	fig|6666666.34159.peg.2202	peg	NODE_96_length_748272_cov_42.517338_152255_152377	152255	2E+05	+	hypothetical protein		
NODE_96_length_748272_cov_42.517338	fig|6666666.34159.peg.2203	peg	NODE_96_length_748272_cov_42.517338_153193_152648	153193	2E+05	-	dTDP-4-dehydrorhamnose 3,5-epimerase (EC 5.1.3.13)	FIG00000408	icw(1);dTDP-rhamnose_synthesis icw(1);Rhamnose_containing_glycans [illegible]
NODE_96_length_748272_cov_42.517338	fig|6666666.34159.peg.2204	peg	NODE_96_length_748272_cov_42.517338_154068_153199	154068	2E+05	-	Glucose-1-phosphate thymidylyltransferase (EC 2.7.7.24)	FIG00000187	icw(2);dTDP-rhamnose_synthesis icw(2);Rhamnose_containing_glycans
NODE_96_length_748272_cov_42.517338	fig|6666666.34159.peg.2205	peg	NODE_96_length_748272_cov_42.517338_155129_154071	155129	2E+05	-	dTDP-glucose 4,6-dehydratase (EC 4.2.1.46)	FIG00030688	idu(1);dTDP-rhamnose_synthesis idu(1);Rhamnose_containing_glycans [illegible]
NODE_96_length_748272_cov_42.517338	fig|6666666.34159.peg.2206	peg	NODE_96_length_748272_cov_42.517338_156423_155281	156423	2E+05	-	Branched-chain amino acid transport system carrier protein	FIG00905542	ff
NODE_96_length_748272_cov_42.517338	fig|6666666.34159.peg.2207	peg	NODE_96_length_748272_cov_42.517338_156972_157247	156972	2E+05	+	hypothetical protein		

NODE_96_length_748272_cov_42.517338	fig|6666666.34159.peg.2208	peg	NODE_96_length_748272_cov_42.517338_157483_157367	157483	2E+05	-	hypothetical protein		
NODE_96_length_748272_cov_42.517338	fig|6666666.34159.peg.2209	peg	NODE_96_length_748272_cov_42.517338_157740_157612	157740	2E+05	-	hypothetical protein		
NODE_96_length_748272_cov_42.517338	fig|6666666.34159.peg.2210	peg	NODE_96_length_748272_cov_42.517338_157727_157945	157727	2E+05	+	hypothetical protein		
NODE_96_length_748272_cov_42.517338	fig|6666666.34159.peg.2211	peg	NODE_96_length_748272_cov_42.517338_158033_159055	158033	2E+05	+	hypothetical protein		
NODE_96_length_748272_cov_42.517338	fig|6666666.34159.peg.2212	peg	NODE_96_length_748272_cov_42.517338_159068_160108	159068	2E+05	+	hypothetical protein		
NODE_96_length_748272_cov_42.517338	fig|6666666.34159.peg.2213	peg	NODE_96_length_748272_cov_42.517338_161997_160213	161997	2E+05	-	EF hand domain/PKD domain protein		
NODE_96_length_748272_cov_42.517338	fig|6666666.34159.peg.2214	peg	NODE_96_length_748272_cov_42.517338_162912_162097	162912	2E+05	-	Ribosomal protein L11 methyltransferase (EC 2.1.1.-)	FIG00000346	isu;Heat_shock_dnaK_gene_cluster_extended
NODE_96_length_748272_cov_42.517338	fig|6666666.34159.peg.2215	peg	NODE_96_length_748272_cov_42.517338_164562_162913	164562	2E+05	-	Potassium uptake protein TrkH	FIG00000736	icw(1);Potassium_homeostasis<br>icw(1);Hyperosmotic_potassium_uptake
NODE_96_length_748272_cov_42.517338	fig|6666666.34159.peg.2216	peg	NODE_96_length_748272_cov_42.517338_165914_164565	165914	2E+05	-	Trk system potassium uptake protein TrkA		isu;Potassium_homeostasis<br>isu;Hyperosmotic_potassium_uptake
NODE_96_length_748272_cov_42.517338	fig|6666666.34159.peg.2217	peg	NODE_96_length_748272_cov_42.517338_166684_166004	166684	2E+05	-	Biotin carboxyl carrier protein	FIG00005415	isu;Fatty_Acid_Biosynthesis_FASII
NODE_96_length_748272_cov_42.517338	fig|6666666.34159.peg.2218	peg	NODE_96_length_748272_cov_42.517338_167675_166707	167675	2E+05	-	dihydrouridine synthase, DuS		
NODE_96_length_748272_cov_42.517338	fig|6666666.34159.peg.2219	peg	NODE_96_length_748272_cov_42.517338_167939_168226	167939	2E+05	+	hypothetical protein		
NODE_96_length_748272_cov_42.517338	fig|6666666.34159.peg.2220	peg	NODE_96_length_748272_cov_42.517338_168317_169657	168317	2E+05	+	Na(+)-translocating NADH-quinone reductase subunit F (EC 1.6.5.-)	FIG00001373	isu;Na(+)-translocating_NADH-quinone_oxidoreductase_and_rnf-like_group_of_electron_transport_complexes
NODE_96_length_748272_cov_42.517338	fig|6666666.34159.peg.2221	peg	NODE_96_length_748272_cov_42.517338_169754_171388	169754	2E+05	+	NAD synthetase (EC 6.3.1.5) / Glutamine amidotransferase chain of NAD synthetase	FIG00000914	isu;NAD_and_NADP_cofactor_biosynthesis_global<br>isu;NAD_and_NADP_cofactor_biosynthesis_glo[illegible]
NODE_96_length_748272_cov_42.517338	fig|6666666.34159.peg.2222	peg	NODE_96_length_748272_cov_42.517338_172089_171394	172089	2E+05	-	hypothetical protein		
NODE_96_length_748272_cov_42.517338	fig|6666666.34159.peg.2223	peg	NODE_96_length_748272_cov_42.517338_175538_172086	175538	2E+05	-	Penicillin-binding protein PBP2	FIG00138520	ff
NODE_96_length_748272_cov_42.517338	fig|6666666.34159.peg.2224	peg	NODE_96_length_748272_cov_42.517338_175689_177560	175689	2E+05	+	Biosynthetic arginine decarboxylase (EC 4.1.1.19)	FIG00002695	isu;Polyamine_Metabolism
NODE_96_length_748272_cov_42.517338	fig|6666666.34159.peg.2225	peg	NODE_96_length_748272_cov_42.517338_177576_178475	177576	2E+05	+	Agmatinase (EC 3.5.3.11)	FIG00052802	icw(1);Polyamine_Metabolism
NODE_96_length_748272_cov_42.517338	fig|6666666.34159.peg.2226	peg	NODE_96_length_748272_cov_42.517338_178479_179444	178479	2E+05	+	Deoxyhypusine synthase (EC 2.5.1.46)	FIG00026855	isu;Alkaloid_biosynthesis_from_L-lysine<br>isu;Modification_of_eukaryotic_initiation_factor_5A
NODE_96_length_748272_cov_42.517338	fig|6666666.34159.peg.2227	peg	NODE_96_length_748272_cov_42.517338_179541_180281	179541	2E+05	+	hypothetical protein	FIG00638284	ff
NODE_96_length_748272_cov_42.517338	fig|6666666.34159.peg.2228	peg	NODE_96_length_748272_cov_42.517338_180494_180619	180494	2E+05	+	hypothetical protein		
NODE_96_length_748272_cov_42.517338	fig|6666666.34159.peg.2229	peg	NODE_96_length_748272_cov_42.517338_180852_180616	180852	2E+05	-	hypothetical protein		
NODE_96_length_748272_cov_42.517338	fig|6666666.34159.peg.2230	peg	NODE_96_length_748272_cov_42.517338_181680_181219	181680	2E+05	-	hypothetical protein		
NODE_96_length_748272_cov_42.517338	fig|6666666.34159.peg.2231	peg	NODE_96_length_748272_cov_42.517338_182059_181793	182059	2E+05	-	hypothetical protein		
NODE_96_length_748272_cov_42.517338	fig|6666666.34159.peg.2232	peg	NODE_96_length_748272_cov_42.517338_182964_182056	182964	2E+05	-	Phosphatidylserine decarboxylase (EC 4.1.1.65)	FIG00001264	ff
NODE_96_length_748272_cov_42.517338	fig|6666666.34159.peg.2233	peg	NODE_96_length_748272_cov_42.517338_183442_182966	183442	2E+05	-	hypothetical protein		
NODE_96_length_748272_cov_42.517338	fig|6666666.34159.peg.2234	peg	NODE_96_length_748272_cov_42.517338_184286_183447	184286	2E+05	-	Translation elongation factor Ts	FIG00000191	isu;Translation_elongation_factors_bacterial<br>icw(1);Ribosome_recycling_related_cluster<br>icw(1);CBSS-312309.3.peg.1965
NODE_96_length_748272_cov_42.517338	fig|6666666.34159.peg.2235	peg	NODE_96_length_748272_cov_42.517338_185096_184290	185096	2E+05	-	SSU ribosomal protein S2p (SAe)	FIG00037273	isu;Ribosome_recycling_related_cluster<br>isu;CBSS-312309.3.peg.1965
NODE_96_length_748272_cov_42.517338	fig|6666666.34159.rna.36	rna	NODE_96_length_748272_cov_42.517338_185259_185189	185259	2E+05	-	tRNA-Gly-TCC		
NODE_96_length_748272_cov_42.517338	fig|6666666.34159.peg.2236	peg	NODE_96_length_748272_cov_42.517338_187294_185327	187294	2E+05	-	hypothetical protein	FIG00638284	ff
NODE_96_length_748272_cov_42.517338	fig|6666666.34159.peg.2237	peg	NODE_96_length_748272_cov_42.517338_189071_187440	189071	2E+05	-	hypothetical protein		
NODE_96_length_748272_cov_42.517338	fig|6666666.34159.peg.2238	peg	NODE_96_length_748272_cov_42.517338_189372_190142	189372	2E+05	+	hypothetical protein		
NODE_96_length_748272_cov_42.517338	fig|6666666.34159.peg.2239	peg	NODE_96_length_748272_cov_42.517338_190545_190204	190545	2E+05	-	hypothetical protein		
NODE_96_length_748272_cov_42.517338	fig|6666666.34159.peg.2240	peg	NODE_96_length_748272_cov_42.517338_192073_190574	192073	2E+05	-	N-acetyl-mannosamine transferase		
NODE_96_length_748272_cov_42.517338	fig|6666666.34159.peg.2241	peg	NODE_96_length_748272_cov_42.517338_194060_192207	194060	2E+05	-	putative sulfate transport protein		
NODE_96_length_748272_cov_42.517338	fig|6666666.34159.peg.2242	peg	NODE_96_length_748272_cov_42.517338_195593_194493	195593	2E+05	-	hypothetical protein		
NODE_96_length_748272_cov_42.517338	fig|6666666.34159.peg.2243	peg	NODE_96_length_748272_cov_42.517338_196347_195664	196347	2E+05	-	hypothetical protein		
NODE_96_length_748272_cov_42.517338	fig|6666666.34159.peg.2244	peg	NODE_96_length_748272_cov_42.517338_196450_196337	196450	2E+05	-	hypothetical protein		
NODE_96_length_748272_cov_42.517338	fig|6666666.34159.peg.2245	peg	NODE_96_length_748272_cov_42.517338_196772_196452	196772	2E+05	-	Archaea-specific enzyme related to ProFAR isomerase (HisA) and containing an additional uncharacterized domain		
NODE_96_length_748272_cov_42.517338	fig|6666666.34159.peg.2246	peg	NODE_96_length_748272_cov_42.517338_197282_196785	197282	2E+05	-	hypothetical protein		
NODE_96_length_748272_cov_42.517338	fig|6666666.34159.peg.2247	peg	NODE_96_length_748272_cov_42.517338_197579_197439	197579	2E+05	-	hypothetical protein		
NODE_96_length_748272_cov_42.517338	fig|6666666.34159.peg.2248	peg	NODE_96_length_748272_cov_42.517338_197531_197758	197531	2E+05	+	hypothetical protein		
NODE_96_length_748272_cov_42.517338	fig|6666666.34159.peg.2249	peg	NODE_96_length_748272_cov_42.517338_198130_199596	198130	2E+05	+	GTP-binding protein EngA	FIG00129616	isu;CBSS-290633.1.peg.1906
NODE_96_length_748272_cov_42.517338	fig|6666666.34159.peg.2250	peg	NODE_96_length_748272_cov_42.517338_200184_200035	200184	2E+05	-	hypothetical protein		
NODE_96_length_748272_cov_42.517338	fig|6666666.34159.peg.2251	peg	NODE_96_length_748272_cov_42.517338_200331_200654	200331	2E+05	+	hypothetical protein		
NODE_96_length_748272_cov_42.517338	fig|6666666.34159.peg.2252	peg	NODE_96_length_748272_cov_42.517338_201005_200805	201005	2E+05	-	putative short-chain dehydrogenase		
NODE_96_length_748272_cov_42.517338	fig|6666666.34159.peg.2253	peg	NODE_96_length_748272_cov_42.517338_201247_201131	201247	2E+05	-	hypothetical protein		
NODE_96_length_748272_cov_42.517338	fig|6666666.34159.peg.2254	peg	NODE_96_length_748272_cov_42.517338_201420_201268	201420	2E+05	-	hypothetical protein		
NODE_96_length_748272_cov_42.517338	fig|6666666.34159.peg.2255	peg	NODE_96_length_748272_cov_42.517338_205228_201524	205228	2E+05	-	hypothetical protein		
NODE_96_length_748272_cov_42.517338	fig|6666666.34159.peg.2256	peg	NODE_96_length_748272_cov_42.517338_205352_206551	205352	2E+05	+	putative tyrosine/tryptophan transport protein		
NODE_96_length_748272_cov_42.517338	fig|6666666.34159.peg.2257	peg	NODE_96_length_748272_cov_42.517338_206738_208009	206738	2E+05	+	Poly(A) polymerase (EC 2.7.7.19)	FIG00000948	isu;Polyadenylation_bacterial
NODE_96_length_748272_cov_42.517338	fig|6666666.34159.peg.2258	peg	NODE_96_length_748272_cov_42.517338_207993_208760	207993	2E+05	+	unknown protein	FIG00764722	ff
NODE_96_length_748272_cov_42.517338	fig|6666666.34159.peg.2259	peg	NODE_96_length_748272_cov_42.517338_208826_209545	208826	2E+05	+	putative dolichol-phosphate mannosyltransferase	FIG00899471	ff
NODE_96_length_748272_cov_42.517338	fig|6666666.34159.peg.2260	peg	NODE_96_length_748272_cov_42.517338_209542_210231	209542	2E+05	+	Lipid-A-disaccharide synthase (EC 2.4.1.182)	FIG00000611	icw(1);Llipid_A_biosynthesis_cluster<br>icw(1);KDO2-Lipid_A_biosynthesis
NODE_96_length_748272_cov_42.517338	fig|6666666.34159.peg.2261	peg	NODE_96_length_748272_cov_42.517338_210228_211400	210228	2E+05	+	Lipid-A-disaccharide synthase (EC 2.4.1.182)	FIG00000611	icw(1);Llipid_A_biosynthesis_cluster<br>icw(1);KDO2-Lipid_A_biosynthesis
NODE_96_length_748272_cov_42.517338	fig|6666666.34159.peg.2262	peg	NODE_96_length_748272_cov_42.517338_211397_212098	211397	2E+05	+	FIG00899414: hypothetical protein	FIG00899413	ff
NODE_96_length_748272_cov_42.517338	fig|6666666.34159.peg.2263	peg	NODE_96_length_748272_cov_42.517338_212538_212227	212538	2E+05	-	hypothetical protein	FIG00638284	ff
NODE_96_length_748272_cov_42.517338	fig|6666666.34159.peg.2264	peg	NODE_96_length_748272_cov_42.517338_212747_213010	212747	2E+05	+	hypothetical protein		
NODE_96_length_748272_cov_42.517338	fig|6666666.34159.peg.2265	peg	NODE_96_length_748272_cov_42.517338_214069_213098	214069	2E+05	-	O-succinylbenzoate synthase (EC 4.2.1.113)	FIG01306622	isu;Muconate_lactonizing_enzyme_family<br>isu;Menaquinone_and_Phylloquinone_Biosynthes[illegible]

NODE_96_length_748272_cov_42.517338	fig|6666666.34159.peg.2266	peg	NODE_96_length_748272_cov_42.517338_215334_214159	215334	2E+05	-	hypothetical protein	FIG00638284	ff
NODE_96_length_748272_cov_42.517338	fig|6666666.34159.peg.2267	peg	NODE_96_length_748272_cov_42.517338_215671_215390	215671	2E+05	-	fatty acid/phospholipid synthesis protein		
NODE_96_length_748272_cov_42.517338	fig|6666666.34159.peg.2268	peg	NODE_96_length_748272_cov_42.517338_216005_215754	216005	2E+05	-	fatty acid/phospholipid synthesis protein		
NODE_96_length_748272_cov_42.517338	fig|6666666.34159.peg.2269	peg	NODE_96_length_748272_cov_42.517338_216488_216703	216488	2E+05	+	hypothetical protein		
NODE_96_length_748272_cov_42.517338	fig|6666666.34159.peg.2270	peg	NODE_96_length_748272_cov_42.517338_217408_217611	217408	2E+05	+	hypothetical protein		
NODE_96_length_748272_cov_42.517338	fig|6666666.34159.peg.2271	peg	NODE_96_length_748272_cov_42.517338_217641_217892	217641	2E+05	+	hypothetical protein		
NODE_96_length_748272_cov_42.517338	fig|6666666.34159.peg.2272	peg	NODE_96_length_748272_cov_42.517338_217930_218055	217930	2E+05	+	hypothetical protein		
NODE_96_length_748272_cov_42.517338	fig|6666666.34159.peg.2273	peg	NODE_96_length_748272_cov_42.517338_219387_218116	219387	2E+05	-	hypothetical protein		
NODE_96_length_748272_cov_42.517338	fig|6666666.34159.peg.2274	peg	NODE_96_length_748272_cov_42.517338_220445_219432	220445	2E+05	-	Phosphate:acyl-ACP acyltransferase PlsX	FIG00000446	ff
NODE_96_length_748272_cov_42.517338	fig|6666666.34159.peg.2275	peg	NODE_96_length_748272_cov_42.517338_221245_220772	221245	2E+05	-	hypothetical protein		
NODE_96_length_748272_cov_42.517338	fig|6666666.34159.peg.2276	peg	NODE_96_length_748272_cov_42.517338_222622_221360	222622	2E+05	-	hypothetical protein		
NODE_96_length_748272_cov_42.517338	fig|6666666.34159.peg.2277	peg	NODE_96_length_748272_cov_42.517338_222686_223069	222686	2E+05	+	hypothetical protein		
NODE_96_length_748272_cov_42.517338	fig|6666666.34159.peg.2278	peg	NODE_96_length_748272_cov_42.517338_223136_223282	223136	2E+05	+	hypothetical protein		
NODE_96_length_748272_cov_42.517338	fig|6666666.34159.peg.2279	peg	NODE_96_length_748272_cov_42.517338_223437_224975	223437	2E+05	+	Cytoplasmic axial filament protein CafA and Ribonuclease G (EC 3.1.4.-)	FIG00059426	isu;RNA_processing_and_degradation,_bacterial
NODE_96_length_748272_cov_42.517338	fig|6666666.34159.peg.2280	peg	NODE_96_length_748272_cov_42.517338_224984_225991	224984	2E+05	+	Glycerol-3-phosphate acyltransferase (EC 2.3.1.15)	FIG00001327	ff
NODE_96_length_748272_cov_42.517338	fig|6666666.34159.peg.2281	peg	NODE_96_length_748272_cov_42.517338_226001_227392	226001	2E+05	+	Oxidoreductase (EC 1.1.1.-)		
NODE_96_length_748272_cov_42.517338	fig|6666666.34159.peg.2282	peg	NODE_96_length_748272_cov_42.517338_227478_227942	227478	2E+05	+	hypothetical protein		
NODE_96_length_748272_cov_42.517338	fig|6666666.34159.peg.2283	peg	NODE_96_length_748272_cov_42.517338_227962_229005	227962	2E+05	+	hypothetical protein		
NODE_96_length_748272_cov_42.517338	fig|6666666.34159.peg.2284	peg	NODE_96_length_748272_cov_42.517338_229262_229924	229262	2E+05	+	hypothetical protein		
NODE_96_length_748272_cov_42.517338	fig|6666666.34159.peg.2285	peg	NODE_96_length_748272_cov_42.517338_231317_230001	231317	2E+05	-	hypothetical protein		
NODE_96_length_748272_cov_42.517338	fig|6666666.34159.peg.2286	peg	NODE_96_length_748272_cov_42.517338_232673_231525	232673	2E+05	-	hypothetical protein		
NODE_96_length_748272_cov_42.517338	fig|6666666.34159.peg.2287	peg	NODE_96_length_748272_cov_42.517338_232797_232642	232797	2E+05	-	hypothetical protein		
NODE_96_length_748272_cov_42.517338	fig|6666666.34159.peg.2288	peg	NODE_96_length_748272_cov_42.517338_233286_232873	233286	2E+05	-	short chain dehydrogenase(EC:1.1.1.-)		
NODE_96_length_748272_cov_42.517338	fig|6666666.34159.rna.37	rna	NODE_96_length_748272_cov_42.517338_233539_233611	233539	2E+05	+	tRNA-Ala-GGC		isu;tRNAs
NODE_96_length_748272_cov_42.517338	fig|6666666.34159.peg.2289	peg	NODE_96_length_748272_cov_42.517338_234292_233612	234292	2E+05	-	unknown protein	FIG00764722	ff
NODE_96_length_748272_cov_42.517338	fig|6666666.34159.peg.2290	peg	NODE_96_length_748272_cov_42.517338_234885_234292	234885	2E+05	-	Nucleoside 5-triphosphatase RdgB (dHAPTP, dITP, XTP-specific) (EC 3.6.1.15)	FIG00000584	isu;Heat_shock_dnaK_gene_cluster_extended isu;Housecleaning_nucleoside_triphosphate_pyrophosphatases
NODE_96_length_748272_cov_42.517338	fig|6666666.34159.peg.2291	peg	NODE_96_length_748272_cov_42.517338_237203_235035	237203	2E+05	-	Transcription elongation factor GreA	FIG00000288	isu;Transcription_factors_bacterial
NODE_96_length_748272_cov_42.517338	fig|6666666.34159.peg.2292	peg	NODE_96_length_748272_cov_42.517338_237785_238231	237785	2E+05	+	hypothetical protein		
NODE_96_length_748272_cov_42.517338	fig|6666666.34159.peg.2293	peg	NODE_96_length_748272_cov_42.517338_238272_239441	238272	2E+05	+	hypothetical protein	FIG00638284	ff
NODE_96_length_748272_cov_42.517338	fig|6666666.34159.peg.2294	peg	NODE_96_length_748272_cov_42.517338_239449_240588	239449	2E+05	+	hypothetical protein		
NODE_96_length_748272_cov_42.517338	fig|6666666.34159.peg.2295	peg	NODE_96_length_748272_cov_42.517338_240621_241277	240621	2E+05	+	Protein-L-isoaspartate O-methyltransferase (EC 2.1.1.77)	FIG00069725	isu;Protein-L-isoaspartate_O-methyltransferase isu;Ton_and_Tol_transport_systems isu;Lanthionine_Synthetases
NODE_96_length_748272_cov_42.517338	fig|6666666.34159.peg.2296	peg	NODE_96_length_748272_cov_42.517338_243106_241271	243106	2E+05	-	hypothetical protein	FIG00638284	ff
NODE_96_length_748272_cov_42.517338	fig|6666666.34159.peg.2297	peg	NODE_96_length_748272_cov_42.517338_243378_245240	243378	2E+05	+	DNA mismatch repair protein MutL	FIG00000380	isu;DNA_repair,_bacterial_MutL-MutS_system
NODE_96_length_748272_cov_42.517338	fig|6666666.34159.peg.2298	peg	NODE_96_length_748272_cov_42.517338_245269_246351	245269	2E+05	+	Aminopeptidase YpdF (MP-, MA-, MS-, AP-, NP- specific)	FIG00135469	idu(1);Protein_degradation
NODE_96_length_748272_cov_42.517338	fig|6666666.34159.peg.2299	peg	NODE_96_length_748272_cov_42.517338_246355_248118	246355	2E+05	+	Phosphate regulon sensor protein PhoR (SphS) (EC 2.7.13.3)	FIG00073301	isu;High_affinity_phosphate_transporter_and_control_of_PHO_regulon isu;PhoR-PhoB_two-component_regulatory_system
NODE_96_length_748272_cov_42.517338	fig|6666666.34159.peg.2300	peg	NODE_96_length_748272_cov_42.517338_249728_248106	249728	2E+05	-	hypothetical protein		
NODE_96_length_748272_cov_42.517338	fig|6666666.34159.peg.2301	peg	NODE_96_length_748272_cov_42.517338_250308_249706	250308	2E+05	-	hypothetical protein		
NODE_96_length_748272_cov_42.517338	fig|6666666.34159.peg.2302	peg	NODE_96_length_748272_cov_42.517338_250961_250524	250961	3E+05	-	CHLPS Euo Protein	FIG00493288	ff
NODE_96_length_748272_cov_42.517338	fig|6666666.34159.peg.2303	peg	NODE_96_length_748272_cov_42.517338_252050_251622	252050	3E+05	-	hypothetical protein		
NODE_96_length_748272_cov_42.517338	fig|6666666.34159.peg.2304	peg	NODE_96_length_748272_cov_42.517338_252605_252183	252605	3E+05	-	hypothetical protein		
NODE_96_length_748272_cov_42.517338	fig|6666666.34159.peg.2305	peg	NODE_96_length_748272_cov_42.517338_253203_252757	253203	3E+05	-	hypothetical protein		
NODE_96_length_748272_cov_42.517338	fig|6666666.34159.peg.2306	peg	NODE_96_length_748272_cov_42.517338_253473_255440	253473	3E+05	+	Peptidase, S41 family	FIG01369989	ff
NODE_96_length_748272_cov_42.517338	fig|6666666.34159.peg.2307	peg	NODE_96_length_748272_cov_42.517338_255569_257083	255569	3E+05	+	Glutamyl-tRNA synthetase (EC 6.1.1.17) @ Glutamyl-tRNA(Gln) synthetase (EC 6.1.1.24)	FIG00000686	isu;Heme_and_Siroheme_Biosynthesis isu;tRNA_aminoacylation,_Glu_and_Gln isu;tRNA_aminoacylation,_Glu_and_Gln
NODE_96_length_748272_cov_42.517338	fig|6666666.34159.peg.2308	peg	NODE_96_length_748272_cov_42.517338_257110_257730	257110	3E+05	+	dedA protein		
NODE_96_length_748272_cov_42.517338	fig|6666666.34159.peg.2309	peg	NODE_96_length_748272_cov_42.517338_257794_259662	257794	3E+05	+	Lead, cadmium, zinc and mercury transporting ATPase (EC 3.6.3.3) (EC 3.6.3.5); Copper-translocating P-type ATPase (EC 3.6.3.4)	FIG00658111	idu(3);Copper_Transport_System idu(3);CBSS-196620.1.peg.2477 idu(3);Copper_homeostasis
NODE_96_length_748272_cov_42.517338	fig|6666666.34159.peg.2310	peg	NODE_96_length_748272_cov_42.517338_259823_260326	259823	3E+05	+	putative Phosphatidylglycerophosphate synthase (= CDP-diacylglycerol-glycerol-3-phosphate 3-phosphatidyltransferase)		
NODE_96_length_748272_cov_42.517338	fig|6666666.34159.peg.2311	peg	NODE_96_length_748272_cov_42.517338_263085_260323	263085	3E+05	-	Chaperone protein DnaK	FIG00023369	idu(1);Heat_shock_dnaK_gene_cluster_extended idu(1);Protein_chaperones
NODE_96_length_748272_cov_42.517338	fig|6666666.34159.peg.2312	peg	NODE_96_length_748272_cov_42.517338_264208_263096	264208	3E+05	-	Alanine dehydrogenase (EC 1.4.1.1)	FIG00000895	icw(1);Pyruvate_Alanine_Serine_Interconversions
NODE_96_length_748272_cov_42.517338	fig|6666666.34159.peg.2313	peg	NODE_96_length_748272_cov_42.517338_264664_264777	264664	3E+05	+	hypothetical protein		
NODE_96_length_748272_cov_42.517338	fig|6666666.34159.peg.2314	peg	NODE_96_length_748272_cov_42.517338_265116_264931	265116	3E+05	-	hypothetical protein		
NODE_96_length_748272_cov_42.517338	fig|6666666.34159.peg.2315	peg	NODE_96_length_748272_cov_42.517338_265318_265097	265318	3E+05	-	Alanine dehydrogenase (EC 1.4.1.1)	FIG00000895	icw(1);Pyruvate_Alanine_Serine_Interconversions
NODE_96_length_748272_cov_42.517338	fig|6666666.34159.peg.2316	peg	NODE_96_length_748272_cov_42.517338_266140_266949	266140	3E+05	+	hypothetical protein		
NODE_96_length_748272_cov_42.517338	fig|6666666.34159.peg.2317	peg	NODE_96_length_748272_cov_42.517338_267397_267212	267397	3E+05	-	hypothetical protein		
NODE_96_length_748272_cov_42.517338	fig|6666666.34159.peg.2318	peg	NODE_96_length_748272_cov_42.517338_271099_268142	271099	3E+05	-	Lanthionine biosynthesis protein LanB		icw(1);Lanthionine_Synthetases
NODE_96_length_748272_cov_42.517338	fig|6666666.34159.peg.2319	peg	NODE_96_length_748272_cov_42.517338_272522_271170	272522	3E+05	-	Lanthionine biosynthesis cyclase LanC	FIG01231877	isu;Lanthionine_Synthetases
NODE_96_length_748272_cov_42.517338	fig|6666666.34159.peg.2320	peg	NODE_96_length_748272_cov_42.517338_272795_272562	272795	3E+05	-	hypothetical protein		
NODE_96_length_748272_cov_42.517338	fig|6666666.34159.peg.2321	peg	NODE_96_length_748272_cov_42.517338_273368_274423	273368	3E+05	+	hypothetical protein		
NODE_96_length_748272_cov_42.517338	fig|6666666.34159.peg.2322	peg	NODE_96_length_748272_cov_42.517338_274483_275526	274483	3E+05	+	hypothetical protein		
NODE_96_length_748272_cov_42.517338	fig|6666666.34159.peg.2323	peg	NODE_96_length_748272_cov_42.517338_275549_276160	275549	3E+05	+	DNA-3-methyladenine glycosylase (EC 3.2.2.20)	FIG00000673	isu;DNA_Repair_Base_Excision isu;CBSS-326442.4.peg.1852

NODE_96_length_748272_cov_42.517338	fig|6666666.34159.peg.2324	peg	NODE_96_length_748272_cov_42.517338_277491_276145	277491	3E+05	-	hypothetical protein		
NODE_96_length_748272_cov_42.517338	fig|6666666.34159.peg.2325	peg	NODE_96_length_748272_cov_42.517338_277721_277608	277721	3E+05	-	hypothetical protein		
NODE_96_length_748272_cov_42.517338	fig|6666666.34159.peg.2326	peg	NODE_96_length_748272_cov_42.517338_277881_278393	277881	3E+05	+	hypothetical protein		
NODE_96_length_748272_cov_42.517338	fig|6666666.34159.peg.2327	peg	NODE_96_length_748272_cov_42.517338_278529_278407	278529	3E+05	-	hypothetical protein		
NODE_96_length_748272_cov_42.517338	fig|6666666.34159.peg.2328	peg	NODE_96_length_748272_cov_42.517338_278552_279706	278552	3E+05	+	hypothetical protein		
NODE_96_length_748272_cov_42.517338	fig|6666666.34159.peg.2329	peg	NODE_96_length_748272_cov_42.517338_279813_280946	279813	3E+05	+	hypothetical protein	FIG00638284	ff
NODE_96_length_748272_cov_42.517338	fig|6666666.34159.peg.2330	peg	NODE_96_length_748272_cov_42.517338_284480_280986	284480	3E+05	-	Chromosome partition protein smc	FIG00026918	isu;DNA_structural_proteins,_bacterial
NODE_96_length_748272_cov_42.517338	fig|6666666.34159.peg.2331	peg	NODE_96_length_748272_cov_42.517338_286153_284498	286153	3E+05	-	Sulfate permease	FIG00005216	isu;Cysteine_Biosynthesis
NODE_96_length_748272_cov_42.517338	fig|6666666.34159.peg.2332	peg	NODE_96_length_748272_cov_42.517338_286351_286560	286351	3E+05	+	hypothetical protein		
NODE_96_length_748272_cov_42.517338	fig|6666666.34159.peg.2333	peg	NODE_96_length_748272_cov_42.517338_286593_286715	286593	3E+05	+	hypothetical protein		
NODE_96_length_748272_cov_42.517338	fig|6666666.34159.peg.2334	peg	NODE_96_length_748272_cov_42.517338_286735_287472	286735	3E+05	+	hypothetical protein		
NODE_96_length_748272_cov_42.517338	fig|6666666.34159.peg.2335	peg	NODE_96_length_748272_cov_42.517338_290040_287491	290040	3E+05	-	hypothetical protein	FIG00638284	ff
NODE_96_length_748272_cov_42.517338	fig|6666666.34159.peg.2336	peg	NODE_96_length_748272_cov_42.517338_291910_290129	291910	3E+05	-	Single-stranded-DNA-specific exonuclease RecJ (EC 3.1.-.-)	FIG00060517	isu;DNA_Repair_Base_Excision
NODE_96_length_748272_cov_42.517338	fig|6666666.34159.peg.2337	peg	NODE_96_length_748272_cov_42.517338_294354_292201	294354	3E+05	-	hypothetical protein		
NODE_96_length_748272_cov_42.517338	fig|6666666.34159.peg.2338	peg	NODE_96_length_748272_cov_42.517338_298041_294592	298041	3E+05	-	hypothetical protein	FIG00638284	ff
NODE_96_length_748272_cov_42.517338	fig|6666666.34159.peg.2339	peg	NODE_96_length_748272_cov_42.517338_302961_298399	302961	3E+05	-	Protein-export membrane protein SecD (TC 3.A.5.1.1) / Protein-export membrane protein SecF (TC 3.A.5.1.1)	FIG00229272	isu;CBSS-211586.1.peg.2832 isu;CBSS-211586.1.peg.2832
NODE_96_length_748272_cov_42.517338	fig|6666666.34159.peg.2340	peg	NODE_96_length_748272_cov_42.517338_303680_303090	303680	3E+05	-	16S rRNA (guanine(966)-N(2))-methyltransferase (EC 2.1.1.171) ## SSU rRNA m(2)G966		isu;RNA_methylation
NODE_96_length_748272_cov_42.517338	fig|6666666.34159.peg.2341	peg	NODE_96_length_748272_cov_42.517338_304903_303683	304903	3E+05	-	hypothetical protein		
NODE_96_length_748272_cov_42.517338	fig|6666666.34159.peg.2342	peg	NODE_96_length_748272_cov_42.517338_306187_305027	306187	3E+05	-	hypothetical protein	FIG00638284	ff
NODE_96_length_748272_cov_42.517338	fig|6666666.34159.peg.2343	peg	NODE_96_length_748272_cov_42.517338_306905_306174	306905	3E+05	-	hypothetical protein		
NODE_96_length_748272_cov_42.517338	fig|6666666.34159.peg.2344	peg	NODE_96_length_748272_cov_42.517338_307826_306993	307826	3E+05	-	Coproporphyrinogen III oxidase, aerobic (EC 1.3.3.3)	FIG00000778	isu;Heme_and_Siroheme_Biosynthesis
NODE_96_length_748272_cov_42.517338	fig|6666666.34159.peg.2345	peg	NODE_96_length_748272_cov_42.517338_308564_307848	308564	3E+05	-	GTP cyclohydrolase I (EC 3.5.4.16) type 1	FIG00000226	idu(1);Folate_Biosynthesis idu(1);tRNA_modification_Bacteria [illegible]
NODE_96_length_748272_cov_42.517338	fig|6666666.34159.peg.2346	peg	NODE_96_length_748272_cov_42.517338_309312_308551	309312	3E+05	-	putative tRNA/rRNA methyltransferase(EC:2.1.1.-)		
NODE_96_length_748272_cov_42.517338	fig|6666666.34159.peg.2347	peg	NODE_96_length_748272_cov_42.517338_310920_309325	310920	3E+05	-	ABC transporter ATP-binding protein uup	FIG00007941	ff
NODE_96_length_748272_cov_42.517338	fig|6666666.34159.peg.2348	peg	NODE_96_length_748272_cov_42.517338_311074_310961	311074	3E+05	-	hypothetical protein		
NODE_96_length_748272_cov_42.517338	fig|6666666.34159.peg.2349	peg	NODE_96_length_748272_cov_42.517338_311180_311049	311180	3E+05	-	hypothetical protein		
NODE_96_length_748272_cov_42.517338	fig|6666666.34159.peg.2350	peg	NODE_96_length_748272_cov_42.517338_311311_312384	311311	3E+05	+	hypothetical protein	FIG00638284	ff
NODE_96_length_748272_cov_42.517338	fig|6666666.34159.peg.2351	peg	NODE_96_length_748272_cov_42.517338_312662_313210	312662	3E+05	+	hypothetical protein		
NODE_96_length_748272_cov_42.517338	fig|6666666.34159.peg.2352	peg	NODE_96_length_748272_cov_42.517338_314757_313285	314757	3E+05	-	hypothetical protein	FIG00638284	ff
NODE_96_length_748272_cov_42.517338	fig|6666666.34159.peg.2353	peg	NODE_96_length_748272_cov_42.517338_314808_314942	314808	3E+05	+	hypothetical protein		
NODE_96_length_748272_cov_42.517338	fig|6666666.34159.peg.2354	peg	NODE_96_length_748272_cov_42.517338_315617_316276	315617	3E+05	+	hypothetical protein		
NODE_96_length_748272_cov_42.517338	fig|6666666.34159.peg.2355	peg	NODE_96_length_748272_cov_42.517338_316484_316320	316484	3E+05	-	hypothetical protein		
NODE_96_length_748272_cov_42.517338	fig|6666666.34159.peg.2356	peg	NODE_96_length_748272_cov_42.517338_317473_316484	317473	3E+05	-	Tyrosine recombinase XerC	FIG00600726	isu;CBSS-323850.3.peg.3269
NODE_96_length_748272_cov_42.517338	fig|6666666.34159.peg.2357	peg	NODE_96_length_748272_cov_42.517338_318525_317617	318525	3E+05	-	Ribonuclease Z (EC 3.1.26.11)	FIG00000828	isu;tRNA_processing
NODE_96_length_748272_cov_42.517338	fig|6666666.34159.peg.2358	peg	NODE_96_length_748272_cov_42.517338_318658_319032	318658	3E+05	+	hypothetical protein		
NODE_96_length_748272_cov_42.517338	fig|6666666.34159.peg.2359	peg	NODE_96_length_748272_cov_42.517338_320214_319033	320214	3E+05	-	putative glpG protein	FIG00899503	ff
NODE_96_length_748272_cov_42.517338	fig|6666666.34159.peg.2360	peg	NODE_96_length_748272_cov_42.517338_320722_320225	320722	3E+05	-	ADP-heptose synthase (EC 2.7.-.-) / D-glycero-beta-D-manno-heptose 7-phosphate kinase	FIG00014339	idu(1);LOS_core_oligosaccharide_biosynthesis idu(1);LOS_core_oligosaccharide_biosynthesis
NODE_96_length_748272_cov_42.517338	fig|6666666.34159.peg.2361	peg	NODE_96_length_748272_cov_42.517338_323224_320732	323224	3E+05	-	ATP-dependent protease La (EC 3.4.21.53) Type I	FIG00132617	isu;Proteolysis_in_bacteria,_ATP-dependent
NODE_96_length_748272_cov_42.517338	fig|6666666.34159.peg.2362	peg	NODE_96_length_748272_cov_42.517338_323293_323409	323293	3E+05	+	hypothetical protein		
NODE_96_length_748272_cov_42.517338	fig|6666666.34159.peg.2363	peg	NODE_96_length_748272_cov_42.517338_323611_325905	323611	3E+05	+	hypothetical protein		
NODE_96_length_748272_cov_42.517338	fig|6666666.34159.peg.2364	peg	NODE_96_length_748272_cov_42.517338_325905_327071	325905	3E+05	+	Deoxyguanosinetriphosphate triphosphohydrolase (EC 3.1.5.1)	FIG00023004	ff
NODE_96_length_748272_cov_42.517338	fig|6666666.34159.peg.2365	peg	NODE_96_length_748272_cov_42.517338_327074_327748	327074	3E+05	+	Inactive homolog of metal-dependent proteases, putative molecular chaperone	FIG00000247	ff
NODE_96_length_748272_cov_42.517338	fig|6666666.34159.peg.2366	peg	NODE_96_length_748272_cov_42.517338_328229_327789	328229	3E+05	-	hypothetical protein		
NODE_96_length_748272_cov_42.517338	fig|6666666.34159.peg.2367	peg	NODE_96_length_748272_cov_42.517338_328928_328377	328928	3E+05	-	hypothetical protein		
NODE_96_length_748272_cov_42.517338	fig|6666666.34159.peg.2368	peg	NODE_96_length_748272_cov_42.517338_329047_330210	329047	3E+05	+	SidB		
NODE_96_length_748272_cov_42.517338	fig|6666666.34159.peg.2369	peg	NODE_96_length_748272_cov_42.517338_330219_331331	330219	3E+05	+	SdbA protein, putative substrate of the Dot/Icm system		
NODE_96_length_748272_cov_42.517338	fig|6666666.34159.peg.2370	peg	NODE_96_length_748272_cov_42.517338_332181_331291	332181	3E+05	-	2-hydroxy-3-oxopropionate reductase (EC 1.1.1.60)	FIG00133229	isu;Glycerate_metabolism isu;Allantoin_Utilization isu;D-galactarate,_D-[illegible]
NODE_96_length_748272_cov_42.517338	fig|6666666.34159.peg.2371	peg	NODE_96_length_748272_cov_42.517338_332333_333436	332333	3E+05	+	hypothetical protein	FIG00638284	ff
NODE_96_length_748272_cov_42.517338	fig|6666666.34159.peg.2372	peg	NODE_96_length_748272_cov_42.517338_335790_336254	335790	3E+05	+	Alanyl-tRNA synthetase (EC 6.1.1.7)	FIG00000139	isu;tRNA_aminoacylation,_Ala
NODE_96_length_748272_cov_42.517338	fig|6666666.34159.peg.2373	peg	NODE_96_length_748272_cov_42.517338_336272_339583	336272	3E+05	+	Transcription-repair coupling factor	FIG00067681	isu;Cell_division-ribosomal_stress_proteins_cluster [illegible]
NODE_96_length_748272_cov_42.517338	fig|6666666.34159.peg.2374	peg	NODE_96_length_748272_cov_42.517338_340419_339580	340419	3E+05	-	AMP nucleosidase (EC 3.2.2.4)	FIG00001232	isu;Purine_conversions
NODE_96_length_748272_cov_42.517338	fig|6666666.34159.peg.2375	peg	NODE_96_length_748272_cov_42.517338_340669_340436	340669	3E+05	-	hypothetical protein		
NODE_96_length_748272_cov_42.517338	fig|6666666.34159.peg.2376	peg	NODE_96_length_748272_cov_42.517338_340881_340669	340881	3E+05	-	hypothetical protein		
NODE_96_length_748272_cov_42.517338	fig|6666666.34159.peg.2377	peg	NODE_96_length_748272_cov_42.517338_342293_340980	342293	3E+05	-	Protein RtcB	FIG00000997	ff
NODE_96_length_748272_cov_42.517338	fig|6666666.34159.peg.2378	peg	NODE_96_length_748272_cov_42.517338_342405_342286	342405	3E+05	-	hypothetical protein		
NODE_96_length_748272_cov_42.517338	fig|6666666.34159.peg.2379	peg	NODE_96_length_748272_cov_42.517338_342421_343815	342421	3E+05	+	hypothetical protein		
NODE_96_length_748272_cov_42.517338	fig|6666666.34159.peg.2380	peg	NODE_96_length_748272_cov_42.517338_343917_344432	343917	3E+05	+	Error-prone repair protein UmuD	FIG00001422	isu;DNA_repair,_bacterial_UmuCD_system
NODE_96_length_748272_cov_42.517338	fig|6666666.34159.peg.2381	peg	NODE_96_length_748272_cov_42.517338_344442_345707	344442	3E+05	+	Error-prone, lesion bypass DNA polymerase V (UmuC)	FIG00001358	icw(1);DNA_repair,_bacterial_UmuCD_system
NODE_96_length_748272_cov_42.517338	fig|6666666.34159.peg.2382	peg	NODE_96_length_748272_cov_42.517338_346163_345711	346163	3E+05	-	hypothetical protein		

NODE_96_length_748272_cov_42.517338	fig|6666666.34159.peg.2383	peg	NODE_96_length_748272_cov_42.517338_346458_346174	346458	3E+05	-	hypothetical protein		
NODE_96_length_748272_cov_42.517338	fig|6666666.34159.peg.2384	peg	NODE_96_length_748272_cov_42.517338_347421_346603	347421	3E+05	-	hypothetical protein		
NODE_96_length_748272_cov_42.517338	fig|6666666.34159.peg.2385	peg	NODE_96_length_748272_cov_42.517338_348915_347539	348915	3E+05	-	hypothetical protein		
NODE_96_length_748272_cov_42.517338	fig|6666666.34159.peg.2386	peg	NODE_96_length_748272_cov_42.517338_349011_352469	349011	4E+05	+	hypothetical protein	FIG00638284	ff
NODE_96_length_748272_cov_42.517338	fig|6666666.34159.peg.2387	peg	NODE_96_length_748272_cov_42.517338_352875_352705	352875	4E+05	-	hypothetical protein		
NODE_96_length_748272_cov_42.517338	fig|6666666.34159.peg.2388	peg	NODE_96_length_748272_cov_42.517338_354004_353015	354004	4E+05	-	Alcohol dehydrogenase (EC 1.1.1.1)	FIG01007502	idu(3);Fermentations:_Mixed_acid idu(3);Butanol_Biosynthesis
NODE_96_length_748272_cov_42.517338	fig|6666666.34159.peg.2389	peg	NODE_96_length_748272_cov_42.517338_355439_354009	355439	4E+05	-	Protein RtcB	FIG00000997	ff
NODE_96_length_748272_cov_42.517338	fig|6666666.34159.peg.2390	peg	NODE_96_length_748272_cov_42.517338_356229_355432	356229	4E+05	-	hypothetical protein	FIG00638284	ff
NODE_96_length_748272_cov_42.517338	fig|6666666.34159.peg.2391	peg	NODE_96_length_748272_cov_42.517338_358864_356222	358864	4E+05	-	Membrane alanine aminopeptidase N (EC 3.4.11.2)	FIG00000714	isu;Aminopeptidases_(EC_3.4.11.-)
NODE_96_length_748272_cov_42.517338	fig|6666666.34159.peg.2392	peg	NODE_96_length_748272_cov_42.517338_359865_358882	359865	4E+05	-	PREDICTED: hypothetical protein		
NODE_96_length_748272_cov_42.517338	fig|6666666.34159.peg.2393	peg	NODE_96_length_748272_cov_42.517338_360702_359995	360702	4E+05	-	hypothetical protein		
NODE_96_length_748272_cov_42.517338	fig|6666666.34159.peg.2394	peg	NODE_96_length_748272_cov_42.517338_368891_360912	368891	4E+05	-	hypothetical protein	FIG00638284	ff
NODE_96_length_748272_cov_42.517338	fig|6666666.34159.peg.2395	peg	NODE_96_length_748272_cov_42.517338_370075_369059	370075	4E+05	-	Probable low-affinity inorganic phosphate transporter	FIG00009601	ff
NODE_96_length_748272_cov_42.517338	fig|6666666.34159.peg.2396	peg	NODE_96_length_748272_cov_42.517338_370700_370077	370700	4E+05	-	Phosphate transport regulator (distant homolog of PhoU)		
NODE_96_length_748272_cov_42.517338	fig|6666666.34159.peg.2397	peg	NODE_96_length_748272_cov_42.517338_370886_372652	370886	4E+05	+	Butyryl-CoA dehydrogenase (EC 1.3.99.2)	FIG00009149	ff
NODE_96_length_748272_cov_42.517338	fig|6666666.34159.peg.2398	peg	NODE_96_length_748272_cov_42.517338_372634_374274	372634	4E+05	+	Methylcrotonyl-CoA carboxylase carboxyl transferase subunit (EC 6.4.1.4)	FIG00138249	ff
NODE_96_length_748272_cov_42.517338	fig|6666666.34159.peg.2399	peg	NODE_96_length_748272_cov_42.517338_374945_376231	374945	4E+05	+	hypothetical protein		
NODE_96_length_748272_cov_42.517338	fig|6666666.34159.peg.2400	peg	NODE_96_length_748272_cov_42.517338_377858_376242	377858	4E+05	-	hypothetical protein		
NODE_96_length_748272_cov_42.517338	fig|6666666.34159.peg.2401	peg	NODE_96_length_748272_cov_42.517338_378973_377990	378973	4E+05	-	hypothetical protein		
NODE_96_length_748272_cov_42.517338	fig|6666666.34159.peg.2402	peg	NODE_96_length_748272_cov_42.517338_379297_379151	379297	4E+05	-	hypothetical protein		
NODE_96_length_748272_cov_42.517338	fig|6666666.34159.peg.2403	peg	NODE_96_length_748272_cov_42.517338_379308_379439	379308	4E+05	+	hypothetical protein		
NODE_96_length_748272_cov_42.517338	fig|6666666.34159.peg.2404	peg	NODE_96_length_748272_cov_42.517338_379572_380942	379572	4E+05	+	hypothetical protein		
NODE_96_length_748272_cov_42.517338	fig|6666666.34159.peg.2405	peg	NODE_96_length_748272_cov_42.517338_380930_381280	380930	4E+05	+	hypothetical protein		
NODE_96_length_748272_cov_42.517338	fig|6666666.34159.peg.2406	peg	NODE_96_length_748272_cov_42.517338_381358_382044	381358	4E+05	+	hypothetical protein		
NODE_96_length_748272_cov_42.517338	fig|6666666.34159.peg.2407	peg	NODE_96_length_748272_cov_42.517338_382083_382907	382083	4E+05	+	beta-lactamase-like		
NODE_96_length_748272_cov_42.517338	fig|6666666.34159.peg.2408	peg	NODE_96_length_748272_cov_42.517338_383284_382904	383284	4E+05	-	hypothetical protein		
NODE_96_length_748272_cov_42.517338	fig|6666666.34159.peg.2409	peg	NODE_96_length_748272_cov_42.517338_383445_384773	383445	4E+05	+	Arabinose-proton symporter	FIG00002070	isu;L-Arabinose_utilization
NODE_96_length_748272_cov_42.517338	fig|6666666.34159.peg.2410	peg	NODE_96_length_748272_cov_42.517338_384861_385943	384861	4E+05	+	Branched-chain alpha-keto acid dehydrogenase, E1 component, alpha subunit (EC 1.2.4.4)	FIG00000964	icw(2);Isoleucine_degradation icw(2);Valine_degradation isu;Dehydrogenase_complexes
NODE_96_length_748272_cov_42.517338	fig|6666666.34159.peg.2411	peg	NODE_96_length_748272_cov_42.517338_385948_386925	385948	4E+05	+	Branched-chain alpha-keto acid dehydrogenase, E1 component, beta subunit (EC 1.2.4.4)	FIG00055130	icw(1);Isoleucine_degradation icw(1);Valine_degradation icw(2);Dehydrogenase_complexes
NODE_96_length_748272_cov_42.517338	fig|6666666.34159.peg.2412	peg	NODE_96_length_748272_cov_42.517338_386938_388146	386938	4E+05	+	Dihydrolipoamide acyltransferase component of branched-chain alpha-keto acid dehydrogenase complex (EC 2.3.1.168)	FIG01304883	isu;Isoleucine_degradation isu;Valine_degradation icw(1);Dehydrogenase_complexes
NODE_96_length_748272_cov_42.517338	fig|6666666.34159.peg.2413	peg	NODE_96_length_748272_cov_42.517338_388266_388141	388266	4E+05	-	hypothetical protein		
NODE_96_length_748272_cov_42.517338	fig|6666666.34159.peg.2414	peg	NODE_96_length_748272_cov_42.517338_388473_388294	388473	4E+05	-	hypothetical protein		
NODE_96_length_748272_cov_42.517338	fig|6666666.34159.peg.2415	peg	NODE_96_length_748272_cov_42.517338_388806_388534	388806	4E+05	-	hypothetical protein		
NODE_96_length_748272_cov_42.517338	fig|6666666.34159.peg.2416	peg	NODE_96_length_748272_cov_42.517338_389221_390942	389221	4E+05	+	hypothetical protein	FIG00638284	ff
NODE_96_length_748272_cov_42.517338	fig|6666666.34159.peg.2417	peg	NODE_96_length_748272_cov_42.517338_391036_393069	391036	4E+05	+	hypothetical protein	FIG00638284	ff
NODE_96_length_748272_cov_42.517338	fig|6666666.34159.peg.2418	peg	NODE_96_length_748272_cov_42.517338_393402_394877	393402	4E+05	+	hypothetical protein		
NODE_96_length_748272_cov_42.517338	fig|6666666.34159.peg.2419	peg	NODE_96_length_748272_cov_42.517338_397863_394924	397863	4E+05	-	Protease III precursor (EC 3.4.24.55)	FIG00002671	isu;DNA_repair,_bacterial_RecBCD_pathway
NODE_96_length_748272_cov_42.517338	fig|6666666.34159.peg.2420	peg	NODE_96_length_748272_cov_42.517338_398175_397912	398175	4E+05	-	hypothetical protein		
NODE_96_length_748272_cov_42.517338	fig|6666666.34159.peg.2421	peg	NODE_96_length_748272_cov_42.517338_398509_398318	398509	4E+05	-	hypothetical protein		
NODE_96_length_748272_cov_42.517338	fig|6666666.34159.peg.2422	peg	NODE_96_length_748272_cov_42.517338_398516_398893	398516	4E+05	+	Queuosine biosynthesis QueD, PTPS-I	FIG00138171	icw(1);tRNA_modification_Bacteria idu(1);Queuosine-Archaeosine_Biosynthesis
NODE_96_length_748272_cov_42.517338	fig|6666666.34159.peg.2423	peg	NODE_96_length_748272_cov_42.517338_398905_399591	398905	4E+05	+	GTP cyclohydrolase I (EC 3.5.4.16) type 1	FIG00000226	idu(1);Folate_Biosynthesis idu(1);tRNA_modification_Bacteria icw(1);Queuosine-Archaeosine_Biosynthesis
NODE_96_length_748272_cov_42.517338	fig|6666666.34159.peg.2424	peg	NODE_96_length_748272_cov_42.517338_402314_399663	402314	4E+05	-	putative rapA, a bacterial member of the swi/snf helicase family		
NODE_96_length_748272_cov_42.517338	fig|6666666.34159.peg.2425	peg	NODE_96_length_748272_cov_42.517338_402439_403728	402439	4E+05	+	hypothetical protein		
NODE_96_length_748272_cov_42.517338	fig|6666666.34159.peg.2426	peg	NODE_96_length_748272_cov_42.517338_405566_403725	405566	4E+05	-	hypothetical protein		
NODE_96_length_748272_cov_42.517338	fig|6666666.34159.peg.2427	peg	NODE_96_length_748272_cov_42.517338_407153_405699	407153	4E+05	-	Glycine dehydrogenase [decarboxylating] (glycine cleavage system P2 protein) (EC 1.4.4.2)	FIG00001328	isu;Glycine_and_Serine_Utilization icw(1);Glycine_cleavage_system
NODE_96_length_748272_cov_42.517338	fig|6666666.34159.peg.2428	peg	NODE_96_length_748272_cov_42.517338_408490_407153	408490	4E+05	-	Glycine dehydrogenase [decarboxylating] (glycine cleavage system P1 protein) (EC 1.4.4.2)	FIG00001364	icw(1);Glycine_and_Serine_Utilization icw(2);Glycine_cleavage_system
NODE_96_length_748272_cov_42.517338	fig|6666666.34159.peg.2429	peg	NODE_96_length_748272_cov_42.517338_408880_408503	408880	4E+05	-	Glycine cleavage system H protein	FIG00000296	icw(2);Glycine_and_Serine_Utilization isu;CBSS-315749.4.peg.3658 isu;Glycine_cleavage_system
NODE_96_length_748272_cov_42.517338	fig|6666666.34159.peg.2430	peg	NODE_96_length_748272_cov_42.517338_409920_408877	409920	4E+05	-	Aminomethyltransferase (glycine cleavage system T protein) (EC 2.1.2.10)	FIG00138538	icw(3);Glycine_and_Serine_Utilization isu;CBSS-87626.3.peg.3639 icw(3);Glycine_cleavage_system
NODE_96_length_748272_cov_42.517338	fig|6666666.34159.peg.2431	peg	NODE_96_length_748272_cov_42.517338_410563_409898	410563	4E+05	-	unknown protein	FIG00764722	ff
NODE_96_length_748272_cov_42.517338	fig|6666666.34159.peg.2432	peg	NODE_96_length_748272_cov_42.517338_412990_410573	412990	4E+05	-	hypothetical protein	FIG00638284	ff
NODE_96_length_748272_cov_42.517338	fig|6666666.34159.peg.2433	peg	NODE_96_length_748272_cov_42.517338_413195_414256	413195	4E+05	+	hypothetical protein	FIG00638284	ff
NODE_96_length_748272_cov_42.517338	fig|6666666.34159.peg.2434	peg	NODE_96_length_748272_cov_42.517338_414267_416003	414267	4E+05	+	hypothetical protein		
NODE_96_length_748272_cov_42.517338	fig|6666666.34159.peg.2435	peg	NODE_96_length_748272_cov_42.517338_416075_417112	416075	4E+05	+	putative polysaccharide export protein wza		
NODE_96_length_748272_cov_42.517338	fig|6666666.34159.peg.2436	peg	NODE_96_length_748272_cov_42.517338_417182_417871	417182	4E+05	+	Dihydrodipicolinate reductase (EC 1.3.1.26)	FIG00000347	isu;Lysine_Biosynthesis_DAP_Pathway,_GJO_scratch isu;Lysine_Biosynthesis_DAP_Pathway
NODE_96_length_748272_cov_42.517338	fig|6666666.34159.peg.2437	peg	NODE_96_length_748272_cov_42.517338_417868_418761	417868	4E+05	+	Dihydrodipicolinate synthase (EC 4.2.1.52)	FIG00019600	icw(2);Lysine_Biosynthesis_DAP_Pathway,_GJO_scratch icw(2);Lysine_Biosynthesis_DAP_Pathway
NODE_96_length_748272_cov_42.517338	fig|6666666.34159.peg.2438	peg	NODE_96_length_748272_cov_42.517338_418769_419842	418769	4E+05	+	Aspartate-semialdehyde dehydrogenase (EC 1.2.1.11)	FIG00026137	icw(1);Lysine_Biosynthesis_DAP_Pathway,_GJO_scratch isu;Threonine_and_Homoserine_Biosynthesis
NODE_96_length_748272_cov_42.517338	fig|6666666.34159.peg.2439	peg	NODE_96_length_748272_cov_42.517338_419839_421017	419839	4E+05	+	L,L-diaminopimelate aminotransferase (EC 2.6.1.83)	FIG01288927	icw(3);Lysine_Biosynthesis_DAP_Pathway,_GJO_scratch icw(3);Lysine_Biosynthesis_DAP_Pathway
NODE_96_length_748272_cov_42.517338	fig|6666666.34159.peg.2440	peg	NODE_96_length_748272_cov_42.517338_421651_421046	421651	4E+05	-	hypothetical protein		
NODE_96_length_748272_cov_42.517338	fig|6666666.34159.peg.2441	peg	NODE_96_length_748272_cov_42.517338_422668_421850	422668	4E+05	-	putative dimethyladenosine transferase(EC:2.1.1.-)		

NODE_96_length_748272_cov_42.517338	fig|6666666.34159.peg.2442	peg	NODE_96_length_748272_cov_42.517338_422849_422730	422849	4E+05	-	hypothetical protein		
NODE_96_length_748272_cov_42.517338	fig|6666666.34159.peg.2443	peg	NODE_96_length_748272_cov_42.517338_424381_422993	424381	4E+05	-	hypothetical protein	FIG00638284	ff
NODE_96_length_748272_cov_42.517338	fig|6666666.34159.peg.2444	peg	NODE_96_length_748272_cov_42.517338_426241_424997	426241	4E+05	-	hypothetical protein		
NODE_96_length_748272_cov_42.517338	fig|6666666.34159.peg.2445	peg	NODE_96_length_748272_cov_42.517338_429279_426328	429279	4E+05	-	hypothetical protein	FIG00638284	ff
NODE_96_length_748272_cov_42.517338	fig|6666666.34159.peg.2446	peg	NODE_96_length_748272_cov_42.517338_430337_429291	430337	4E+05	-	hypothetical protein	FIG00638284	ff
NODE_96_length_748272_cov_42.517338	fig|6666666.34159.peg.2447	peg	NODE_96_length_748272_cov_42.517338_431904_430519	431904	4E+05	-	Fibronectin/fibrinogen-binding protein		isu;Adhesion_of_Campylobacter
NODE_96_length_748272_cov_42.517338	fig|6666666.34159.peg.2448	peg	NODE_96_length_748272_cov_42.517338_433060_431894	433060	4E+05	-	S-adenosylmethionine synthetase (EC 2.5.1.6)	FIG00000329	isu;Methionine_Degradation
NODE_96_length_748272_cov_42.517338	fig|6666666.34159.peg.2449	peg	NODE_96_length_748272_cov_42.517338_433151_433591	433151	4E+05	+	hypothetical protein	FIG00638284	ff
NODE_96_length_748272_cov_42.517338	fig|6666666.34159.peg.2450	peg	NODE_96_length_748272_cov_42.517338_435410_433857	435410	4E+05	-	ADP/ATP Translocase, NTT1	FIG01345036	ff
NODE_96_length_748272_cov_42.517338	fig|6666666.34159.peg.2451	peg	NODE_96_length_748272_cov_42.517338_436158_435631	436158	4E+05	-	FIG00493911: hypothetical protein	FIG00493632	ff
NODE_96_length_748272_cov_42.517338	fig|6666666.34159.peg.2452	peg	NODE_96_length_748272_cov_42.517338_436926_437774	436926	4E+05	+	Scaffold protein for [4Fe-4S] cluster assembly ApbC, MRP-like	FIG00001492	isu;Iron-sulfur_cluster_assembly
NODE_96_length_748272_cov_42.517338	fig|6666666.34159.peg.2453	peg	NODE_96_length_748272_cov_42.517338_437771_438097	437771	4E+05	+	FIG00899427: hypothetical protein	FIG00899426	ff
NODE_96_length_748272_cov_42.517338	fig|6666666.34159.peg.2454	peg	NODE_96_length_748272_cov_42.517338_438126_439121	438126	4E+05	+	Manganese ABC transporter, periplasmic-binding protein SitA	FIG00627311	ff
NODE_96_length_748272_cov_42.517338	fig|6666666.34159.peg.2455	peg	NODE_96_length_748272_cov_42.517338_439105_439896	439105	4E+05	+	Manganese ABC transporter, ATP-binding protein SitB	FIG01322879	ff
NODE_96_length_748272_cov_42.517338	fig|6666666.34159.peg.2456	peg	NODE_96_length_748272_cov_42.517338_439906_441249	439906	4E+05	+	Manganese ABC transporter, inner membrane permease protein SitC	FIG00905727	ff
NODE_96_length_748272_cov_42.517338	fig|6666666.34159.peg.2457	peg	NODE_96_length_748272_cov_42.517338_441259_442245	441259	4E+05	+	Manganese ABC transporter, inner membrane permease protein SitD	FIG01046910	ff
NODE_96_length_748272_cov_42.517338	fig|6666666.34159.peg.2458	peg	NODE_96_length_748272_cov_42.517338_442985_442242	442985	4E+05	-	putative secreted lipase		
NODE_96_length_748272_cov_42.517338	fig|6666666.34159.peg.2459	peg	NODE_96_length_748272_cov_42.517338_443258_443551	443258	4E+05	+	Heat shock protein 60 family co-chaperone GroES	FIG00009229	idu(1);GroEL_GroES
NODE_96_length_748272_cov_42.517338	fig|6666666.34159.peg.2460	peg	NODE_96_length_748272_cov_42.517338_443588_445270	443588	4E+05	+	Heat shock protein 60 family chaperone GroEL	FIG00000056	idu(2);Staphylococcal_pathogenicity_islands_SaPI icw(1);GroEL_GroES
NODE_96_length_748272_cov_42.517338	fig|6666666.34159.peg.2461	peg	NODE_96_length_748272_cov_42.517338_445778_446239	445778	4E+05	+	hypothetical protein		
NODE_96_length_748272_cov_42.517338	fig|6666666.34159.peg.2462	peg	NODE_96_length_748272_cov_42.517338_446376_446236	446376	4E+05	-	hypothetical protein		
NODE_96_length_748272_cov_42.517338	fig|6666666.34159.peg.2463	peg	NODE_96_length_748272_cov_42.517338_446975_447163	446975	4E+05	+	hypothetical protein		
NODE_96_length_748272_cov_42.517338	fig|6666666.34159.peg.2464	peg	NODE_96_length_748272_cov_42.517338_447446_447790	447446	4E+05	+	hypothetical protein		
NODE_96_length_748272_cov_42.517338	fig|6666666.34159.peg.2465	peg	NODE_96_length_748272_cov_42.517338_447816_448190	447816	4E+05	+	hypothetical protein		
NODE_96_length_748272_cov_42.517338	fig|6666666.34159.peg.2466	peg	NODE_96_length_748272_cov_42.517338_448334_448753	448334	4E+05	+	hypothetical protein		
NODE_96_length_748272_cov_42.517338	fig|6666666.34159.peg.2467	peg	NODE_96_length_748272_cov_42.517338_450451_448814	450451	4E+05	-	Thymidylate synthase thyX (EC 2.1.1.-)	FIG00001348	isu;Folate_Biosynthesis
NODE_96_length_748272_cov_42.517338	fig|6666666.34159.peg.2468	peg	NODE_96_length_748272_cov_42.517338_451394_450561	451394	5E+05	-	hypothetical protein	FIG00638284	ff
NODE_96_length_748272_cov_42.517338	fig|6666666.34159.peg.2469	peg	NODE_96_length_748272_cov_42.517338_452221_451451	452221	5E+05	-	tRNA pseudouridine synthase A (EC 4.2.1.70)	FIG00000093	idu(1);tRNA_processing idu(1);tRNA_modification_Bacteria [illegible]
NODE_96_length_748272_cov_42.517338	fig|6666666.34159.peg.2470	peg	NODE_96_length_748272_cov_42.517338_452900_452211	452900	5E+05	-	2-C-methyl-D-erythritol 4-phosphate cytidylyltransferase (EC 2.7.7.60)	FIG00074225	isu;Nonmevalonate_Branch_of_Isoprenoid_Biosynthesis isu;Isoprenoid_Biosynthesis [illegible]
NODE_96_length_748272_cov_42.517338	fig|6666666.34159.peg.2471	peg	NODE_96_length_748272_cov_42.517338_453880_452897	453880	5E+05	-	phosphohydrolase	FIG00899411	ff
NODE_96_length_748272_cov_42.517338	fig|6666666.34159.peg.2472	peg	NODE_96_length_748272_cov_42.517338_454306_454013	454306	5E+05	-	SWIB (YM74) complex protein	FIG00899487	ff
NODE_96_length_748272_cov_42.517338	fig|6666666.34159.peg.2473	peg	NODE_96_length_748272_cov_42.517338_454568_456832	454568	5E+05	+	hypothetical protein	FIG00638284	ff
NODE_96_length_748272_cov_42.517338	fig|6666666.34159.peg.2474	peg	NODE_96_length_748272_cov_42.517338_456915_457982	456915	5E+05	+	efflux transporter, RND family, MFP subunit		
NODE_96_length_748272_cov_42.517338	fig|6666666.34159.peg.2475	peg	NODE_96_length_748272_cov_42.517338_457988_461110	457988	5E+05	+	RND multidrug efflux transporter; Acriflavin resistance protein	FIG00132942	isu;Multidrug_Resistance_Efflux_Pumps
NODE_96_length_748272_cov_42.517338	fig|6666666.34159.peg.2476	peg	NODE_96_length_748272_cov_42.517338_461103_462365	461103	5E+05	+	Type I secretion outer membrane protein, TolC precursor	FIG00028132	isu;Ton_and_Tol_transport_systems icw(1);Multidrug_Resistance_Efflux_Pumps
NODE_96_length_748272_cov_42.517338	fig|6666666.34159.peg.2477	peg	NODE_96_length_748272_cov_42.517338_466009_462362	466009	5E+05	-	hypothetical protein	FIG00638284	ff
NODE_96_length_748272_cov_42.517338	fig|6666666.34159.peg.2478	peg	NODE_96_length_748272_cov_42.517338_466486_466184	466486	5E+05	-	hypothetical protein		
NODE_96_length_748272_cov_42.517338	fig|6666666.34159.peg.2479	peg	NODE_96_length_748272_cov_42.517338_466865_466692	466865	5E+05	-	hypothetical protein		
NODE_96_length_748272_cov_42.517338	fig|6666666.34159.peg.2480	peg	NODE_96_length_748272_cov_42.517338_467306_467148	467306	5E+05	-	hypothetical protein		
NODE_96_length_748272_cov_42.517338	fig|6666666.34159.peg.2481	peg	NODE_96_length_748272_cov_42.517338_467545_467384	467545	5E+05	-	hypothetical protein		
NODE_96_length_748272_cov_42.517338	fig|6666666.34159.peg.2482	peg	NODE_96_length_748272_cov_42.517338_468863_467799	468863	5E+05	-	Choloylglycine hydrolase (EC 3.5.1.24)	FIG00009563	isu;Bile_hydrolysis
NODE_96_length_748272_cov_42.517338	fig|6666666.34159.peg.2483	peg	NODE_96_length_748272_cov_42.517338_469018_471894	469018	5E+05	+	Aconitate hydratase (EC 4.2.1.3)	FIG00022996	isu;TCA_Cycle isu;Glyoxylate_bypass
NODE_96_length_748272_cov_42.517338	fig|6666666.34159.peg.2484	peg	NODE_96_length_748272_cov_42.517338_472060_473034	472060	5E+05	+	hypothetical protein		
NODE_96_length_748272_cov_42.517338	fig|6666666.34159.peg.2485	peg	NODE_96_length_748272_cov_42.517338_474036_473029	474036	5E+05	-	hypothetical protein	FIG00638284	ff
NODE_96_length_748272_cov_42.517338	fig|6666666.34159.peg.2486	peg	NODE_96_length_748272_cov_42.517338_475546_474101	475546	5E+05	-	RND efflux system, outer membrane lipoprotein CmeC	FIG00006235	idu(2);Multidrug_Resistance_Efflux_Pumps
NODE_96_length_748272_cov_42.517338	fig|6666666.34159.peg.2487	peg	NODE_96_length_748272_cov_42.517338_476714_475605	476714	5E+05	-	ABC transport system, permease component YbhR	FIG00042275	isu;ATP-dependent_efflux_pump_transporter_Ybh
NODE_96_length_748272_cov_42.517338	fig|6666666.34159.peg.2488	peg	NODE_96_length_748272_cov_42.517338_477864_476716	477864	5E+05	-	ABC transport system, permease component YbhS	FIG00060294	icw(3);ATP-dependent_efflux_pump_transporter_Ybh
NODE_96_length_748272_cov_42.517338	fig|6666666.34159.peg.2489	peg	NODE_96_length_748272_cov_42.517338_479588_477861	479588	5E+05	-	ABC transporter multidrug efflux pump, fused ATP-binding domains	FIG00003387	icw(2);ATP-dependent_efflux_pump_transporter_Ybh
NODE_96_length_748272_cov_42.517338	fig|6666666.34159.peg.2490	peg	NODE_96_length_748272_cov_42.517338_480439_479585	480439	5E+05	-	Predicted membrane fusion protein (MFP) component of efflux pump, membrane anchor protein YbhG	FIG00002351	icw(1);ATP-dependent_efflux_pump_transporter_Ybh
NODE_96_length_748272_cov_42.517338	fig|6666666.34159.peg.2491	peg	NODE_96_length_748272_cov_42.517338_480648_481805	480648	5E+05	+	Sulfite reductase [NADPH] flavoprotein alpha-component (EC 1.8.1.2)	FIG00001318	isu;Cysteine_Biosynthesis isu;Inorganic_Sulfur_Assimilation
NODE_96_length_748272_cov_42.517338	fig|6666666.34159.peg.2492	peg	NODE_96_length_748272_cov_42.517338_482444_482187	482444	5E+05	-	hypothetical protein		
NODE_96_length_748272_cov_42.517338	fig|6666666.34159.peg.2493	peg	NODE_96_length_748272_cov_42.517338_483064_482573	483064	5E+05	-	2-C-methyl-D-erythritol 2,4-cyclodiphosphate synthase (EC 4.6.1.12)	FIG00084105	isu;Nonmevalonate_Branch_of_Isoprenoid_Biosynthesis isu;Isoprenoid_Biosynthesis [illegible]
NODE_96_length_748272_cov_42.517338	fig|6666666.34159.peg.2494	peg	NODE_96_length_748272_cov_42.517338_483364_484767	483364	5E+05	+	UDP-N-acetylglucosamine 1-carboxyvinyltransferase (EC 2.5.1.7)	FIG00034392	isu;UDP-N-acetylmuramate_from_Fructose-6-phosphate_Biosynthesis
NODE_96_length_748272_cov_42.517338	fig|6666666.34159.peg.2495	peg	NODE_96_length_748272_cov_42.517338_484913_485512	484913	5E+05	+	ATP-dependent Clp protease proteolytic subunit (EC 3.4.21.92)	FIG00000028	idu(1);Proteolysis_in_bacteria,_ATP-dependent idu(1);cAMP_signaling_in_bacteria
NODE_96_length_748272_cov_42.517338	fig|6666666.34159.peg.2496	peg	NODE_96_length_748272_cov_42.517338_485518_486318	485518	5E+05	+	Diaminopimelate epimerase (EC 5.1.1.7)	FIG00000421	isu;CBSS-323850.3.peg.3269 isu;CBSS-84588.1.peg.1247 [illegible]
NODE_96_length_748272_cov_42.517338	fig|6666666.34159.peg.2497	peg	NODE_96_length_748272_cov_42.517338_487306_486296	487306	5E+05	-	hypothetical protein		
NODE_96_length_748272_cov_42.517338	fig|6666666.34159.peg.2498	peg	NODE_96_length_748272_cov_42.517338_487489_488676	487489	5E+05	+	FIG047466: hypothetical protein	FIG00493938	ff
NODE_96_length_748272_cov_42.517338	fig|6666666.34159.peg.2499	peg	NODE_96_length_748272_cov_42.517338_488696_489457	488696	5E+05	+	COG1496: Uncharacterized conserved protein	FIG01348339	ff
NODE_96_length_748272_cov_42.517338	fig|6666666.34159.peg.2500	peg	NODE_96_length_748272_cov_42.517338_489495_490358	489495	5E+05	+	hypothetical protein		

NODE_96_length_748272_cov_42.517338	fig|6666666.34159.peg.2501	peg	NODE_96_length_748272_cov_42.517338_490365_491615	490365	5E+05	+	alternate gene name: yzbB	FIG00001850	ff
NODE_96_length_748272_cov_42.517338	fig|6666666.34159.peg.2502	peg	NODE_96_length_748272_cov_42.517338_492475_491618	492475	5E+05	-	Type III secretion inner membrane protein SctT	FIG00899477	ff
NODE_96_length_748272_cov_42.517338	fig|6666666.34159.peg.2503	peg	NODE_96_length_748272_cov_42.517338_492778_492500	492778	5E+05	-	probable type III secretion inner membrane protein SctS	FIG00899497	ff
NODE_96_length_748272_cov_42.517338	fig|6666666.34159.peg.2504	peg	NODE_96_length_748272_cov_42.517338_493758_492799	493758	5E+05	-	Type III secretion inner membrane protein SctR	FIG00899422	ff
NODE_96_length_748272_cov_42.517338	fig|6666666.34159.peg.2505	peg	NODE_96_length_748272_cov_42.517338_494470_493790	494470	5E+05	-	Type III secretion translocase SctL	FIG00899462	ff
NODE_96_length_748272_cov_42.517338	fig|6666666.34159.peg.2506	peg	NODE_96_length_748272_cov_42.517338_495332_494460	495332	5E+05	-	FIG00899422: hypothetical protein	FIG00899417	ff
NODE_96_length_748272_cov_42.517338	fig|6666666.34159.peg.2507	peg	NODE_96_length_748272_cov_42.517338_496322_495357	496322	5E+05	-	Type III secretion protein SctJ	FIG00899443	ff
NODE_96_length_748272_cov_42.517338	fig|6666666.34159.peg.2508	peg	NODE_96_length_748272_cov_42.517338_496578_498128	496578	5E+05	+	hypothetical protein	FIG00638284	ff
NODE_96_length_748272_cov_42.517338	fig|6666666.34159.peg.2509	peg	NODE_96_length_748272_cov_42.517338_498154_498399	498154	5E+05	+	hypothetical protein		
NODE_96_length_748272_cov_42.517338	fig|6666666.34159.peg.2510	peg	NODE_96_length_748272_cov_42.517338_499429_498401	499429	5E+05	-	tRNA dihydrouridine synthase B (EC 1.-.-.-)	FIG00000580	isu;tRNA_modification_Bacteria
NODE_96_length_748272_cov_42.517338	fig|6666666.34159.peg.2511	peg	NODE_96_length_748272_cov_42.517338_499692_499435	499692	5E+05	-	Cell division protein YlmG/Ycf19 (putative), YggT family		
NODE_96_length_748272_cov_42.517338	fig|6666666.34159.peg.2512	peg	NODE_96_length_748272_cov_42.517338_500738_499770	500738	5E+05	-	dTDP-glucose 4,6-dehydratase (EC 4.2.1.46)	FIG00030688	idu(1);dTDP-rhamnose_synthesis idu(1);Rhamnose_containing_glycans idu(1);CBSS-296591.1.peg.2330
NODE_96_length_748272_cov_42.517338	fig|6666666.34159.peg.2513	peg	NODE_96_length_748272_cov_42.517338_504562_500735	504562	5E+05	-	hypothetical protein	FIG00638284	ff
NODE_96_length_748272_cov_42.517338	fig|6666666.34159.peg.2514	peg	NODE_96_length_748272_cov_42.517338_506127_504706	506127	5E+05	-	Para-aminobenzoate synthase, aminase component (EC 2.6.1.85)	FIG00341164	isu;Chorismate:_Intermediate_for_synthesis_of_Tryptophan,_PAPA_antibiotics,_PABA,_3-hydroxyanthranilate_and_more
NODE_96_length_748272_cov_42.517338	fig|6666666.34159.peg.2515	peg	NODE_96_length_748272_cov_42.517338_506857_506120	506857	5E+05	-	FIG000859: hypothetical protein	FIG00000859	ff
NODE_96_length_748272_cov_42.517338	fig|6666666.34159.peg.2516	peg	NODE_96_length_748272_cov_42.517338_507532_506873	507532	5E+05	-	hypothetical protein	FIG00638284	ff
NODE_96_length_748272_cov_42.517338	fig|6666666.34159.peg.2517	peg	NODE_96_length_748272_cov_42.517338_508072_507566	508072	5E+05	-	Amino Group Acetyl Transferase	FIG00493178	ff
NODE_96_length_748272_cov_42.517338	fig|6666666.34159.peg.2518	peg	NODE_96_length_748272_cov_42.517338_509094_508069	509094	5E+05	-	Peptide chain release factor 2; programmed frameshift-containing	FIG00624891	isu;Programmed_frameshift isu;Programmed_frameshift isu;Translation_termination_factors_bacterial
NODE_96_length_748272_cov_42.517338	fig|6666666.34159.peg.2519	peg	NODE_96_length_748272_cov_42.517338_509280_509489	509280	5E+05	+	hypothetical protein		
NODE_96_length_748272_cov_42.517338	fig|6666666.34159.peg.2520	peg	NODE_96_length_748272_cov_42.517338_509993_509529	509993	5E+05	-	hypothetical protein		
NODE_96_length_748272_cov_42.517338	fig|6666666.34159.peg.2521	peg	NODE_96_length_748272_cov_42.517338_510760_510200	510760	5E+05	-	hypothetical protein		
NODE_96_length_748272_cov_42.517338	fig|6666666.34159.peg.2522	peg	NODE_96_length_748272_cov_42.517338_511024_510863	511024	5E+05	-	hypothetical protein		
NODE_96_length_748272_cov_42.517338	fig|6666666.34159.peg.2523	peg	NODE_96_length_748272_cov_42.517338_511199_511963	511199	5E+05	+	loricrin		
NODE_96_length_748272_cov_42.517338	fig|6666666.34159.peg.2524	peg	NODE_96_length_748272_cov_42.517338_512063_512527	512063	5E+05	+	hypothetical protein		
NODE_96_length_748272_cov_42.517338	fig|6666666.34159.peg.2525	peg	NODE_96_length_748272_cov_42.517338_512578_513426	512578	5E+05	+	Uncharacterized protein conserved in bacteria, NMA0228-like	FIG01515244	ff
NODE_96_length_748272_cov_42.517338	fig|6666666.34159.peg.2526	peg	NODE_96_length_748272_cov_42.517338_513423_514313	513423	5E+05	+	Conserved domain protein	FIG01349516	ff
NODE_96_length_748272_cov_42.517338	fig|6666666.34159.peg.2527	peg	NODE_96_length_748272_cov_42.517338_514344_515597	514344	5E+05	+	Ribosomal RNA small subunit methyltransferase B (EC 2.1.1.-)	FIG00138175	ff
NODE_96_length_748272_cov_42.517338	fig|6666666.34159.peg.2528	peg	NODE_96_length_748272_cov_42.517338_517141_515594	517141	5E+05	-	putative membrane protein	FIG00672847	ff
NODE_96_length_748272_cov_42.517338	fig|6666666.34159.peg.2529	peg	NODE_96_length_748272_cov_42.517338_517108_517233	517108	5E+05	+	hypothetical protein		
NODE_96_length_748272_cov_42.517338	fig|6666666.34159.peg.2530	peg	NODE_96_length_748272_cov_42.517338_518006_517209	518006	5E+05	-	Streptomycin resistance protein		
NODE_96_length_748272_cov_42.517338	fig|6666666.34159.peg.2531	peg	NODE_96_length_748272_cov_42.517338_518412_518008	518412	5E+05	-	protein of unknown function DUF1486		
NODE_96_length_748272_cov_42.517338	fig|6666666.34159.peg.2532	peg	NODE_96_length_748272_cov_42.517338_519509_518427	519509	5E+05	-	hypothetical protein	FIG00638284	ff
NODE_96_length_748272_cov_42.517338	fig|6666666.34159.peg.2533	peg	NODE_96_length_748272_cov_42.517338_520756_519563	520756	5E+05	-	Aspartate aminotransferase (EC 2.6.1.1)	FIG00719736	idu(1);Coenzyme_M_biosynthesis_--_gjo idu(1);Glutamine,_Glutamate,_Aspartate_and_Asparagine_Biosynthesis idu(1);CBSS
NODE_96_length_748272_cov_42.517338	fig|6666666.34159.peg.2534	peg	NODE_96_length_748272_cov_42.517338_523830_520759	523830	5E+05	-	Protein export cytoplasm protein SecA ATPase RNA helicase (TC 3.A.5.1.1)	FIG00034293	ff
NODE_96_length_748272_cov_42.517338	fig|6666666.34159.peg.2535	peg	NODE_96_length_748272_cov_42.517338_524925_523966	524925	5E+05	-	Arabinose 5-phosphate isomerase (EC 5.3.1.13)	FIG00000743	isu;KDO2-Lipid_A_biosynthesis
NODE_96_length_748272_cov_42.517338	fig|6666666.34159.peg.2536	peg	NODE_96_length_748272_cov_42.517338_525097_526551	525097	5E+05	+	Isocitrate dehydrogenase [NADP] (EC 1.1.1.42)	FIG00000600	isu;TCA_Cycle
NODE_96_length_748272_cov_42.517338	fig|6666666.34159.peg.2537	peg	NODE_96_length_748272_cov_42.517338_526710_527900	526710	5E+05	+	hypothetical protein		
NODE_96_length_748272_cov_42.517338	fig|6666666.34159.peg.2538	peg	NODE_96_length_748272_cov_42.517338_528718_527876	528718	5E+05	-	Glutamate racemase (EC 5.1.1.3)	FIG00000397	isu;Glutamine,_Glutamate,_Aspartate_and_Asparagine_Biosynthesis isu;Poly-gamma-glutamate_biosynthesis
NODE_96_length_748272_cov_42.517338	fig|6666666.34159.peg.2539	peg	NODE_96_length_748272_cov_42.517338_529391_528723	529391	5E+05	-	hypothetical protein		
NODE_96_length_748272_cov_42.517338	fig|6666666.34159.peg.2540	peg	NODE_96_length_748272_cov_42.517338_530116_529400	530116	5E+05	-	Outer membrane lipoprotein omp16 precursor	FIG00552544	isu;Ton_and_Tol_transport_systems
NODE_96_length_748272_cov_42.517338	fig|6666666.34159.peg.2541	peg	NODE_96_length_748272_cov_42.517338_530354_530482	530354	5E+05	+	hypothetical protein		
NODE_96_length_748272_cov_42.517338	fig|6666666.34159.peg.2542	peg	NODE_96_length_748272_cov_42.517338_530540_531793	530540	5E+05	+	Molybdopterin binding motif, CinA N-terminal domain / C-terminal domain of CinA type S		isu;NAD_and_NADP_cofactor_biosynthesis_global isu;Riboflavin_synthesis_cluster
NODE_96_length_748272_cov_42.517338	fig|6666666.34159.peg.2543	peg	NODE_96_length_748272_cov_42.517338_532189_533382	532189	5E+05	+	hypothetical protein	FIG00638284	ff
NODE_96_length_748272_cov_42.517338	fig|6666666.34159.peg.2544	peg	NODE_96_length_748272_cov_42.517338_533725_533516	533725	5E+05	-	hypothetical protein		
NODE_96_length_748272_cov_42.517338	fig|6666666.34159.peg.2545	peg	NODE_96_length_748272_cov_42.517338_534142_535329	534142	5E+05	+	unknown		
NODE_96_length_748272_cov_42.517338	fig|6666666.34159.peg.2546	peg	NODE_96_length_748272_cov_42.517338_536038_535337	536038	5E+05	-	hypothetical protein		
NODE_96_length_748272_cov_42.517338	fig|6666666.34159.peg.2547	peg	NODE_96_length_748272_cov_42.517338_536342_536091	536342	5E+05	-	hypothetical protein		
NODE_96_length_748272_cov_42.517338	fig|6666666.34159.peg.2548	peg	NODE_96_length_748272_cov_42.517338_536938_536348	536938	5E+05	-	hypothetical protein	FIG00638284	ff
NODE_96_length_748272_cov_42.517338	fig|6666666.34159.peg.2549	peg	NODE_96_length_748272_cov_42.517338_537255_536941	537255	5E+05	-	hypothetical protein		
NODE_96_length_748272_cov_42.517338	fig|6666666.34159.peg.2550	peg	NODE_96_length_748272_cov_42.517338_537386_538459	537386	5E+05	+	hypothetical protein	FIG00638284	ff
NODE_96_length_748272_cov_42.517338	fig|6666666.34159.peg.2551	peg	NODE_96_length_748272_cov_42.517338_538776_538501	538776	5E+05	-	COG3237: Uncharacterized protein conserved in bacteria		
NODE_96_length_748272_cov_42.517338	fig|6666666.34159.peg.2552	peg	NODE_96_length_748272_cov_42.517338_539344_538832	539344	5E+05	-	Protein yciF		
NODE_96_length_748272_cov_42.517338	fig|6666666.34159.peg.2553	peg	NODE_96_length_748272_cov_42.517338_540067_539513	540067	5E+05	-	Succinyl-CoA ligase [ADP-forming] beta chain (EC 6.2.1.5)	FIG00000337	idu(1);TCA_Cycle
NODE_96_length_748272_cov_42.517338	fig|6666666.34159.peg.2554	peg	NODE_96_length_748272_cov_42.517338_540585_540103	540585	5E+05	-	putative hyperosmotically inducible periplasmic protein		
NODE_96_length_748272_cov_42.517338	fig|6666666.34159.peg.2555	peg	NODE_96_length_748272_cov_42.517338_540879_544313	540879	5E+05	+	Exodeoxyribonuclease V gamma chain (EC 3.1.11.5)	FIG00493440	icw(1);DNA_repair,_bacterial_RecBCD_pathway
NODE_96_length_748272_cov_42.517338	fig|6666666.34159.peg.2556	peg	NODE_96_length_748272_cov_42.517338_544306_547809	544306	5E+05	+	Exodeoxyribonuclease V beta chain (EC 3.1.11.5)	FIG00043030	isu;DNA_repair,_bacterial_RecBCD_pathway
NODE_96_length_748272_cov_42.517338	fig|6666666.34159.peg.2557	peg	NODE_96_length_748272_cov_42.517338_547797_549590	547797	5E+05	+	Exodeoxyribonuclease V alpha chain (EC 3.1.11.5)	FIG00047674	isu;CBSS-269801.1.peg.2186 icw(1);DNA_repair,_bacterial_RecBCD_pathway
NODE_96_length_748272_cov_42.517338	fig|6666666.34159.peg.2558	peg	NODE_96_length_748272_cov_42.517338_550614_549598	550614	5E+05	-	hypothetical protein		
NODE_96_length_748272_cov_42.517338	fig|6666666.34159.peg.2559	peg	NODE_96_length_748272_cov_42.517338_550931_550632	550931	6E+05	-	hypothetical protein		

NODE_96_length_748272_cov_42.517338	fig|6666666.34159.peg.2560	peg	NODE_96_length_748272_cov_42.517338_552292_551099	552292	6E+05	-	Probable Co/Zn/Cd efflux system membrane fusion protein	FIG00001932	icw(1);Cobalt-zinc-cadmium_resistance
NODE_96_length_748272_cov_42.517338	fig|6666666.34159.peg.2561	peg	NODE_96_length_748272_cov_42.517338_555497_552285	555497	6E+05	-	Cobalt-zinc-cadmium resistance protein CzcA; Cation efflux system protein CusA	FIG00008299	idu(2);Cobalt-zinc-cadmium_resistance icw(1);Cobalt-zinc-cadmium_resistance
NODE_96_length_748272_cov_42.517338	fig|6666666.34159.peg.2562	peg	NODE_96_length_748272_cov_42.517338_555636_556979	555636	6E+05	+	putative outer membrane protein		
NODE_96_length_748272_cov_42.517338	fig|6666666.34159.peg.2563	peg	NODE_96_length_748272_cov_42.517338_558147_556984	558147	6E+05	-	hypothetical protein		
NODE_96_length_748272_cov_42.517338	fig|6666666.34159.peg.2564	peg	NODE_96_length_748272_cov_42.517338_559390_558140	559390	6E+05	-	Integral membrane protein TerC	FIG00948083	ff
NODE_96_length_748272_cov_42.517338	fig|6666666.34159.peg.2565	peg	NODE_96_length_748272_cov_42.517338_560545_559454	560545	6E+05	-	hypothetical protein		
NODE_96_length_748272_cov_42.517338	fig|6666666.34159.peg.2566	peg	NODE_96_length_748272_cov_42.517338_561941_560721	561941	6E+05	-	LPS biosynthesis protein-related protein		
NODE_96_length_748272_cov_42.517338	fig|6666666.34159.peg.2567	peg	NODE_96_length_748272_cov_42.517338_563417_561945	563417	6E+05	-	hypothetical protein	FIG00638284	ff
NODE_96_length_748272_cov_42.517338	fig|6666666.34159.peg.2568	peg	NODE_96_length_748272_cov_42.517338_563578_564267	563578	6E+05	+	Phosphate regulon transcriptional regulatory protein PhoB (SphR)	FIG01290653	isu;High_affinity_phosphate_transporter_and_control_of_PHO_regulon isu;PhoR-PhoB_two-component_regulatory_system
NODE_96_length_748272_cov_42.517338	fig|6666666.34159.peg.2569	peg	NODE_96_length_748272_cov_42.517338_564866_564264	564866	6E+05	-	Phosphoserine phosphatase (EC 3.1.3.3)	FIG00000149	idu(1);Glycine_and_Serine_Utilization idu(1);Serine_Biosynthesis
NODE_96_length_748272_cov_42.517338	fig|6666666.34159.peg.2570	peg	NODE_96_length_748272_cov_42.517338_564986_565879	564986	6E+05	+	hypothetical protein	FIG00638284	ff
NODE_96_length_748272_cov_42.517338	fig|6666666.34159.peg.2571	peg	NODE_96_length_748272_cov_42.517338_565979_566467	565979	6E+05	+	integral membrane protein		
NODE_96_length_748272_cov_42.517338	fig|6666666.34159.peg.2572	peg	NODE_96_length_748272_cov_42.517338_568502_566475	568502	6E+05	-	methyl-accepting chemotaxis transducer		
NODE_96_length_748272_cov_42.517338	fig|6666666.34159.peg.2573	peg	NODE_96_length_748272_cov_42.517338_568703_570151	568703	6E+05	+	FOG: Ankyrin repeat-like		
NODE_96_length_748272_cov_42.517338	fig|6666666.34159.peg.2574	peg	NODE_96_length_748272_cov_42.517338_570463_571683	570463	6E+05	+	Tyrosine-specific transport protein	FIG00004484	ff
NODE_96_length_748272_cov_42.517338	fig|6666666.34159.peg.2575	peg	NODE_96_length_748272_cov_42.517338_571688_572905	571688	6E+05	+	Tyrosine-specific transport protein	FIG00004484	ff
NODE_96_length_748272_cov_42.517338	fig|6666666.34159.peg.2576	peg	NODE_96_length_748272_cov_42.517338_572920_573990	572920	6E+05	+	Tryptophanyl-tRNA synthetase (EC 6.1.1.2)	FIG00000055	isu;tRNA_aminoacylation,_Trp
NODE_96_length_748272_cov_42.517338	fig|6666666.34159.peg.2577	peg	NODE_96_length_748272_cov_42.517338_573987_575996	573987	6E+05	+	Excinuclease ABC subunit B	FIG00000146	isu;DNA_repair,_UvrABC_system
NODE_96_length_748272_cov_42.517338	fig|6666666.34159.peg.2578	peg	NODE_96_length_748272_cov_42.517338_575996_577159	575996	6E+05	+	hypothetical protein	FIG00638284	ff
NODE_96_length_748272_cov_42.517338	fig|6666666.34159.peg.2579	peg	NODE_96_length_748272_cov_42.517338_577484_577149	577484	6E+05	-	hypothetical protein		
NODE_96_length_748272_cov_42.517338	fig|6666666.34159.peg.2580	peg	NODE_96_length_748272_cov_42.517338_578995_577478	578995	6E+05	-	hypothetical protein	FIG00638284	ff
NODE_96_length_748272_cov_42.517338	fig|6666666.34159.peg.2581	peg	NODE_96_length_748272_cov_42.517338_579191_579694	579191	6E+05	+	Mobile element protein	FIG01306568	ff
NODE_96_length_748272_cov_42.517338	fig|6666666.34159.peg.2582	peg	NODE_96_length_748272_cov_42.517338_580652_580494	580652	6E+05	-	hypothetical protein		
NODE_96_length_748272_cov_42.517338	fig|6666666.34159.peg.2583	peg	NODE_96_length_748272_cov_42.517338_580672_581487	580672	6E+05	+	Cytochrome O ubiquinol oxidase subunit II (EC 1.10.3.-)	FIG00001095	icw(3);Terminal_cytochrome_O_ubiquinol_oxidase icw(3);Terminal_cytochrome_oxidases
NODE_96_length_748272_cov_42.517338	fig|6666666.34159.peg.2584	peg	NODE_96_length_748272_cov_42.517338_581495_583465	581495	6E+05	+	Cytochrome O ubiquinol oxidase subunit I (EC 1.10.3.-)	FIG00001077	icw(2);Terminal_cytochrome_O_ubiquinol_oxidase icw(2);Terminal_cytochrome_oxidases
NODE_96_length_748272_cov_42.517338	fig|6666666.34159.peg.2585	peg	NODE_96_length_748272_cov_42.517338_583462_584058	583462	6E+05	+	Cytochrome O ubiquinol oxidase subunit III (EC 1.10.3.-)	FIG00001056	isu;Terminal_cytochrome_O_ubiquinol_oxidase isu;Terminal_cytochrome_oxidases
NODE_96_length_748272_cov_42.517338	fig|6666666.34159.peg.2586	peg	NODE_96_length_748272_cov_42.517338_584059_584421	584059	6E+05	+	Cytochrome O ubiquinol oxidase subunit IV (EC 1.10.3.-)	FIG00001063	icw(1);Terminal_cytochrome_O_ubiquinol_oxidase icw(1);Terminal_cytochrome_oxidases
NODE_96_length_748272_cov_42.517338	fig|6666666.34159.peg.2587	peg	NODE_96_length_748272_cov_42.517338_584414_585262	584414	6E+05	+	Heme O synthase, protoheme IX farnesyltransferase (EC 2.5.1.-) COX10-CtaB	FIG00000497	isu;CBSS-316057.3.peg.563 isu;Biogenesis_of_cytochrome_c_oxidases
NODE_96_length_748272_cov_42.517338	fig|6666666.34159.peg.2588	peg	NODE_96_length_748272_cov_42.517338_585414_586250	585414	6E+05	+	GCN5-related N-acetyltransferase	FIG01272034	ff
NODE_96_length_748272_cov_42.517338	fig|6666666.34159.peg.2589	peg	NODE_96_length_748272_cov_42.517338_587027_586251	587027	6E+05	-	Succinate dehydrogenase iron-sulfur protein (EC 1.3.99.1)	FIG00026006	icw(1);TCA_Cycle isu;Succinate_dehydrogenase
NODE_96_length_748272_cov_42.517338	fig|6666666.34159.peg.2590	peg	NODE_96_length_748272_cov_42.517338_588937_587039	588937	6E+05	-	Succinate dehydrogenase flavoprotein subunit (EC 1.3.99.1)	FIG00136922	isu;TCA_Cycle icw(2);Succinate_dehydrogenase
NODE_96_length_748272_cov_42.517338	fig|6666666.34159.peg.2591	peg	NODE_96_length_748272_cov_42.517338_589970_588951	589970	6E+05	-	Succinate dehydrogenase cytochrome b558 subunit	FIG01304139	icw(1);Succinate_dehydrogenase
NODE_96_length_748272_cov_42.517338	fig|6666666.34159.peg.2592	peg	NODE_96_length_748272_cov_42.517338_590448_590179	590448	6E+05	-	hypothetical protein		
NODE_96_length_748272_cov_42.517338	fig|6666666.34159.peg.2593	peg	NODE_96_length_748272_cov_42.517338_590778_590948	590778	6E+05	+	Mobile element protein	FIG01306568	ff
NODE_96_length_748272_cov_42.517338	fig|6666666.34159.peg.2594	peg	NODE_96_length_748272_cov_42.517338_591580_591762	591580	6E+05	+	hypothetical protein		
NODE_96_length_748272_cov_42.517338	fig|6666666.34159.peg.2595	peg	NODE_96_length_748272_cov_42.517338_592034_592681	592034	6E+05	+	hypothetical protein		
NODE_96_length_748272_cov_42.517338	fig|6666666.34159.peg.2596	peg	NODE_96_length_748272_cov_42.517338_593382_593801	593382	6E+05	+	hypothetical protein		
NODE_96_length_748272_cov_42.517338	fig|6666666.34159.peg.2597	peg	NODE_96_length_748272_cov_42.517338_593865_595262	593865	6E+05	+	hypothetical protein		
NODE_96_length_748272_cov_42.517338	fig|6666666.34159.peg.2598	peg	NODE_96_length_748272_cov_42.517338_595394_595567	595394	6E+05	+	putative transposase		
NODE_96_length_748272_cov_42.517338	fig|6666666.34159.peg.2599	peg	NODE_96_length_748272_cov_42.517338_595607_596137	595607	6E+05	+	putative transposase		
NODE_96_length_748272_cov_42.517338	fig|6666666.34159.peg.2600	peg	NODE_96_length_748272_cov_42.517338_596186_597649	596186	6E+05	+	hypothetical protein		
NODE_96_length_748272_cov_42.517338	fig|6666666.34159.peg.2601	peg	NODE_96_length_748272_cov_42.517338_597770_597892	597770	6E+05	+	hypothetical protein		
NODE_96_length_748272_cov_42.517338	fig|6666666.34159.peg.2602	peg	NODE_96_length_748272_cov_42.517338_597997_598956	597997	6E+05	+	hypothetical protein		
NODE_96_length_748272_cov_42.517338	fig|6666666.34159.peg.2603	peg	NODE_96_length_748272_cov_42.517338_599926_598943	599926	6E+05	-	hypothetical protein		
NODE_96_length_748272_cov_42.517338	fig|6666666.34159.peg.2604	peg	NODE_96_length_748272_cov_42.517338_600789_599923	600789	6E+05	-	hypothetical protein PA3071	FIG00137450	isu;Aerotolerance_operon_in_Bacteroides_and_potentially_orthologous_operons_in_other_organisms
NODE_96_length_748272_cov_42.517338	fig|6666666.34159.peg.2605	peg	NODE_96_length_748272_cov_42.517338_601976_600792	601976	6E+05	-	Arsenic efflux pump protein	FIG00081580	idu(1);Arsenic_resistance
NODE_96_length_748272_cov_42.517338	fig|6666666.34159.peg.2606	peg	NODE_96_length_748272_cov_42.517338_602301_602062	602301	6E+05	-	hypothetical protein		
NODE_96_length_748272_cov_42.517338	fig|6666666.34159.peg.2607	peg	NODE_96_length_748272_cov_42.517338_603549_602374	603549	6E+05	-	Pheromone shutdown protein		
NODE_96_length_748272_cov_42.517338	fig|6666666.34159.peg.2608	peg	NODE_96_length_748272_cov_42.517338_604339_603623	604339	6E+05	-	Uracil-DNA glycosylase, family 1	FIG00000538	isu;DNA_Repair_Base_Excision isu;Uracil-DNA_glycosylase
NODE_96_length_748272_cov_42.517338	fig|6666666.34159.peg.2609	peg	NODE_96_length_748272_cov_42.517338_604325_604468	604325	6E+05	+	hypothetical protein		
NODE_96_length_748272_cov_42.517338	fig|6666666.34159.peg.2610	peg	NODE_96_length_748272_cov_42.517338_604503_604640	604503	6E+05	+	hypothetical protein		
NODE_96_length_748272_cov_42.517338	fig|6666666.34159.peg.2611	peg	NODE_96_length_748272_cov_42.517338_604671_605399	604671	6E+05	+	Uridylate kinase (EC 2.7.4.-)	FIG00001819	ff
NODE_96_length_748272_cov_42.517338	fig|6666666.34159.peg.2612	peg	NODE_96_length_748272_cov_42.517338_605418_605969	605418	6E+05	+	Ribosome recycling factor	FIG00000202	isu;Ribosome_recycling_related_cluster isu;Translation_termination_factors_bacterial
NODE_96_length_748272_cov_42.517338	fig|6666666.34159.rna.38	rna	NODE_96_length_748272_cov_42.517338_606064_606138	606064	6E+05	+	tRNA-Glu-TTC		
NODE_96_length_748272_cov_42.517338	fig|6666666.34159.rna.39	rna	NODE_96_length_748272_cov_42.517338_606149_606220	606149	6E+05	+	tRNA-Lys-TTT		
NODE_96_length_748272_cov_42.517338	fig|6666666.34159.peg.2613	peg	NODE_96_length_748272_cov_42.517338_606384_606947	606384	6E+05	+	FIG00493978: hypothetical protein	FIG00493320	ff
NODE_96_length_748272_cov_42.517338	fig|6666666.34159.peg.2614	peg	NODE_96_length_748272_cov_42.517338_606934_607998	606934	6E+05	+	Putative ATP:guanido phosphotransferase YacI (EC 2.7.3.-)	FIG00007067	isu;Proteolysis_in_bacteria,_ATP-dependent
NODE_96_length_748272_cov_42.517338	fig|6666666.34159.peg.2615	peg	NODE_96_length_748272_cov_42.517338_608005_609090	608005	6E+05	+	hypothetical protein		
NODE_96_length_748272_cov_42.517338	fig|6666666.34159.peg.2616	peg	NODE_96_length_748272_cov_42.517338_610067_609087	610067	6E+05	-	Lysine 2,3-aminomutase (EC 5.4.3.2)	FIG00134830	isu;Lysine_degradation

NODE_96_length_748272_cov_42.517338	fig|6666666.34159.peg.2617	peg	NODE_96_length_748272_cov_42.517338_610157_611122	610157	6E+05	+	Deacetylases, including yeast histone deacetylase and acetoin utilization protein	FIG00001794	isu;Hydantoin_metabolism
NODE_96_length_748272_cov_42.517338	fig|6666666.34159.peg.2618	peg	NODE_96_length_748272_cov_42.517338_611558_611205	611558	6E+05	-	hypothetical protein		
NODE_96_length_748272_cov_42.517338	fig|6666666.34159.peg.2619	peg	NODE_96_length_748272_cov_42.517338_612007_613347	612007	6E+05	+	hypothetical protein	FIG00638284	ff
NODE_96_length_748272_cov_42.517338	fig|6666666.34159.peg.2620	peg	NODE_96_length_748272_cov_42.517338_614342_613344	614342	6E+05	-	hypothetical protein		
NODE_96_length_748272_cov_42.517338	fig|6666666.34159.peg.2621	peg	NODE_96_length_748272_cov_42.517338_615229_614345	615229	6E+05	-	possible Glycosyl transferase		
NODE_96_length_748272_cov_42.517338	fig|6666666.34159.peg.2622	peg	NODE_96_length_748272_cov_42.517338_615987_615238	615987	6E+05	-	glycosyltransferase family 6		
NODE_96_length_748272_cov_42.517338	fig|6666666.34159.peg.2623	peg	NODE_96_length_748272_cov_42.517338_616147_616926	616147	6E+05	+	hypothetical protein		
NODE_96_length_748272_cov_42.517338	fig|6666666.34159.peg.2624	peg	NODE_96_length_748272_cov_42.517338_617070_618113	617070	6E+05	+	hypothetical protein		
NODE_96_length_748272_cov_42.517338	fig|6666666.34159.peg.2625	peg	NODE_96_length_748272_cov_42.517338_618298_618624	618298	6E+05	+	hypothetical protein		
NODE_96_length_748272_cov_42.517338	fig|6666666.34159.peg.2626	peg	NODE_96_length_748272_cov_42.517338_618729_620432	618729	6E+05	+	hypothetical protein	FIG00638284	ff
NODE_96_length_748272_cov_42.517338	fig|6666666.34159.peg.2627	peg	NODE_96_length_748272_cov_42.517338_620439_621062	620439	6E+05	+	hypothetical protein		
NODE_96_length_748272_cov_42.517338	fig|6666666.34159.peg.2628	peg	NODE_96_length_748272_cov_42.517338_621065_622345	621065	6E+05	+	hypothetical protein		
NODE_96_length_748272_cov_42.517338	fig|6666666.34159.peg.2629	peg	NODE_96_length_748272_cov_42.517338_622342_622455	622342	6E+05	+	hypothetical protein		
NODE_96_length_748272_cov_42.517338	fig|6666666.34159.peg.2630	peg	NODE_96_length_748272_cov_42.517338_622653_624983	622653	6E+05	+	hypothetical protein	FIG00638284	ff
NODE_96_length_748272_cov_42.517338	fig|6666666.34159.rna.40	rna	NODE_96_length_748272_cov_42.517338_625248_625161	625248	6E+05	-	tRNA-Ser-GGA		isu;tRNAs
NODE_96_length_748272_cov_42.517338	fig|6666666.34159.peg.2631	peg	NODE_96_length_748272_cov_42.517338_625605_626723	625605	6E+05	+	hypothetical protein		
NODE_96_length_748272_cov_42.517338	fig|6666666.34159.peg.2632	peg	NODE_96_length_748272_cov_42.517338_627177_626701	627177	6E+05	-	hypothetical protein		
NODE_96_length_748272_cov_42.517338	fig|6666666.34159.peg.2633	peg	NODE_96_length_748272_cov_42.517338_627387_628685	627387	6E+05	+	UDP-glucose dehydrogenase (EC 1.1.1.22)	FIG00000532	idu(1);Inteins icw(1);Lipid_A-Ara4N_pathway_(_Polymyxin_resistance_)
NODE_96_length_748272_cov_42.517338	fig|6666666.34159.peg.2634	peg	NODE_96_length_748272_cov_42.517338_628707_629813	628707	6E+05	+	UDP-4-amino-4-deoxy-L-arabinose--oxoglutarate aminotransferase (EC 2.6.1.-)	FIG00046703	isu;Lipid_A-Ara4N_pathway_(_Polymyxin_resistance_)
NODE_96_length_748272_cov_42.517338	fig|6666666.34159.peg.2635	peg	NODE_96_length_748272_cov_42.517338_629829_631496	629829	6E+05	+	2,3,4,5-tetrahydropyridine-2,6-dicarboxylate N-acetyltransferase (EC 2.3.1.89)	FIG00001821	isu;Lysine_Biosynthesis_DAP_Pathway,_GJO_scratch isu;Lysine_Biosynthesis_DAP_Pathway
NODE_96_length_748272_cov_42.517338	fig|6666666.34159.peg.2636	peg	NODE_96_length_748272_cov_42.517338_631508_632584	631508	6E+05	+	UDP-N-acetylglucosamine 2-epimerase (EC 5.1.3.14)	FIG00000482	isu;CMP-N-acetylneuraminate_Biosynthesis isu;Sialic_Acid_Metabolism
NODE_96_length_748272_cov_42.517338	fig|6666666.34159.peg.2637	peg	NODE_96_length_748272_cov_42.517338_632959_632642	632959	6E+05	-	hypothetical protein		
NODE_96_length_748272_cov_42.517338	fig|6666666.34159.peg.2638	peg	NODE_96_length_748272_cov_42.517338_633018_633173	633018	6E+05	+	hypothetical protein		
NODE_96_length_748272_cov_42.517338	fig|6666666.34159.peg.2639	peg	NODE_96_length_748272_cov_42.517338_633383_633538	633383	6E+05	+	hypothetical protein		
NODE_96_length_748272_cov_42.517338	fig|6666666.34159.peg.2640	peg	NODE_96_length_748272_cov_42.517338_634970_633933	634970	6E+05	-	Ribonucleotide reductase of class Ia (aerobic), beta subunit (EC 1.17.4.1)	FIG00000585	isu;Ribonucleotide_reduction
NODE_96_length_748272_cov_42.517338	fig|6666666.34159.peg.2641	peg	NODE_96_length_748272_cov_42.517338_637870_635015	637870	6E+05	-	Ribonucleotide reductase of class Ia (aerobic), alpha subunit (EC 1.17.4.1)	FIG00000606	icw(1);Ribonucleotide_reduction
NODE_96_length_748272_cov_42.517338	fig|6666666.34159.peg.2642	peg	NODE_96_length_748272_cov_42.517338_638375_639193	638375	6E+05	+	Inositol-1-monophosphatase (EC 3.1.3.25)	FIG00002332	idu(1);Di-Inositol-Phosphate_biosynthesis
NODE_96_length_748272_cov_42.517338	fig|6666666.34159.peg.2643	peg	NODE_96_length_748272_cov_42.517338_640698_639217	640698	6E+05	-	hypothetical protein		
NODE_96_length_748272_cov_42.517338	fig|6666666.34159.peg.2644	peg	NODE_96_length_748272_cov_42.517338_641072_642145	641072	6E+05	+	RecA protein	FIG00000234	isu;DNA_repair,_bacterial_UmuCD_system isu;DNA_repair_system_including_RecA,_MutS_and_a_hypothetical_protein
NODE_96_length_748272_cov_42.517338	fig|6666666.34159.peg.2645	peg	NODE_96_length_748272_cov_42.517338_643041_642148	643041	6E+05	-	Octaprenyl-diphosphate synthase (EC 2.5.1.-) / Dimethylallyltransferase (EC 2.5.1.1) / Geranyltranstransferase (farnesyldiphosphate synthase) (EC 2.5.1.10) / Geranylgeranyl	FIG00000019	idu(1);Isoprenoid_Biosynthesis
NODE_96_length_748272_cov_42.517338	fig|6666666.34159.peg.2646	peg	NODE_96_length_748272_cov_42.517338_644193_643141	644193	6E+05	-	hypothetical protein		
NODE_96_length_748272_cov_42.517338	fig|6666666.34159.peg.2647	peg	NODE_96_length_748272_cov_42.517338_644887_644186	644887	6E+05	-	Glucosamine-1-phosphate N-acetyltransferase (EC 2.3.1.157)	FIG00061832	isu;Sialic_Acid_Metabolism isu;UDP-N-acetylmuramate_from_Fructose-6-phosphate_Biosynthesis
NODE_96_length_748272_cov_42.517338	fig|6666666.34159.peg.2648	peg	NODE_96_length_748272_cov_42.517338_645119_645250	645119	6E+05	+	hypothetical protein		
NODE_96_length_748272_cov_42.517338	fig|6666666.34159.peg.2649	peg	NODE_96_length_748272_cov_42.517338_645391_645507	645391	6E+05	+	hypothetical protein		
NODE_96_length_748272_cov_42.517338	fig|6666666.34159.peg.2650	peg	NODE_96_length_748272_cov_42.517338_645591_646241	645591	6E+05	+	FIG002283: Isochorismatase family protein	FIG00002283	ff
NODE_96_length_748272_cov_42.517338	fig|6666666.34159.peg.2651	peg	NODE_96_length_748272_cov_42.517338_647016_646285	647016	6E+05	-	probable cytosol aminopeptidase(EC:3.4.11.1)	FIG01748060	ff
NODE_96_length_748272_cov_42.517338	fig|6666666.34159.peg.2652	peg	NODE_96_length_748272_cov_42.517338_647153_648982	647153	6E+05	+	Exoenzymes regulatory protein AepA precursor	FIG00002517	ff
NODE_96_length_748272_cov_42.517338	fig|6666666.34159.peg.2653	peg	NODE_96_length_748272_cov_42.517338_649236_648991	649236	6E+05	-	hypothetical protein		
NODE_96_length_748272_cov_42.517338	fig|6666666.34159.peg.2654	peg	NODE_96_length_748272_cov_42.517338_649457_649218	649457	6E+05	-	hypothetical protein		
NODE_96_length_748272_cov_42.517338	fig|6666666.34159.peg.2655	peg	NODE_96_length_748272_cov_42.517338_649607_649473	649607	6E+05	-	hypothetical protein		
NODE_96_length_748272_cov_42.517338	fig|6666666.34159.peg.2656	peg	NODE_96_length_748272_cov_42.517338_649784_649641	649784	6E+05	-	hypothetical protein		
NODE_96_length_748272_cov_42.517338	fig|6666666.34159.peg.2657	peg	NODE_96_length_748272_cov_42.517338_649872_650072	649872	7E+05	+	hypothetical protein		
NODE_96_length_748272_cov_42.517338	fig|6666666.34159.peg.2658	peg	NODE_96_length_748272_cov_42.517338_650032_650193	650032	7E+05	+	hypothetical protein		
NODE_96_length_748272_cov_42.517338	fig|6666666.34159.rna.41	rna	NODE_96_length_748272_cov_42.517338_650456_650382	650456	7E+05	-	tRNA-Pro-GGG		isu;tRNAs
NODE_96_length_748272_cov_42.517338	fig|6666666.34159.peg.2659	peg	NODE_96_length_748272_cov_42.517338_650718_650467	650718	7E+05	-	hypothetical protein		
NODE_96_length_748272_cov_42.517338	fig|6666666.34159.peg.2660	peg	NODE_96_length_748272_cov_42.517338_652194_650791	652194	7E+05	-	putative exported protein	FIG01956019	ff
NODE_96_length_748272_cov_42.517338	fig|6666666.34159.peg.2661	peg	NODE_96_length_748272_cov_42.517338_652409_653005	652409	7E+05	+	hypothetical protein		
NODE_96_length_748272_cov_42.517338	fig|6666666.34159.peg.2662	peg	NODE_96_length_748272_cov_42.517338_654363_653050	654363	7E+05	-	hypothetical protein		
NODE_96_length_748272_cov_42.517338	fig|6666666.34159.peg.2663	peg	NODE_96_length_748272_cov_42.517338_655208_654369	655208	7E+05	-	hypothetical protein		
NODE_96_length_748272_cov_42.517338	fig|6666666.34159.peg.2664	peg	NODE_96_length_748272_cov_42.517338_656497_655205	656497	7E+05	-	TPR/glycosyl transferase domain protein	FIG01338019	ff
NODE_96_length_748272_cov_42.517338	fig|6666666.34159.peg.2665	peg	NODE_96_length_748272_cov_42.517338_657180_656584	657180	7E+05	-	Pyridoxine biosynthesis glutamine amidotransferase, glutaminase subunit (EC 2.4.2.-)	FIG00001203	icw(1);Pyridoxin_(Vitamin_B6)_Biosynthesis
NODE_96_length_748272_cov_42.517338	fig|6666666.34159.peg.2666	peg	NODE_96_length_748272_cov_42.517338_658092_657184	658092	7E+05	-	Pyridoxine biosynthesis glutamine amidotransferase, synthase subunit (EC 2.4.2.-)	FIG00021445	isu;Pyridoxin_(Vitamin_B6)_Biosynthesis
NODE_96_length_748272_cov_42.517338	fig|6666666.34159.peg.2667	peg	NODE_96_length_748272_cov_42.517338_658930_658181	658930	7E+05	-	DNA polymerase III subunit epsilon	FIG01333888	ff
NODE_96_length_748272_cov_42.517338	fig|6666666.34159.peg.2668	peg	NODE_96_length_748272_cov_42.517338_659586_658969	659586	7E+05	-	GTP-binding protein EngB	FIG00000298	ff
NODE_96_length_748272_cov_42.517338	fig|6666666.34159.peg.2669	peg	NODE_96_length_748272_cov_42.517338_660029_659583	660029	7E+05	-	ATPase YjeE, predicted to have essential role in cell wall biosynthesis	FIG00000228	ff
NODE_96_length_748272_cov_42.517338	fig|6666666.34159.peg.2670	peg	NODE_96_length_748272_cov_42.517338_660922_660200	660922	7E+05	-	FIG00899449: hypothetical protein	FIG00899448	ff
NODE_96_length_748272_cov_42.517338	fig|6666666.34159.peg.2671	peg	NODE_96_length_748272_cov_42.517338_661224_662300	661224	7E+05	+	Leucine dehydrogenase (EC 1.4.1.9)	FIG00020626	isu;Branched-Chain_Amino_Acid_Biosynthesis
NODE_96_length_748272_cov_42.517338	fig|6666666.34159.peg.2672	peg	NODE_96_length_748272_cov_42.517338_662878_662348	662878	7E+05	-	Substrate-specific component BioY of biotin ECF transporter	FIG00012067	isu;Biotin_synthesis_cluster isu;Biotin_biosynthesis
NODE_96_length_748272_cov_42.517338	fig|6666666.34159.peg.2673	peg	NODE_96_length_748272_cov_42.517338_663859_662945	663859	7E+05	-	Twin-arginine translocation protein TatC	FIG00000335	isu;Twin-arginine_translocation_system

NODE_96_length_748272_cov_42.517338	fig|6666666.34159.peg.2674	peg	NODE_96_length_748272_cov_42.517338_664069_663866	664069	7E+05	-		hypothetical protein		
NODE_96_length_748272_cov_42.517338	fig|6666666.34159.peg.2675	peg	NODE_96_length_748272_cov_42.517338_664828_664082	664828	7E+05	-		Virulence plasmid protein pGP6-D		
NODE_96_length_748272_cov_42.517338	fig|6666666.34159.peg.2676	peg	NODE_96_length_748272_cov_42.517338_665636_664881	665636	7E+05	-		Septum site-determining protein MinD	FIG00000519	idu(1);Septum_site-determining_cluster_Min
NODE_96_length_748272_cov_42.517338	fig|6666666.34159.peg.2677	peg	NODE_96_length_748272_cov_42.517338_667690_665765	667690	7E+05	-		Threonyl-tRNA synthetase (EC 6.1.1.3)	FIG00000101	idu(1);tRNA_aminoacylation,_Thr
NODE_96_length_748272_cov_42.517338	fig|6666666.34159.peg.2678	peg	NODE_96_length_748272_cov_42.517338_667837_670806	667837	7E+05	+		hypothetical protein	FIG00638284	ff
NODE_96_length_748272_cov_42.517338	fig|6666666.34159.peg.2679	peg	NODE_96_length_748272_cov_42.517338_671022_671159	671022	7E+05	+		hypothetical protein		
NODE_96_length_748272_cov_42.517338	fig|6666666.34159.peg.2680	peg	NODE_96_length_748272_cov_42.517338_671295_672689	671295	7E+05	+		hypothetical protein		
NODE_96_length_748272_cov_42.517338	fig|6666666.34159.peg.2681	peg	NODE_96_length_748272_cov_42.517338_673519_673364	673519	7E+05	-		hypothetical protein		
NODE_96_length_748272_cov_42.517338	fig|6666666.34159.peg.2682	peg	NODE_96_length_748272_cov_42.517338_674163_674008	674163	7E+05	-		hypothetical protein		
NODE_96_length_748272_cov_42.517338	fig|6666666.34159.peg.2683	peg	NODE_96_length_748272_cov_42.517338_674347_679908	674347	7E+05	+		hypothetical protein	FIG00638284	ff
NODE_96_length_748272_cov_42.517338	fig|6666666.34159.peg.2684	peg	NODE_96_length_748272_cov_42.517338_680050_681732	680050	7E+05	+		hypothetical protein	FIG00638284	ff
NODE_96_length_748272_cov_42.517338	fig|6666666.34159.peg.2685	peg	NODE_96_length_748272_cov_42.517338_681925_682329	681925	7E+05	+		hypothetical protein		
NODE_96_length_748272_cov_42.517338	fig|6666666.34159.peg.2686	peg	NODE_96_length_748272_cov_42.517338_682920_682330	682920	7E+05	-		Alkyl hydroperoxide reductase subunit C-like protein	FIG01258694	idu(1);Oxidative_stress idu(1);Rubrerythrin idu(1);Thioredoxin-disulfide_reductase
NODE_96_length_748272_cov_42.517338	fig|6666666.34159.peg.2687	peg	NODE_96_length_748272_cov_42.517338_683604_682969	683604	7E+05	-		hypothetical protein		
NODE_96_length_748272_cov_42.517338	fig|6666666.34159.rna.42	rna	NODE_96_length_748272_cov_42.517338_683678_683605	683678	7E+05	-		tRNA-Arg-ACG		isu;tRNAs
NODE_96_length_748272_cov_42.517338	fig|6666666.34159.peg.2688	peg	NODE_96_length_748272_cov_42.517338_685138_683759	685138	7E+05	-		tolB protein precursor, periplasmic protein involved in the tonb-independent uptake of group A colicins	FIG00000654	icw(1);Ton_and_Tol_transport_systems
NODE_96_length_748272_cov_42.517338	fig|6666666.34159.peg.2689	peg	NODE_96_length_748272_cov_42.517338_686005_685154	686005	7E+05	-		hypothetical protein		
NODE_96_length_748272_cov_42.517338	fig|6666666.34159.peg.2690	peg	NODE_96_length_748272_cov_42.517338_686508_686086	686508	7E+05	-		Biopolymer transport protein ExbD/TolR	FIG00017761	icw(2);Ton_and_Tol_transport_systems
NODE_96_length_748272_cov_42.517338	fig|6666666.34159.peg.2691	peg	NODE_96_length_748272_cov_42.517338_687244_686519	687244	7E+05	-		MotA/TolQ/ExbB proton channel family protein	FIG00002161	idu(1);Ton_and_Tol_transport_systems
NODE_96_length_748272_cov_42.517338	fig|6666666.34159.peg.2692	peg	NODE_96_length_748272_cov_42.517338_687372_687488	687372	7E+05	+		hypothetical protein		
NODE_96_length_748272_cov_42.517338	fig|6666666.34159.peg.2693	peg	NODE_96_length_748272_cov_42.517338_687573_689759	687573	7E+05	+		Cytochrome c-type biogenesis protein DsbD, protein-disulfide reductase (EC 1.8.1.8)	FIG00000709	isu;Biogenesis_of_c-type_cytochromes isu;Periplasmic_disulfide_interchange
NODE_96_length_748272_cov_42.517338	fig|6666666.34159.peg.2694	peg	NODE_96_length_748272_cov_42.517338_689806_690558	689806	7E+05	+		Putative deoxyribonuclease YcfH	FIG00000184	isu;YcfH
NODE_96_length_748272_cov_42.517338	fig|6666666.34159.peg.2695	peg	NODE_96_length_748272_cov_42.517338_693387_690547	693387	7E+05	-		hypothetical protein	FIG00638284	ff
NODE_96_length_748272_cov_42.517338	fig|6666666.34159.peg.2696	peg	NODE_96_length_748272_cov_42.517338_694150_693395	694150	7E+05	-		hypothetical protein		
NODE_96_length_748272_cov_42.517338	fig|6666666.34159.peg.2697	peg	NODE_96_length_748272_cov_42.517338_695385_694147	695385	7E+05	-		tRNA nucleotidyltransferase (EC 2.7.7.21) (EC 2.7.7.25)	FIG00000289	isu;Polyadenylation_bacterial isu;tRNA_nucleotidyltransferase
NODE_96_length_748272_cov_42.517338	fig|6666666.34159.peg.2698	peg	NODE_96_length_748272_cov_42.517338_696683_695451	696683	7E+05	-		hypothetical protein		
NODE_96_length_748272_cov_42.517338	fig|6666666.34159.peg.2699	peg	NODE_96_length_748272_cov_42.517338_697894_696839	697894	7E+05	-		hypothetical protein	FIG00638284	ff
NODE_96_length_748272_cov_42.517338	fig|6666666.34159.peg.2700	peg	NODE_96_length_748272_cov_42.517338_699122_698184	699122	7E+05	-		Permease of the drug/metabolite transporter (DMT) superfamily	FIG00846773	isu;Queuosine-Archaeosine_Biosynthesis
NODE_96_length_748272_cov_42.517338	fig|6666666.34159.peg.2701	peg	NODE_96_length_748272_cov_42.517338_700241_699207	700241	7E+05	-		hypothetical protein	FIG00638284	ff
NODE_96_length_748272_cov_42.517338	fig|6666666.34159.peg.2702	peg	NODE_96_length_748272_cov_42.517338_701303_700248	701303	7E+05	-		nucleotide sugar epimerase/dehydratase	FIG01493118	ff
NODE_96_length_748272_cov_42.517338	fig|6666666.34159.peg.2703	peg	NODE_96_length_748272_cov_42.517338_702247_701300	702247	7E+05	-		nucleotide sugar epimerase/dehydratase		
NODE_96_length_748272_cov_42.517338	fig|6666666.34159.peg.2704	peg	NODE_96_length_748272_cov_42.517338_704229_702286	704229	7E+05	-		Asparagine synthetase [glutamine-hydrolyzing] (EC 6.3.5.4)	FIG00035175	idu(1);Glutamine,_Glutamate,_Aspartate_and_Asparagine_Biosynthesis idu(1);Glutamate_and_Aspartate_uptake_in_Bact
NODE_96_length_748272_cov_42.517338	fig|6666666.34159.peg.2705	peg	NODE_96_length_748272_cov_42.517338_704862_704401	704862	7E+05	-		hypothetical protein		
NODE_96_length_748272_cov_42.517338	fig|6666666.34159.peg.2706	peg	NODE_96_length_748272_cov_42.517338_705247_706530	705247	7E+05	+		hypothetical protein	FIG00638284	ff
NODE_96_length_748272_cov_42.517338	fig|6666666.34159.peg.2707	peg	NODE_96_length_748272_cov_42.517338_707881_706985	707881	7E+05	-		Uncharacterized isomerase yddE, PhzC-PhzF family		
NODE_96_length_748272_cov_42.517338	fig|6666666.34159.peg.2708	peg	NODE_96_length_748272_cov_42.517338_708501_707881	708501	7E+05	-		YheO-like PAS domain		
NODE_96_length_748272_cov_42.517338	fig|6666666.34159.peg.2709	peg	NODE_96_length_748272_cov_42.517338_708800_708684	708800	7E+05	-		hypothetical protein		
NODE_96_length_748272_cov_42.517338	fig|6666666.34159.peg.2710	peg	NODE_96_length_748272_cov_42.517338_710264_708837	710264	7E+05	-		RND efflux system, outer membrane lipoprotein, NodT family	FIG00005510	isu;Multidrug_Resistance_Efflux_Pumps
NODE_96_length_748272_cov_42.517338	fig|6666666.34159.peg.2711	peg	NODE_96_length_748272_cov_42.517338_711319_710261	711319	7E+05	-		hypothetical protein	FIG00638284	ff
NODE_96_length_748272_cov_42.517338	fig|6666666.34159.peg.2712	peg	NODE_96_length_748272_cov_42.517338_712002_711316	712002	7E+05	-		ABC transporter related	FIG01124919	ff
NODE_96_length_748272_cov_42.517338	fig|6666666.34159.peg.2713	peg	NODE_96_length_748272_cov_42.517338_713149_712004	713149	7E+05	-		hypothetical protein	FIG00638284	ff
NODE_96_length_748272_cov_42.517338	fig|6666666.34159.peg.2714	peg	NODE_96_length_748272_cov_42.517338_714221_713211	714221	7E+05	-		hypothetical protein		
NODE_96_length_748272_cov_42.517338	fig|6666666.34159.peg.2715	peg	NODE_96_length_748272_cov_42.517338_714457_715803	714457	7E+05	+		MATE efflux family protein		
NODE_96_length_748272_cov_42.517338	fig|6666666.34159.peg.2716	peg	NODE_96_length_748272_cov_42.517338_716524_715793	716524	7E+05	-		hypothetical protein	FIG00638284	ff
NODE_96_length_748272_cov_42.517338	fig|6666666.34159.peg.2717	peg	NODE_96_length_748272_cov_42.517338_717266_716514	717266	7E+05	-		Putative glutamine transport system permease	FIG01330392	ff
NODE_96_length_748272_cov_42.517338	fig|6666666.34159.peg.2718	peg	NODE_96_length_748272_cov_42.517338_718100_717285	718100	7E+05	-		Glutamine-binding periplasmic protein	FIG01308703	ff
NODE_96_length_748272_cov_42.517338	fig|6666666.34159.peg.2719	peg	NODE_96_length_748272_cov_42.517338_718221_718102	718221	7E+05	-		hypothetical protein		
NODE_96_length_748272_cov_42.517338	fig|6666666.34159.peg.2720	peg	NODE_96_length_748272_cov_42.517338_719641_718184	719641	7E+05	-		HtrA protease/chaperone protein	FIG00003002	isu;Periplasmic_Stress_Response
NODE_96_length_748272_cov_42.517338	fig|6666666.34159.peg.2721	peg	NODE_96_length_748272_cov_42.517338_719684_720286	719684	7E+05	+		hypothetical protein		
NODE_96_length_748272_cov_42.517338	fig|6666666.34159.peg.2722	peg	NODE_96_length_748272_cov_42.517338_720385_721677	720385	7E+05	+		Proton/glutamate symport protein @ Sodium/glutamate symport protein	FIG00008055	idu(2);Glutamate_and_Aspartate_uptake_in_Bacteria
NODE_96_length_748272_cov_42.517338	fig|6666666.34159.peg.2723	peg	NODE_96_length_748272_cov_42.517338_721683_722810	721683	7E+05	+		tRNA-specific 2-thiouridylase MnmA	FIG00000626	isu;RNA_methylation
NODE_96_length_748272_cov_42.517338	fig|6666666.34159.peg.2724	peg	NODE_96_length_748272_cov_42.517338_728081_723090	728081	7E+05	-		hypothetical protein	FIG00638284	ff
NODE_96_length_748272_cov_42.517338	fig|6666666.34159.peg.2725	peg	NODE_96_length_748272_cov_42.517338_728859_728113	728859	7E+05	-		Putative two-domain glycosyltransferase	FIG00014684	isu;LOS_core_oligosaccharide_biosynthesis
NODE_96_length_748272_cov_42.517338	fig|6666666.34159.peg.2726	peg	NODE_96_length_748272_cov_42.517338_729461_728901	729461	7E+05	-		Thiol peroxidase, Bcp-type (EC 1.11.1.15)	FIG00132548	isu;Thioredoxin-disulfide_reductase
NODE_96_length_748272_cov_42.517338	fig|6666666.34159.peg.2727	peg	NODE_96_length_748272_cov_42.517338_730339_729563	730339	7E+05	-		unknown protein		
NODE_96_length_748272_cov_42.517338	fig|6666666.34159.peg.2728	peg	NODE_96_length_748272_cov_42.517338_731090_730329	731090	7E+05	-		hypothetical protein		
NODE_96_length_748272_cov_42.517338	fig|6666666.34159.peg.2729	peg	NODE_96_length_748272_cov_42.517338_731073_731201	731073	7E+05	+		hypothetical protein		
NODE_96_length_748272_cov_42.517338	fig|6666666.34159.peg.2730	peg	NODE_96_length_748272_cov_42.517338_731249_732283	731249	7E+05	+		Porphobilinogen synthase (EC 4.2.1.24)	FIG00000348	isu;Heme_and_Siroheme_Biosynthesis
NODE_96_length_748272_cov_42.517338	fig|6666666.34159.peg.2731	peg	NODE_96_length_748272_cov_42.517338_734447_732327	734447	7E+05	-		hypothetical protein	FIG00638284	ff

NODE_96_length_748272_cov_42.517338	fig|6666666.34159.peg.2732	peg	NODE_96_length_748272_cov_42.517338_734794_734621	734794	7E+05	-	hypothetical protein		
NODE_96_length_748272_cov_42.517338	fig|6666666.34159.peg.2733	peg	NODE_96_length_748272_cov_42.517338_734753_736048	734753	7E+05	+	hypothetical protein		
NODE_96_length_748272_cov_42.517338	fig|6666666.34159.peg.2734	peg	NODE_96_length_748272_cov_42.517338_737436_736045	737436	7E+05	-	Na(+)-translocating NADH-quinone reductase subunit A (EC 1.6.5.-)	FIG00001337	isu;Na(+)-translocating_NADH-quinone_oxidoreductase_and_rnf-like_group_of_electron_transport_complexes
NODE_96_length_748272_cov_42.517338	fig|6666666.34159.peg.2735	peg	NODE_96_length_748272_cov_42.517338_738527_737565	738527	7E+05	-	hypothetical protein		
NODE_96_length_748272_cov_42.517338	fig|6666666.34159.peg.2736	peg	NODE_96_length_748272_cov_42.517338_738647_739912	738647	7E+05	+	hypothetical protein	FIG00638284	ff
NODE_96_length_748272_cov_42.517338	fig|6666666.34159.peg.2737	peg	NODE_96_length_748272_cov_42.517338_740553_739873	740553	7E+05	-	pseudouridine synthase		
NODE_96_length_748272_cov_42.517338	fig|6666666.34159.peg.2738	peg	NODE_96_length_748272_cov_42.517338_741383_740550	741383	7E+05	-	macromolecule metabolism; macromolecule synthesis, modification; rna synthesis, modification , dna transcription	FIG00493830	ff
NODE_96_length_748272_cov_42.517338	fig|6666666.34159.peg.2739	peg	NODE_96_length_748272_cov_42.517338_741525_743177	741525	7E+05	+	Adenine deaminase (EC 3.5.4.2)	FIG00001522	isu;Purine_conversions
NODE_96_length_748272_cov_42.517338	fig|6666666.34159.peg.2740	peg	NODE_96_length_748272_cov_42.517338_743356_743174	743356	7E+05	-	hypothetical protein		
NODE_96_length_748272_cov_42.517338	fig|6666666.34159.peg.2741	peg	NODE_96_length_748272_cov_42.517338_743592_743455	743592	7E+05	-	hypothetical protein		
NODE_96_length_748272_cov_42.517338	fig|6666666.34159.peg.2742	peg	NODE_96_length_748272_cov_42.517338_744304_744459	744304	7E+05	+	hypothetical protein		
NODE_96_length_748272_cov_42.517338	fig|6666666.34159.peg.2743	peg	NODE_96_length_748272_cov_42.517338_744887_744432	744887	7E+05	-	hypothetical protein		
NODE_96_length_748272_cov_42.517338	fig|6666666.34159.peg.2744	peg	NODE_96_length_748272_cov_42.517338_744994_745116	744994	7E+05	+	hypothetical protein		
NODE_96_length_748272_cov_42.517338	fig|6666666.34159.peg.2745	peg	NODE_96_length_748272_cov_42.517338_746033_745497	746033	7E+05	-	hypothetical protein		
NODE_96_length_748272_cov_42.517338	fig|6666666.34159.peg.2746	peg	NODE_96_length_748272_cov_42.517338_746028_746231	746028	7E+05	+	hypothetical protein		
NODE_96_length_748272_cov_42.517338	fig|6666666.34159.peg.2747	peg	NODE_96_length_748272_cov_42.517338_746543_746331	746543	7E+05	-	hypothetical protein		
NODE_96_length_748272_cov_42.517338	fig|6666666.34159.peg.2748	peg	NODE_96_length_748272_cov_42.517338_746683_746543	746683	7E+05	-	hypothetical protein		
NODE_96_length_748272_cov_42.517338	fig|6666666.34159.peg.2749	peg	NODE_96_length_748272_cov_42.517338_747751_746720	747751	7E+05	-	hypothetical protein		
NODE_96_length_748272_cov_42.517338	fig|6666666.34159.peg.2750	peg	NODE_96_length_748272_cov_42.517338_747707_748198	747707	7E+05	+	transposase and inactivated derivative		
