# Supplementary material for: Amoebal Endosymbiont Parachlamydia acanthamoebae Bn9 Can Grow in Immortal Human Epithelial HEp-2 Cells at Low Temperature; An In Vitro Model System to Study Chlamydial Evolution
Source: PLoS One. 2015 Feb 2;10(2):e0116486. doi: 10.1371/journal.pone.0116486 (PMC4314085; doi:10.1371/journal.pone.0116486)
Supplement: S2 Table — (PDF) [file pone.0116486.s010.pdf]

Supplementary table 2. Properties with sources of *Parachlamydia* and *Protochlamydia* strains used for comparative genomic analysis

Strain name	Source	Detailed data	Genomic information	Accession number	ATCC assigned name	Reference
Parachlamydia						
Bn_9	Human	Nasal mucosa	Draft genome (this study)	BAWW01000001-BAWW01000072	VR-1476	Amann et al. (1997) [33]
Hall'coccus	Natural environmemt	Waterwest plant	Draft genome	ACZE00000000.1	Unavailable	Greub et al. (2003) [30]
UV-7	Natural environmemt	Water of a humidifier	Completed genome	NC_015702.1	Unavailable	Collingro et al. (2005) [31]
Protochlamydia						
UWE25	Natural environment	Soil	Completed genome	NC_005861.1	PRA-7*	Horn et al. (2004) [2]
R18	Natural environment	Soil	Draft genome (our previous study)	BASL01000001-BASL01000795	Unavailable	Ishida et al. (2014) [32]

*Acanthamoeba UWC1E25 (host amoebae)
